# Screening and validation of nickel ion cytotoxicity biomarkers based on transcriptomic and proteomic technology

**DOI:** 10.1093/rb/rbac073

**Published:** 2022-09-26

**Authors:** Fudan Zhang, Yan Huang, Yajing Zhang, Xiaoying Lü

**Affiliations:** State Key Laboratory of Bioelectronics, School of Biological Science and Medical Engineering, Southeast University, Nanjing 210096, China; State Key Laboratory of Bioelectronics, School of Biological Science and Medical Engineering, Southeast University, Nanjing 210096, China; State Key Laboratory of Bioelectronics, School of Biological Science and Medical Engineering, Southeast University, Nanjing 210096, China; State Key Laboratory of Bioelectronics, School of Biological Science and Medical Engineering, Southeast University, Nanjing 210096, China

**Keywords:** nickel ion (Ni^2+^), cytotoxicity biomarkers, transcriptomic/proteomic, bioinformatics

## Abstract

The aim of this study was to screen cytotoxicity biomarkers of nickel ions (Ni^2+^) using transcriptomic and proteomic approaches combined with molecular biology validation. First, the MTT method was used to evaluate cytotoxicity in L929 cells treated with Ni^2+^ at different concentrations. Ni^2+^ at both 100 μM and 200 μM affected cell proliferation. Then, transcriptomic and proteomic technology was used to study the effects of Ni^2+^ on the expression of genes/proteins in cells. It was found that 1490, 789, 652 and 729 genes (12, 24, 48 and 72 h, respectively) and 177, 2191 and 2095 proteins (12, 24 and 48 h, respectively) were differentially expressed after treatment with 100 μM Ni^2+^. In total, 1403, 963, 916 and 1230 genes (12, 24, 48 and 72 h, respectively) and 83, 1681 and 2398 proteins (12, 24 and 48 h, respectively) were differentially expressed after treatment with 200 μM Ni^2+^. Then, four target gene/protein biomarkers were filtered by combined screening using gene/proteomic experimental data and biological pathway analyses. Further expression level validation of all these target biomarkers and functional validation of selected gene/protein biomarkers were carried out, and a final gene/protein biomarker (UQCRB) was identified.

## Graphical Abstract

**Figure:**
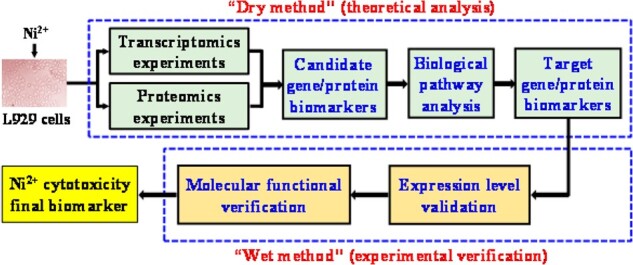

## Introduction

Biomarkers are indicators that can be used to objectively monitor and evaluate normal biological processes, pathological processes and drug intervention reactions [1]. Biomarkers are also important early indicators that warn of damage to an organism, including changes in the cell molecular structure and function; changes in biochemical metabolic processes; the abnormal performance of physiological activities; and abnormal changes in individuals, groups or the entire ecosystem [2]. With the advantages of fast, simple and rapid detection, biomarkers have been applied in medical diagnosis and clinical research, drug screening and evaluation, environmental assessment (ecological risk assessment) and toxicity assessment of biomaterials.

Because of the shape memory effect, nickel and titanium alloys are often used in artificial bones, orthodontic wires, etc. However, nickel–titanium alloys have a high nickel content; thus, after implantation into the human body, nickel ions (Ni^2+^) are released, which leads to certain toxicity. Therefore, it is necessary to study biomarkers that can be used to assess the cytotoxicity of Ni^2+^ early. Clemens *et al*. studied the expression of the Ect2 proto-oncogene and protein in mouse fibroblasts after treatment with Ni^2+^ for 48 h by RT–PCR and northern blot analysis. The results showed that Ect2 can be used as an effective biomarker to monitor changes in cell morphology or tumorigenesis induced by Ni^2+^ or nickel compounds [3]. Kwon *et al*. identified 20 genes (*ACTB*, *AFAP*1, etc.) and 16 proteins (UTS2, ACTB, *etc.*) as potential biomarkers that can be used to test nickel toxicity by a gene expression microarray, functional proteomics and pathway analysis [4].

The above-mentioned studies investigating Ni^2+^ toxicity biomarkers were carried out by either traditional molecular biology methods or a single biomics method, and joint analyses based on multiomics methods are lacking; this has led to the problem that biomarkers have been inconsistently screened at different levels. Furthermore, the above studies only describe bioinformatics analyses of the experimental results of biomics (‘dry method’) and reveal the theoretical functions of biomarkers and do not report a series of verification experiments (‘wet method’) to validate the screened biomarkers. Therefore, biomarker investigation is still at the ‘on-paper’ stage.

Regarding the issue above, the aim of this study was to use transcriptomic and proteomic technologies combined with bioinformatics analyses to screen cytotoxicity biomarkers of Ni^2+^ to avoid the inconsistency resulting from the screening of biomarkers using different biomics platforms. Then, a series of molecular biology experiments were carried out to verify the expression levels and function levels of the screened biomarkers to ensure the reliability of the biomics experiments and bioinformatics analysis. First, transcriptomic and proteomic methods were used to study the effects of Ni^2+^ on the expression of genes/proteins in L929 cells. Then, the transcriptomic/proteomic data were jointly screened, and genes/proteins that were differentially expressed in multiple experimental groups with expression patterns that were basically consistent at the mRNA and protein levels were selected as candidate gene/protein biomarkers (‘dry method’). Furthermore, a biological pathway analysis of these candidate biomarkers was performed to identify cytotoxicity-related pathways enriched in the target gene/protein biomarkers. Finally, qRT–PCR and western blot analysis were used to verify the expression levels of the target gene/protein biomarkers. The functions of the target gene/protein biomarkers were verified by assays to determine the cell proliferation rate, oxidative stress and the mitochondrial membrane potential (MMP) (‘wet method’), and the final gene/protein biomarkers of Ni^2+^ cytotoxicity were determined.

## Materials and methods

### Preparation of materials

Hexahydrate nickel chloride powders (National Medicine Group Chemical Reagent Co., China) were dissolved in RPMI-1640 complete medium (89% RPMI-1640 (Gibco, USA), 10% fetal bovine serum (Hangzhou Sijiqing Bioengineering Co., China) and 1% penicillin–streptomycin (Gibco)) to a final concentration of 100 μM and 200 μM Ni^2+^.

### Cell culture

L929 mouse fibroblast cells were purchased from the Shanghai Cell Bank of the Chinese Academy of Sciences and cultured in complete RPMI-1640 medium at 37°C under 5% CO_2_ and saturated humidity. All experiments were performed with cells in the logarithmic growth phase.

### MTT assay to determine the cell cytotoxicity of Ni^2+^

Nickel chloride hexahydrate powder (2.376 g) was weighed and dissolved in 100 ml of complete RPMI-1640 medium to obtain 0.1 M Ni^2+^. Then, 0.1 ml of the stock solution was diluted 1000-fold and 500-fold with complete RPMI-1640 medium to obtain 100 μM and 200 μM Ni^2+^, respectively.

The *in vitro* cytotoxicity of 100 μM and 200 μM Ni^2+^ in L929 cells after 12, 24, 48 and 72 h of treatment was examined using a MTT assay as described in a previous study [5]. Cells that were not treated with Ni^2+^ served as a negative control, and cells that were treated with 0.7% acrylamide in complete RPMI-1640 medium served as a positive control. The sample size was six.

### Biomics experiments

#### Transcriptomics experiments

L929 cells were cultured in a cell culture flask with a bottom surface area of 75 cm^2^. The number of seeded cells was 4 × 10^6^, 3 × 10^6^, 1.5 × 10^6^ or 10^6^ in the experimental groups (cells treated with 100 μM or 200 μM Ni^2+^ for 12, 24, 48 or 72 h) and 10^6^ in the control group (cells not treated with Ni^2+^ and cultured for 72 h). After 24 h, the culture medium in the experimental groups was replaced with medium containing 100 μM or 200 μM Ni^2+^, and the cells were cultured for another 12, 24, 48 or 72 h.

After culture for a given duration, the cells were lysed, total RNA was extracted and transcriptome sequencing experiments were performed by Shanghai Omicsspace Biotech Co., Ltd. (Shanghai, China). The experimental process is briefly described as follows: mRNA purification and reverse transcription were performed, and then, the constructed library was used for quality inspection. Then, sequencing experiments were carried out, and the gene expression in each group was analyzed. Genes with a log_2_ fold change (log_2_FC) > 1 were defined as upregulated, and those with a log_2_FC < –1 were defined as downregulated. The sample size was three.

#### Proteomics experiments

iTRAQ-based proteomics technology was used to study the protein expression profile in L929 cells after treatment with 100 μM Ni^2+^ for 12, 24 or 48 h in our previous research, and the differentially expressed proteins in each Ni^2+^-treated group were identified [6]. In this study, the effect of 200 μM Ni^2+^ on protein expression profiles was further investigated. The sample size was three.

### Screening of target gene/protein biomarkers

#### Screening of candidate gene/protein biomarkers

The differentially expressed genes and proteins whose screening is described in the ‘Biomics experiments’ Section were analyzed, and the genes/proteins that were coexpressed in five or more groups of Ni^2+^-treated L929 cells were selected. Further joint analysis of the differentially expressed genes and proteins was performed, and those genes/proteins that were differentially expressed at both the mRNA and protein levels with basically consistent expression patterns were screened as candidate biomarkers.

#### Determination of target gene/protein biomarkers

DAVID (http://david.abcc.ncifcrf.gov/) was used to perform a KEGG biological pathway analysis of the candidate gene/protein biomarkers, and those enriched in Ni^2+^ cytotoxicity-related pathways were determined to be target gene/protein biomarkers.

### Validation of the expression level of target gene/protein biomarkers

#### qRT–PCR

After L929 cells were treated with 100 or 200 μM Ni^2+^ for 12, 24, 48 or 72 h, total RNA was extracted, and the expression levels of four genes (*Uqcrb*, *Csf1*, *Cth* and *Acot2*) were detected with qRT–PCR. Cells that were not treated with Ni^2+^ and cultured for 72 h served as the control. The sample size was three.

#### Western blot analysis

After L929 cells were treated with 100 or 200 μM Ni^2+^ for 12, 24, 48 or 72 h, the total protein was extracted, and the expression levels of four proteins (ubiquinol-cytochrome c reductase-binding protein (UQCRB), colony stimulating factor 1 (CSF1), cystathionase (CTH) and ACOT2) were detected by western blot analysis. GAPDH was used as an internal control. Cells that were not treated with Ni^2+^ and cultured for 72 h served as the control. The sample size was three.

### Functional validation of gene/protein biomarkers

#### Construction of stable gene interference/overexpression cell lines

Primers were designed based on the sequences of *Uqcrb* and *Csf1* and synthesized, and then, target gene lentivirus interference/overexpression vectors were constructed. The lentiviruses were then subjected to packaging and viral titer determination, followed by lentivirus infection and drug screening. qRT–PCR and western blot analysis were used to detect the expression of the target genes and proteins to verify the effectiveness of target gene interference or overexpression. The sample size was three. Cell lines with stable gene interference were constructed by Shanghai Omicsspace Biotech Co., Ltd., and cell lines with stable gene overexpression were constructed by Zhenjiang Aibimeng Biotechnology Co., Ltd.

#### Validation experiments

##### Cell proliferation assay.

The MTT method was used to evaluate the effects of 100 and 200 μM Ni^2+^ on cell proliferation after different durations (12, 24, 48 and 72 h) in different cells (normal L929 cells, *Csf1*-silenced L929 cells and *Csf1*-overexpressing L929 cells). The experimental process was the same as that described in the Section ‘MTT assay to determine the cell cytotoxicity of Ni^2+^’. Normal L929 cells that were not treated with Ni^2+^ served as the control. The sample size was six.

##### Oxidative stress analysis.

Normal L929 cells, *Uqcrb*-silenced L929 cells and *Uqcrb*-overexpressing L929 cells were seeded in 96-well plates and then treated with 100 or 200 μM Ni^2+^ for 12, 24, 48 or 72 h. Normal L929 cells that were not treated with Ni^2+^ served as the control. After the cells were cultured for a given time, Dihydroethidium (DHE) and Hoechst staining were performed in sequence, the fluorescence intensity of ethidium bromide was measured by a high-content cell analysis system (Cellomics ArrayScan VTI HCS Reader, Thermo Fisher, USA) and the cells were counted. The average fluorescence intensity of ethidium bromide was used to evaluate the level of superoxide anion in the cells [7]. The sample size was six.

##### MMP detection.

Ni^2+^ treatment was applied as described for the oxidative stress analysis experiment [8]. After the cells had been cultured for a given time (12, 24, 48 or 72 h), JC-1 and Hoechst staining was performed in turn, and a high-content cell analysis system was used to determine the fluorescence intensities of the JC-1 monomer (green fluorescence) and polymer (red fluorescence). The ratio of green to red fluorescence was used to evaluate the MMP. Normal L929 cells that were not treated with Ni^2+^ served as the control. The sample size was six.

### Statistical analysis

The data are expressed as the mean ± SD of at least three independent experiments. Unless stated otherwise, statistical analysis was performed using Student’s t test. The results were considered statistically significant when *P *<* *0.05 and highly significant when *P *<* *0.01.

## Results and discussion

### Results of the cell cytotoxicity assay

**Figure 1.**
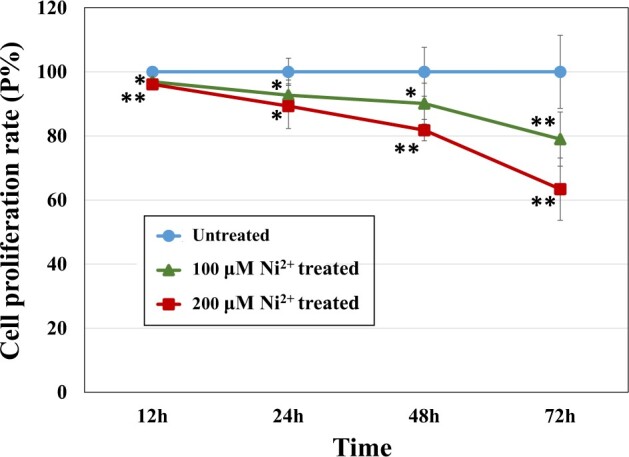
Proliferation of L929 cells treated with 100 or 200 μM Ni^2+^ for different durations. For toxicity grades, see Reference [5]. Toxicity Grade 0 means Pϵ [81%, 100%], Grade 1 means Pϵ [61%, 80%], Grade 2 means Pϵ [41%, 60%], Grade 3 means Pϵ [21%, 40%] and Grade 4 means Pϵ [0, 20%]. *n* = 6, *P <* *0.05, **P <* *0.01.

Figure 1 shows the results of the cell proliferation assay of L929 cells treated with 100 or 200 μM Ni^2+^ for different durations (12, 24, 48 or 72 h). The cell proliferation rate of the Ni^2+^-treated groups decreased as the Ni^2+^ concentration and action time increased. Grade 1 cytotoxicity occurred after 72 h of Ni^2+^ treatment, indicating that Ni^2+^ impacted cell proliferation. This result is consistent with our previous studies [5, 9].

### Biomics experiments

#### Results of the transcriptomics experiments

The differentially expressed genes in L929 cells after 12, 24, 48 or 72 h of treatment with 100 or 200 μM Ni^2+^ compared to control L929 cells are shown in Table 1, and detailed information is shown in Supplementary Tables S1 and S2.

**Table 1.** Number of differentially expressed genes in L929 cells treated with Ni^2+^ for different durations

Group	Upregulated genes	Downregulated genes	Total differentially expressed genes
100 μM-12 h	413	1077	1490
100 μM-24 h	322	467	789
100 μM-48 h	324	328	652
100 μM-72 h	381	348	729
200 μM-12 h	454	949	1403
200 μM-24 h	539	424	963
200 μM-48 h	494	422	916
200 μM-72 h	627	603	1230

#### Results of the proteomics experiments

Using proteomics technology, it was found that 177, 2191 and 2095 proteins were differentially expressed after 12, 24 and 48 h of exposure to 100 μM Ni^2+^, respectively, in our previous study [6]. Here, 83, 1681 and 2398 proteins were differentially expressed after treatment with 200 μM Ni^2+^ for 12, 24 and 48 h, respectively (Table 2). Detailed information is shown in Supplementary Table S3.

**Table 2.** Number of differentially expressed proteins in L929 cells treated with Ni^2+^ for different durations

Group	Upregulated proteins	Downregulated proteins	Total differentially expressed proteins
100 μM-12 h	16	161	177
100 μM-24 h	2	2189	2191
100 μM-48 h	11	2084	2095
200 μM-12 h	23	60	83
200 μM-24 h	9	1672	1681
200 μM-48 h	6	2392	2398

### Screening of target gene/protein biomarkers

#### Screening of candidate gene/protein biomarkers

The differentially expressed genes and proteins identified in the ‘Biomics experiments’ Section were screened. A total of 127 genes were coexpressed in any five of the 8 Ni^2+^-treated L929 cell groups, 106 genes were coexpressed in any six of the 8 Ni^2+^-treated L929 cell groups, 100 genes were coexpressed in any seven of the 8 Ni^2+^-treated L929 cell groups and 147 genes were coexpressed in all eight Ni^2+^-treated L929 cell groups (as listed in Table 1), yielding a total of 480 genes. Detailed information is shown in Supplementary Table S4. A total of 119 proteins were coexpressed in any five of the 6 Ni^2+^-treated L929 cell groups and 31 proteins were coexpressed in all six Ni^2+^-treated L929 cell groups (as listed in Table 2), for a total of 150. Detailed information is shown in Supplementary Table S5.

The above-mentioned 480 differentially expressed genes and 150 differentially expressed proteins were further compared, and in total, 12 gene/protein pairs that were differentially expressed at both the mRNA and protein levels were screened. Their relative expression values are shown in Table 3. The expression patterns of CSF1, Acyl-CoA thioesterase 2 (ACOT2), FAH, LBH and HYOU1 were the same at the mRNA and protein levels and were downregulated (Table 3, Nos. 1–5). UQCRB and CTH were downregulated at the protein level and mainly downregulated at the mRNA level (Table 3, Nos. 6–7). HIST1H1C, FDPS, LSS, PMVK and NUCB22 exhibited opposite changes in expression upon Ni^2+^ treatment at the mRNA level (upregulated) and protein level (downregulated) (Table 3, Nos. 8–12). This might be because mRNA produced after gene transcription is finely regulated in terms of its processing, maturation, degradation and translation initiation. Among them, a series of small RNA molecules, such as microRNAs and small interfering RNAs, have been found to have a regulatory role in the process by which mRNA is translated into protein, which results in the absence of a quantitative relationship or a weak or even an opposite correlation in expression [10]. Therefore, CSF1, ACOT2, FAH, LBH, HYOU1, UQCRB and CTH were selected as candidate gene/protein biomarkers.

**Table 3.** Relative expression values of gene/protein pairs determined in the transcriptomic and proteomic experiments (*n* = 3, mean values are listed in the table)

No.	Official gene symbol	Experiment	100μM				200μM					
			12h	24h	48h	72h	12h		24h	48h		72h
1	CSF1	Transcriptomics			0.46↓	0.49↓			0.38↓	0.36↓		0.49↓
		Proteomics	0.73↓	0.23↓	0.55↓		0.76↓		0.31↓	0.40↓		
2	ACOT2	Transcriptomics	0.42↓		0.49↓	0.47↓	0.46↓					0.48↓
		Proteomics	0.75↓	0.42↓	0.64↓		0.80↓		0.48↓	0.62↓		
3	FAH	Transcriptomics	0.33↓	0.47↓	0.48↓	0.49↓	0.40↓					
		Proteomics	0.78↓	0.56↓	0.69↓				0.63↓	0.53↓		
4	LBH	Transcriptomics	0.48↓				0.46 ↓		0.49↓	0.49↓		0.46↓
		Proteomics	0.77↓	0.68↓	0.55↓		0.78↓		0.71↓	0.47↓		
5	HYOU1	Transcriptomics	0.25↓	0.40↓	0.45↓	0.42↓	0.31↓					
		Proteomics	0.83↓	0.48↓	0.72↓				0.59↓	0.65↓		
6	UQCRB	Transcriptomics	2.15↑	2.36↑		0.42↓	0.49↓		0.46↓			
		Proteomics	0.81↓	0.38↓	0.62↓		0.81↓		0.44↓	0.52↓		
7	CTH	Transcriptomics	2.13↑	2.28↑	0.34↓	0.35↓						0.47↓
		Proteomics	0.69↓	0.39↓	0.53↓		0.73↓		0.49↓	0.50↓		
8	HIST1H1C	Transcriptomics	2.99↑	2.14↑		2.03↑	2.66↑		2.23↑			2.01↑
		Proteomics	0.76↓	0.46↓	0.71↓				0.49↓	0.56↓		
9	FDPS	Transcriptomics	3.52↑	2.68↑			3.03↑		3.27↑	2.02↑		2.46↑
		Proteomics	0.76↓	0.37↓	0.57↓		0.82↓		0.40↓	0.46↓		
10	LSS	Transcriptomics	2.78↑	2.65↑			3.00↑		3.00↑	2.33↑		2.95↑
		Proteomics	0.81↓	0.49↓	0.60↓		0.81↓		0.62↓	0.50↓		
11	PMVK	Transcriptomics	2.99↑	2.44↑			2.93↑		3.10↑	2.17↑		2.32↑
		Proteomics	0.78↓	0.21↓	0.56↓				0.26↓	0.40↓		
12	NUCB2	Transcriptomics		2.14↑	2.06↑	4.06↑	0.46↓			2.03↑	4.41↑	
		Proteomics	0.79↓	0.49↓	0.68↓			0.60↓		0.64↓		

#### Determination of target gene/protein biomarkers

To further screen the target gene/protein biomarkers of Ni^2+^ cytotoxicity, a KEGG biological pathway analysis of the seven obtained candidate gene/protein biomarkers was performed (Table 3, Nos. 1–7). Detailed information is shown in Supplementary Table S6. According to previous research related to the cytotoxicity mechanism of Ni^2+^ by our group, Ni^2+^ mainly exerts its toxic effects by affecting the following factors: (i) the cytoskeleton and cell adhesion, (ii) cell growth and proliferation, (iii) the expression of related genes and proteins in cells, (iv) intracellular energy metabolism and (v) oxidative stress and inflammation [5, 9]. Therefore, the genes/proteins involved in the pathways related to Ni^2+^ cytotoxicity were screened according to the above factors. In total, four of the seven candidate gene/protein biomarkers in Table 3 were found to participate in pathways related to Ni^2+^ cytotoxicity (Table 4), while the other three did not participate in such pathways; thus, these four candidate gene/protein biomarkers (CSF1, CTH, ACOT2 and UQCRB) were determined to be target biomarkers.

**Table 4.** Target gene/protein biomarkers involved in pathways related to Ni^2+^ cytotoxicity

No.	Target gene/protein biomarker	Pathway
1	CSF1	Cytokine–cytokine receptor interaction MAPK signaling pathway Ras signaling pathway PI3K-Akt signaling pathway Rap1 signaling pathway TNF signaling pathway
2	CTH	Biosynthesis of amino acids Cysteine and methionine metabolism Glycine, serine and threonine metabolism
3	ACOT2	Biosynthesis of unsaturated fatty acids Fatty acid elongation
4	UQCRB	Oxidative phosphorylation

**Figure 2.**
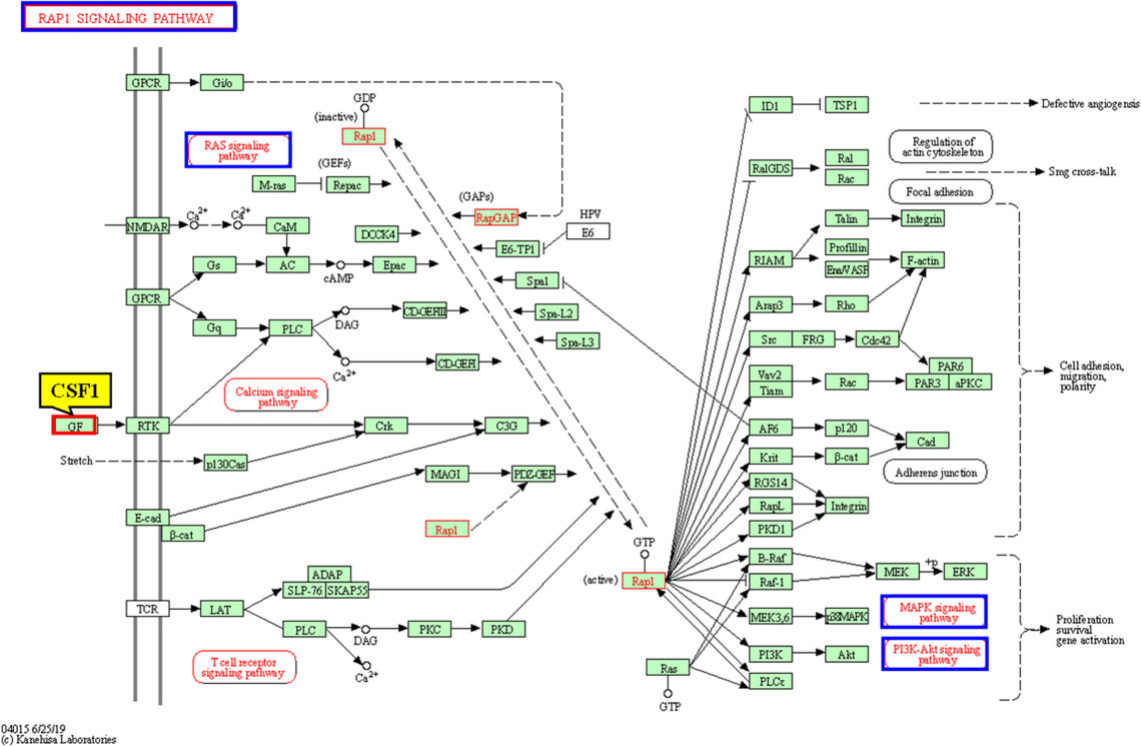
The Rap1 signaling pathway in which CSF1 is involved [14].

Table 4 shows that CSF1 participates in the following six pathways related to Ni^2+^ cytotoxicity: (i) the cytokine–cytokine receptor interaction pathway (cytokines are important cell regulators and drivers of cell biological activities that induce responses by binding specific receptors on the surface of target cells and have functions such as regulating cell growth, differentiation, immune activity and inflammatory responses) [11], (ii) Rap1 signaling pathway (Fig. 2), (iii) Ras signaling pathway, (iv) PI3K-Akt signaling pathway, (v) Mitogen-activated protein kinase (MAPK) signaling pathway and (vi) tumor necrosis factor (TNF) signaling pathway. The latter five pathways are connected to each other. Rap1 is a small GTPase that controls various cellular processes, such as cell adhesion and cell polarity. Rap1 controls cell–cell and cell–cell–matrix interactions by regulating the functions of integrins and other adhesion molecules in different cell types. Furthermore, Rap1 can regulate MAP kinase activity [12]. Ras and PI(3)K are protein kinases that are closely related to cell proliferation. Ras and PI(3)K signaling plays a key role in regulating cell growth by regulating the downstream molecule mTOR [13]. The basic MAPK pathway is structured as a three-level kinase model consisting of MAP kinase, MAPK kinase and Mitogen-activated protein kinse-ERK kinase (MEK). The activation of these three kinases together regulates several important cellular physiological/pathological processes, including cell growth, differentiation, adaptation to environmental stress and the inflammatory response. As an important cytokine, TNF can induce the activation of various intracellular signaling pathways, including apoptosis, cell survival, inflammation and immunity. Therefore, the above indicates that CSF1 met the screening conditions for Ni^2+^ cytotoxicity markers.

**Figure 3.**
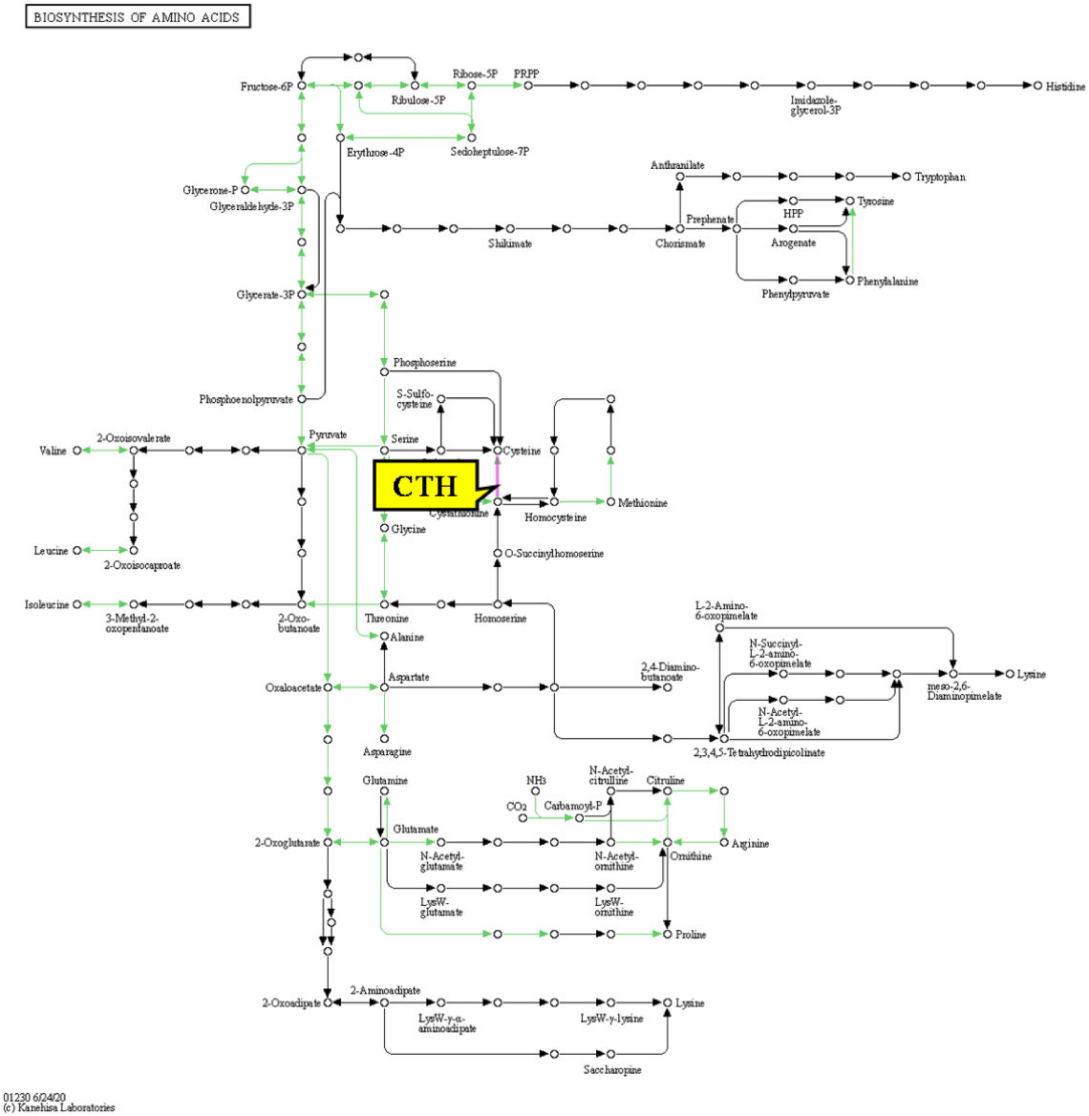
The biosynthesis of amino acids pathway in which CTH is involved [14].

CTH participated in three cytotoxicity-related pathways (biosynthesis of amino acids pathway (Fig. 3); cysteine and methionine metabolism pathway; and glycine, serine and threonine metabolism pathway), all of which are amino acid metabolism pathways. Amino acid metabolism is very important for biological activities. In this process, digested and decomposed amino acids from ingested proteins are used to synthesize tissue proteins, and amino acids can be used to synthesize other nitrogenous substances useful for the human body or decomposed to provide energy.

ACOT2 was found to be involved in two cytotoxicity-related pathways (biosynthesis of unsaturated fatty acids pathway and fatty acid elongation pathway). Fatty acids are the main components of neutral fats, phospholipids and glycolipids. With the help of various related enzymes, fat is digested, absorbed, synthesized, decomposed and processed into the substances needed by the body to ensure normal physiological function. Lipids are important substances for energy storage and energy supply in the body and important structural components of biological membranes. Therefore, fat metabolism is of great importance to biological activities.

UQCRB was found to participate in one cytotoxicity-related pathway (the oxidative phosphorylation pathway), which belongs to the energy metabolism pathway. Oxidative phosphorylation is the coupling of energy released when substances are oxidized in the body to supply ADP and inorganic phosphorus to synthesize ATP through the respiratory chain.

In conclusion, CSF1, CTH, ACOT2 and UQCRB were all shown to be involved in Ni^2+^ cytotoxicity-related pathways; thus, they were identified as target gene/protein biomarkers of Ni^2+^ cytotoxicity. The other three candidate gene/protein biomarkers (FAH, LBH and HYOU1) did not participate in a Ni^2+^ cytotoxicity-related pathway, so they were not used as target biomarkers and are not discussed further.

### Validation of the expression of target gene/protein biomarkers

#### qRT–PCR

**Figure 4.**
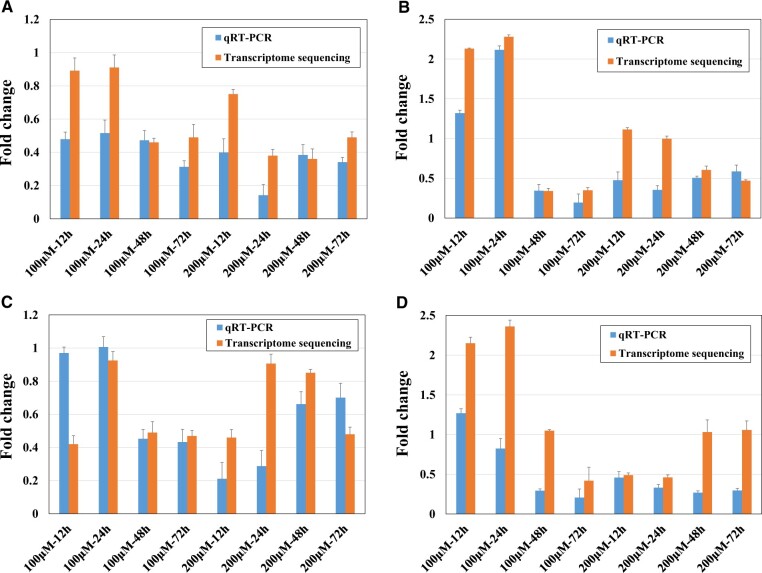
qRT–PCR analysis of four target gene markers and comparison with transcriptome sequencing results: (**A**) *Csf1*, (**B**) *Cth*, (**C**) *Acot2*, (**D**) *Uqcrb*. *n* = 3.

Figure 4 shows the results of qRT–PCR analysis of the expression levels of the four target gene markers (*Csf1*, *Cth*, *Acot2* and *Uqcrb*) in L929 cells after 12, 24, 48 or 72 h of treatment with 100 or 200 μM Ni^2+^, and the results were compared with the transcriptome sequencing results. The qRT–PCR results showed that the four genes were downregulated in six or more Ni^2+^-treated groups, and the *Csf1* gene was downregulated in all groups. Therefore, the expression patterns of these four target biomarkers at the mRNA level were consistent, so further experiments to verify their expression patterns at the protein level were carried out.

#### Western blot analysis

**Figure 5.**
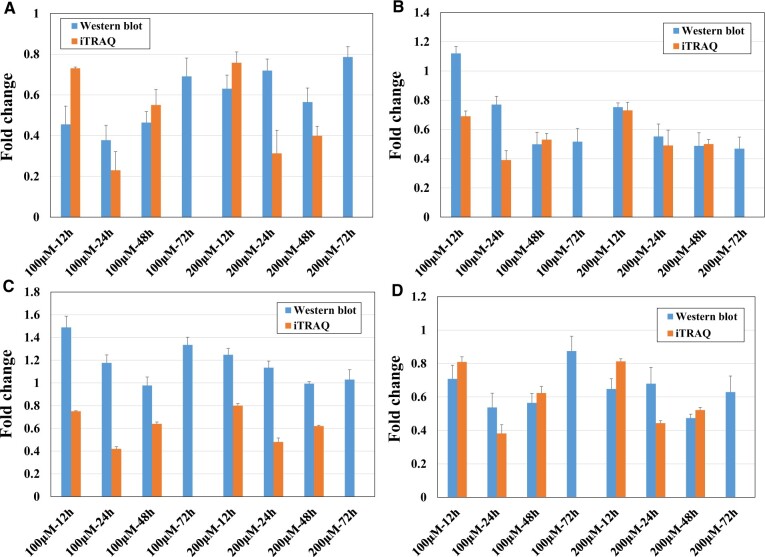
Western blot analysis of four target protein biomarkers and comparison with the proteomics results: (**A**) CSF1, (**B**) CTH, (**C**) ACOT2, (**D**) UQCRB. *n* = 3.

Figure 5 shows the results of western blot analysis of the expression levels of the four target protein markers (CSF1, CTH, ACOT2 and UQCRB) in L929 cells after 12, 24, 48 or 72 h of treatment with 100 or 200 μM Ni^2+^, and the results were compared with the proteomics results. The gel electropherogram and semiquantitative data are shown in Supplementary Fig. S1 and Table S7–S10.

The expression patterns of three proteins (CSF1, CTH and UQCRB) determined by western blot analysis were basically consistent with the results of the proteomics experiment, which showed downregulation. However, the expression patterns of ACOT2 determined by the two experiments were inconsistent. Therefore, the three target protein biomarkers (CSF1, CTH and UQCRB) whose expression patterns were consistent are discussed next.

CSF1 is a cytokine that is expressed in many cell types and plays a crucial role in cell survival, proliferation and differentiation [15]. CSF1 usually binds CSF1R, and the CSF1/CSF1R signaling pathway can activate the MAPK signaling pathway to promote cell proliferation or differentiation [16]. Therefore, we further verified the function of CSF1 by a cell proliferation assay.

CTH is mainly expressed in cells as cystathionine γ-lyase (CSE). In the liver, CSE is the source of H_2_S, which can regulate the production of glucose, in the blood vessels [17]. Since the function of CTH is not directly related to Ni^2+^ cytotoxicity, the function of CTH was not verified.

UQCRB is a part of the mitochondrial respiratory chain and plays an important role in electron transport and redox-related proton transport. Jung *et al*. studied the effect of UQCRB on the oxygen-sensing mechanism under conditions of overexpression/interference and found that a change in the UQCRB expression level could regulate the ROS content in cells and affect electron transfer [18]. Therefore, the function of UQCRB was further verified by detection of the ROS level and MMP.

### Functional validation of the gene/protein biomarkers

The above experiments to verify expression patterns ensured that the results of experiments used to determine expression of the selected target gene/protein biomarkers were correct and reliable. However, whether the changes in their expression levels and cell function are related could not be determined by purely theoretical analysis. Therefore, it was necessary to combine the ‘dry method’ (theoretical analysis) with the ‘wet method’ (experimental verification). The possible molecular functions of the biomarkers determined by bioinformatics analysis were verified by cellular/molecular biology experiments to determine whether they play a specific role in Ni^2+^-induced cytotoxicity and their mechanisms. Since RNA interference and overexpression can significantly downregulate and upregulate the expression levels of specific genes in cells, cell biology experiments can be used to study whether such downregulation and upregulation lead to corresponding changes in cell function to determine whether the gene can regulate cell function; thus, this technology is often used to study and verify the functions of specific genes [19–21].

In this study, RNA interference/overexpression technology was first used to construct stable cell lines with *Csf1* and *Uqcrb* interference/overexpression. Then, through detecting cell proliferation, oxidative stress and the MMP, we investigated whether the downregulation and upregulation of these two genes were related with the changes in cell function. The functions of the two target biomarkers were finally verified.

#### Construction of stable gene interference/overexpression cell lines

**Figure 6.**
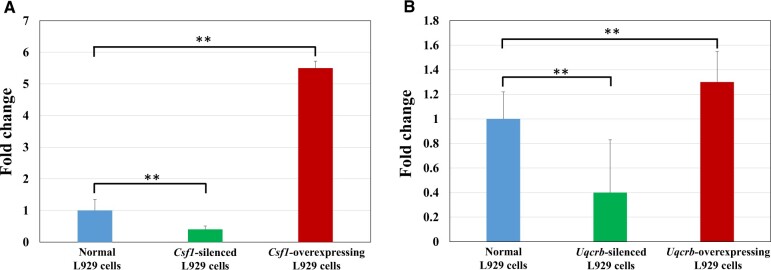
(**A**) Expression levels of *Csf1* in normal L929 cells, *Csf1*-silenced L929 cells and *Csf1*-overexpressing L929 cells, (**B**) expression levels of *Uqcrb* in normal L929 cells, *Uqcrb*-silenced L929 cells and *Uqcrb*- overexpressing L929 cells. *n* = 3, **P <* *0.01.

The expression of *Csf1* and *Uqcrb* in normal L929 cells, L929 cells with stable gene interference and L929 cells with stable overexpression was verified by qRT–PCR, and the results are shown in Fig. 6.

**Figure 7.**
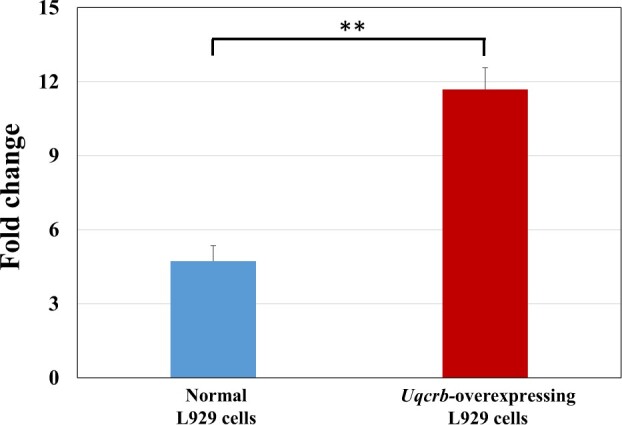
Expression level of the UQCRB protein in normal L929 cells and *Uqcrb*-overexpressing L929 cells, *n* = 3, **P <* *0.01.

It is generally believed that when target gene expression levels in cells after RNA interference and overexpression decrease by 50% or increase by more than 2-fold, respectively, interference and overexpression have been successful. Figure 6 shows that the *Csf1* expression value in the interference group was 60% lower than that in the normal group and that the *Csf1* expression value in the overexpression group was 450% higher than that in the normal group, implying that the *Csf1-*silenced and *Csf1-*overexpressing cell lines had been successfully constructed. The expression level of *Uqcrb* in the interference group was 60% lower than that in the normal group, but the expression level of *Uqcrb* in the overexpression group was only 30% higher than that in the normal group, suggesting that the efficiency of gene overexpression was not satisfactory. Therefore, the protein expression level of UQCRB in normal L929 cells and L929 cells with stable *Uqcrb* overexpression was further determined by western blot analysis (Fig. 7). The UQCRB protein content in the overexpression group was more than two times higher than that in the normal cells. Combined with the results of the qRT–PCR and western blot experiments, these results suggested that the *Uqcrb-*silenced and *Uqcrb-*overexpression cell lines had been successfully constructed.

#### Validation experiments

##### Cell proliferation assay.

**Figure 8.**
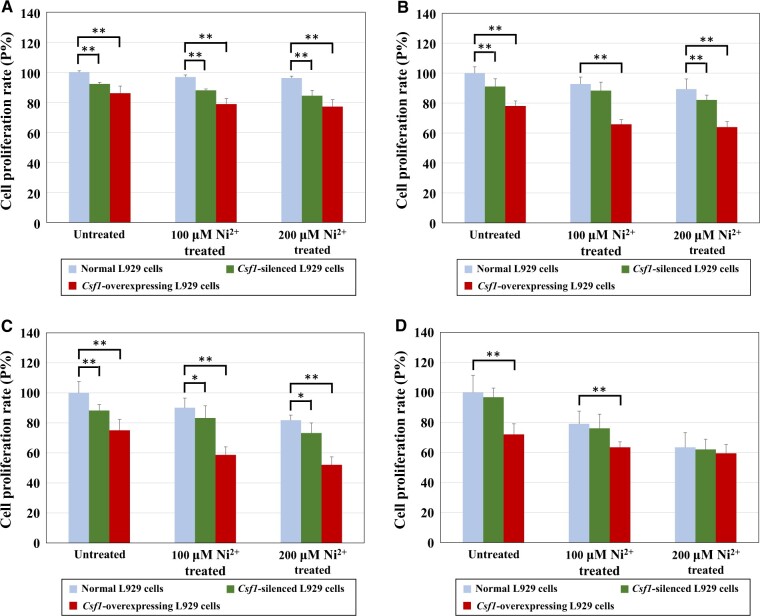
The cell proliferation rate results of normal L929 cells, *Csf1*-silenced L929 cells and *Csf1*-overexpressing L929 cells after treatment with 100 or 200 μM Ni^2+^ for 12 h (**A**), 24 h (**B**), 48 h (**C**) or 72 h (**D**). *n* = 6, *P <* *0.05, **P <* *0.01.

Figure 8 shows the cell proliferation rates of three types of L929 cells (normal L929 cells, *Csf1*-silenced L929 cells and *Csf1*-overexpressing L929 cells) after treatment with 100 or 200 μM Ni^2+^ for different durations (12, 24, 48 or 72 h). As the Ni^2+^ concentration increased, the cell proliferation rate of each Ni^2+^-treated group gradually decreased in the same type of cells, and all experimental groups showed evidence of cytotoxicity after 72 h of Ni^2+^-treatment (*P* < 80%). These results showed that Ni^2+^ could affect cell proliferation and was cytotoxic and were consistent with our previous conclusions [5, 9].

Further analysis showed that the cell proliferation rates of the *Csf1-silenced*/*Csf1-*overexpressing L929 cells were lower than those of the normal cells. Thus, both the downregulation and upregulation of the *Csf1* gene led to a decrease in the proliferation rate of the L929 cells, indicating that the biological function of the *Csf1* gene is not necessarily related to cell proliferation. Shen *et al*. indicated that Ni^2+^ toxicity and processes such as cell adhesion and migration may be connected [22]. Therefore, the CSF1 gene/protein was not selected as a biomarker for assessing Ni^2+^ cytotoxicity.

##### Oxidative stress analysis.

**Figure 9.**
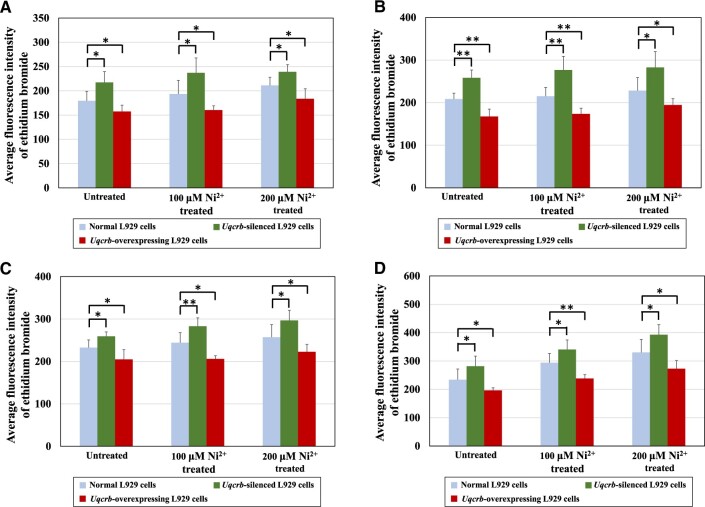
Oxidative stress analysis of normal L929 cells, *Uqcrb*-silenced L929 cells and *Uqcrb*-overexpressing L929 cells after treatment with 100 or 200 μM Ni^2+^ for 12 h (**A**), 24 h (**B**), 48 h (**C**) or 72 h (**D**). *n* = 6, *P <* *0.05.

Figure 9 shows the mean fluorescence intensity of ethidium bromide in three types of L929 cells (normal L929 cells, *Uqcrb-*silenced L929 cells and *Uqcrb-*overexpressing L929 cells) after treatment with 100 or 200 μM Ni^2+^ for different durations (12, 24, 48 or 72 h). The higher the fluorescence intensity was, the stronger the oxidative stress was. As the Ni^2+^ concentration and exposure time increased, the degree of oxidative stress in each Ni^2+^-treated group increased in the same type of L929 cells. These results showed that Ni^2+^ could induce oxidative stress, which is also consistent with our previous conclusions [9].

Further analysis revealed that after treatment with Ni^2+^ at two concentrations for different durations, the oxidative stress level in the *Uqcrb*-silenced L929 cells was significantly higher than that in normal cells (*P *<* *0.05), while the oxidative stress level in the *Uqcrb-*overexpressing L929 cells was significantly lower than that in the normal cells (*P *<* *0.05). These results indicated that the downregulation and upregulation of the *Uqcrb* gene led to an increase or decrease, respectively, in the level of intracellular ROS, suggesting that the *Uqcrb* gene plays a role in regulating intracellular ROS.

##### MMP detection.

**Figure 10.**
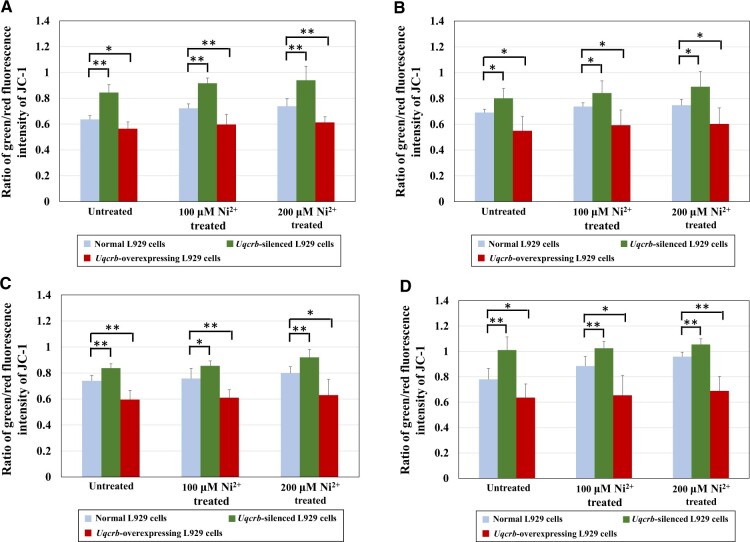
MMP detection in normal L929 cells, *Uqcrb*-silenced L929 cells and *Uqcrb*-overexpressing L929 cells after treatment with 100 or 200 μM Ni^2+^ for 12 h (**A**), 24 h (**B**), 48 h (**C**) or 72 h (**D**). *n* = 6, *P <* *0.05, **P <* *0.01.

Figure 10 shows the ratio of the green/red fluorescence intensity of JC-1 in three types of L929 cells (normal L929 cells, *Uqcrb*-silenced L929 cells and *Uqcrb*-overexpressing L929 cells) after treatment with 100 or 200 μM Ni^2+^ for different durations (12, 24, 48 or 72 h); the higher the ratio was, the lower the MMP was. As the Ni^2+^ concentration increased, the MMP in each Ni^2+^-treated group decreased (the ratio of green/red fluorescence intensity increased) in the same type of cells. These results showed that Ni^2+^ could lead to a decrease in the MPP.

Further analysis revealed that after treatment with Ni^2+^ at two concentrations for different durations, the MMP in the *Uqcrb*-silenced L929 cells was significantly (*P *<* *0.05) or highly significantly (*P *<* *0.01) lower than that in the normal cells, while the MMP in the *Uqcrb*-overexpressing L929 cells was significantly (*P *<* *0.05) or highly significantly (*P *<* *0.01) higher than that in the normal cells. These results indicated that the downregulation/upregulation of the *Uqcrb* gene could lead to a decrease/increase in the MMP, proving that the *Uqcrb* gene plays a role in regulating the MMP.

The above indicated that the *Uqcrb* gene regulates the ROS content and MMP in cells, and these biological functions are related to the biological process involved in Ni^2+^ cytotoxicity.

Hong *et al*. and Kim *et al*. demonstrated that UQCRB expression in colorectal cancer cells and tissues was correlated with colorectal cancer progression; thus, UQCRB is a biomarker for diagnosing colorectal cancer [23, 24]. Based on the above results of expression to verify the expression levels and function of this biomarker, the UQCRB gene/protein could serve as a final biomarker for evaluating Ni^2+^ cytotoxicity.

## Conclusion

**Figure 11.**
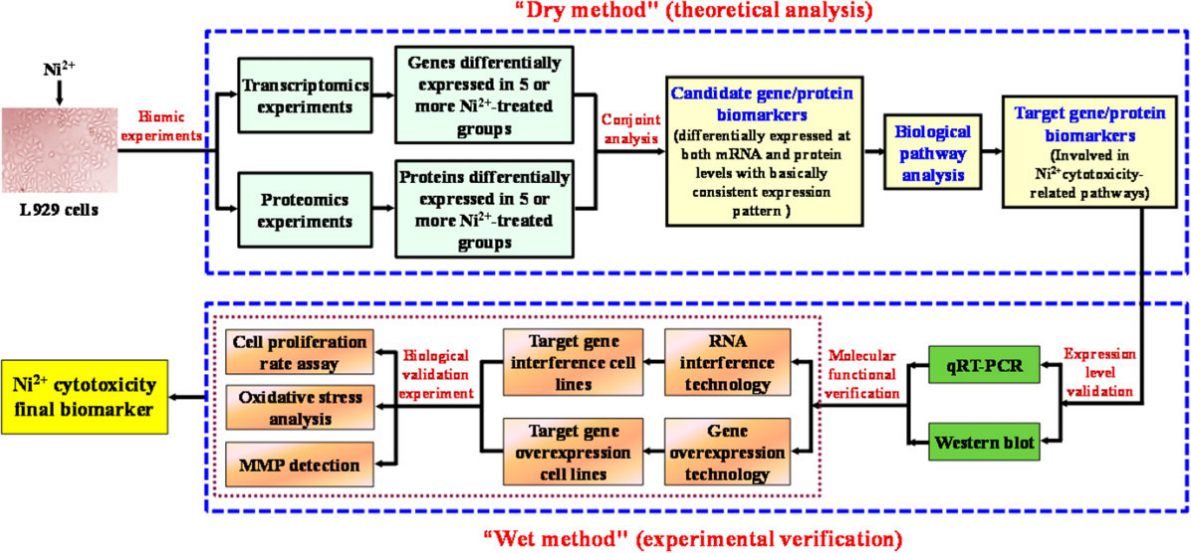
Technical route for research investigating Ni^2+^ cytotoxicity biomarkers.

This study establishes an innovative technical method for the screening of biomarkers of biomaterial cytotoxicity by combining the ‘dry method’ and ‘wet method’ (Fig. 11). This technique was used to conduct a joint multiomics screening of Ni^2+^ cytotoxicity biomarkers for the first time through combined analysis of transcriptomics and proteomics data (‘dry method’). Four target gene/protein biomarkers were first identified, and one final gene/protein biomarker (UQCRB) was identified through a series of cytomolecular experiments (‘wet method’). This study provides new insight into investigating cytotoxicity biomarkers of other biomaterials.

## Supplementary data

Supplementary data are available at *REGBIO* online.

## Funding

This paper was supported by the National Natural Science Foundation of China (31971254).

*Conflicts of interest statement*. None declared.

## Supplementary Material

rbac073_Supplementary_Data
