# Supplementary material for: Screening and validation of nickel ion cytotoxicity biomarkers based on transcriptomic and proteomic technology
Source: Regen Biomater. 2022 Sep 26;9:rbac073. doi: 10.1093/rb/rbac073 (PMC9550229; doi:10.1093/rb/rbac073)
Supplement: rbac073_Supplementary_Data [file rbac073_supplementary_data.docx]

**Supplementary Table S1:** Differentially expressed genes in L929 cells treated with 100μM Ni^2+^ for different durations.

| **100μM-12h** | | **100μM-24h** | | **100μM-48h** | | **100μM-72h** | |
| --- | --- | --- | --- | --- | --- | --- | --- |
| Entrez Gene ID | log_2_FC | Entrez Gene ID | log_2_FC | Entrez Gene ID | log_2_FC | Entrez Gene ID | log_2_FC |
| novel_G000500 | 4.97 | novel_G000829 | 4.98 | novel_G000829 | 4.93 | novel_G000829 | 4.66 |
| 237940 | 4.34 | novel_G000020 | 4.84 | 237940 | 4.05 | 237940 | 3.86 |
| 319152 | 3.03 | novel_G000640 | 3.41 | 78558 | 3.26 | 78558 | 3.60 |
| novel_G000640 | 2.78 | 12865 | 3.18 | 94180 | 2.91 | novel_G000640 | 3.57 |
| 16835 | 2.73 | novel_G000795 | 2.86 | 381284 | 2.84 | 18787 | 3.33 |
| novel_G000795 | 2.68 | 78558 | 2.73 | 22403 | 2.73 | 22403 | 3.19 |
| 319166 | 2.65 | 1E+08 | 2.54 | 77059 | 2.63 | 94180 | 3.16 |
| 665433 | 2.65 | 215627 | 2.48 | 76873 | 2.59 | 213393 | 3.09 |
| 81840 | 2.64 | 56295 | 2.42 | 70186 | 2.58 | 20753 | 3.07 |
| 78558 | 2.60 | 94180 | 2.39 | 12865 | 2.58 | 11450 | 2.96 |
| novel_G000839 | 2.45 | 76873 | 2.37 | novel_G000197 | 2.52 | novel_G000795 | 2.92 |
| 381284 | 2.42 | 74185 | 2.35 | 112407 | 2.43 | 76873 | 2.87 |
| NR_145503 | 2.29 | novel_G000500 | 2.34 | 14166 | 2.41 | 12865 | 2.85 |
| 112407 | 2.29 | 80879 | 2.33 | 80885 | 2.38 | 112407 | 2.76 |
| 319176 | 2.26 | novel_G000308 | 2.31 | 74185 | 2.34 | 107753 | 2.71 |
| novel_G000216 | 2.18 | NM_001349066 | 2.31 | 23945 | 2.30 | 18812 | 2.71 |
| 319182 | 2.14 | novel_G000092 | 2.27 | novel_G000247 | 2.29 | 70186 | 2.70 |
| 94180 | 2.12 | 112407 | 2.25 | novel_G000216 | 2.26 | novel_G000046 | 2.68 |
| 20250 | 2.12 | 70713 | 2.24 | 269275 | 2.26 | 23945 | 2.68 |
| 18378 | 2.10 | 654824 | 2.24 | novel_G000795 | 2.25 | 77059 | 2.67 |
| 12865 | 2.04 | 64095 | 2.17 | 100041375 | 2.17 | 81840 | 2.67 |
| 100041230 | 2.01 | 228026 | 2.11 | 12176 | 2.14 | novel_G000247 | 2.65 |
| 74747 | 2.00 | 12176 | 2.10 | 81840 | 2.13 | 109697 | 2.63 |
| 215627 | 2.00 | novel_G000097 | 2.10 | 70713 | 2.11 | 70045 | 2.61 |
| novel_G000732 | 2.00 | novel_G000247 | 2.09 | 433456 | 2.10 | 15267 | 2.61 |
| novel_G000645 | 2.00 | 626275 | 2.07 | 230099 | 2.10 | 433456 | 2.60 |
| 208715 | 2.00 | 74747 | 2.06 | 15267 | 2.09 | 74185 | 2.60 |
| 100039192 | 1.98 | 74121 | 2.05 | 69788 | 2.07 | 15439 | 2.59 |
| novel_G000308 | 1.97 | 23863 | 2.04 | 56295 | 2.06 | 381284 | 2.57 |
| 654824 | 1.97 | novel_G000709 | 2.02 | 331491 | 2.05 | 70713 | 2.54 |
| 319154 | 1.96 | 319152 | 2.00 | 667728 | 2.05 | 319189 | 2.53 |
| novel_G000378 | 1.91 | novel_G000093 | 1.98 | 319189 | 2.03 | 80885 | 2.50 |
| 78754 | 1.91 | novel_G000839 | 1.97 | 20753 | 2.03 | 74747 | 2.47 |
| 20135 | 1.88 | novel_G000545 | 1.97 | 19011 | 2.00 | novel_G000383 | 2.44 |
| 66234 | 1.87 | 17750 | 1.93 | 57435 | 2.00 | 66425 | 2.43 |
| 15331 | 1.87 | 70162 | 1.91 | 17988 | 1.99 | 94352 | 2.42 |
| 80879 | 1.86 | 269275 | 1.91 | 11600 | 1.98 | 230099 | 2.41 |
| novel_G000093 | 1.85 | 319166 | 1.91 | 20342 | 1.98 | 654824 | 2.40 |
| 19652 | 1.84 | novel_G000907 | 1.91 | 94352 | 1.97 | 14200 | 2.40 |
| 110460 | 1.84 | novel_G000197 | 1.90 | novel_G000092 | 1.96 | 16000 | 2.39 |
| 20198 | 1.83 | 74629 | 1.88 | 64095 | 1.95 | 64095 | 2.39 |
| 18194 | 1.82 | 18655 | 1.87 | 195359 | 1.95 | 17988 | 2.38 |
| 110196 | 1.81 | novel_G000362 | 1.86 | 654824 | 1.95 | 14311 | 2.37 |
| 108176 | 1.81 | novel_G000342 | 1.85 | 228026 | 1.94 | 407790 | 2.35 |
| 74246 | 1.81 | 545652 | 1.85 | 27367 | 1.93 | 12176 | 2.34 |
| 667766 | 1.79 | 407790 | 1.85 | 74747 | 1.92 | 20342 | 2.34 |
| 16906 | 1.78 | 11535 | 1.85 | 80879 | 1.89 | 11600 | 2.33 |
| 76220 | 1.76 | 17988 | 1.84 | 22283 | 1.87 | 11770 | 2.31 |
| 19348 | 1.76 | 20250 | 1.84 | 224813 | 1.87 | 57435 | 2.30 |
| 17748 | 1.76 | 72240 | 1.84 | 319190 | 1.85 | 16890 | 2.28 |
| 16592 | 1.75 | 20755 | 1.83 | 319181 | 1.83 | 74121 | 2.27 |
| 64095 | 1.75 | 1E+08 | 1.83 | 14857 | 1.82 | 14166 | 2.24 |
| 67642 | 1.74 | novel_G000732 | 1.81 | novel_G000383 | 1.82 | 56295 | 2.23 |
| 107995 | 1.74 | 69788 | 1.80 | 107753 | 1.81 | 11535 | 2.22 |
| 626904 | 1.73 | 70186 | 1.80 | 70045 | 1.81 | 14433 | 2.17 |
| 12428 | 1.72 | 109697 | 1.79 | novel_G000582 | 1.81 | 269275 | 2.16 |
| 72326 | 1.70 | 435766 | 1.79 | novel_G000592 | 1.80 | 13175 | 2.03 |
| 77056 | 1.70 | 72326 | 1.79 | 269423 | 1.79 | 12778 | 2.03 |
| 77940 | 1.69 | 320377 | 1.76 | 23980 | 1.78 | 53322 | 2.02 |
| 207728 | 1.69 | 433456 | 1.76 | 72157 | 1.78 | 68465 | 2.02 |
| 66447 | 1.68 | 16854 | 1.74 | novel_G000342 | 1.78 | 18426 | 2.00 |
| 70835 | 1.67 | novel_G000831 | 1.74 | 18655 | 1.78 | 171531 | 2.00 |
| 14793 | 1.66 | 1.01E+08 | 1.74 | 381232 | 1.77 | 14115 | 2.00 |
| 17750 | 1.66 | 360198 | 1.73 | 407790 | 1.76 | 667728 | 1.97 |
| 74341 | 1.65 | 381284 | 1.72 | 57390 | 1.76 | 72157 | 1.96 |
| 319181 | 1.65 | 77056 | 1.72 | 66838 | 1.75 | novel_G000214 | 1.96 |
| 228026 | 1.64 | 1E+08 | 1.70 | 320739 | 1.75 | 20341 | 1.95 |
| 232889 | 1.63 | 230099 | 1.69 | 12452 | 1.74 | novel_G000216 | 1.94 |
| 20419 | 1.63 | 72157 | 1.68 | 242642 | 1.73 | 20250 | 1.94 |
| 99543 | 1.62 | 72391 | 1.68 | 112405 | 1.72 | 228026 | 1.94 |
| novel_G000047 | 1.62 | 81840 | 1.67 | 13175 | 1.72 | 112405 | 1.93 |
| 56295 | 1.61 | 12452 | 1.67 | novel_G000732 | 1.71 | 50527 | 1.93 |
| 52530 | 1.60 | 73720 | 1.67 | 171531 | 1.70 | 80879 | 1.92 |
| 11600 | 1.59 | 11639 | 1.66 | 76905 | 1.70 | 245050 | 1.91 |
| 18641 | 1.59 | 170776 | 1.65 | 387514 | 1.69 | 208982 | 1.91 |
| 22042 | 1.59 | 1.01E+08 | 1.65 | 20341 | 1.68 | 66447 | 1.91 |
| 17218 | 1.58 | 77940 | 1.64 | novel_G000897 | 1.68 | 76905 | 1.90 |
| 68603 | 1.58 | 20525 | 1.64 | novel_G000097 | 1.67 | 15937 | 1.88 |
| 50708 | 1.58 | 20342 | 1.62 | novel_G000642 | 1.66 | 14282 | 1.88 |
| 16854 | 1.58 | 17748 | 1.61 | novel_G000545 | 1.65 | 100637 | 1.88 |
| 18140 | 1.58 | 14857 | 1.61 | 69454 | 1.64 | 69574 | 1.86 |
| 12306 | 1.57 | 545261 | 1.61 | 76378 | 1.62 | 235416 | 1.85 |
| 17215 | 1.57 | 18641 | 1.60 | 14200 | 1.61 | 12452 | 1.82 |
| 110033 | 1.56 | 208084 | 1.60 | 20250 | 1.61 | 19016 | 1.81 |
| 97165 | 1.55 | 14958 | 1.60 | 18787 | 1.60 | 68024 | 1.81 |
| 73720 | 1.55 | 94352 | 1.59 | 18405 | 1.59 | 67703 | 1.80 |
| 231070 | 1.53 | 328949 | 1.58 | 230895 | 1.59 | 319181 | 1.79 |
| 170776 | 1.53 | 330921 | 1.58 | 18563 | 1.59 | 23928 | 1.79 |
| 433456 | 1.53 | 107995 | 1.58 | 14433 | 1.57 | 545652 | 1.78 |
| 545261 | 1.51 | 269630 | 1.57 | novel_G000496 | 1.57 | 20657 | 1.78 |
| 16828 | 1.51 | 11676 | 1.57 | 18641 | 1.56 | 384071 | 1.78 |
| 16647 | 1.51 | novel_G000378 | 1.56 | 68465 | 1.56 | 50994 | 1.75 |
| 272551 | 1.51 | 16835 | 1.56 | 16854 | 1.56 | 57444 | 1.75 |
| 21335 | 1.51 | 66447 | 1.55 | 17001 | 1.56 | 18655 | 1.74 |
| 268697 | 1.50 | 381822 | 1.55 | 11676 | 1.55 | 18641 | 1.74 |
| 216148 | 1.50 | 1.01E+08 | 1.54 | 69574 | 1.54 | 20525 | 1.74 |
| 13121 | 1.49 | 15277 | 1.54 | 15399 | 1.53 | 20148 | 1.73 |
| 110956 | 1.49 | 16828 | 1.54 | 215627 | 1.53 | 23880 | 1.73 |
| 12843 | 1.48 | 235416 | 1.53 | 23880 | 1.53 | 70417 | 1.73 |
| 71406 | 1.48 | novel_G000696 | 1.53 | 105180375 | 1.52 | 17001 | 1.73 |
| NM_001348199 | 1.48 | NM_001348199 | 1.53 | 11535 | 1.52 | 23863 | 1.72 |
| 74185 | 1.48 | 381045 | 1.53 | 320046 | 1.52 | 1.01E+08 | 1.72 |
| 12722 | 1.48 | 244859 | 1.52 | 665268 | 1.51 | 331491 | 1.72 |
| 16987 | 1.47 | NM_001348198 | 1.52 | novel_G000696 | 1.50 | 13602 | 1.70 |
| 12316 | 1.47 | 70281 | 1.51 | 229699 | 1.50 | 230895 | 1.69 |
| novel_G000462 | 1.47 | 667766 | 1.51 | novel_G000246 | 1.49 | 20893 | 1.68 |
| 18787 | 1.47 | 112405 | 1.50 | 545652 | 1.49 | 114663 | 1.68 |
| 18655 | 1.46 | 16592 | 1.50 | 58210 | 1.48 | 13853 | 1.68 |
| 52276 | 1.46 | 319190 | 1.50 | 66447 | 1.48 | 18563 | 1.67 |
| 78653 | 1.46 | 20893 | 1.49 | 27981 | 1.48 | 394435 | 1.67 |
| 20893 | 1.46 | 12316 | 1.48 | 73673 | 1.47 | 320046 | 1.66 |
| novel_G000886 | 1.45 | novel_G000010 | 1.48 | 71769 | 1.47 | 545261 | 1.66 |
| 72391 | 1.45 | 433182 | 1.47 | 20525 | 1.46 | 18405 | 1.65 |
| 231801 | 1.45 | 50527 | 1.46 | 381853 | 1.46 | 56615 | 1.65 |
| 19891 | 1.45 | 60406 | 1.46 | 18407 | 1.45 | novel_G000092 | 1.64 |
| 12615 | 1.44 | novel_G000143 | 1.45 | 17436 | 1.45 | 319190 | 1.64 |
| 14235 | 1.44 | 227526 | 1.44 | 433182 | 1.45 | 20710 | 1.64 |
| 100039781 | 1.44 | 21991 | 1.44 | 545936 | 1.45 | 244859 | 1.64 |
| 545652 | 1.44 | 13175 | 1.43 | 77940 | 1.45 | 239691 | 1.64 |
| 414101 | 1.44 | 320046 | 1.43 | 399558 | 1.44 | 269423 | 1.64 |
| 78767 | 1.44 | 110196 | 1.42 | 271278 | 1.44 | 24052 | 1.63 |
| 56075 | 1.44 | 12217 | 1.42 | 19016 | 1.43 | novel_G000696 | 1.63 |
| 17345 | 1.43 | 69573 | 1.42 | 56615 | 1.43 | 22283 | 1.62 |
| 654467 | 1.43 | 19348 | 1.41 | 16828 | 1.43 | novel_G000197 | 1.62 |
| 11958 | 1.43 | 14714 | 1.41 | 14714 | 1.42 | 16854 | 1.62 |
| 14857 | 1.43 | 16987 | 1.41 | 328949 | 1.42 | 435766 | 1.62 |
| 208084 | 1.43 | 14433 | 1.40 | 11770 | 1.41 | 381853 | 1.61 |
| 66101 | 1.43 | 18563 | 1.40 | 50527 | 1.41 | 20787 | 1.61 |
| 109212 | 1.42 | 20709 | 1.39 | 235416 | 1.41 | 407786 | 1.61 |
| 17855 | 1.41 | 17436 | 1.39 | 407786 | 1.40 | 399558 | 1.60 |
| 107753 | 1.41 | 20198 | 1.38 | 245050 | 1.40 | 57875 | 1.60 |
| 504193 | 1.41 | 14936 | 1.38 | 71406 | 1.40 | 11676 | 1.60 |
| 67629 | 1.41 | 74341 | 1.38 | 12722 | 1.40 | 78512 | 1.60 |
| 69325 | 1.40 | 20725 | 1.38 | 384071 | 1.39 | 113848 | 1.59 |
| 66461 | 1.40 | 665433 | 1.38 | 18746 | 1.39 | novel_G000897 | 1.59 |
| novel_G000806 | 1.40 | 20148 | 1.38 | 57444 | 1.39 | 19252 | 1.59 |
| 74016 | 1.40 | novel_G000199 | 1.37 | 233789 | 1.38 | 76854 | 1.58 |
| 30878 | 1.40 | 269423 | 1.37 | 14115 | 1.37 | 20971 | 1.58 |
| 234577 | 1.40 | novel_G000385 | 1.37 | novel_G000385 | 1.37 | 1E+08 | 1.57 |
| 20525 | 1.40 | 18173 | 1.36 | 18648 | 1.37 | 80976 | 1.57 |
| 68278 | 1.39 | 14793 | 1.36 | 50782 | 1.37 | 84004 | 1.56 |
| 67052 | 1.39 | 77059 | 1.36 | 100503178 | 1.36 | 11832 | 1.56 |
| 72657 | 1.39 | 545936 | 1.36 | 11906 | 1.36 | 19268 | 1.56 |
| 12442 | 1.39 | 114230 | 1.36 | 20710 | 1.36 | 11881 | 1.56 |
| 381822 | 1.38 | 21929 | 1.35 | 80976 | 1.36 | 18648 | 1.56 |
| 14115 | 1.38 | 24052 | 1.35 | novel_G000199 | 1.35 | 11674 | 1.55 |
| 67486 | 1.38 | 77533 | 1.35 | 216859 | 1.35 | 26432 | 1.54 |
| 67681 | 1.38 | 18746 | 1.35 | 104263 | 1.34 | 14281 | 1.53 |
| 66197 | 1.38 | 18787 | 1.35 | 14936 | 1.34 | 21942 | 1.52 |
| 78512 | 1.38 | 15586 | 1.35 | 230098 | 1.33 | 74341 | 1.52 |
| 19655 | 1.37 | 72655 | 1.35 | 14311 | 1.32 | novel_G000342 | 1.52 |
| 240892 | 1.37 | 57435 | 1.34 | 71361 | 1.31 | 433182 | 1.52 |
| 21991 | 1.37 | 70045 | 1.33 | 213988 | 1.31 | 1.01E+08 | 1.52 |
| 319554 | 1.37 | 384071 | 1.33 | 100039060 | 1.31 | 14936 | 1.52 |
| 435766 | 1.36 | 1E+08 | 1.32 | 626275 | 1.30 | 21807 | 1.51 |
| 68298 | 1.36 | 71653 | 1.31 | 11639 | 1.30 | 21929 | 1.51 |
| 68385 | 1.36 | 1.01E+08 | 1.30 | 227526 | 1.30 | 11639 | 1.50 |
| 66192 | 1.36 | 18648 | 1.30 | 213393 | 1.30 | 18746 | 1.49 |
| 11535 | 1.35 | 12722 | 1.30 | 109672 | 1.29 | 16956 | 1.49 |
| 68026 | 1.35 | 18302 | 1.30 | 545261 | 1.29 | 16828 | 1.49 |
| 71241 | 1.35 | 1E+08 | 1.30 | novel_G000210 | 1.29 | 12835 | 1.49 |
| 16907 | 1.35 | 1E+08 | 1.29 | 394435 | 1.29 | 207728 | 1.49 |
| 72157 | 1.34 | 68603 | 1.29 | 380753 | 1.28 | 17436 | 1.48 |
| novel_G000345 | 1.34 | 12532 | 1.29 | 113848 | 1.28 | 545936 | 1.48 |
| 433182 | 1.34 | 12306 | 1.28 | 16890 | 1.28 | 237847 | 1.48 |
| 68816 | 1.34 | 414072 | 1.28 | 208777 | 1.28 | 328949 | 1.48 |
| 68612 | 1.34 | 93694 | 1.28 | 214150 | 1.28 | 18452 | 1.47 |
| 18817 | 1.34 | 414101 | 1.28 | 207728 | 1.27 | 11520 | 1.46 |
| 20133 | 1.34 | 19652 | 1.28 | 14751 | 1.27 | 211666 | 1.45 |
| 18392 | 1.34 | 270685 | 1.28 | 20893 | 1.27 | 74782 | 1.44 |
| 100034739 | 1.34 | 239691 | 1.27 | 211666 | 1.27 | 104080 | 1.44 |
| 67305 | 1.34 | novel_G000144 | 1.27 | 74341 | 1.26 | 20709 | 1.44 |
| 15357 | 1.33 | 13885 | 1.27 | 114663 | 1.25 | 13601 | 1.44 |
| 50927 | 1.33 | novel_G000375 | 1.26 | 208151 | 1.25 | 20198 | 1.44 |
| 13167 | 1.32 | 229841 | 1.26 | 20887 | 1.24 | novel_G000246 | 1.43 |
| 18538 | 1.32 | 13806 | 1.25 | 320965 | 1.24 | 71361 | 1.43 |
| 57435 | 1.32 | 18412 | 1.25 | 237847 | 1.24 | 77940 | 1.42 |
| 15163 | 1.32 | 213393 | 1.25 | novel_G000961 | 1.23 | 99543 | 1.42 |
| NM_001348198 | 1.31 | 108000 | 1.25 | 104080 | 1.23 | 434147 | 1.42 |
| 15366 | 1.31 | 1E+08 | 1.25 | 20186 | 1.23 | 71406 | 1.42 |
| 328949 | 1.31 | 69206 | 1.25 | 69772 | 1.23 | 67088 | 1.41 |
| 68671 | 1.31 | 12615 | 1.25 | 14723 | 1.23 | 12491 | 1.40 |
| 76873 | 1.30 | 20710 | 1.24 | 13885 | 1.23 | 30878 | 1.40 |
| 67037 | 1.30 | 381308 | 1.24 | 22352 | 1.23 | 12833 | 1.39 |
| 14936 | 1.30 | 68465 | 1.24 | novel_G000093 | 1.22 | 14601 | 1.39 |
| 626275 | 1.30 | 67530 | 1.24 | 52662 | 1.22 | 108150 | 1.39 |
| 110809 | 1.30 | 71406 | 1.24 | 73720 | 1.22 | 15586 | 1.39 |
| 110208 | 1.30 | novel_G000091 | 1.23 | 67101 | 1.21 | 665306 | 1.38 |
| 19016 | 1.30 | 104263 | 1.23 | 76441 | 1.21 | novel_G000148 | 1.38 |
| 209737 | 1.30 | 12428 | 1.23 | 108150 | 1.21 | 380912 | 1.38 |
| 13605 | 1.29 | 26432 | 1.22 | 24052 | 1.21 | 15258 | 1.38 |
| 242691 | 1.29 | 20971 | 1.22 | 100861531 | 1.21 | 215627 | 1.38 |
| 108000 | 1.29 | 270906 | 1.22 | 19329 | 1.21 | 68404 | 1.37 |
| 11639 | 1.29 | 109672 | 1.22 | 338360 | 1.20 | 16592 | 1.37 |
| 19361 | 1.28 | 68612 | 1.21 | 100038453 | 1.20 | 235435 | 1.37 |
| 26914 | 1.28 | 230098 | 1.21 | 213649 | 1.20 | novel_G000859 | 1.37 |
| 16571 | 1.28 | 78575 | 1.21 | 109689 | 1.20 | 27219 | 1.37 |
| 22228 | 1.27 | 20341 | 1.21 | 11674 | 1.20 | 380753 | 1.37 |
| 67678 | 1.27 | 67669 | 1.21 | 14205 | 1.19 | 14751 | 1.36 |
| 14431 | 1.27 | 70454 | 1.21 | 13807 | 1.19 | 14723 | 1.36 |
| 67693 | 1.27 | 83436 | 1.20 | 282619 | 1.19 | 11826 | 1.36 |
| 109672 | 1.27 | novel_G000797 | 1.20 | 100039781 | 1.19 | 193796 | 1.36 |
| 20710 | 1.27 | 399558 | 1.20 | 18173 | 1.19 | 69772 | 1.36 |
| 69731 | 1.27 | 73673 | 1.20 | 21807 | 1.19 | 104263 | 1.36 |
| 50783 | 1.26 | 74782 | 1.19 | 68235 | 1.19 | 11906 | 1.35 |
| 14283 | 1.26 | 107869 | 1.19 | 18452 | 1.19 | 50791 | 1.35 |
| 68226 | 1.26 | 11674 | 1.18 | 20709 | 1.19 | 319162 | 1.35 |
| 268977 | 1.26 | 17319 | 1.18 | 269198 | 1.18 | 109689 | 1.35 |
| 244550 | 1.26 | 72607 | 1.18 | 84004 | 1.18 | 269630 | 1.35 |
| 69639 | 1.26 | 193796 | 1.17 | 243538 | 1.18 | 1.01E+08 | 1.34 |
| 72341 | 1.25 | 78767 | 1.17 | 12778 | 1.18 | 21991 | 1.34 |
| 17865 | 1.25 | 268697 | 1.17 | 100504267 | 1.18 | 108767 | 1.34 |
| 73804 | 1.25 | 52276 | 1.17 | 320181 | 1.17 | 69573 | 1.33 |
| 19385 | 1.25 | 380753 | 1.17 | 83436 | 1.17 | 214137 | 1.33 |
| 107435 | 1.25 | 74107 | 1.16 | 17311 | 1.17 | 12257 | 1.33 |
| 18648 | 1.24 | 320700 | 1.16 | 18193 | 1.17 | 665268 | 1.33 |
| novel_G000646 | 1.24 | 22042 | 1.16 | 21924 | 1.16 | 329252 | 1.33 |
| 12532 | 1.23 | 13605 | 1.16 | 69399 | 1.16 | 20411 | 1.33 |
| 66140 | 1.23 | 110033 | 1.15 | 15400 | 1.16 | 1.01E+08 | 1.32 |
| 58859 | 1.23 | 104080 | 1.15 | 626832 | 1.16 | 240327 | 1.32 |
| 22352 | 1.23 | 14166 | 1.15 | 20148 | 1.16 | 320678 | 1.32 |
| 112405 | 1.23 | 108912 | 1.14 | 621976 | 1.16 | 21415 | 1.32 |
| 19944 | 1.23 | 240892 | 1.14 | 15586 | 1.16 | 320700 | 1.32 |
| 70247 | 1.23 | 237847 | 1.14 | 380912 | 1.15 | 230098 | 1.32 |
| 100910 | 1.23 | 28010 | 1.14 | 54524 | 1.15 | 12722 | 1.32 |
| 319190 | 1.22 | novel_G000491 | 1.14 | novel_G000976 | 1.15 | 52662 | 1.31 |
| 70454 | 1.22 | 27981 | 1.13 | 21929 | 1.15 | 16527 | 1.31 |
| 72107 | 1.22 | 677884 | 1.13 | 26388 | 1.15 | 320181 | 1.31 |
| 74107 | 1.22 | 68226 | 1.13 | 76854 | 1.15 | 20682 | 1.30 |
| 69736 | 1.22 | 108150 | 1.13 | 16527 | 1.15 | 56338 | 1.30 |
| 240514 | 1.22 | 216859 | 1.13 | 546726 | 1.14 | novel_G000491 | 1.30 |
| 76478 | 1.21 | 14235 | 1.13 | 320678 | 1.14 | 104252 | 1.30 |
| 14205 | 1.21 | 73204 | 1.12 | 71302 | 1.14 | 11303 | 1.30 |
| 12534 | 1.21 | 78754 | 1.12 | 20787 | 1.14 | 97122 | 1.29 |
| 19076 | 1.21 | 18822 | 1.12 | 72240 | 1.14 | 15399 | 1.29 |
| 20586 | 1.21 | 67052 | 1.12 | 20657 | 1.13 | 18451 | 1.29 |
| 12176 | 1.21 | 14325 | 1.12 | 20971 | 1.13 | 216859 | 1.29 |
| 83701 | 1.21 | 20878 | 1.12 | 320842 | 1.13 | 11717 | 1.28 |
| 11826 | 1.21 | 207728 | 1.12 | 269630 | 1.13 | 83436 | 1.27 |
| 270906 | 1.21 | 329650 | 1.11 | 11430 | 1.13 | 18126 | 1.27 |
| 27387 | 1.21 | 1.01E+08 | 1.11 | 22436 | 1.13 | 328967 | 1.27 |
| 20878 | 1.20 | 12442 | 1.11 | 19041 | 1.12 | 246228 | 1.27 |
| 19285 | 1.20 | 654467 | 1.11 | 12217 | 1.12 | 1.01E+08 | 1.27 |
| novel_G000326 | 1.20 | 238130 | 1.11 | novel_G000815 | 1.12 | 13806 | 1.27 |
| 13806 | 1.20 | 17345 | 1.10 | 223650 | 1.12 | 1E+08 | 1.26 |
| 21877 | 1.19 | 53322 | 1.10 | 74183 | 1.12 | 107581 | 1.26 |
| 380752 | 1.19 | 50708 | 1.10 | 11520 | 1.12 | 80288 | 1.26 |
| 18127 | 1.19 | 66234 | 1.09 | novel_G000949 | 1.11 | 22695 | 1.26 |
| 68219 | 1.19 | 626877 | 1.09 | 16000 | 1.11 | 22339 | 1.25 |
| 17217 | 1.18 | 11906 | 1.09 | 20411 | 1.11 | 13807 | 1.25 |
| 270685 | 1.18 | 54725 | 1.09 | 319162 | 1.11 | 78754 | 1.25 |
| 68743 | 1.18 | 219144 | 1.09 | 214133 | 1.11 | 69399 | 1.25 |
| 69535 | 1.18 | 64009 | 1.09 | 193796 | 1.11 | 1E+08 | 1.24 |
| novel_G000092 | 1.18 | novel_G000584 | 1.09 | 12663 | 1.10 | 338360 | 1.24 |
| 20709 | 1.18 | 13807 | 1.09 | novel_G000854 | 1.10 | 114606 | 1.24 |
| 11799 | 1.18 | 211666 | 1.08 | 94089 | 1.10 | 12215 | 1.24 |
| 18746 | 1.18 | 1E+08 | 1.08 | 21942 | 1.10 | 207259 | 1.23 |
| 16551 | 1.17 | 67170 | 1.08 | 97775 | 1.10 | 26388 | 1.23 |
| 224171 | 1.17 | 20419 | 1.08 | 270685 | 1.10 | 78575 | 1.23 |
| 19041 | 1.17 | 67486 | 1.08 | 435766 | 1.10 | 17311 | 1.22 |
| 18173 | 1.17 | 14283 | 1.08 | 74396 | 1.10 | 237928 | 1.22 |
| 12704 | 1.17 | 18817 | 1.08 | 18451 | 1.10 | 69745 | 1.22 |
| 108912 | 1.17 | 319722 | 1.08 | 19885 | 1.09 | novel_G000097 | 1.22 |
| 229841 | 1.16 | 171531 | 1.08 | 66359 | 1.09 | 208659 | 1.21 |
| 108907 | 1.16 | 12257 | 1.08 | 26432 | 1.09 | 18822 | 1.21 |
| 381045 | 1.16 | 66197 | 1.07 | 67689 | 1.09 | 60363 | 1.21 |
| 13730 | 1.15 | novel_G000525 | 1.07 | 104252 | 1.09 | 16782 | 1.21 |
| 56207 | 1.15 | 234577 | 1.07 | novel_G000525 | 1.08 | 18173 | 1.20 |
| 13358 | 1.15 | 13730 | 1.07 | 13602 | 1.08 | 11658 | 1.20 |
| 67293 | 1.15 | 108811 | 1.07 | 11832 | 1.08 | 73673 | 1.19 |
| 69706 | 1.15 | 76220 | 1.07 | 20198 | 1.08 | 217082 | 1.19 |
| 445007 | 1.15 | 18452 | 1.07 | 216616 | 1.08 | 19041 | 1.19 |
| 216616 | 1.15 | 15366 | 1.06 | 21991 | 1.08 | 11430 | 1.19 |
| 66977 | 1.15 | 97165 | 1.06 | 68404 | 1.08 | 217837 | 1.19 |
| 72119 | 1.15 | 99543 | 1.06 | 78512 | 1.07 | 71769 | 1.19 |
| novel_G000445 | 1.15 | 54524 | 1.06 | 97122 | 1.07 | 1E+08 | 1.19 |
| 260315 | 1.15 | 50791 | 1.06 | 14598 | 1.07 | 100910 | 1.19 |
| novel_G000468 | 1.14 | 110208 | 1.06 | 11443 | 1.07 | 381399 | 1.18 |
| 100502766 | 1.14 | 229699 | 1.06 | 18190 | 1.07 | 20623 | 1.18 |
| 71819 | 1.14 | 109212 | 1.06 | 11303 | 1.07 | 66610 | 1.18 |
| 224530 | 1.14 | 108907 | 1.05 | 13853 | 1.07 | 93692 | 1.18 |
| 70025 | 1.14 | 68982 | 1.05 | 12257 | 1.06 | 72240 | 1.18 |
| 12235 | 1.14 | 233406 | 1.05 | novel_G000491 | 1.06 | novel_G000093 | 1.18 |
| 74782 | 1.13 | 319707 | 1.05 | 13601 | 1.05 | 15275 | 1.18 |
| 67951 | 1.13 | 17930 | 1.05 | 227195 | 1.05 | 16859 | 1.18 |
| 66373 | 1.13 | 74246 | 1.05 | 269695 | 1.05 | 270685 | 1.17 |
| 59001 | 1.13 | 66388 | 1.05 | 240327 | 1.05 | novel_G000525 | 1.17 |
| 14433 | 1.13 | novel_G000246 | 1.05 | 383348 | 1.05 | 213945 | 1.17 |
| 18102 | 1.13 | 269610 | 1.05 | 14325 | 1.05 | 213649 | 1.17 |
| 17220 | 1.13 | 16322 | 1.05 | 16592 | 1.04 | 232232 | 1.16 |
| 56505 | 1.12 | 74034 | 1.05 | 14860 | 1.04 | 235431 | 1.16 |
| 22390 | 1.12 | 399101 | 1.05 | 381022 | 1.04 | 332934 | 1.16 |
| 17192 | 1.12 | 213054 | 1.04 | 53322 | 1.04 | 108811 | 1.16 |
| 14137 | 1.12 | 72341 | 1.04 | 236733 | 1.04 | 231863 | 1.15 |
| 66570 | 1.12 | 13167 | 1.04 | novel_G000531 | 1.03 | 12177 | 1.15 |
| 19362 | 1.12 | 57444 | 1.04 | 381399 | 1.03 | 77864 | 1.15 |
| 23834 | 1.12 | 69399 | 1.04 | 244310 | 1.03 | 244310 | 1.15 |
| 233406 | 1.11 | 16580 | 1.04 | 100504191 | 1.03 | 107817 | 1.15 |
| 66442 | 1.11 | 107817 | 1.04 | 216505 | 1.03 | 381045 | 1.14 |
| 235416 | 1.11 | 19655 | 1.03 | 619547 | 1.03 | 12825 | 1.14 |
| 14211 | 1.11 | 77619 | 1.03 | 107747 | 1.03 | 19329 | 1.14 |
| 19895 | 1.11 | 72080 | 1.03 | 382913 | 1.02 | 16973 | 1.14 |
| 66929 | 1.11 | 207259 | 1.03 | 70417 | 1.02 | 67689 | 1.14 |
| 67530 | 1.11 | 233107 | 1.03 | 13806 | 1.02 | 12816 | 1.14 |
| 12449 | 1.10 | 216345 | 1.03 | 77864 | 1.02 | 14431 | 1.13 |
| 234723 | 1.10 | 17873 | 1.03 | 16859 | 1.02 | novel_G000375 | 1.13 |
| 12833 | 1.10 | 74183 | 1.02 | 216377 | 1.02 | 237858 | 1.13 |
| 26934 | 1.10 | 74205 | 1.02 | 12833 | 1.02 | 244238 | 1.13 |
| 280408 | 1.10 | 224170 | 1.02 | 58239 | 1.02 | 216616 | 1.13 |
| 76936 | 1.10 | 18451 | 1.02 | 68982 | 1.02 | 77619 | 1.13 |
| 68201 | 1.10 | 224171 | 1.02 | 73251 | 1.02 | 233789 | 1.12 |
| 104112 | 1.10 | 11605 | 1.02 | 214162 | 1.01 | 241452 | 1.12 |
| novel_G000564 | 1.10 | 217837 | 1.02 | 114606 | 1.01 | 19088 | 1.12 |
| 319707 | 1.10 | 11770 | 1.01 | 21415 | 1.01 | 74202 | 1.12 |
| 76915 | 1.10 | 1.01E+08 | 1.01 | 329251 | 1.01 | 67101 | 1.12 |
| 12452 | 1.09 | 70900 | 1.01 | 16956 | 1.01 | novel_G000210 | 1.12 |
| 269630 | 1.09 | 105440 | 1.01 | 11826 | 1.01 | 66079 | 1.12 |
| 107869 | 1.09 | 70417 | 1.01 | 57294 | 1.01 | 56421 | 1.11 |
| 114663 | 1.09 | 209737 | 1.01 | 22228 | 1.01 | 213988 | 1.11 |
| 404710 | 1.09 | 76222 | 1.01 | 209239 | 1.01 | 14205 | 1.11 |
| 67141 | 1.09 | 330737 | 1.01 | 245622 | 1.01 | novel_G000095 | 1.11 |
| 104080 | 1.09 | 72357 | 1.01 | 64009 | 1.00 | 78330 | 1.11 |
| 57905 | 1.09 | 14867 | -1.00 | 246228 | 1.00 | 12306 | 1.10 |
| 72269 | 1.09 | 207785 | -1.00 | 319565 | 1.00 | 12842 | 1.10 |
| 28200 | 1.09 | 242125 | -1.01 | 60345 | -1.00 | 16367 | 1.09 |
| 16905 | 1.09 | 55948 | -1.01 | 76088 | -1.00 | 22436 | 1.09 |
| 237847 | 1.09 | 28105 | -1.01 | 241633 | -1.00 | 18302 | 1.09 |
| 68465 | 1.09 | novel_G000120 | -1.01 | 15936 | -1.00 | 22352 | 1.09 |
| 15354 | 1.08 | 245527 | -1.01 | 67724 | -1.00 | 238130 | 1.09 |
| novel_G000696 | 1.08 | novel_G000190 | -1.01 | 20583 | -1.01 | 17691 | 1.09 |
| 233876 | 1.08 | 29809 | -1.01 | 381924 | -1.01 | 259302 | 1.08 |
| novel_G000525 | 1.08 | 27204 | -1.01 | 266459 | -1.01 | 11568 | 1.08 |
| 66493 | 1.08 | 329735 | -1.02 | 100039691 | -1.01 | 66812 | 1.08 |
| 78890 | 1.08 | 14991 | -1.02 | 19698 | -1.01 | 269855 | 1.08 |
| 100503178 | 1.08 | 1.01E+08 | -1.02 | 218630 | -1.01 | 240514 | 1.08 |
| 12236 | 1.08 | 214952 | -1.02 | 208266 | -1.01 | 353502 | 1.08 |
| novel_G000097 | 1.08 | 100604 | -1.02 | 76429 | -1.01 | 18003 | 1.08 |
| 110854 | 1.08 | 269589 | -1.02 | 12322 | -1.01 | 118452 | 1.08 |
| 66556 | 1.08 | 17069 | -1.03 | 66583 | -1.02 | 252972 | 1.08 |
| 24052 | 1.07 | 17304 | -1.03 | 210992 | -1.02 | 26401 | 1.07 |
| 77777 | 1.07 | 12862 | -1.03 | 71884 | -1.02 | 140792 | 1.07 |
| 15469 | 1.07 | 234593 | -1.04 | 380863 | -1.02 | 11443 | 1.06 |
| 94352 | 1.07 | novel_G000088 | -1.04 | 207181 | -1.02 | 15460 | 1.06 |
| 268373 | 1.07 | 1E+08 | -1.04 | 75801 | -1.02 | 29815 | 1.06 |
| 16580 | 1.07 | 54614 | -1.04 | 15200 | -1.02 | 319942 | 1.06 |
| 236082 | 1.07 | 77974 | -1.04 | 209558 | -1.02 | 269198 | 1.06 |
| 17216 | 1.07 | 12509 | -1.04 | 171210 | -1.03 | 235599 | 1.06 |
| 111241 | 1.07 | 58182 | -1.05 | 18393 | -1.03 | 319876 | 1.06 |
| 18563 | 1.07 | 50722 | -1.05 | 67876 | -1.03 | 229595 | 1.06 |
| 20787 | 1.06 | novel_G000192 | -1.05 | 105083 | -1.03 | 210126 | 1.06 |
| 66276 | 1.06 | 59031 | -1.05 | 13617 | -1.04 | 216505 | 1.06 |
| 78286 | 1.06 | 13110 | -1.05 | 110454 | -1.04 | 13829 | 1.05 |
| 21956 | 1.06 | 57814 | -1.05 | 56200 | -1.04 | 12217 | 1.05 |
| 72080 | 1.06 | 668212 | -1.05 | 16007 | -1.04 | 12953 | 1.05 |
| 74268 | 1.06 | 64294 | -1.05 | 67838 | -1.04 | 68952 | 1.05 |
| 74356 | 1.06 | 12577 | -1.05 | 76884 | -1.04 | 56078 | 1.05 |
| 216873 | 1.06 | 271842 | -1.06 | NM_001348231 | -1.04 | 54725 | 1.04 |
| 17219 | 1.05 | 1E+08 | -1.06 | 75778 | -1.05 | 243270 | 1.04 |
| 26442 | 1.05 | 68396 | -1.06 | 13654 | -1.05 | 22042 | 1.04 |
| 269582 | 1.05 | 544881 | -1.06 | 76718 | -1.05 | 15400 | 1.04 |
| 230753 | 1.05 | 18791 | -1.06 | 18442 | -1.05 | 223650 | 1.04 |
| 100113398 | 1.05 | 68728 | -1.06 | 105727 | -1.06 | 67446 | 1.04 |
| 72692 | 1.05 | 21899 | -1.06 | 226351 | -1.06 | 13047 | 1.03 |
| 791299 | 1.05 | 14102 | -1.06 | 73737 | -1.06 | 11364 | 1.03 |
| 17279 | 1.05 | 71998 | -1.07 | 14085 | -1.06 | 66834 | 1.03 |
| 74205 | 1.04 | 105727 | -1.07 | 23892 | -1.06 | 27981 | 1.03 |
| 53333 | 1.04 | 21664 | -1.07 | novel_G000058 | -1.07 | 12834 | 1.03 |
| 108961 | 1.04 | 209558 | -1.07 | 13537 | -1.08 | 73720 | 1.03 |
| 69860 | 1.04 | 17063 | -1.07 | 109095 | -1.08 | 17534 | 1.02 |
| 545056 | 1.04 | 12322 | -1.07 | 74178 | -1.09 | 50708 | 1.02 |
| 19387 | 1.04 | 14085 | -1.08 | 101809 | -1.09 | 245622 | 1.02 |
| 17089 | 1.04 | 16408 | -1.09 | 380930 | -1.09 | 22228 | 1.01 |
| 20641 | 1.04 | novel_G000987 | -1.09 | 70769 | -1.09 | 66487 | 1.01 |
| 381308 | 1.04 | novel_G000412 | -1.09 | 21664 | -1.09 | 12263 | 1.01 |
| novel_G000197 | 1.04 | 16470 | -1.09 | 19699 | -1.09 | 13885 | 1.01 |
| 223870 | 1.03 | 13982 | -1.10 | 74155 | -1.10 | 319939 | 1.01 |
| novel_G000815 | 1.03 | 214084 | -1.10 | 56219 | -1.10 | 24132 | 1.00 |
| 70900 | 1.03 | 11642 | -1.10 | 13479 | -1.10 | 626832 | 1.00 |
| novel_G000949 | 1.03 | 243382 | -1.11 | 21857 | -1.10 | 208076 | 1.00 |
| 104263 | 1.03 | 106861 | -1.11 | 12977 | -1.11 | 208777 | 1.00 |
| 219144 | 1.03 | 11603 | -1.11 | 76367 | -1.11 | 13170 | 1.00 |
| 54364 | 1.03 | 78749 | -1.12 | 245527 | -1.11 | 243574 | -1.00 |
| 19179 | 1.03 | 1.03E+08 | -1.12 | 20537 | -1.11 | 1E+08 | -1.00 |
| 11770 | 1.03 | 20732 | -1.12 | 18574 | -1.11 | 106952 | -1.01 |
| 67895 | 1.03 | 72393 | -1.12 | 240672 | -1.12 | 259044 | -1.01 |
| 13433 | 1.03 | 71519 | -1.13 | 13610 | -1.12 | 11304 | -1.01 |
| 23997 | 1.03 | 353235 | -1.13 | 18755 | -1.12 | 14085 | -1.01 |
| 78894 | 1.03 | 228357 | -1.13 | 11838 | -1.12 | 18218 | -1.02 |
| 14241 | 1.02 | 16531 | -1.13 | 11997 | -1.13 | 13617 | -1.02 |
| 16765 | 1.02 | 67138 | -1.13 | 246727 | -1.13 | 257633 | -1.02 |
| 15277 | 1.02 | 14367 | -1.13 | 53414 | -1.13 | 15569 | -1.02 |
| 380753 | 1.02 | 229445 | -1.13 | 19368 | -1.13 | 14969 | -1.02 |
| 72357 | 1.02 | 319236 | -1.13 | 17972 | -1.14 | 216439 | -1.03 |
| 12934 | 1.02 | 218311 | -1.13 | novel_G000834 | -1.14 | 15574 | -1.03 |
| 72140 | 1.02 | 1E+08 | -1.13 | 268567 | -1.14 | 1E+08 | -1.03 |
| 66047 | 1.02 | novel_G000188 | -1.13 | 12508 | -1.15 | 52850 | -1.03 |
| 595139 | 1.02 | 68347 | -1.14 | 93838 | -1.15 | novel_G000412 | -1.03 |
| 76238 | 1.01 | 224093 | -1.14 | 328162 | -1.15 | 268656 | -1.03 |
| 21929 | 1.01 | 17940 | -1.14 | 207182 | -1.15 | 18191 | -1.03 |
| novel_G000430 | 1.01 | 68176 | -1.14 | 117167 | -1.16 | 14828 | -1.03 |
| 68988 | 1.01 | 211378 | -1.15 | 12282 | -1.16 | 271842 | -1.04 |
| 242705 | 1.01 | 629016 | -1.15 | novel_G000189 | -1.16 | 54712 | -1.04 |
| 69573 | 1.01 | 14632 | -1.15 | 117606 | -1.16 | 70383 | -1.04 |
| 80288 | 1.01 | 219151 | -1.15 | 70383 | -1.17 | 12977 | -1.04 |
| 16881 | 1.01 | 12304 | -1.15 | 230766 | -1.17 | 11987 | -1.04 |
| 11676 | 1.01 | 73737 | -1.16 | 229949 | -1.17 | 69066 | -1.04 |
| 68044 | 1.01 | 24066 | -1.16 | 20112 | -1.17 | 74840 | -1.05 |
| 28030 | 1.01 | novel_G000189 | -1.16 | 59014 | -1.17 | 21452 | -1.06 |
| 27214 | 1.00 | 14979 | -1.16 | 67603 | -1.17 | 12223 | -1.06 |
| 71843 | 1.00 | 19219 | -1.16 | 16574 | -1.18 | 1E+08 | -1.06 |
| 13361 | 1.00 | 98682 | -1.16 | 19260 | -1.18 | 414069 | -1.06 |
| 52033 | 1.00 | 80907 | -1.16 | 22361 | -1.18 | 19183 | -1.07 |
| 18861 | 1.00 | 18295 | -1.17 | 67065 | -1.18 | 72121 | -1.07 |
| 235533 | -1.00 | 56336 | -1.17 | 20355 | -1.19 | 67246 | -1.07 |
| 212974 | -1.00 | 239122 | -1.17 | 227731 | -1.19 | 103511 | -1.07 |
| 320172 | -1.00 | 276919 | -1.17 | 54342 | -1.19 | 74229 | -1.07 |
| 66065 | -1.00 | 252973 | -1.17 | 15414 | -1.19 | 60345 | -1.07 |
| 433752 | -1.00 | 1E+08 | -1.18 | 319583 | -1.19 | 13531 | -1.07 |
| 68943 | -1.00 | 396184 | -1.18 | 545539 | -1.20 | 246727 | -1.07 |
| 13710 | -1.01 | 20684 | -1.18 | 72544 | -1.20 | 668212 | -1.07 |
| 103836 | -1.01 | 18987 | -1.18 | 56309 | -1.20 | 384701 | -1.07 |
| 78321 | -1.01 | 15353 | -1.18 | 72361 | -1.21 | 171210 | -1.07 |
| 19290 | -1.01 | 15567 | -1.18 | 228858 | -1.22 | 54342 | -1.08 |
| 71761 | -1.01 | 67603 | -1.18 | 14985 | -1.22 | 110172 | -1.08 |
| 69528 | -1.01 | novel_G000332 | -1.18 | 85031 | -1.22 | 1E+08 | -1.08 |
| 52132 | -1.01 | 13120 | -1.19 | 64385 | -1.22 | 328162 | -1.08 |
| 271842 | -1.01 | 20562 | -1.19 | 17872 | -1.22 | 27204 | -1.08 |
| 269585 | -1.01 | 18755 | -1.19 | 12609 | -1.24 | 207785 | -1.08 |
| 14598 | -1.01 | 232087 | -1.19 | 110172 | -1.24 | 72668 | -1.08 |
| 230766 | -1.01 | 78771 | -1.19 | 19653 | -1.25 | 13120 | -1.09 |
| 13110 | -1.01 | 240672 | -1.20 | 67966 | -1.25 | 70561 | -1.09 |
| 71382 | -1.01 | 93838 | -1.20 | 21844 | -1.25 | 78895 | -1.09 |
| 100271882 | -1.02 | 213522 | -1.21 | 13110 | -1.26 | 664779 | -1.10 |
| 72040 | -1.02 | 142980 | -1.21 | 107094 | -1.26 | 11775 | -1.10 |
| 11980 | -1.02 | 70370 | -1.22 | 16149 | -1.27 | 72017 | -1.11 |
| 72296 | -1.02 | 15064 | -1.22 | 68632 | -1.27 | 192120 | -1.11 |
| 235086 | -1.02 | 70561 | -1.22 | 241528 | -1.27 | 329152 | -1.11 |
| 100042198 | -1.02 | 116847 | -1.22 | 329152 | -1.29 | 16408 | -1.11 |
| 231630 | -1.02 | 74315 | -1.22 | 11987 | -1.29 | 12304 | -1.11 |
| 67752 | -1.02 | 26366 | -1.22 | 76737 | -1.29 | 226351 | -1.12 |
| 18193 | -1.02 | 228550 | -1.22 | NM_001348248 | -1.29 | 72361 | -1.12 |
| 71586 | -1.03 | NM_001348248 | -1.22 | 20215 | -1.30 | 667666 | -1.12 |
| 56380 | -1.03 | 13527 | -1.22 | 19281 | -1.31 | 85031 | -1.13 |
| 67102 | -1.03 | 76737 | -1.23 | 75590 | -1.31 | 21857 | -1.13 |
| 68178 | -1.03 | 81879 | -1.23 | 12795 | -1.31 | 22654 | -1.13 |
| 211378 | -1.03 | 214968 | -1.24 | 108673 | -1.32 | 212980 | -1.13 |
| 76491 | -1.03 | 1.01E+08 | -1.24 | 20515 | -1.32 | 235169 | -1.14 |
| 100177 | -1.03 | 15936 | -1.25 | 231474 | -1.33 | 16011 | -1.14 |
| 270035 | -1.03 | 545156 | -1.25 | 545554 | -1.33 | 109095 | -1.15 |
| 239559 | -1.03 | 12839 | -1.25 | 56336 | -1.33 | 22329 | -1.15 |
| 74201 | -1.03 | 16970 | -1.25 | 246190 | -1.34 | 233876 | -1.15 |
| 67246 | -1.03 | 93880 | -1.26 | 14725 | -1.34 | 207182 | -1.16 |
| 22695 | -1.03 | 21926 | -1.27 | 100034251 | -1.34 | 20515 | -1.16 |
| 11492 | -1.04 | 93732 | -1.27 | 13653 | -1.34 | novel_G000719 | -1.17 |
| 380930 | -1.04 | 1.01E+08 | -1.27 | 100087 | -1.36 | 12293 | -1.17 |
| 57138 | -1.04 | 107227 | -1.28 | 68048 | -1.37 | 56336 | -1.17 |
| 11522 | -1.04 | 16477 | -1.28 | 11989 | -1.38 | 20215 | -1.17 |
| 97998 | -1.04 | 192970 | -1.28 | 545156 | -1.38 | 67279 | -1.17 |
| 68421 | -1.04 | 1E+08 | -1.28 | 93732 | -1.39 | 74041 | -1.17 |
| novel_G000262 | -1.04 | 15039 | -1.28 | 544881 | -1.39 | 208624 | -1.18 |
| novel_G000329 | -1.04 | 80884 | -1.28 | 11538 | -1.39 | 108673 | -1.18 |
| 70300 | -1.04 | 17329 | -1.29 | 100042856 | -1.40 | 619715 | -1.18 |
| 383295 | -1.05 | 228993 | -1.29 | 11910 | -1.40 | 23886 | -1.19 |
| 107885 | -1.05 | 101320 | -1.29 | 67416 | -1.40 | 66752 | -1.19 |
| 20540 | -1.05 | 224697 | -1.29 | 384701 | -1.40 | 228993 | -1.19 |
| 12519 | -1.05 | 207182 | -1.29 | novel_G000188 | -1.41 | 98682 | -1.19 |
| 24051 | -1.05 | 11447 | -1.30 | 331004 | -1.41 | 11642 | -1.19 |
| 103220 | -1.05 | 85031 | -1.30 | 109032 | -1.41 | 12839 | -1.19 |
| 268706 | -1.05 | 76884 | -1.30 | 69065 | -1.41 | 637515 | -1.20 |
| 17777 | -1.05 | 230766 | -1.30 | 232087 | -1.43 | 64136 | -1.20 |
| 22781 | -1.05 | 11541 | -1.30 | 11447 | -1.44 | 76563 | -1.20 |
| 67230 | -1.05 | 1E+08 | -1.31 | 16145 | -1.44 | 11989 | -1.20 |
| 72446 | -1.06 | 19085 | -1.31 | 435337 | -1.45 | 11567 | -1.20 |
| 26968 | -1.06 | 26381 | -1.31 | 215243 | -1.45 | 67838 | -1.20 |
| 216136 | -1.06 | 20351 | -1.31 | 78771 | -1.45 | 381812 | -1.21 |
| 13641 | -1.06 | 20210 | -1.31 | 19700 | -1.45 | 76429 | -1.21 |
| 17122 | -1.06 | 74153 | -1.31 | 72393 | -1.46 | 241528 | -1.21 |
| 58182 | -1.06 | 12282 | -1.31 | 98682 | -1.46 | 71780 | -1.22 |
| 242864 | -1.06 | 20715 | -1.31 | 667214 | -1.46 | 67138 | -1.22 |
| 77889 | -1.06 | 11484 | -1.31 | 19085 | -1.47 | 93838 | -1.23 |
| 26436 | -1.06 | 77125 | -1.32 | 68041 | -1.47 | 67530 | -1.24 |
| NM_001348248 | -1.06 | 14985 | -1.32 | 53973 | -1.47 | 12282 | -1.24 |
| 75785 | -1.06 | 329152 | -1.33 | 107751 | -1.48 | 1.01E+08 | -1.25 |
| 268857 | -1.06 | 243923 | -1.33 | 67373 | -1.49 | 100087 | -1.25 |
| 327954 | -1.06 | 271424 | -1.33 | 18606 | -1.49 | 229949 | -1.26 |
| 18126 | -1.06 | 52882 | -1.33 | 20128 | -1.49 | 106877 | -1.26 |
| 235345 | -1.07 | 11459 | -1.33 | 230678 | -1.49 | 18574 | -1.26 |
| 71481 | -1.07 | 227671 | -1.35 | NM_001348222 | -1.50 | 213389 | -1.26 |
| novel_G000301 | -1.07 | 435337 | -1.35 | 14164 | -1.50 | 14067 | -1.27 |
| 217303 | -1.07 | 1E+08 | -1.35 | 14067 | -1.51 | 209558 | -1.27 |
| novel_G000945 | -1.07 | 230787 | -1.36 | 78459 | -1.51 | 1E+08 | -1.27 |
| 18124 | -1.07 | 77975 | -1.36 | 208624 | -1.51 | 399566 | -1.28 |
| 23960 | -1.07 | 21827 | -1.36 | 70809 | -1.52 | 71242 | -1.28 |
| novel_G000816 | -1.07 | 20355 | -1.36 | novel_G000517 | -1.52 | 268930 | -1.28 |
| 54445 | -1.07 | novel_G000284 | -1.36 | 18218 | -1.52 | 19009 | -1.28 |
| 118453 | -1.08 | 26399 | -1.36 | novel_G000532 | -1.53 | 13655 | -1.29 |
| 107986 | -1.08 | novel_G000874 | -1.36 | novel_G000190 | -1.53 | 192970 | -1.29 |
| 12700 | -1.08 | novel_G000879 | -1.37 | 12862 | -1.53 | 20112 | -1.30 |
| 68128 | -1.08 | 12515 | -1.37 | novel_G000713 | -1.53 | 67966 | -1.31 |
| 380969 | -1.08 | 20500 | -1.37 | 12994 | -1.54 | 67168 | -1.31 |
| 66129 | -1.08 | 72544 | -1.37 | 15531 | -1.54 | 72500 | -1.32 |
| 106639 | -1.08 | 228846 | -1.37 | 94226 | -1.54 | 26434 | -1.32 |
| 14697 | -1.09 | 104086 | -1.37 | 15205 | -1.55 | novel_G000189 | -1.32 |
| 18037 | -1.09 | 20537 | -1.38 | 16531 | -1.56 | 16716 | -1.32 |
| 246727 | -1.09 | 227059 | -1.38 | 213556 | -1.56 | 94279 | -1.32 |
| 56316 | -1.09 | 20556 | -1.38 | 108672 | -1.56 | 110696 | -1.33 |
| 71795 | -1.09 | 140494 | -1.38 | 107869 | -1.56 | 117167 | -1.33 |
| 66961 | -1.09 | 13139 | -1.39 | 83430 | -1.57 | novel_G000190 | -1.34 |
| 18030 | -1.09 | 67475 | -1.39 | 100039315 | -1.57 | 231474 | -1.34 |
| 74008 | -1.09 | 13479 | -1.39 | 20344 | -1.58 | 631304 | -1.35 |
| 12363 | -1.09 | 233529 | -1.39 | 545370 | -1.58 | 671535 | -1.35 |
| 22329 | -1.09 | 70110 | -1.39 | 14969 | -1.58 | 56636 | -1.35 |
| 67880 | -1.09 | 72500 | -1.40 | 212980 | -1.58 | 56309 | -1.36 |
| 74309 | -1.09 | 93875 | -1.40 | 671535 | -1.58 | 66922 | -1.37 |
| 242083 | -1.09 | novel_G000876 | -1.40 | 15203 | -1.59 | 72393 | -1.37 |
| 12577 | -1.09 | 12223 | -1.41 | 70762 | -1.59 | 381413 | -1.37 |
| 545428 | -1.09 | 73061 | -1.41 | 64454 | -1.59 | 71206 | -1.37 |
| 106759 | -1.09 | 18011 | -1.41 | 12227 | -1.60 | 67416 | -1.38 |
| 64209 | -1.09 | 109676 | -1.41 | 18710 | -1.60 | 13527 | -1.38 |
| 211798 | -1.09 | 18022 | -1.41 | 74469 | -1.61 | 14985 | -1.39 |
| 319266 | -1.10 | 20128 | -1.41 | 66922 | -1.63 | 20537 | -1.39 |
| 171543 | -1.10 | 93878 | -1.42 | 320664 | -1.63 | 114564 | -1.39 |
| 71706 | -1.10 | 12227 | -1.42 | 106877 | -1.63 | 20620 | -1.39 |
| 214854 | -1.10 | 14281 | -1.42 | 544963 | -1.64 | 545156 | -1.39 |
| 100503167 | -1.10 | 67416 | -1.43 | 94176 | -1.65 | 14469 | -1.40 |
| 102278 | -1.11 | 268527 | -1.43 | 54199 | -1.65 | 73998 | -1.42 |
| 208820 | -1.11 | 208869 | -1.44 | 100417829 | -1.67 | 19699 | -1.42 |
| 239759 | -1.11 | 13610 | -1.44 | 631304 | -1.67 | 17972 | -1.42 |
| 231724 | -1.11 | 13617 | -1.44 | 634104 | -1.68 | 1E+08 | -1.43 |
| 235628 | -1.11 | 15370 | -1.44 | novel_G000192 | -1.68 | 20558 | -1.43 |
| 215708 | -1.11 | 15213 | -1.44 | 252973 | -1.69 | 71998 | -1.44 |
| 330627 | -1.11 | 217258 | -1.44 | 385643 | -1.69 | 109676 | -1.44 |
| 226641 | -1.11 | novel_G000520 | -1.44 | 14585 | -1.71 | 379043 | -1.44 |
| 545471 | -1.12 | 379043 | -1.46 | 13842 | -1.71 | 19281 | -1.45 |
| 70370 | -1.12 | 26382 | -1.46 | 72500 | -1.72 | 22361 | -1.46 |
| 26399 | -1.12 | 11474 | -1.46 | 17472 | -1.73 | 76737 | -1.46 |
| 12508 | -1.12 | novel_G000193 | -1.47 | 109676 | -1.73 | 544881 | -1.46 |
| 101809 | -1.12 | 239790 | -1.47 | 217843 | -1.74 | 17687 | -1.47 |
| 242125 | -1.12 | 231805 | -1.47 | 12560 | -1.74 | 1E+08 | -1.47 |
| 75311 | -1.12 | 13531 | -1.47 | 58176 | -1.75 | 435337 | -1.47 |
| 12660 | -1.12 | 12609 | -1.48 | 434760 | -1.75 | 93875 | -1.48 |
| 320064 | -1.12 | 21886 | -1.48 | 12686 | -1.75 | 11997 | -1.49 |
| 78749 | -1.12 | 240913 | -1.48 | 15896 | -1.75 | 1E+08 | -1.49 |
| 50767 | -1.12 | 17312 | -1.49 | 320360 | -1.77 | 107869 | -1.49 |
| 71751 | -1.13 | 15483 | -1.49 | 13531 | -1.77 | 12862 | -1.49 |
| 20351 | -1.13 | 16007 | -1.49 | 100042776 | -1.77 | 18710 | -1.50 |
| 83965 | -1.13 | 69066 | -1.49 | 16160 | -1.78 | 58176 | -1.50 |
| 319673 | -1.14 | 625530 | -1.50 | 68396 | -1.79 | 12686 | -1.50 |
| 574428 | -1.14 | 210992 | -1.51 | 18705 | -1.79 | 243374 | -1.50 |
| 320139 | -1.14 | 331004 | -1.51 | 26381 | -1.80 | 17540 | -1.51 |
| 15114 | -1.14 | 1.05E+08 | -1.51 | 17312 | -1.81 | 75778 | -1.51 |
| 18707 | -1.14 | 110454 | -1.51 | 69865 | -1.81 | 16145 | -1.52 |
| 100216455 | -1.14 | 207181 | -1.51 | 396184 | -1.82 | 232087 | -1.53 |
| 28105 | -1.14 | 636741 | -1.51 | 18197 | -1.82 | 66696 | -1.53 |
| 12322 | -1.15 | 23886 | -1.51 | 192113 | -1.83 | 20128 | -1.54 |
| novel_G000914 | -1.15 | 66922 | -1.52 | 269831 | -1.83 | 110454 | -1.54 |
| 72017 | -1.15 | 384701 | -1.52 | 12818 | -1.83 | 14869 | -1.54 |
| 170460 | -1.15 | 71738 | -1.53 | 18815 | -1.83 | 13110 | -1.55 |
| 237711 | -1.15 | 21940 | -1.53 | 235180 | -1.83 | 544963 | -1.56 |
| 59033 | -1.15 | 58229 | -1.53 | 54378 | -1.84 | 20355 | -1.57 |
| 75731 | -1.15 | 68632 | -1.53 | 668357 | -1.85 | 93732 | -1.59 |
| 106369 | -1.15 | 20344 | -1.54 | 381413 | -1.85 | NM_001348222 | -1.59 |
| 380855 | -1.15 | 18606 | -1.54 | 70747 | -1.86 | 72097 | -1.59 |
| 244237 | -1.15 | 19073 | -1.54 | 68800 | -1.87 | 236573 | -1.60 |
| 14256 | -1.15 | 73648 | -1.54 | 21926 | -1.87 | 13164 | -1.60 |
| 93878 | -1.15 | 68526 | -1.54 | 215378 | -1.88 | 18022 | -1.60 |
| 80884 | -1.16 | 17972 | -1.55 | 116903 | -1.88 | 69770 | -1.61 |
| 74126 | -1.16 | novel_G000305 | -1.55 | 69635 | -1.88 | 14825 | -1.61 |
| 228357 | -1.16 | 16780 | -1.56 | 634882 | -1.90 | 78771 | -1.61 |
| 232087 | -1.16 | 246727 | -1.57 | 18671 | -1.90 | 240667 | -1.61 |
| 17175 | -1.16 | 73998 | -1.57 | 114564 | -1.91 | 192161 | -1.64 |
| 243197 | -1.16 | novel_G000885 | -1.57 | 94090 | -1.91 | 11910 | -1.64 |
| 15039 | -1.16 | novel_G000875 | -1.57 | 26434 | -1.91 | 213556 | -1.65 |
| 54393 | -1.16 | 1E+08 | -1.58 | novel_G000885 | -1.92 | 21926 | -1.66 |
| 328263 | -1.16 | 213556 | -1.59 | 80721 | -1.92 | 75590 | -1.66 |
| 103172 | -1.16 | 94090 | -1.59 | 16331 | -1.92 | 68041 | -1.66 |
| novel_G000652 | -1.16 | 545554 | -1.59 | 93837 | -1.93 | 68800 | -1.68 |
| 320982 | -1.16 | 15016 | -1.60 | 17540 | -1.94 | 15203 | -1.68 |
| 18220 | -1.16 | novel_G000561 | -1.60 | 208943 | -1.95 | 1E+08 | -1.69 |
| 227059 | -1.17 | 14164 | -1.61 | 12519 | -1.96 | 192190 | -1.69 |
| 110876 | -1.17 | 234515 | -1.61 | 76074 | -1.97 | 74711 | -1.70 |
| 223774 | -1.17 | 108096 | -1.61 | 56533 | -1.99 | 12994 | -1.72 |
| 14824 | -1.17 | 15203 | -1.62 | 18022 | -2.01 | 434760 | -1.72 |
| 330890 | -1.17 | 67606 | -1.62 | 20620 | -2.01 | 69017 | -1.73 |
| 629016 | -1.17 | 15953 | -1.62 | novel_G000561 | -2.02 | 116903 | -1.73 |
| 78070 | -1.17 | 654796 | -1.62 | 100039042 | -2.02 | 634104 | -1.73 |
| 259044 | -1.17 | 1E+08 | -1.63 | 53416 | -2.04 | novel_G000285 | -1.73 |
| 20500 | -1.17 | 75801 | -1.63 | 192190 | -2.04 | 76718 | -1.74 |
| 192976 | -1.17 | 67216 | -1.63 | 13505 | -2.05 | novel_G000987 | -1.74 |
| 17346 | -1.17 | 13505 | -1.64 | 67593 | -2.05 | 17395 | -1.74 |
| 74315 | -1.17 | 18574 | -1.64 | 637004 | -2.07 | 109979 | -1.74 |
| 60440 | -1.18 | 13537 | -1.64 | 67216 | -2.08 | 654796 | -1.75 |
| 68177 | -1.18 | 619289 | -1.64 | 258513 | -2.08 | 269346 | -1.76 |
| 72185 | -1.18 | 13164 | -1.64 | 208869 | -2.10 | 16531 | -1.77 |
| 319720 | -1.18 | 27411 | -1.65 | 23964 | -2.13 | 394436 | -1.77 |
| 11444 | -1.18 | 76718 | -1.65 | 668940 | -2.13 | 12288 | -1.77 |
| 70561 | -1.18 | 53416 | -1.65 | 12515 | -2.15 | 620913 | -1.77 |
| 75084 | -1.19 | 192161 | -1.65 | 16425 | -2.17 | 13842 | -1.78 |
| 58222 | -1.19 | 208943 | -1.66 | 26464 | -2.17 | 1E+08 | -1.78 |
| 13198 | -1.19 | 17472 | -1.66 | novel_G000072 | -2.17 | 68396 | -1.78 |
| 14367 | -1.19 | 266459 | -1.66 | 209387 | -2.17 | 1E+08 | -1.78 |
| 69329 | -1.19 | 11668 | -1.66 | 269346 | -2.18 | 215446 | -1.78 |
| 117590 | -1.19 | novel_G000515 | -1.67 | 11642 | -2.20 | 17312 | -1.78 |
| 12353 | -1.19 | 21452 | -1.67 | 100322896 | -2.20 | 11682 | -1.79 |
| 668917 | -1.20 | 1E+08 | -1.67 | 81879 | -2.21 | 12399 | -1.79 |
| 56734 | -1.20 | 56448 | -1.67 | 23886 | -2.22 | 109032 | -1.79 |
| 27204 | -1.20 | 93837 | -1.68 | 76998 | -2.22 | 70127 | -1.79 |
| 74153 | -1.20 | novel_G000411 | -1.68 | 16819 | -2.23 | 60504 | -1.80 |
| 668215 | -1.20 | 227120 | -1.68 | 224761 | -2.23 | 66039 | -1.80 |
| 242109 | -1.21 | 434223 | -1.70 | 67547 | -2.25 | 268527 | -1.81 |
| 13024 | -1.21 | 24117 | -1.70 | 71738 | -2.25 | 239790 | -1.81 |
| 192193 | -1.21 | 209773 | -1.70 | 20704 | -2.25 | 56533 | -1.81 |
| 637515 | -1.21 | 21952 | -1.71 | novel_G000138 | -2.27 | novel_G000411 | -1.81 |
| 231946 | -1.22 | 56838 | -1.71 | 21683 | -2.32 | 17472 | -1.81 |
| 17996 | -1.22 | 224344 | -1.71 | 16970 | -2.33 | novel_G000885 | -1.82 |
| 80910 | -1.22 | 269346 | -1.71 | novel_G000282 | -2.34 | 75801 | -1.82 |
| 620419 | -1.22 | 622283 | -1.72 | 67928 | -2.36 | 69865 | -1.83 |
| 68659 | -1.22 | 54199 | -1.72 | novel_G000833 | -2.36 | 78459 | -1.83 |
| 77041 | -1.22 | 67547 | -1.74 | 54563 | -2.38 | 396184 | -1.84 |
| 19219 | -1.22 | 231946 | -1.75 | 56636 | -2.42 | 1E+08 | -1.84 |
| 227746 | -1.22 | 18815 | -1.75 | 236312 | -2.43 | 216019 | -1.84 |
| 100043335 | -1.22 | 330122 | -1.75 | 244698 | -2.46 | 208869 | -1.85 |
| 12832 | -1.22 | 56460 | -1.75 | 73998 | -2.48 | 93837 | -1.85 |
| 229949 | -1.22 | 72361 | -1.75 | 53603 | -2.49 | 69635 | -1.85 |
| NM_001348205 | -1.22 | 69698 | -1.76 | 54427 | -2.51 | 217843 | -1.85 |
| NM_001348222 | -1.22 | 12038 | -1.76 | 68774 | -2.52 | 107751 | -1.86 |
| 18791 | -1.22 | 435626 | -1.76 | 620913 | -2.54 | 16181 | -1.86 |
| 20965 | -1.22 | 109979 | -1.76 | 434223 | -2.58 | 244698 | -1.87 |
| 22625 | -1.23 | 18073 | -1.77 | 140709 | -2.61 | 94090 | -1.88 |
| 382985 | -1.23 | 12268 | -1.77 | 16428 | -2.64 | novel_G000192 | -1.88 |
| 224090 | -1.23 | 319317 | -1.77 | 239790 | -2.65 | 77974 | -1.89 |
| 319613 | -1.23 | 216739 | -1.77 | 234515 | -2.65 | 108672 | -1.91 |
| 72997 | -1.23 | 16011 | -1.77 | 103964 | -2.67 | novel_G000134 | -1.91 |
| 245666 | -1.24 | 65255 | -1.78 | 269959 | -2.69 | 668357 | -1.91 |
| 53614 | -1.24 | 12354 | -1.78 | 67606 | -2.72 | 23960 | -1.91 |
| 75605 | -1.24 | 20339 | -1.78 | 269854 | -2.73 | 320664 | -1.92 |
| 19152 | -1.24 | 215243 | -1.78 | 14825 | -2.75 | 67373 | -1.94 |
| 73368 | -1.24 | 23964 | -1.78 | 58218 | -2.78 | novel_G000058 | -1.95 |
| 67731 | -1.24 | 21683 | -1.78 | 101320 | -2.85 | 619326 | -1.95 |
| 171210 | -1.24 | 105859 | -1.78 | novel_G000286 | -2.86 | 252973 | -1.97 |
| 18826 | -1.24 | 14347 | -1.79 | 213002 | -2.96 | 236451 | -1.98 |
| 235493 | -1.25 | 93671 | -1.79 | 74020 | -3.07 | 668940 | -1.99 |
| 232086 | -1.25 | 74180 | -1.79 | novel_G000025 | -3.09 | 53973 | -1.99 |
| 544817 | -1.25 | 12483 | -1.80 | 243369 | -3.10 | 68632 | -2.00 |
| 338351 | -1.25 | 243277 | -1.80 | 59083 | -3.18 | 20344 | -2.00 |
| 101023 | -1.25 | novel_G000030 | -1.80 | 20302 | -3.20 | 330671 | -2.01 |
| 107477 | -1.25 | 15122 | -1.81 | 20311 | -3.28 | 104759 | -2.01 |
| 74048 | -1.25 | 212980 | -1.82 | 20310 | -3.31 | 236749 | -2.02 |
| 381924 | -1.25 | 353234 | -1.82 | 16193 | -3.36 | 545370 | -2.03 |
| 68737 | -1.26 | 11838 | -1.83 | 18419 | -3.38 | 14051 | -2.03 |
| 100041379 | -1.26 | novel_G000731 | -1.84 | 20297 | -4.35 | 18671 | -2.04 |
| 12659 | -1.26 | 192163 | -1.84 | novel_G000835 | -5.57 | 331004 | -2.05 |
| 209692 | -1.26 | 93674 | -1.84 |  |  | 64385 | -2.07 |
| 20338 | -1.26 | 66857 | -1.84 |  |  | 16819 | -2.10 |
| 105841 | -1.26 | novel_G000522 | -1.85 |  |  | 16970 | -2.11 |
| novel_G000651 | -1.26 | 224129 | -1.86 |  |  | novel_G000833 | -2.12 |
| 76187 | -1.26 | 26367 | -1.86 |  |  | 246190 | -2.13 |
| 11988 | -1.26 | novel_G000031 | -1.86 |  |  | 637004 | -2.14 |
| 20688 | -1.26 | 16160 | -1.86 |  |  | novel_G000305 | -2.14 |
| 320662 | -1.27 | 15559 | -1.87 |  |  | 12515 | -2.14 |
| 69944 | -1.27 | 18488 | -1.87 |  |  | 12519 | -2.14 |
| 70266 | -1.27 | novel_G000138 | -1.87 |  |  | 64454 | -2.17 |
| 100038464 | -1.27 | 15360 | -1.87 |  |  | 233801 | -2.17 |
| 17872 | -1.27 | 21897 | -1.88 |  |  | 667214 | -2.17 |
| 630836 | -1.27 | 71740 | -1.88 |  |  | 26381 | -2.18 |
| 14538 | -1.27 | novel_G000981 | -1.88 |  |  | 269831 | -2.19 |
| 195522 | -1.27 | 1E+08 | -1.88 |  |  | 330122 | -2.20 |
| 56219 | -1.27 | 75858 | -1.89 |  |  | 101320 | -2.22 |
| 13610 | -1.28 | 98303 | -1.91 |  |  | 71738 | -2.23 |
| 69870 | -1.28 | 19281 | -1.91 |  |  | novel_G000518 | -2.23 |
| 117606 | -1.28 | 16574 | -1.92 |  |  | 16425 | -2.23 |
| 232748 | -1.28 | 19737 | -1.92 |  |  | 74020 | -2.24 |
| 237930 | -1.28 | novel_G000833 | -1.92 |  |  | 20310 | -2.25 |
| 20661 | -1.28 | 71912 | -1.93 |  |  | 634882 | -2.25 |
| 93876 | -1.28 | 241528 | -1.94 |  |  | 67606 | -2.26 |
| 320292 | -1.28 | 1E+08 | -1.94 |  |  | 23964 | -2.31 |
| novel_G000877 | -1.29 | 15531 | -1.94 |  |  | novel_G000879 | -2.31 |
| 70381 | -1.29 | 433944 | -1.95 |  |  | 67216 | -2.32 |
| 243538 | -1.29 | 74020 | -1.95 |  |  | 668039 | -2.35 |
| 245128 | -1.29 | 58218 | -1.96 |  |  | 1E+08 | -2.35 |
| 50765 | -1.29 | 18197 | -1.97 |  |  | 1E+08 | -2.35 |
| 60599 | -1.29 | 52389 | -1.97 |  |  | 215243 | -2.35 |
| 101502 | -1.29 | 16819 | -1.97 |  |  | 26464 | -2.37 |
| 102294 | -1.29 | 12267 | -1.99 |  |  | 12818 | -2.41 |
| 67603 | -1.29 | 56843 | -1.99 |  |  | 67928 | -2.42 |
| 14314 | -1.29 | 22361 | -1.99 |  |  | novel_G000138 | -2.42 |
| 74959 | -1.29 | 73075 | -1.99 |  |  | 18703 | -2.43 |
| 226255 | -1.30 | novel_G000290 | -2.00 |  |  | 16160 | -2.43 |
| novel_G000733 | -1.30 | 67928 | -2.00 |  |  | 258513 | -2.44 |
| 72230 | -1.30 | 56533 | -2.00 |  |  | 12038 | -2.44 |
| 240869 | -1.30 | 114564 | -2.01 |  |  | 140709 | -2.46 |
| 26366 | -1.30 | 16145 | -2.01 |  |  | 103964 | -2.47 |
| 56338 | -1.30 | 272009 | -2.02 |  |  | 208943 | -2.48 |
| 15018 | -1.30 | 108154 | -2.02 |  |  | 209387 | -2.48 |
| 68625 | -1.30 | 269120 | -2.03 |  |  | 12268 | -2.55 |
| novel_G000517 | -1.30 | 211480 | -2.03 |  |  | 236312 | -2.55 |
| 69221 | -1.31 | 12182 | -2.03 |  |  | 81879 | -2.58 |
| 67865 | -1.31 | 17285 | -2.03 |  |  | 269959 | -2.59 |
| 56149 | -1.31 | 74469 | -2.04 |  |  | 192113 | -2.59 |
| 319263 | -1.31 | 235636 | -2.05 |  |  | 20311 | -2.60 |
| 13654 | -1.31 | 109032 | -2.06 |  |  | 14585 | -2.62 |
| 17957 | -1.31 | 68279 | -2.07 |  |  | 16193 | -2.64 |
| 240753 | -1.31 | 11538 | -2.10 |  |  | 213002 | -2.67 |
| 100534273 | -1.32 | 74229 | -2.11 |  |  | 18606 | -2.70 |
| 17540 | -1.32 | 70747 | -2.12 |  |  | 76998 | -2.74 |
| 271639 | -1.32 | novel_G000367 | -2.13 |  |  | 13505 | -2.75 |
| 432611 | -1.32 | 236312 | -2.13 |  |  | 269854 | -2.76 |
| 19009 | -1.33 | 14051 | -2.13 |  |  | 53416 | -2.79 |
| 13617 | -1.33 | 229706 | -2.13 |  |  | 27052 | -2.82 |
| 73690 | -1.33 | 269959 | -2.15 |  |  | 74469 | -2.84 |
| 54614 | -1.33 | 110696 | -2.15 |  |  | 54563 | -2.84 |
| 319236 | -1.33 | 19739 | -2.16 |  |  | 68774 | -2.91 |
| 14632 | -1.33 | 64385 | -2.17 |  |  | 21683 | -2.92 |
| 21899 | -1.33 | 381411 | -2.17 |  |  | 20302 | -2.94 |
| 235527 | -1.33 | 58176 | -2.17 |  |  | 234515 | -2.97 |
| novel_G000076 | -1.33 | 15205 | -2.19 |  |  | 20704 | -3.04 |
| 68347 | -1.33 | 394436 | -2.20 |  |  | 224761 | -3.20 |
| 18796 | -1.34 | 50498 | -2.20 |  |  | 18419 | -3.21 |
| 12527 | -1.34 | 116903 | -2.20 |  |  | 70747 | -3.26 |
| 100043040 | -1.34 | novel_G000516 | -2.21 |  |  | 434223 | -3.40 |
| 69504 | -1.34 | 76507 | -2.21 |  |  | 16428 | -3.44 |
| 320571 | -1.34 | 11682 | -2.23 |  |  | 59083 | -3.50 |
| 13924 | -1.34 | 12608 | -2.24 |  |  | 243369 | -3.56 |
| 68126 | -1.35 | 671535 | -2.24 |  |  | 20297 | -3.61 |
| 235504 | -1.35 | 18616 | -2.26 |  |  | novel_G000051 | -3.62 |
| 216188 | -1.35 | 14825 | -2.27 |  |  | novel_G000280 | -3.87 |
| 215814 | -1.35 | 16428 | -2.27 |  |  | 58218 | -4.00 |
| 26367 | -1.35 | 381538 | -2.28 |  |  | novel_G000030 | -4.47 |
| 240261 | -1.35 | 19065 | -2.28 |  |  | novel_G000708 | -4.96 |
| 18113 | -1.35 | novel_G000622 | -2.31 |  |  |  |  |
| 100732 | -1.36 | 330409 | -2.31 |  |  |  |  |
| 93889 | -1.36 | 192190 | -2.32 |  |  |  |  |
| 23886 | -1.36 | novel_G000025 | -2.34 |  |  |  |  |
| 20620 | -1.36 | 108672 | -2.34 |  |  |  |  |
| 319887 | -1.36 | 12994 | -2.37 |  |  |  |  |
| 214111 | -1.36 | 634882 | -2.37 |  |  |  |  |
| 171285 | -1.37 | 214240 | -2.38 |  |  |  |  |
| 12183 | -1.37 | 117167 | -2.41 |  |  |  |  |
| 381126 | -1.37 | 76074 | -2.42 |  |  |  |  |
| 18987 | -1.37 | 637004 | -2.42 |  |  |  |  |
| 217356 | -1.37 | novel_G000134 | -2.42 |  |  |  |  |
| 213522 | -1.37 | 71884 | -2.44 |  |  |  |  |
| 100041433 | -1.37 | 546024 | -2.45 |  |  |  |  |
| 14991 | -1.37 | 13421 | -2.46 |  |  |  |  |
| 227671 | -1.37 | novel_G000643 | -2.50 |  |  |  |  |
| 240041 | -1.37 | 68774 | -2.50 |  |  |  |  |
| 67009 | -1.38 | 94176 | -2.50 |  |  |  |  |
| 16477 | -1.38 | 67593 | -2.52 |  |  |  |  |
| 113848 | -1.38 | 667214 | -2.53 |  |  |  |  |
| 66274 | -1.38 | 71345 | -2.55 |  |  |  |  |
| 14979 | -1.38 | 104759 | -2.57 |  |  |  |  |
| 22351 | -1.38 | 258513 | -2.57 |  |  |  |  |
| 225631 | -1.38 | 103964 | -2.59 |  |  |  |  |
| 70040 | -1.39 | 12007 | -2.61 |  |  |  |  |
| 219131 | -1.39 | 16149 | -2.62 |  |  |  |  |
| 234353 | -1.39 | 20704 | -2.63 |  |  |  |  |
| 208117 | -1.39 | 14067 | -2.63 |  |  |  |  |
| 330427 | -1.39 | 14469 | -2.64 |  |  |  |  |
| 240672 | -1.39 | 668940 | -2.64 |  |  |  |  |
| 319924 | -1.40 | 66696 | -2.66 |  |  |  |  |
| 17687 | -1.40 | 236573 | -2.66 |  |  |  |  |
| 667666 | -1.40 | 12231 | -2.68 |  |  |  |  |
| 619715 | -1.40 | 20302 | -2.69 |  |  |  |  |
| 50723 | -1.40 | 16193 | -2.70 |  |  |  |  |
| 11603 | -1.40 | 217169 | -2.71 |  |  |  |  |
| 68070 | -1.40 | 16425 | -2.74 |  |  |  |  |
| 14828 | -1.40 | novel_G000713 | -2.78 |  |  |  |  |
| 100616095 | -1.41 | 20310 | -2.81 |  |  |  |  |
| novel_G000986 | -1.41 | 140709 | -2.88 |  |  |  |  |
| 12266 | -1.41 | 12818 | -2.89 |  |  |  |  |
| 66102 | -1.41 | 54563 | -2.90 |  |  |  |  |
| 17105 | -1.41 | 20297 | -2.91 |  |  |  |  |
| 381339 | -1.41 | 244698 | -2.98 |  |  |  |  |
| 67138 | -1.41 | 217843 | -2.98 |  |  |  |  |
| 14702 | -1.41 | 620913 | -3.06 |  |  |  |  |
| 227580 | -1.42 | 26464 | -3.07 |  |  |  |  |
| 93734 | -1.42 | 1.01E+08 | -3.09 |  |  |  |  |
| 14748 | -1.42 | 209387 | -3.14 |  |  |  |  |
| 226098 | -1.42 | 64454 | -3.15 |  |  |  |  |
| 14422 | -1.42 | novel_G000230 | -3.21 |  |  |  |  |
| 11727 | -1.42 | 59083 | -3.21 |  |  |  |  |
| 24066 | -1.42 | 213002 | -3.22 |  |  |  |  |
| 224093 | -1.42 | 269854 | -3.27 |  |  |  |  |
| 216439 | -1.42 | 243369 | -3.30 |  |  |  |  |
| 223433 | -1.42 | 27052 | -3.50 |  |  |  |  |
| 66922 | -1.43 | 20311 | -3.54 |  |  |  |  |
| 320705 | -1.43 | 14969 | -3.54 |  |  |  |  |
| 223649 | -1.43 | 224761 | -3.69 |  |  |  |  |
| 112422 | -1.44 | 18419 | -4.50 |  |  |  |  |
| 69564 | -1.44 |  |  |  |  |  |  |
| 192216 | -1.44 |  |  |  |  |  |  |
| 15936 | -1.44 |  |  |  |  |  |  |
| 12509 | -1.44 |  |  |  |  |  |  |
| 20556 | -1.44 |  |  |  |  |  |  |
| 12304 | -1.45 |  |  |  |  |  |  |
| 20229 | -1.45 |  |  |  |  |  |  |
| 15444 | -1.45 |  |  |  |  |  |  |
| 19143 | -1.45 |  |  |  |  |  |  |
| 67573 | -1.46 |  |  |  |  |  |  |
| 26434 | -1.46 |  |  |  |  |  |  |
| 18783 | -1.46 |  |  |  |  |  |  |
| 17772 | -1.46 |  |  |  |  |  |  |
| 237636 | -1.46 |  |  |  |  |  |  |
| 106861 | -1.46 |  |  |  |  |  |  |
| 76737 | -1.46 |  |  |  |  |  |  |
| novel_G000340 | -1.46 |  |  |  |  |  |  |
| 15898 | -1.47 |  |  |  |  |  |  |
| 228139 | -1.47 |  |  |  |  |  |  |
| 51791 | -1.47 |  |  |  |  |  |  |
| 74761 | -1.47 |  |  |  |  |  |  |
| 207819 | -1.47 |  |  |  |  |  |  |
| 68055 | -1.47 |  |  |  |  |  |  |
| 11304 | -1.47 |  |  |  |  |  |  |
| 64294 | -1.47 |  |  |  |  |  |  |
| 108800 | -1.47 |  |  |  |  |  |  |
| 72289 | -1.48 |  |  |  |  |  |  |
| 20583 | -1.48 |  |  |  |  |  |  |
| 394432 | -1.48 |  |  |  |  |  |  |
| 21886 | -1.48 |  |  |  |  |  |  |
| 14282 | -1.48 |  |  |  |  |  |  |
| 66090 | -1.48 |  |  |  |  |  |  |
| 19273 | -1.48 |  |  |  |  |  |  |
| 215303 | -1.48 |  |  |  |  |  |  |
| 114642 | -1.49 |  |  |  |  |  |  |
| 100037258 | -1.49 |  |  |  |  |  |  |
| 353025 | -1.49 |  |  |  |  |  |  |
| 73603 | -1.49 |  |  |  |  |  |  |
| 241520 | -1.49 |  |  |  |  |  |  |
| 218311 | -1.49 |  |  |  |  |  |  |
| 12293 | -1.49 |  |  |  |  |  |  |
| novel_G000796 | -1.50 |  |  |  |  |  |  |
| novel_G000374 | -1.50 |  |  |  |  |  |  |
| 20558 | -1.50 |  |  |  |  |  |  |
| 100604 | -1.50 |  |  |  |  |  |  |
| 234593 | -1.51 |  |  |  |  |  |  |
| 213389 | -1.51 |  |  |  |  |  |  |
| 26382 | -1.51 |  |  |  |  |  |  |
| 333424 | -1.51 |  |  |  |  |  |  |
| 242122 | -1.51 |  |  |  |  |  |  |
| 380698 | -1.51 |  |  |  |  |  |  |
| 170835 | -1.52 |  |  |  |  |  |  |
| 192120 | -1.52 |  |  |  |  |  |  |
| 12862 | -1.52 |  |  |  |  |  |  |
| 56188 | -1.53 |  |  |  |  |  |  |
| 442834 | -1.53 |  |  |  |  |  |  |
| 16323 | -1.53 |  |  |  |  |  |  |
| 15016 | -1.53 |  |  |  |  |  |  |
| NM_001348168 | -1.54 |  |  |  |  |  |  |
| 74580 | -1.54 |  |  |  |  |  |  |
| 17684 | -1.54 |  |  |  |  |  |  |
| 435337 | -1.54 |  |  |  |  |  |  |
| 231474 | -1.54 |  |  |  |  |  |  |
| 100504591 | -1.54 |  |  |  |  |  |  |
| 17207 | -1.55 |  |  |  |  |  |  |
| 380714 | -1.55 |  |  |  |  |  |  |
| 238161 | -1.55 |  |  |  |  |  |  |
| 18125 | -1.55 |  |  |  |  |  |  |
| 12695 | -1.56 |  |  |  |  |  |  |
| 71086 | -1.56 |  |  |  |  |  |  |
| 27362 | -1.56 |  |  |  |  |  |  |
| 18606 | -1.56 |  |  |  |  |  |  |
| 379043 | -1.56 |  |  |  |  |  |  |
| 207785 | -1.57 |  |  |  |  |  |  |
| 74011 | -1.57 |  |  |  |  |  |  |
| 23801 | -1.57 |  |  |  |  |  |  |
| 271711 | -1.57 |  |  |  |  |  |  |
| 333670 | -1.58 |  |  |  |  |  |  |
| 15213 | -1.58 |  |  |  |  |  |  |
| 233335 | -1.58 |  |  |  |  |  |  |
| 18846 | -1.59 |  |  |  |  |  |  |
| 12227 | -1.59 |  |  |  |  |  |  |
| 71145 | -1.59 |  |  |  |  |  |  |
| 407803 | -1.59 |  |  |  |  |  |  |
| 14085 | -1.59 |  |  |  |  |  |  |
| 100702 | -1.59 |  |  |  |  |  |  |
| 15571 | -1.60 |  |  |  |  |  |  |
| 619548 | -1.60 |  |  |  |  |  |  |
| 17910 | -1.60 |  |  |  |  |  |  |
| 218461 | -1.60 |  |  |  |  |  |  |
| 245527 | -1.60 |  |  |  |  |  |  |
| 338523 | -1.60 |  |  |  |  |  |  |
| 231691 | -1.61 |  |  |  |  |  |  |
| 103511 | -1.61 |  |  |  |  |  |  |
| 17972 | -1.61 |  |  |  |  |  |  |
| 15064 | -1.62 |  |  |  |  |  |  |
| novel_G000876 | -1.62 |  |  |  |  |  |  |
| 78251 | -1.62 |  |  |  |  |  |  |
| 68632 | -1.63 |  |  |  |  |  |  |
| 69698 | -1.63 |  |  |  |  |  |  |
| 241732 | -1.63 |  |  |  |  |  |  |
| 68016 | -1.63 |  |  |  |  |  |  |
| 24014 | -1.63 |  |  |  |  |  |  |
| 71584 | -1.63 |  |  |  |  |  |  |
| 54342 | -1.63 |  |  |  |  |  |  |
| 381678 | -1.63 |  |  |  |  |  |  |
| novel_G000801 | -1.63 |  |  |  |  |  |  |
| 266459 | -1.63 |  |  |  |  |  |  |
| 56631 | -1.63 |  |  |  |  |  |  |
| 75778 | -1.63 |  |  |  |  |  |  |
| novel_G000878 | -1.64 |  |  |  |  |  |  |
| 269589 | -1.64 |  |  |  |  |  |  |
| 213311 | -1.64 |  |  |  |  |  |  |
| 68852 | -1.64 |  |  |  |  |  |  |
| 14867 | -1.64 |  |  |  |  |  |  |
| 252973 | -1.64 |  |  |  |  |  |  |
| 106766 | -1.64 |  |  |  |  |  |  |
| 243914 | -1.64 |  |  |  |  |  |  |
| novel_G000190 | -1.65 |  |  |  |  |  |  |
| 15122 | -1.65 |  |  |  |  |  |  |
| 12609 | -1.65 |  |  |  |  |  |  |
| 71519 | -1.65 |  |  |  |  |  |  |
| 98682 | -1.65 |  |  |  |  |  |  |
| 57911 | -1.66 |  |  |  |  |  |  |
| 245884 | -1.66 |  |  |  |  |  |  |
| 17940 | -1.66 |  |  |  |  |  |  |
| 24117 | -1.66 |  |  |  |  |  |  |
| 76982 | -1.66 |  |  |  |  |  |  |
| 319446 | -1.66 |  |  |  |  |  |  |
| 19124 | -1.66 |  |  |  |  |  |  |
| novel_G000734 | -1.66 |  |  |  |  |  |  |
| 215707 | -1.66 |  |  |  |  |  |  |
| 229445 | -1.67 |  |  |  |  |  |  |
| 242443 | -1.67 |  |  |  |  |  |  |
| 72578 | -1.67 |  |  |  |  |  |  |
| 105727 | -1.68 |  |  |  |  |  |  |
| 16803 | -1.68 |  |  |  |  |  |  |
| novel_G000088 | -1.68 |  |  |  |  |  |  |
| 545554 | -1.68 |  |  |  |  |  |  |
| 217258 | -1.68 |  |  |  |  |  |  |
| 224697 | -1.68 |  |  |  |  |  |  |
| 634650 | -1.69 |  |  |  |  |  |  |
| 619289 | -1.69 |  |  |  |  |  |  |
| 211135 | -1.69 |  |  |  |  |  |  |
| novel_G000575 | -1.69 |  |  |  |  |  |  |
| 214968 | -1.69 |  |  |  |  |  |  |
| 29867 | -1.69 |  |  |  |  |  |  |
| 66734 | -1.69 |  |  |  |  |  |  |
| 68127 | -1.69 |  |  |  |  |  |  |
| 320225 | -1.70 |  |  |  |  |  |  |
| 320560 | -1.70 |  |  |  |  |  |  |
| 60533 | -1.70 |  |  |  |  |  |  |
| 77125 | -1.70 |  |  |  |  |  |  |
| 16572 | -1.70 |  |  |  |  |  |  |
| 58809 | -1.70 |  |  |  |  |  |  |
| 55948 | -1.71 |  |  |  |  |  |  |
| novel_G000516 | -1.71 |  |  |  |  |  |  |
| 192970 | -1.71 |  |  |  |  |  |  |
| 70726 | -1.71 |  |  |  |  |  |  |
| 17951 | -1.72 |  |  |  |  |  |  |
| 433470 | -1.72 |  |  |  |  |  |  |
| 13139 | -1.72 |  |  |  |  |  |  |
| 100041574 | -1.72 |  |  |  |  |  |  |
| 18073 | -1.72 |  |  |  |  |  |  |
| 231805 | -1.72 |  |  |  |  |  |  |
| 109095 | -1.73 |  |  |  |  |  |  |
| 100039691 | -1.73 |  |  |  |  |  |  |
| 93875 | -1.73 |  |  |  |  |  |  |
| 100039087 | -1.74 |  |  |  |  |  |  |
| 56460 | -1.74 |  |  |  |  |  |  |
| 13527 | -1.74 |  |  |  |  |  |  |
| 13835 | -1.74 |  |  |  |  |  |  |
| 68195 | -1.74 |  |  |  |  |  |  |
| 100504464 | -1.74 |  |  |  |  |  |  |
| 12815 | -1.74 |  |  |  |  |  |  |
| 16847 | -1.74 |  |  |  |  |  |  |
| 11538 | -1.75 |  |  |  |  |  |  |
| 13067 | -1.75 |  |  |  |  |  |  |
| 242362 | -1.75 |  |  |  |  |  |  |
| 207798 | -1.75 |  |  |  |  |  |  |
| 13531 | -1.75 |  |  |  |  |  |  |
| 69865 | -1.75 |  |  |  |  |  |  |
| 11720 | -1.75 |  |  |  |  |  |  |
| 11472 | -1.76 |  |  |  |  |  |  |
| 243382 | -1.76 |  |  |  |  |  |  |
| 378425 | -1.76 |  |  |  |  |  |  |
| 17063 | -1.76 |  |  |  |  |  |  |
| 66107 | -1.77 |  |  |  |  |  |  |
| 234199 | -1.77 |  |  |  |  |  |  |
| 236366 | -1.77 |  |  |  |  |  |  |
| 15559 | -1.77 |  |  |  |  |  |  |
| 11775 | -1.77 |  |  |  |  |  |  |
| 13479 | -1.78 |  |  |  |  |  |  |
| 14468 | -1.78 |  |  |  |  |  |  |
| 76884 | -1.78 |  |  |  |  |  |  |
| 15162 | -1.78 |  |  |  |  |  |  |
| 228993 | -1.78 |  |  |  |  |  |  |
| 100503361 | -1.78 |  |  |  |  |  |  |
| 212980 | -1.78 |  |  |  |  |  |  |
| 241633 | -1.79 |  |  |  |  |  |  |
| 234673 | -1.79 |  |  |  |  |  |  |
| novel_G000745 | -1.80 |  |  |  |  |  |  |
| 319278 | -1.80 |  |  |  |  |  |  |
| 70291 | -1.80 |  |  |  |  |  |  |
| 209558 | -1.80 |  |  |  |  |  |  |
| 74152 | -1.81 |  |  |  |  |  |  |
| 21819 | -1.81 |  |  |  |  |  |  |
| 75292 | -1.81 |  |  |  |  |  |  |
| novel_G000192 | -1.82 |  |  |  |  |  |  |
| 12839 | -1.82 |  |  |  |  |  |  |
| 544881 | -1.83 |  |  |  |  |  |  |
| 74180 | -1.83 |  |  |  |  |  |  |
| 18752 | -1.83 |  |  |  |  |  |  |
| 192161 | -1.84 |  |  |  |  |  |  |
| 69585 | -1.84 |  |  |  |  |  |  |
| 66039 | -1.84 |  |  |  |  |  |  |
| 14469 | -1.84 |  |  |  |  |  |  |
| 213556 | -1.84 |  |  |  |  |  |  |
| 78459 | -1.85 |  |  |  |  |  |  |
| 230979 | -1.85 |  |  |  |  |  |  |
| 14067 | -1.85 |  |  |  |  |  |  |
| 99662 | -1.85 |  |  |  |  |  |  |
| 67373 | -1.85 |  |  |  |  |  |  |
| 100503265 | -1.85 |  |  |  |  |  |  |
| 12496 | -1.86 |  |  |  |  |  |  |
| 13537 | -1.86 |  |  |  |  |  |  |
| 208285 | -1.86 |  |  |  |  |  |  |
| 216198 | -1.86 |  |  |  |  |  |  |
| 671535 | -1.86 |  |  |  |  |  |  |
| 238055 | -1.87 |  |  |  |  |  |  |
| 93838 | -1.87 |  |  |  |  |  |  |
| 230787 | -1.87 |  |  |  |  |  |  |
| 432589 | -1.87 |  |  |  |  |  |  |
| 11468 | -1.87 |  |  |  |  |  |  |
| 243374 | -1.87 |  |  |  |  |  |  |
| 22635 | -1.87 |  |  |  |  |  |  |
| 100043868 | -1.88 |  |  |  |  |  |  |
| 54427 | -1.88 |  |  |  |  |  |  |
| 207182 | -1.88 |  |  |  |  |  |  |
| 12223 | -1.88 |  |  |  |  |  |  |
| 68728 | -1.88 |  |  |  |  |  |  |
| 328424 | -1.88 |  |  |  |  |  |  |
| 116847 | -1.88 |  |  |  |  |  |  |
| 243277 | -1.88 |  |  |  |  |  |  |
| 71998 | -1.88 |  |  |  |  |  |  |
| 94090 | -1.89 |  |  |  |  |  |  |
| 71069 | -1.89 |  |  |  |  |  |  |
| 15205 | -1.89 |  |  |  |  |  |  |
| 78779 | -1.89 |  |  |  |  |  |  |
| 18213 | -1.89 |  |  |  |  |  |  |
| 15203 | -1.89 |  |  |  |  |  |  |
| novel_G000411 | -1.90 |  |  |  |  |  |  |
| 67416 | -1.90 |  |  |  |  |  |  |
| 83767 | -1.91 |  |  |  |  |  |  |
| 22371 | -1.91 |  |  |  |  |  |  |
| 170722 | -1.91 |  |  |  |  |  |  |
| 433502 | -1.91 |  |  |  |  |  |  |
| 100039042 | -1.91 |  |  |  |  |  |  |
| novel_G000406 | -1.91 |  |  |  |  |  |  |
| 12628 | -1.91 |  |  |  |  |  |  |
| 93711 | -1.91 |  |  |  |  |  |  |
| 56843 | -1.92 |  |  |  |  |  |  |
| 207181 | -1.92 |  |  |  |  |  |  |
| 14275 | -1.92 |  |  |  |  |  |  |
| 240913 | -1.92 |  |  |  |  |  |  |
| 381463 | -1.92 |  |  |  |  |  |  |
| 12984 | -1.92 |  |  |  |  |  |  |
| 108151 | -1.93 |  |  |  |  |  |  |
| 69480 | -1.93 |  |  |  |  |  |  |
| 214084 | -1.93 |  |  |  |  |  |  |
| 18295 | -1.93 |  |  |  |  |  |  |
| 18491 | -1.93 |  |  |  |  |  |  |
| novel_G000189 | -1.93 |  |  |  |  |  |  |
| 71213 | -1.93 |  |  |  |  |  |  |
| 20128 | -1.93 |  |  |  |  |  |  |
| 22788 | -1.93 |  |  |  |  |  |  |
| 105594 | -1.93 |  |  |  |  |  |  |
| 74488 | -1.93 |  |  |  |  |  |  |
| 384701 | -1.93 |  |  |  |  |  |  |
| 277743 | -1.94 |  |  |  |  |  |  |
| 216739 | -1.94 |  |  |  |  |  |  |
| 574403 | -1.94 |  |  |  |  |  |  |
| 319555 | -1.94 |  |  |  |  |  |  |
| 100042776 | -1.94 |  |  |  |  |  |  |
| 268379 | -1.94 |  |  |  |  |  |  |
| 56741 | -1.94 |  |  |  |  |  |  |
| 100038628 | -1.94 |  |  |  |  |  |  |
| 72361 | -1.94 |  |  |  |  |  |  |
| 270190 | -1.95 |  |  |  |  |  |  |
| novel_G000713 | -1.95 |  |  |  |  |  |  |
| 245827 | -1.95 |  |  |  |  |  |  |
| 57429 | -1.95 |  |  |  |  |  |  |
| 17068 | -1.96 |  |  |  |  |  |  |
| 634882 | -1.96 |  |  |  |  |  |  |
| 13421 | -1.96 |  |  |  |  |  |  |
| 228846 | -1.97 |  |  |  |  |  |  |
| 85031 | -1.97 |  |  |  |  |  |  |
| 21405 | -1.97 |  |  |  |  |  |  |
| 12981 | -1.97 |  |  |  |  |  |  |
| 83560 | -1.97 |  |  |  |  |  |  |
| 17285 | -1.97 |  |  |  |  |  |  |
| 15567 | -1.98 |  |  |  |  |  |  |
| 18574 | -1.98 |  |  |  |  |  |  |
| 18131 | -1.98 |  |  |  |  |  |  |
| 238393 | -1.98 |  |  |  |  |  |  |
| 19073 | -1.98 |  |  |  |  |  |  |
| 69547 | -1.98 |  |  |  |  |  |  |
| 103012 | -1.98 |  |  |  |  |  |  |
| 140493 | -1.98 |  |  |  |  |  |  |
| 195208 | -1.98 |  |  |  |  |  |  |
| 18008 | -1.98 |  |  |  |  |  |  |
| 81907 | -1.98 |  |  |  |  |  |  |
| 114142 | -1.99 |  |  |  |  |  |  |
| 16007 | -1.99 |  |  |  |  |  |  |
| 12282 | -1.99 |  |  |  |  |  |  |
| 100040462 | -1.99 |  |  |  |  |  |  |
| 100039315 | -1.99 |  |  |  |  |  |  |
| 272381 | -1.99 |  |  |  |  |  |  |
| 474332 | -1.99 |  |  |  |  |  |  |
| novel_G000879 | -2.00 |  |  |  |  |  |  |
| 56448 | -2.00 |  |  |  |  |  |  |
| 14783 | -2.00 |  |  |  |  |  |  |
| novel_G000622 | -2.00 |  |  |  |  |  |  |
| 546024 | -2.00 |  |  |  |  |  |  |
| 18011 | -2.00 |  |  |  |  |  |  |
| 71960 | -2.00 |  |  |  |  |  |  |
| 80857 | -2.01 |  |  |  |  |  |  |
| 22317 | -2.01 |  |  |  |  |  |  |
| 667214 | -2.01 |  |  |  |  |  |  |
| 77974 | -2.03 |  |  |  |  |  |  |
| novel_G000875 | -2.03 |  |  |  |  |  |  |
| 70638 | -2.03 |  |  |  |  |  |  |
| 224129 | -2.03 |  |  |  |  |  |  |
| 217843 | -2.03 |  |  |  |  |  |  |
| 21941 | -2.03 |  |  |  |  |  |  |
| 224019 | -2.03 |  |  |  |  |  |  |
| 110891 | -2.03 |  |  |  |  |  |  |
| 15483 | -2.03 |  |  |  |  |  |  |
| 20339 | -2.04 |  |  |  |  |  |  |
| 93671 | -2.04 |  |  |  |  |  |  |
| 211612 | -2.04 |  |  |  |  |  |  |
| 329735 | -2.04 |  |  |  |  |  |  |
| 330188 | -2.05 |  |  |  |  |  |  |
| 26918 | -2.05 |  |  |  |  |  |  |
| 207920 | -2.05 |  |  |  |  |  |  |
| 232941 | -2.05 |  |  |  |  |  |  |
| 16193 | -2.05 |  |  |  |  |  |  |
| 76572 | -2.06 |  |  |  |  |  |  |
| 208080 | -2.06 |  |  |  |  |  |  |
| 214240 | -2.06 |  |  |  |  |  |  |
| 625167 | -2.06 |  |  |  |  |  |  |
| 72393 | -2.06 |  |  |  |  |  |  |
| 230828 | -2.07 |  |  |  |  |  |  |
| 224044 | -2.07 |  |  |  |  |  |  |
| 13164 | -2.07 |  |  |  |  |  |  |
| 18191 | -2.08 |  |  |  |  |  |  |
| 11484 | -2.08 |  |  |  |  |  |  |
| 381546 | -2.08 |  |  |  |  |  |  |
| 11668 | -2.08 |  |  |  |  |  |  |
| 227696 | -2.08 |  |  |  |  |  |  |
| 209773 | -2.09 |  |  |  |  |  |  |
| 14281 | -2.09 |  |  |  |  |  |  |
| 330483 | -2.09 |  |  |  |  |  |  |
| 235636 | -2.10 |  |  |  |  |  |  |
| 435626 | -2.10 |  |  |  |  |  |  |
| 116838 | -2.10 |  |  |  |  |  |  |
| 94094 | -2.11 |  |  |  |  |  |  |
| 12841 | -2.11 |  |  |  |  |  |  |
| 66857 | -2.11 |  |  |  |  |  |  |
| 68526 | -2.11 |  |  |  |  |  |  |
| 667742 | -2.11 |  |  |  |  |  |  |
| 97122 | -2.11 |  |  |  |  |  |  |
| 80334 | -2.12 |  |  |  |  |  |  |
| 12661 | -2.12 |  |  |  |  |  |  |
| 69787 | -2.12 |  |  |  |  |  |  |
| 320360 | -2.12 |  |  |  |  |  |  |
| 20210 | -2.12 |  |  |  |  |  |  |
| 210530 | -2.12 |  |  |  |  |  |  |
| 74409 | -2.12 |  |  |  |  |  |  |
| novel_G000944 | -2.12 |  |  |  |  |  |  |
| 327978 | -2.13 |  |  |  |  |  |  |
| 67475 | -2.13 |  |  |  |  |  |  |
| 29809 | -2.13 |  |  |  |  |  |  |
| 228858 | -2.14 |  |  |  |  |  |  |
| novel_G000188 | -2.15 |  |  |  |  |  |  |
| 110637 | -2.15 |  |  |  |  |  |  |
| 12361 | -2.15 |  |  |  |  |  |  |
| 240667 | -2.15 |  |  |  |  |  |  |
| 242608 | -2.15 |  |  |  |  |  |  |
| 16780 | -2.16 |  |  |  |  |  |  |
| 71390 | -2.16 |  |  |  |  |  |  |
| 630146 | -2.16 |  |  |  |  |  |  |
| 67896 | -2.16 |  |  |  |  |  |  |
| 12258 | -2.16 |  |  |  |  |  |  |
| novel_G000193 | -2.16 |  |  |  |  |  |  |
| 21682 | -2.16 |  |  |  |  |  |  |
| 330305 | -2.16 |  |  |  |  |  |  |
| 73895 | -2.17 |  |  |  |  |  |  |
| 50722 | -2.17 |  |  |  |  |  |  |
| 16970 | -2.17 |  |  |  |  |  |  |
| novel_G000774 | -2.17 |  |  |  |  |  |  |
| 235604 | -2.17 |  |  |  |  |  |  |
| 11302 | -2.17 |  |  |  |  |  |  |
| 244646 | -2.17 |  |  |  |  |  |  |
| 19260 | -2.17 |  |  |  |  |  |  |
| 381113 | -2.17 |  |  |  |  |  |  |
| 215632 | -2.18 |  |  |  |  |  |  |
| 20741 | -2.18 |  |  |  |  |  |  |
| 170677 | -2.19 |  |  |  |  |  |  |
| 105242399 | -2.19 |  |  |  |  |  |  |
| 246049 | -2.19 |  |  |  |  |  |  |
| 381538 | -2.19 |  |  |  |  |  |  |
| 240752 | -2.19 |  |  |  |  |  |  |
| 12877 | -2.19 |  |  |  |  |  |  |
| 16145 | -2.20 |  |  |  |  |  |  |
| 17312 | -2.20 |  |  |  |  |  |  |
| 14758 | -2.20 |  |  |  |  |  |  |
| 12291 | -2.21 |  |  |  |  |  |  |
| 16581 | -2.21 |  |  |  |  |  |  |
| 100502829 | -2.21 |  |  |  |  |  |  |
| 15370 | -2.22 |  |  |  |  |  |  |
| 21417 | -2.22 |  |  |  |  |  |  |
| 74419 | -2.23 |  |  |  |  |  |  |
| 14411 | -2.23 |  |  |  |  |  |  |
| 433944 | -2.23 |  |  |  |  |  |  |
| 50934 | -2.23 |  |  |  |  |  |  |
| 545156 | -2.23 |  |  |  |  |  |  |
| novel_G000874 | -2.24 |  |  |  |  |  |  |
| 68396 | -2.24 |  |  |  |  |  |  |
| 20537 | -2.25 |  |  |  |  |  |  |
| 11910 | -2.25 |  |  |  |  |  |  |
| 353235 | -2.25 |  |  |  |  |  |  |
| 52389 | -2.25 |  |  |  |  |  |  |
| 229706 | -2.25 |  |  |  |  |  |  |
| 330409 | -2.25 |  |  |  |  |  |  |
| 53973 | -2.25 |  |  |  |  |  |  |
| 12182 | -2.26 |  |  |  |  |  |  |
| novel_G000412 | -2.26 |  |  |  |  |  |  |
| 21940 | -2.26 |  |  |  |  |  |  |
| 94176 | -2.26 |  |  |  |  |  |  |
| 74229 | -2.26 |  |  |  |  |  |  |
| 245126 | -2.26 |  |  |  |  |  |  |
| 12265 | -2.26 |  |  |  |  |  |  |
| 100043188 | -2.27 |  |  |  |  |  |  |
| 71345 | -2.27 |  |  |  |  |  |  |
| 20715 | -2.27 |  |  |  |  |  |  |
| 271375 | -2.27 |  |  |  |  |  |  |
| 17395 | -2.27 |  |  |  |  |  |  |
| 268902 | -2.28 |  |  |  |  |  |  |
| 20511 | -2.28 |  |  |  |  |  |  |
| 20303 | -2.28 |  |  |  |  |  |  |
| 108096 | -2.28 |  |  |  |  |  |  |
| 12038 | -2.28 |  |  |  |  |  |  |
| 15953 | -2.28 |  |  |  |  |  |  |
| 12268 | -2.28 |  |  |  |  |  |  |
| 16426 | -2.29 |  |  |  |  |  |  |
| 636741 | -2.29 |  |  |  |  |  |  |
| 13982 | -2.29 |  |  |  |  |  |  |
| 224997 | -2.29 |  |  |  |  |  |  |
| 269346 | -2.30 |  |  |  |  |  |  |
| 381411 | -2.30 |  |  |  |  |  |  |
| 20215 | -2.30 |  |  |  |  |  |  |
| 100503468 | -2.30 |  |  |  |  |  |  |
| 625530 | -2.30 |  |  |  |  |  |  |
| 22355 | -2.30 |  |  |  |  |  |  |
| 67547 | -2.31 |  |  |  |  |  |  |
| 17883 | -2.31 |  |  |  |  |  |  |
| novel_G000290 | -2.31 |  |  |  |  |  |  |
| 216049 | -2.31 |  |  |  |  |  |  |
| 14051 | -2.32 |  |  |  |  |  |  |
| 233079 | -2.33 |  |  |  |  |  |  |
| novel_G000139 | -2.33 |  |  |  |  |  |  |
| 320664 | -2.33 |  |  |  |  |  |  |
| 232984 | -2.33 |  |  |  |  |  |  |
| 320975 | -2.33 |  |  |  |  |  |  |
| 54139 | -2.33 |  |  |  |  |  |  |
| 67317 | -2.33 |  |  |  |  |  |  |
| 622283 | -2.33 |  |  |  |  |  |  |
| 67119 | -2.34 |  |  |  |  |  |  |
| 50498 | -2.34 |  |  |  |  |  |  |
| 69635 | -2.34 |  |  |  |  |  |  |
| 11838 | -2.35 |  |  |  |  |  |  |
| 78803 | -2.35 |  |  |  |  |  |  |
| 104086 | -2.35 |  |  |  |  |  |  |
| 272009 | -2.35 |  |  |  |  |  |  |
| 239827 | -2.36 |  |  |  |  |  |  |
| 767815 | -2.36 |  |  |  |  |  |  |
| 14725 | -2.37 |  |  |  |  |  |  |
| novel_G000885 | -2.38 |  |  |  |  |  |  |
| 75858 | -2.38 |  |  |  |  |  |  |
| 16819 | -2.39 |  |  |  |  |  |  |
| 277154 | -2.39 |  |  |  |  |  |  |
| 223648 | -2.39 |  |  |  |  |  |  |
| 224079 | -2.40 |  |  |  |  |  |  |
| 19699 | -2.40 |  |  |  |  |  |  |
| 71912 | -2.41 |  |  |  |  |  |  |
| 100504285 | -2.42 |  |  |  |  |  |  |
| 140709 | -2.42 |  |  |  |  |  |  |
| 327958 | -2.42 |  |  |  |  |  |  |
| 331004 | -2.43 |  |  |  |  |  |  |
| 12483 | -2.43 |  |  |  |  |  |  |
| 16164 | -2.43 |  |  |  |  |  |  |
| 243881 | -2.44 |  |  |  |  |  |  |
| 73707 | -2.44 |  |  |  |  |  |  |
| 17203 | -2.44 |  |  |  |  |  |  |
| 15360 | -2.44 |  |  |  |  |  |  |
| novel_G000031 | -2.45 |  |  |  |  |  |  |
| 66696 | -2.45 |  |  |  |  |  |  |
| 396184 | -2.45 |  |  |  |  |  |  |
| 74559 | -2.46 |  |  |  |  |  |  |
| 21897 | -2.46 |  |  |  |  |  |  |
| 74116 | -2.46 |  |  |  |  |  |  |
| 271424 | -2.46 |  |  |  |  |  |  |
| 58229 | -2.47 |  |  |  |  |  |  |
| 70747 | -2.49 |  |  |  |  |  |  |
| 16181 | -2.49 |  |  |  |  |  |  |
| 56375 | -2.49 |  |  |  |  |  |  |
| 209387 | -2.50 |  |  |  |  |  |  |
| 69066 | -2.50 |  |  |  |  |  |  |
| 74002 | -2.51 |  |  |  |  |  |  |
| 18705 | -2.51 |  |  |  |  |  |  |
| 20265 | -2.51 |  |  |  |  |  |  |
| 229900 | -2.51 |  |  |  |  |  |  |
| 12515 | -2.52 |  |  |  |  |  |  |
| 100417829 | -2.52 |  |  |  |  |  |  |
| 81879 | -2.52 |  |  |  |  |  |  |
| 11979 | -2.52 |  |  |  |  |  |  |
| 12608 | -2.52 |  |  |  |  |  |  |
| 54199 | -2.52 |  |  |  |  |  |  |
| 18616 | -2.53 |  |  |  |  |  |  |
| 107227 | -2.53 |  |  |  |  |  |  |
| 100034251 | -2.53 |  |  |  |  |  |  |
| 15531 | -2.53 |  |  |  |  |  |  |
| 12994 | -2.56 |  |  |  |  |  |  |
| novel_G000332 | -2.56 |  |  |  |  |  |  |
| 668357 | -2.57 |  |  |  |  |  |  |
| 105859 | -2.58 |  |  |  |  |  |  |
| 207596 | -2.58 |  |  |  |  |  |  |
| 100039467 | -2.59 |  |  |  |  |  |  |
| 233529 | -2.60 |  |  |  |  |  |  |
| 83672 | -2.60 |  |  |  |  |  |  |
| 104759 | -2.61 |  |  |  |  |  |  |
| 208943 | -2.61 |  |  |  |  |  |  |
| 13505 | -2.61 |  |  |  |  |  |  |
| 20562 | -2.61 |  |  |  |  |  |  |
| 16011 | -2.62 |  |  |  |  |  |  |
| 269854 | -2.62 |  |  |  |  |  |  |
| 17329 | -2.63 |  |  |  |  |  |  |
| 58176 | -2.65 |  |  |  |  |  |  |
| 20476 | -2.65 |  |  |  |  |  |  |
| 27404 | -2.66 |  |  |  |  |  |  |
| 268527 | -2.67 |  |  |  |  |  |  |
| 21844 | -2.67 |  |  |  |  |  |  |
| 108154 | -2.68 |  |  |  |  |  |  |
| 23844 | -2.68 |  |  |  |  |  |  |
| 381686 | -2.69 |  |  |  |  |  |  |
| 192163 | -2.69 |  |  |  |  |  |  |
| 14825 | -2.70 |  |  |  |  |  |  |
| 19337 | -2.70 |  |  |  |  |  |  |
| 14102 | -2.70 |  |  |  |  |  |  |
| 21452 | -2.70 |  |  |  |  |  |  |
| 14969 | -2.70 |  |  |  |  |  |  |
| 16574 | -2.71 |  |  |  |  |  |  |
| 16531 | -2.72 |  |  |  |  |  |  |
| novel_G000580 | -2.73 |  |  |  |  |  |  |
| 71740 | -2.74 |  |  |  |  |  |  |
| 11682 | -2.74 |  |  |  |  |  |  |
| 16790 | -2.75 |  |  |  |  |  |  |
| 52882 | -2.76 |  |  |  |  |  |  |
| 236573 | -2.76 |  |  |  |  |  |  |
| 108672 | -2.77 |  |  |  |  |  |  |
| 22361 | -2.78 |  |  |  |  |  |  |
| 243634 | -2.78 |  |  |  |  |  |  |
| 58218 | -2.78 |  |  |  |  |  |  |
| 12349 | -2.78 |  |  |  |  |  |  |
| 20344 | -2.78 |  |  |  |  |  |  |
| novel_G000561 | -2.80 |  |  |  |  |  |  |
| 67593 | -2.81 |  |  |  |  |  |  |
| 11997 | -2.81 |  |  |  |  |  |  |
| 76507 | -2.82 |  |  |  |  |  |  |
| 20449 | -2.82 |  |  |  |  |  |  |
| 102371 | -2.82 |  |  |  |  |  |  |
| 269120 | -2.84 |  |  |  |  |  |  |
| 11642 | -2.87 |  |  |  |  |  |  |
| 21952 | -2.87 |  |  |  |  |  |  |
| 76998 | -2.87 |  |  |  |  |  |  |
| 233801 | -2.91 |  |  |  |  |  |  |
| 329152 | -2.91 |  |  |  |  |  |  |
| 319317 | -2.92 |  |  |  |  |  |  |
| novel_G000058 | -2.92 |  |  |  |  |  |  |
| 109979 | -2.93 |  |  |  |  |  |  |
| 192113 | -2.93 |  |  |  |  |  |  |
| 544963 | -2.94 |  |  |  |  |  |  |
| 235180 | -2.94 |  |  |  |  |  |  |
| 53416 | -2.95 |  |  |  |  |  |  |
| 269959 | -2.96 |  |  |  |  |  |  |
| 107751 | -2.96 |  |  |  |  |  |  |
| 14585 | -2.97 |  |  |  |  |  |  |
| 27052 | -2.97 |  |  |  |  |  |  |
| 17472 | -2.98 |  |  |  |  |  |  |
| 109032 | -3.00 |  |  |  |  |  |  |
| 76074 | -3.01 |  |  |  |  |  |  |
| 11447 | -3.02 |  |  |  |  |  |  |
| 14985 | -3.03 |  |  |  |  |  |  |
| 116903 | -3.05 |  |  |  |  |  |  |
| 60345 | -3.05 |  |  |  |  |  |  |
| 21683 | -3.05 |  |  |  |  |  |  |
| 16425 | -3.05 |  |  |  |  |  |  |
| 19739 | -3.06 |  |  |  |  |  |  |
| 78771 | -3.06 |  |  |  |  |  |  |
| 319713 | -3.06 |  |  |  |  |  |  |
| 16160 | -3.07 |  |  |  |  |  |  |
| 103406 | -3.07 |  |  |  |  |  |  |
| 76718 | -3.08 |  |  |  |  |  |  |
| 56838 | -3.10 |  |  |  |  |  |  |
| 64385 | -3.11 |  |  |  |  |  |  |
| 70762 | -3.11 |  |  |  |  |  |  |
| 68774 | -3.12 |  |  |  |  |  |  |
| 56533 | -3.13 |  |  |  |  |  |  |
| 101320 | -3.14 |  |  |  |  |  |  |
| 224796 | -3.16 |  |  |  |  |  |  |
| 234515 | -3.17 |  |  |  |  |  |  |
| 258513 | -3.17 |  |  |  |  |  |  |
| 75801 | -3.18 |  |  |  |  |  |  |
| 14164 | -3.20 |  |  |  |  |  |  |
| 13120 | -3.20 |  |  |  |  |  |  |
| 246190 | -3.21 |  |  |  |  |  |  |
| 20355 | -3.21 |  |  |  |  |  |  |
| 224344 | -3.22 |  |  |  |  |  |  |
| 110696 | -3.23 |  |  |  |  |  |  |
| 330122 | -3.23 |  |  |  |  |  |  |
| 74469 | -3.23 |  |  |  |  |  |  |
| 668940 | -3.23 |  |  |  |  |  |  |
| 68800 | -3.26 |  |  |  |  |  |  |
| 67216 | -3.27 |  |  |  |  |  |  |
| 227120 | -3.28 |  |  |  |  |  |  |
| 100042856 | -3.32 |  |  |  |  |  |  |
| 114564 | -3.32 |  |  |  |  |  |  |
| 654796 | -3.32 |  |  |  |  |  |  |
| 71724 | -3.38 |  |  |  |  |  |  |
| 16149 | -3.38 |  |  |  |  |  |  |
| 12007 | -3.41 |  |  |  |  |  |  |
| 26381 | -3.42 |  |  |  |  |  |  |
| novel_G000834 | -3.42 |  |  |  |  |  |  |
| 18671 | -3.43 |  |  |  |  |  |  |
| 20311 | -3.44 |  |  |  |  |  |  |
| 71884 | -3.44 |  |  |  |  |  |  |
| 213002 | -3.45 |  |  |  |  |  |  |
| novel_G000835 | -3.46 |  |  |  |  |  |  |
| 20704 | -3.48 |  |  |  |  |  |  |
| novel_G000890 | -3.49 |  |  |  |  |  |  |
| novel_G000833 | -3.51 |  |  |  |  |  |  |
| 18022 | -3.53 |  |  |  |  |  |  |
| 93837 | -3.56 |  |  |  |  |  |  |
| 16516 | -3.57 |  |  |  |  |  |  |
| 244698 | -3.58 |  |  |  |  |  |  |
| 224761 | -3.59 |  |  |  |  |  |  |
| 53603 | -3.59 |  |  |  |  |  |  |
| 12818 | -3.60 |  |  |  |  |  |  |
| 239790 | -3.60 |  |  |  |  |  |  |
| 17386 | -3.64 |  |  |  |  |  |  |
| 59083 | -3.64 |  |  |  |  |  |  |
| 620913 | -3.66 |  |  |  |  |  |  |
| 215243 | -3.68 |  |  |  |  |  |  |
| 18703 | -3.69 |  |  |  |  |  |  |
| novel_G000518 | -3.70 |  |  |  |  |  |  |
| 241528 | -3.73 |  |  |  |  |  |  |
| 20310 | -3.73 |  |  |  |  |  |  |
| 217169 | -3.73 |  |  |  |  |  |  |
| 64454 | -3.74 |  |  |  |  |  |  |
| 236312 | -3.75 |  |  |  |  |  |  |
| novel_G000138 | -3.76 |  |  |  |  |  |  |
| 67928 | -3.78 |  |  |  |  |  |  |
| novel_G000025 | -3.86 |  |  |  |  |  |  |
| 12231 | -3.87 |  |  |  |  |  |  |
| 20302 | -3.98 |  |  |  |  |  |  |
| 20297 | -4.01 |  |  |  |  |  |  |
| 73998 | -4.02 |  |  |  |  |  |  |
| 434223 | -4.03 |  |  |  |  |  |  |
| 74020 | -4.06 |  |  |  |  |  |  |
| novel_G000230 | -4.06 |  |  |  |  |  |  |
| 100322896 | -4.11 |  |  |  |  |  |  |
| 71738 | -4.14 |  |  |  |  |  |  |
| 117167 | -4.22 |  |  |  |  |  |  |
| 192190 | -4.27 |  |  |  |  |  |  |
| 103964 | -4.35 |  |  |  |  |  |  |
| 54563 | -4.36 |  |  |  |  |  |  |
| 208869 | -4.39 |  |  |  |  |  |  |
| 637004 | -4.40 |  |  |  |  |  |  |
| 67606 | -4.41 |  |  |  |  |  |  |
| 109676 | -4.46 |  |  |  |  |  |  |
| novel_G000515 | -4.48 |  |  |  |  |  |  |
| 93732 | -4.59 |  |  |  |  |  |  |
| 545370 | -4.64 |  |  |  |  |  |  |
| 23964 | -4.66 |  |  |  |  |  |  |
| 16428 | -4.79 |  |  |  |  |  |  |
| 19281 | -4.86 |  |  |  |  |  |  |
| 243369 | -5.07 |  |  |  |  |  |  |
| 26464 | -5.16 |  |  |  |  |  |  |
| 18419 | -5.50 |  |  |  |  |  |  |
| novel_G000519 | -5.98 |  |  |  |  |  |  |

**Supplementary Table S2:** Differentially expressed genes in L929 cells treated with 200μM Ni^2+^ for different durations.

| **200μM-12h** | | **200μM-24h** | | **200μM-48h** | | **200μM-72h** | |
| --- | --- | --- | --- | --- | --- | --- | --- |
| Entrez Gene ID | log_2_FC | Entrez Gene ID | log_2_FC | Entrez Gene ID | log_2_FC | Entrez Gene ID | log_2_FC |
| novel_G000020 | 6.49 | novel_G000829 | 5.83 | 237940 | 4.92 | novel_G000829 | 5.63 |
| novel_G000829 | 5.50 | novel_G000020 | 4.92 | novel_G000829 | 4.91 | 237940 | 5.02 |
| novel_G000500 | 4.49 | novel_G000500 | 4.32 | 78558 | 4.35 | 78558 | 4.57 |
| 170574 | 3.85 | 237940 | 4.29 | novel_G000247 | 4.04 | novel_G000247 | 4.54 |
| 237940 | 3.69 | 78558 | 4.06 | 14200 | 3.78 | novel_G000500 | 4.40 |
| 78558 | 3.62 | 14857 | 3.59 | 1E+08 | 3.75 | novel_G000020 | 4.24 |
| 81840 | 3.30 | 12865 | 3.51 | 70045 | 3.62 | 14200 | 4.20 |
| 1.06E+08 | 2.86 | 74185 | 3.40 | 15267 | 3.60 | 22403 | 4.08 |
| 14857 | 2.70 | novel_G000092 | 3.29 | novel_G000092 | 3.38 | 20707 | 3.97 |
| 1E+08 | 2.68 | 56295 | 3.14 | novel_G000020 | 3.35 | 18787 | 3.89 |
| 381284 | 2.65 | 215627 | 3.10 | 18426 | 3.34 | 244859 | 3.88 |
| 16835 | 2.59 | novel_G000097 | 2.97 | 12865 | 3.28 | 15267 | 3.77 |
| 18378 | 2.58 | novel_G000395 | 2.91 | 244859 | 3.27 | 70045 | 3.74 |
| 215627 | 2.57 | 76873 | 2.88 | 70186 | 3.26 | 20753 | 3.71 |
| 232889 | 2.56 | 80879 | 2.87 | 22403 | 3.25 | 18426 | 3.67 |
| 112407 | 2.51 | 70153 | 2.86 | 94180 | 3.22 | novel_G000640 | 3.59 |
| novel_G000839 | 2.45 | 94180 | 2.86 | 20289 | 3.13 | 319189 | 3.59 |
| 94180 | 2.43 | 70045 | 2.79 | 56295 | 3.11 | novel_G000092 | 3.59 |
| 56295 | 2.33 | 64095 | 2.75 | 20710 | 3.03 | 77059 | 3.50 |
| 654824 | 2.30 | 654824 | 2.69 | 74185 | 3.03 | 20710 | 3.47 |
| 64095 | 2.29 | novel_G000265 | 2.68 | 18787 | 3.01 | 66425 | 3.46 |
| novel_G000043 | 2.28 | 18426 | 2.68 | 235416 | 2.98 | 94180 | 3.44 |
| 80879 | 2.28 | novel_G000247 | 2.67 | 14857 | 2.98 | 16000 | 3.40 |
| 272158 | 2.28 | novel_G000593 | 2.67 | 667728 | 2.97 | novel_G000246 | 3.37 |
| 50994 | 2.27 | novel_G000545 | 2.67 | 77059 | 2.92 | 667728 | 3.33 |
| 20250 | 2.27 | 70186 | 2.57 | novel_G000246 | 2.89 | 22283 | 3.24 |
| 665433 | 2.26 | 70713 | 2.57 | 66425 | 2.87 | 545936 | 3.18 |
| 74185 | 2.25 | novel_G000604 | 2.57 | 319189 | 2.87 | 74185 | 3.11 |
| novel_G000657 | 2.20 | novel_G000795 | 2.55 | novel_G000795 | 2.77 | 12865 | 3.07 |
| 74747 | 2.18 | novel_G000342 | 2.53 | 20709 | 2.76 | 20289 | 3.05 |
| 11535 | 2.14 | 216859 | 2.52 | 76873 | 2.74 | 213393 | 3.02 |
| 626275 | 2.12 | 170776 | 2.46 | 22283 | 2.73 | 70186 | 3.00 |
| novel_G000197 | 2.07 | 235416 | 2.46 | novel_G000582 | 2.71 | 213409 | 2.99 |
| novel_G000092 | 2.05 | NM_001349066 | 2.43 | 12176 | 2.68 | 235416 | 2.97 |
| 20755 | 2.05 | novel_G000093 | 2.42 | 64095 | 2.67 | 94352 | 2.93 |
| 1E+08 | 2.05 | 1.01E+08 | 2.42 | 213171 | 2.65 | 213171 | 2.90 |
| novel_G000545 | 2.05 | 12176 | 2.39 | 17988 | 2.65 | novel_G000795 | 2.90 |
| 631323 | 2.03 | 20707 | 2.37 | novel_G000395 | 2.64 | 56295 | 2.88 |
| 242642 | 2.02 | 112405 | 2.37 | 1.01E+08 | 2.63 | 76873 | 2.87 |
| 170776 | 1.97 | 1.01E+08 | 2.34 | 20753 | 2.60 | 433456 | 2.86 |
| novel_G000097 | 1.96 | 242642 | 2.34 | 269275 | 2.56 | 14311 | 2.85 |
| 360198 | 1.94 | 545936 | 2.34 | 14166 | 2.55 | 18947 | 2.83 |
| novel_G000272 | 1.93 | 17988 | 2.32 | 243083 | 2.55 | novel_G000216 | 2.82 |
| novel_G000636 | 1.93 | 20342 | 2.31 | novel_G000097 | 2.54 | 20342 | 2.82 |
| 18787 | 1.91 | 269275 | 2.30 | 94352 | 2.54 | 15586 | 2.82 |
| 20135 | 1.91 | 16854 | 2.29 | 407790 | 2.52 | 17988 | 2.81 |
| 546049 | 1.90 | 67855 | 2.29 | 20342 | 2.52 | 11535 | 2.76 |
| 228026 | 1.86 | 13089 | 2.29 | 68891 | 2.51 | 20709 | 2.73 |
| 319166 | 1.86 | 11535 | 2.28 | 80879 | 2.49 | 81840 | 2.72 |
| novel_G000152 | 1.85 | 407790 | 2.26 | 15586 | 2.48 | 20250 | 2.70 |
| 12865 | 1.85 | 112407 | 2.25 | 70713 | 2.47 | 70713 | 2.65 |
| 77056 | 1.84 | 665180 | 2.24 | 70153 | 2.47 | 17533 | 2.64 |
| novel_G000308 | 1.84 | 243083 | 2.24 | 112405 | 2.45 | 14559 | 2.64 |
| novel_G000093 | 1.83 | 228026 | 2.24 | novel_G000093 | 2.45 | 407790 | 2.64 |
| 76873 | 1.83 | 230863 | 2.23 | 545936 | 2.45 | 102614 | 2.63 |
| 20525 | 1.83 | 229665 | 2.22 | 11535 | 2.45 | 269423 | 2.62 |
| 545652 | 1.82 | 230099 | 2.21 | 20707 | 2.44 | 78668 | 2.62 |
| 433456 | 1.82 | 66425 | 2.21 | 433456 | 2.43 | 216859 | 2.60 |
| 70835 | 1.82 | novel_G000152 | 2.20 | 329738 | 2.35 | 64095 | 2.56 |
| 22042 | 1.81 | novel_G000246 | 2.19 | 1.01E+08 | 2.34 | 12176 | 2.55 |
| 21647 | 1.81 | 70162 | 2.18 | 216859 | 2.32 | novel_G000593 | 2.55 |
| 328949 | 1.80 | 15586 | 2.18 | 230099 | 2.31 | 320046 | 2.55 |
| 667766 | 1.80 | 74747 | 2.17 | novel_G000342 | 2.31 | 332942 | 2.55 |
| 20198 | 1.79 | 20525 | 2.17 | novel_G000593 | 2.30 | 269275 | 2.54 |
| 78754 | 1.78 | 18655 | 2.17 | 17930 | 2.29 | novel_G000093 | 2.53 |
| 18641 | 1.78 | 72157 | 2.16 | 654824 | 2.29 | novel_G000342 | 2.52 |
| NM_001348198 | 1.78 | 73720 | 2.15 | 213393 | 2.29 | 12816 | 2.52 |
| 1E+08 | 1.77 | 20250 | 2.15 | 72157 | 2.27 | 68891 | 2.48 |
| 99543 | 1.77 | 72240 | 2.15 | 16000 | 2.26 | novel_G000214 | 2.41 |
| 74246 | 1.77 | 381822 | 2.14 | 81840 | 2.26 | 74747 | 2.40 |
| 19652 | 1.75 | novel_G000308 | 2.13 | 69665 | 2.26 | 23928 | 2.39 |
| 16828 | 1.74 | 11639 | 2.12 | 57444 | 2.24 | 12000 | 2.39 |
| 107753 | 1.74 | 14166 | 2.11 | 20250 | 2.24 | 230099 | 2.37 |
| novel_G000645 | 1.74 | 14714 | 2.11 | 102614 | 2.24 | 112405 | 2.36 |
| novel_G000920 | 1.74 | 76573 | 2.09 | 74747 | 2.23 | 243083 | 2.36 |
| 235416 | 1.74 | 17533 | 2.08 | 17533 | 2.21 | novel_G000952 | 2.36 |
| 16854 | 1.74 | 13885 | 2.06 | 23863 | 2.21 | 57435 | 2.35 |
| 20893 | 1.74 | 319166 | 2.06 | 16854 | 2.20 | 654824 | 2.34 |
| 77940 | 1.71 | 94352 | 2.06 | 20341 | 2.19 | 13175 | 2.34 |
| 1E+08 | 1.71 | novel_G000010 | 2.06 | 75647 | 2.18 | 16669 | 2.33 |
| 14433 | 1.71 | 20341 | 2.05 | 11639 | 2.17 | 112407 | 2.33 |
| 73673 | 1.70 | 14433 | 2.04 | 23928 | 2.17 | 74341 | 2.33 |
| 12843 | 1.69 | 20408 | 2.04 | 18655 | 2.16 | 319154 | 2.32 |
| 72157 | 1.68 | novel_G000481 | 2.03 | 328190 | 2.14 | novel_G000010 | 2.31 |
| 16592 | 1.67 | novel_G000645 | 2.03 | novel_G000696 | 2.14 | 109697 | 2.31 |
| 73649 | 1.67 | novel_G000696 | 2.02 | 80885 | 2.14 | 381284 | 2.30 |
| 112405 | 1.67 | 320700 | 2.02 | novel_G000216 | 2.14 | 16835 | 2.29 |
| 15331 | 1.66 | 269423 | 2.01 | 381284 | 2.14 | 626275 | 2.27 |
| NM_001348199 | 1.66 | 20710 | 2.00 | 12778 | 2.13 | novel_G000480 | 2.26 |
| 69573 | 1.65 | 69573 | 1.99 | 56441 | 2.13 | 97122 | 2.25 |
| 18655 | 1.65 | 12452 | 1.99 | 20525 | 2.13 | 13081 | 2.25 |
| 433182 | 1.65 | 17750 | 1.99 | 56485 | 2.13 | 20525 | 2.25 |
| 12306 | 1.65 | 381284 | 1.99 | 112407 | 2.12 | 13041 | 2.23 |
| 73720 | 1.64 | 11676 | 1.98 | 14311 | 2.09 | 20341 | 2.22 |
| 11639 | 1.64 | 56441 | 1.98 | 18947 | 2.09 | 14166 | 2.21 |
| 94352 | 1.64 | 230098 | 1.98 | 76378 | 2.07 | 80879 | 2.21 |
| novel_G000312 | 1.64 | novel_G000658 | 1.97 | 239691 | 2.06 | 72157 | 2.21 |
| 70713 | 1.64 | 20289 | 1.97 | novel_G000545 | 2.06 | 57444 | 2.19 |
| 14936 | 1.63 | 14200 | 1.96 | 12816 | 2.06 | 56485 | 2.19 |
| 12176 | 1.63 | 69772 | 1.93 | 13885 | 2.05 | 14433 | 2.18 |
| 207728 | 1.63 | 545652 | 1.93 | 228026 | 2.04 | 1E+08 | 2.17 |
| 16906 | 1.61 | 57444 | 1.93 | 20408 | 2.03 | 208164 | 2.16 |
| 76220 | 1.60 | 667766 | 1.93 | 13081 | 2.03 | 118449 | 2.16 |
| 213393 | 1.60 | 14936 | 1.92 | 12452 | 2.02 | 14601 | 2.16 |
| 110196 | 1.60 | 102614 | 1.92 | NR_145504 | 2.00 | 20755 | 2.15 |
| 1.01E+08 | 1.60 | 16835 | 1.91 | 76072 | 2.00 | 71297 | 2.14 |
| 212439 | 1.60 | 407800 | 1.91 | 269423 | 2.00 | novel_G000482 | 2.14 |
| 17218 | 1.59 | 69788 | 1.91 | 20491 | 1.99 | 53322 | 2.14 |
| 19348 | 1.58 | 17748 | 1.91 | novel_G000636 | 1.98 | 12778 | 2.14 |
| 16987 | 1.58 | 214425 | 1.90 | 19011 | 1.98 | 493583 | 2.14 |
| 18140 | 1.58 | 77940 | 1.90 | 11676 | 1.97 | 545652 | 2.13 |
| 107995 | 1.58 | 1E+08 | 1.90 | 320046 | 1.97 | 68024 | 2.13 |
| novel_G000795 | 1.57 | novel_G000362 | 1.90 | 77940 | 1.97 | 193280 | 2.12 |
| 69325 | 1.56 | 67669 | 1.90 | 229665 | 1.96 | 75647 | 2.11 |
| 30878 | 1.56 | 17436 | 1.90 | novel_G000090 | 1.96 | 217837 | 2.10 |
| 66234 | 1.56 | 77056 | 1.89 | 13175 | 1.95 | 72373 | 2.10 |
| 319190 | 1.55 | 16828 | 1.89 | novel_G000214 | 1.94 | novel_G000095 | 2.09 |
| 15277 | 1.55 | 20753 | 1.89 | 74341 | 1.94 | 11639 | 2.08 |
| 1E+08 | 1.55 | 239691 | 1.88 | 69675 | 1.93 | 1.01E+08 | 2.08 |
| 68603 | 1.55 | 18787 | 1.87 | 20733 | 1.92 | 1E+08 | 2.08 |
| 216148 | 1.55 | 20709 | 1.86 | 74782 | 1.92 | novel_G000097 | 2.08 |
| 21991 | 1.54 | 18641 | 1.86 | 16828 | 1.91 | 243958 | 2.08 |
| 110208 | 1.53 | 329738 | 1.86 | 21929 | 1.91 | 328190 | 2.07 |
| 110460 | 1.52 | 72391 | 1.86 | novel_G000480 | 1.91 | 1E+08 | 2.07 |
| 19891 | 1.52 | 70419 | 1.85 | 50527 | 1.91 | 107753 | 2.05 |
| 12316 | 1.51 | 435766 | 1.85 | 17436 | 1.91 | 18405 | 2.03 |
| 208084 | 1.51 | 72326 | 1.85 | 74626 | 1.89 | 18655 | 2.02 |
| 381822 | 1.51 | 319154 | 1.84 | 14325 | 1.89 | novel_G000696 | 2.02 |
| 69918 | 1.50 | 414072 | 1.84 | 66113 | 1.88 | 56441 | 2.01 |
| 242691 | 1.50 | novel_G000144 | 1.84 | 211652 | 1.88 | 12452 | 2.01 |
| 68278 | 1.50 | 73649 | 1.84 | 69908 | 1.88 | 15220 | 2.01 |
| 52530 | 1.50 | 21924 | 1.84 | 69772 | 1.85 | 16854 | 2.00 |
| 12428 | 1.50 | 319181 | 1.83 | 319154 | 1.85 | 16592 | 1.99 |
| 72607 | 1.50 | 433456 | 1.83 | 18641 | 1.84 | 76072 | 1.98 |
| 319707 | 1.49 | novel_G000491 | 1.83 | 433182 | 1.84 | 84004 | 1.97 |
| 329371 | 1.49 | 244859 | 1.82 | 73720 | 1.84 | 21953 | 1.97 |
| 70045 | 1.49 | 1E+08 | 1.82 | 73649 | 1.84 | 19268 | 1.96 |
| 17988 | 1.48 | 1E+08 | 1.81 | 23796 | 1.84 | 214137 | 1.95 |
| 17748 | 1.48 | 76072 | 1.81 | 74121 | 1.84 | 1.01E+08 | 1.95 |
| 19655 | 1.48 | 269630 | 1.81 | NM_001348167 | 1.83 | 228026 | 1.95 |
| 68671 | 1.48 | 434147 | 1.80 | 214137 | 1.82 | 381232 | 1.94 |
| 20342 | 1.48 | 50527 | 1.80 | 17873 | 1.82 | 18641 | 1.94 |
| 17750 | 1.47 | 213393 | 1.80 | 68404 | 1.82 | 70153 | 1.94 |
| 14704 | 1.46 | 20755 | 1.80 | 118452 | 1.79 | 12557 | 1.94 |
| 1E+08 | 1.46 | 1E+08 | 1.80 | NM_001349066 | 1.79 | 631323 | 1.93 |
| 195434 | 1.46 | 433182 | 1.79 | 16835 | 1.79 | 19011 | 1.93 |
| 20709 | 1.46 | 15458 | 1.79 | 11674 | 1.78 | 624845 | 1.93 |
| 66461 | 1.46 | 107995 | 1.77 | novel_G000375 | 1.78 | 11676 | 1.92 |
| novel_G000886 | 1.46 | 70281 | 1.77 | 14601 | 1.77 | novel_G000854 | 1.92 |
| 108000 | 1.45 | 320301 | 1.77 | 84004 | 1.77 | 77940 | 1.92 |
| 654467 | 1.45 | 237847 | 1.77 | 243958 | 1.77 | 80885 | 1.92 |
| 260315 | 1.44 | 16592 | 1.74 | novel_G000095 | 1.77 | 23796 | 1.92 |
| 269275 | 1.44 | 20893 | 1.74 | 217837 | 1.77 | 20893 | 1.91 |
| 208715 | 1.43 | 14325 | 1.74 | novel_G000491 | 1.77 | 21929 | 1.91 |
| 17215 | 1.42 | 105418 | 1.74 | 18648 | 1.76 | 107934 | 1.91 |
| 14283 | 1.42 | 217837 | 1.74 | 1E+08 | 1.76 | 207259 | 1.91 |
| 14793 | 1.42 | 18302 | 1.73 | 332942 | 1.76 | 108078 | 1.90 |
| 18746 | 1.42 | 329371 | 1.73 | 242642 | 1.76 | 50527 | 1.90 |
| 18392 | 1.41 | 81840 | 1.73 | 18452 | 1.75 | 69665 | 1.89 |
| 17345 | 1.41 | 18746 | 1.73 | 108150 | 1.75 | novel_G000350 | 1.89 |
| 545261 | 1.41 | 114676 | 1.72 | 1E+08 | 1.75 | 11717 | 1.89 |
| 74782 | 1.41 | 13807 | 1.72 | 209351 | 1.74 | 22173 | 1.88 |
| 11958 | 1.41 | 60406 | 1.72 | 381404 | 1.74 | 20408 | 1.88 |
| 50708 | 1.41 | 108150 | 1.72 | 1E+08 | 1.74 | 626009 | 1.88 |
| novel_G000463 | 1.41 | 27981 | 1.71 | 216148 | 1.73 | 668415 | 1.88 |
| 240514 | 1.40 | 110196 | 1.71 | 12557 | 1.73 | 17930 | 1.88 |
| 18648 | 1.40 | 1E+08 | 1.70 | 75180 | 1.73 | novel_G000556 | 1.88 |
| 270685 | 1.40 | 21748 | 1.70 | 18405 | 1.73 | novel_G000090 | 1.88 |
| 231070 | 1.40 | 16322 | 1.69 | 57435 | 1.73 | 68404 | 1.87 |
| 14166 | 1.40 | 381045 | 1.69 | 16165 | 1.72 | 214425 | 1.87 |
| 12217 | 1.40 | 320456 | 1.69 | 14936 | 1.72 | 319181 | 1.86 |
| 66447 | 1.40 | novel_G000854 | 1.69 | 381045 | 1.72 | 20186 | 1.86 |
| 57435 | 1.39 | 14860 | 1.68 | 107934 | 1.71 | 14857 | 1.86 |
| 234577 | 1.39 | 384071 | 1.68 | 1E+08 | 1.71 | 20733 | 1.85 |
| 21335 | 1.39 | 328949 | 1.67 | 232714 | 1.71 | 74782 | 1.85 |
| 320700 | 1.39 | 68404 | 1.67 | 70162 | 1.70 | 19116 | 1.85 |
| 104263 | 1.39 | 12615 | 1.67 | 20893 | 1.70 | 330277 | 1.84 |
| 14235 | 1.39 | 17319 | 1.66 | 18746 | 1.69 | 11906 | 1.84 |
| 13807 | 1.38 | 626275 | 1.66 | 72240 | 1.69 | novel_G000381 | 1.84 |
| 104080 | 1.38 | 665433 | 1.66 | 69573 | 1.69 | 66650 | 1.84 |
| 21929 | 1.38 | 72655 | 1.66 | 170745 | 1.68 | 69386 | 1.84 |
| 17865 | 1.38 | 14793 | 1.66 | 56223 | 1.68 | 23945 | 1.83 |
| 269630 | 1.38 | 18648 | 1.66 | 330277 | 1.68 | 328949 | 1.83 |
| novel_G000696 | 1.38 | 18173 | 1.65 | 545261 | 1.67 | novel_G000496 | 1.83 |
| 67486 | 1.37 | 208084 | 1.65 | 76062 | 1.67 | 16828 | 1.83 |
| 16907 | 1.37 | 13175 | 1.64 | 244550 | 1.67 | 70162 | 1.83 |
| 74341 | 1.37 | novel_G000797 | 1.64 | novel_G000854 | 1.66 | 654472 | 1.83 |
| 67194 | 1.36 | 216148 | 1.64 | 242594 | 1.66 | novel_G000485 | 1.83 |
| 20419 | 1.36 | 319798 | 1.63 | novel_G000148 | 1.65 | 17436 | 1.83 |
| 230098 | 1.36 | 17930 | 1.63 | 23945 | 1.65 | 11674 | 1.83 |
| 12452 | 1.35 | 68603 | 1.63 | 236899 | 1.65 | 240332 | 1.82 |
| 381045 | 1.35 | 72080 | 1.63 | 215627 | 1.65 | 407786 | 1.82 |
| novel_G000342 | 1.34 | 330921 | 1.63 | 207259 | 1.64 | 27028 | 1.82 |
| 242705 | 1.34 | 381853 | 1.63 | 246228 | 1.64 | 73598 | 1.82 |
| 72391 | 1.33 | 12217 | 1.63 | 328949 | 1.64 | 215627 | 1.81 |
| 13806 | 1.33 | 19348 | 1.63 | 1.01E+08 | 1.64 | 118452 | 1.81 |
| 216859 | 1.33 | 68612 | 1.63 | 213696 | 1.63 | 68952 | 1.81 |
| 97165 | 1.33 | 21929 | 1.61 | 101544 | 1.63 | 14282 | 1.80 |
| 14115 | 1.33 | 83436 | 1.61 | 83436 | 1.62 | 57257 | 1.80 |
| 60406 | 1.33 | 74782 | 1.61 | novel_G000091 | 1.62 | 211652 | 1.80 |
| 72657 | 1.33 | 665268 | 1.61 | 216377 | 1.62 | 109689 | 1.80 |
| 15368 | 1.32 | 15277 | 1.61 | 381582 | 1.62 | 195359 | 1.80 |
| 268697 | 1.32 | 12306 | 1.60 | 97122 | 1.61 | 18648 | 1.79 |
| 100910 | 1.32 | 56410 | 1.60 | 1E+08 | 1.61 | 69772 | 1.79 |
| 80288 | 1.32 | 236899 | 1.60 | 320181 | 1.60 | 18828 | 1.79 |
| 209737 | 1.31 | 21991 | 1.60 | 118449 | 1.60 | NM_001349066 | 1.79 |
| 11600 | 1.31 | 108101 | 1.60 | 24052 | 1.60 | 14714 | 1.78 |
| 83701 | 1.31 | 545261 | 1.60 | 237847 | 1.60 | 73106 | 1.78 |
| novel_G000646 | 1.31 | 16987 | 1.59 | 319162 | 1.60 | 320700 | 1.77 |
| 18173 | 1.31 | 20733 | 1.58 | 1.01E+08 | 1.59 | 77577 | 1.77 |
| 74931 | 1.30 | 15368 | 1.58 | 73106 | 1.59 | 383766 | 1.77 |
| 66101 | 1.30 | 1.01E+08 | 1.58 | 68841 | 1.58 | 208982 | 1.77 |
| 56075 | 1.30 | 320046 | 1.58 | 383766 | 1.58 | 70417 | 1.77 |
| 12722 | 1.30 | 1.05E+08 | 1.58 | 11906 | 1.57 | 83554 | 1.76 |
| 72341 | 1.29 | 66447 | 1.58 | 56410 | 1.57 | 381287 | 1.76 |
| 237847 | 1.29 | 74341 | 1.58 | 26432 | 1.56 | 384071 | 1.76 |
| 52276 | 1.29 | 18412 | 1.57 | 18302 | 1.56 | 1.01E+08 | 1.76 |
| 68385 | 1.29 | 319190 | 1.57 | 19329 | 1.56 | 11443 | 1.76 |
| 12704 | 1.29 | 11674 | 1.57 | 320700 | 1.55 | 230587 | 1.76 |
| 15586 | 1.28 | 14579 | 1.56 | 66153 | 1.55 | 18452 | 1.76 |
| 13167 | 1.28 | 18563 | 1.56 | 246177 | 1.55 | 230806 | 1.75 |
| 545647 | 1.28 | 56193 | 1.56 | 626275 | 1.55 | 12217 | 1.75 |
| 18538 | 1.28 | 216831 | 1.56 | 27981 | 1.54 | 57385 | 1.74 |
| 68465 | 1.28 | 114230 | 1.56 | 22346 | 1.54 | 381853 | 1.74 |
| 50927 | 1.28 | novel_G000090 | 1.55 | 14714 | 1.53 | 20713 | 1.74 |
| 244550 | 1.28 | 13806 | 1.55 | 12215 | 1.52 | 20491 | 1.73 |
| 78286 | 1.28 | 170745 | 1.54 | 18451 | 1.52 | 20682 | 1.72 |
| 18563 | 1.27 | 383766 | 1.54 | 11717 | 1.52 | 665268 | 1.72 |
| 68298 | 1.27 | 104080 | 1.53 | 104263 | 1.51 | novel_G000897 | 1.72 |
| 110809 | 1.27 | 211652 | 1.53 | 269630 | 1.50 | 97908 | 1.72 |
| 319998 | 1.27 | 17873 | 1.53 | 72607 | 1.50 | 1E+08 | 1.72 |
| 16580 | 1.27 | 319504 | 1.53 | 109689 | 1.50 | 16068 | 1.71 |
| 110033 | 1.27 | 104263 | 1.52 | 213391 | 1.50 | 192897 | 1.71 |
| 229699 | 1.26 | novel_G000375 | 1.52 | 107753 | 1.50 | 242642 | 1.71 |
| 17855 | 1.26 | 18452 | 1.52 | 107817 | 1.49 | 545261 | 1.70 |
| 223650 | 1.26 | 72607 | 1.50 | 14433 | 1.49 | 14115 | 1.70 |
| 75957 | 1.26 | 13853 | 1.50 | 83554 | 1.48 | 70835 | 1.69 |
| 435766 | 1.26 | 56289 | 1.50 | 78600 | 1.48 | 272667 | 1.69 |
| 72080 | 1.25 | 107753 | 1.50 | 13806 | 1.48 | 22249 | 1.69 |
| 20710 | 1.25 | 66153 | 1.49 | 14579 | 1.47 | 170745 | 1.69 |
| 69731 | 1.25 | 216377 | 1.49 | 16592 | 1.47 | 271127 | 1.69 |
| 59001 | 1.25 | novel_G000091 | 1.49 | 665268 | 1.47 | 16782 | 1.69 |
| 1.01E+08 | 1.24 | 19309 | 1.49 | 104252 | 1.46 | 19252 | 1.68 |
| 71406 | 1.24 | 1E+08 | 1.48 | 70281 | 1.46 | 67552 | 1.68 |
| 110956 | 1.24 | 110208 | 1.48 | 68024 | 1.46 | 18746 | 1.67 |
| 67951 | 1.24 | 320842 | 1.48 | 140486 | 1.46 | 14936 | 1.67 |
| 13121 | 1.23 | 74183 | 1.48 | 213948 | 1.45 | 239691 | 1.67 |
| 230908 | 1.23 | novel_G000525 | 1.48 | 18412 | 1.45 | 234683 | 1.66 |
| 56289 | 1.23 | 270685 | 1.48 | 12306 | 1.45 | 320456 | 1.66 |
| 15357 | 1.23 | 1E+08 | 1.48 | 380753 | 1.44 | 108150 | 1.66 |
| 11826 | 1.23 | 192897 | 1.47 | 12217 | 1.43 | 18019 | 1.65 |
| novel_G000326 | 1.23 | 12778 | 1.47 | 71653 | 1.43 | novel_G000491 | 1.65 |
| 78767 | 1.23 | 16580 | 1.47 | 104080 | 1.43 | 320181 | 1.65 |
| 111970 | 1.22 | 74053 | 1.46 | 76438 | 1.42 | 433182 | 1.64 |
| 109212 | 1.22 | 319189 | 1.46 | 230098 | 1.42 | 213948 | 1.64 |
| 67681 | 1.22 | 12532 | 1.46 | 20197 | 1.42 | 56410 | 1.64 |
| 14205 | 1.22 | 1.01E+08 | 1.46 | novel_G000230 | 1.42 | 244550 | 1.64 |
| 114663 | 1.22 | 330277 | 1.46 | 78405 | 1.41 | 269630 | 1.64 |
| 76464 | 1.22 | novel_G000903 | 1.46 | 13853 | 1.41 | 18193 | 1.64 |
| 68612 | 1.22 | 207259 | 1.45 | 13849 | 1.40 | 22788 | 1.64 |
| 1E+08 | 1.22 | 77059 | 1.45 | 11443 | 1.40 | 83436 | 1.64 |
| 74107 | 1.22 | 12816 | 1.45 | 384071 | 1.40 | 277978 | 1.63 |
| 67629 | 1.21 | 74286 | 1.45 | 227526 | 1.39 | 26432 | 1.63 |
| 11906 | 1.21 | 11906 | 1.44 | novel_G000897 | 1.38 | 381045 | 1.63 |
| 12615 | 1.21 | 20725 | 1.44 | 14751 | 1.38 | 78512 | 1.62 |
| 74016 | 1.21 | 107817 | 1.44 | 74076 | 1.38 | 104263 | 1.62 |
| 12557 | 1.21 | 74410 | 1.44 | 216456 | 1.37 | 72607 | 1.62 |
| 268977 | 1.21 | 109672 | 1.44 | 110310 | 1.37 | 69573 | 1.61 |
| 13175 | 1.21 | 101544 | 1.44 | 26388 | 1.37 | 80976 | 1.61 |
| 107528 | 1.21 | 56229 | 1.43 | 14598 | 1.37 | 1.01E+08 | 1.60 |
| 270906 | 1.20 | 22042 | 1.43 | 17319 | 1.37 | 242594 | 1.60 |
| 271278 | 1.20 | 192734 | 1.43 | 66834 | 1.36 | 22339 | 1.59 |
| 237436 | 1.20 | NM_001348199 | 1.43 | 1E+08 | 1.36 | novel_G000148 | 1.59 |
| 69706 | 1.20 | novel_G000433 | 1.43 | 56018 | 1.36 | 74476 | 1.59 |
| 399558 | 1.20 | 227526 | 1.43 | 70417 | 1.35 | 240168 | 1.59 |
| 18194 | 1.20 | 13643 | 1.43 | 14723 | 1.35 | 213043 | 1.58 |
| 223780 | 1.20 | 78575 | 1.42 | 68763 | 1.35 | 380753 | 1.58 |
| 16647 | 1.20 | 268697 | 1.41 | 14428 | 1.35 | 1E+08 | 1.58 |
| 272551 | 1.20 | 232714 | 1.41 | novel_G000525 | 1.35 | 1E+08 | 1.58 |
| 74356 | 1.20 | novel_G000145 | 1.41 | 1.01E+08 | 1.35 | novel_G000976 | 1.58 |
| 109672 | 1.20 | 76438 | 1.41 | 211666 | 1.35 | 101544 | 1.58 |
| 16571 | 1.20 | 68952 | 1.41 | 12904 | 1.35 | 30878 | 1.57 |
| 12532 | 1.19 | 74246 | 1.41 | 20971 | 1.35 | 216377 | 1.57 |
| 22390 | 1.19 | 22346 | 1.41 | novel_G000481 | 1.34 | 380711 | 1.57 |
| 12449 | 1.19 | 71653 | 1.41 | 108767 | 1.34 | 246228 | 1.57 |
| 320301 | 1.19 | 213391 | 1.41 | 66447 | 1.34 | 70281 | 1.57 |
| 67037 | 1.19 | 237436 | 1.41 | 15277 | 1.34 | 107817 | 1.57 |
| 22352 | 1.18 | 381287 | 1.40 | 60406 | 1.33 | 70419 | 1.57 |
| 11676 | 1.18 | 239096 | 1.40 | 270685 | 1.33 | 17131 | 1.56 |
| 214133 | 1.18 | 118452 | 1.40 | 21991 | 1.32 | 16987 | 1.56 |
| 68982 | 1.18 | 20878 | 1.40 | 13807 | 1.32 | 140486 | 1.56 |
| 56207 | 1.17 | 68616 | 1.39 | 103844 | 1.32 | 14325 | 1.55 |
| 22346 | 1.17 | 26432 | 1.39 | 382913 | 1.31 | 12215 | 1.55 |
| 231801 | 1.17 | novel_G000463 | 1.39 | 15460 | 1.31 | 23801 | 1.55 |
| 319722 | 1.17 | 110033 | 1.38 | 108816 | 1.31 | 73673 | 1.54 |
| 17436 | 1.17 | 20198 | 1.38 | 15571 | 1.30 | 13885 | 1.54 |
| novel_G000345 | 1.17 | novel_G000385 | 1.38 | 74053 | 1.30 | 329738 | 1.54 |
| 380753 | 1.17 | 193796 | 1.38 | novel_G000797 | 1.30 | 232714 | 1.54 |
| 381853 | 1.17 | 229699 | 1.38 | 18173 | 1.30 | 56702 | 1.54 |
| 217558 | 1.17 | 1E+08 | 1.37 | 22187 | 1.30 | 15937 | 1.54 |
| 66140 | 1.16 | 407786 | 1.37 | 30878 | 1.29 | 320301 | 1.54 |
| 72240 | 1.16 | 12428 | 1.37 | 12790 | 1.29 | 319162 | 1.54 |
| 72655 | 1.16 | 18019 | 1.36 | 193796 | 1.28 | 399558 | 1.53 |
| 414115 | 1.16 | 14704 | 1.36 | 20186 | 1.28 | 14428 | 1.53 |
| 240892 | 1.16 | 70454 | 1.36 | 381853 | 1.28 | 17873 | 1.53 |
| 19361 | 1.16 | novel_G000857 | 1.35 | 1E+08 | 1.27 | 230895 | 1.53 |
| 380912 | 1.15 | 270906 | 1.35 | 50997 | 1.27 | 435766 | 1.53 |
| 193796 | 1.15 | 20971 | 1.35 | 13824 | 1.27 | novel_G000199 | 1.53 |
| 271127 | 1.15 | 214292 | 1.34 | 208659 | 1.27 | 14723 | 1.52 |
| 67052 | 1.15 | 66234 | 1.34 | 53328 | 1.27 | 621976 | 1.52 |
| 227195 | 1.15 | 24052 | 1.34 | 76441 | 1.27 | 24052 | 1.52 |
| 67693 | 1.15 | 70252 | 1.34 | 399558 | 1.27 | 213391 | 1.51 |
| novel_G000525 | 1.15 | 1.01E+08 | 1.33 | 13685 | 1.26 | 217082 | 1.51 |
| 217316 | 1.15 | 320181 | 1.33 | 16210 | 1.26 | 18451 | 1.50 |
| 68816 | 1.15 | 28010 | 1.33 | 18453 | 1.26 | 58210 | 1.50 |
| 19309 | 1.15 | 223780 | 1.33 | 69354 | 1.26 | 29818 | 1.50 |
| 13605 | 1.14 | 208151 | 1.32 | 99543 | 1.26 | 319160 | 1.49 |
| 67669 | 1.14 | 12316 | 1.32 | 665270 | 1.26 | 13849 | 1.49 |
| 77777 | 1.14 | 214137 | 1.31 | 1.01E+08 | 1.25 | 78575 | 1.48 |
| 56522 | 1.14 | 1E+08 | 1.31 | 207596 | 1.25 | 14751 | 1.47 |
| 20133 | 1.14 | novel_G000897 | 1.31 | 67669 | 1.25 | 56018 | 1.47 |
| 629159 | 1.14 | 242050 | 1.31 | 17748 | 1.25 | 193796 | 1.46 |
| 23834 | 1.13 | 108000 | 1.30 | 237928 | 1.25 | 17060 | 1.46 |
| 68026 | 1.13 | 108912 | 1.30 | 213484 | 1.25 | 20197 | 1.46 |
| 19385 | 1.13 | 67103 | 1.30 | 14998 | 1.25 | 66234 | 1.46 |
| 20971 | 1.13 | 29861 | 1.30 | 1E+08 | 1.25 | 69002 | 1.45 |
| 66197 | 1.13 | 233107 | 1.30 | 14860 | 1.24 | 19329 | 1.45 |
| 13885 | 1.13 | 319722 | 1.29 | 17196 | 1.23 | 50997 | 1.45 |
| 17691 | 1.13 | 19329 | 1.29 | 93806 | 1.23 | 66447 | 1.44 |
| 12569 | 1.13 | 109212 | 1.29 | 26395 | 1.23 | 69454 | 1.44 |
| 17131 | 1.13 | 1E+08 | 1.29 | 19981 | 1.23 | 15277 | 1.44 |
| 217203 | 1.13 | 22352 | 1.29 | 67101 | 1.23 | 13807 | 1.44 |
| 14017 | 1.12 | 52815 | 1.28 | 108960 | 1.23 | 56631 | 1.44 |
| 19041 | 1.12 | 108811 | 1.28 | 19268 | 1.23 | 11602 | 1.44 |
| 18817 | 1.12 | 319707 | 1.28 | 69399 | 1.22 | 114663 | 1.43 |
| 70247 | 1.12 | 668253 | 1.28 | 12177 | 1.22 | 665306 | 1.43 |
| 78653 | 1.12 | 20186 | 1.27 | 621976 | 1.22 | 14245 | 1.43 |
| 384071 | 1.11 | 57435 | 1.27 | 67446 | 1.22 | 216438 | 1.43 |
| 11674 | 1.11 | 268319 | 1.27 | 16987 | 1.22 | 12836 | 1.42 |
| 381801 | 1.11 | 66197 | 1.27 | 217082 | 1.22 | 71361 | 1.42 |
| 105171 | 1.11 | 629159 | 1.27 | 626832 | 1.22 | 70788 | 1.42 |
| 229841 | 1.11 | 94089 | 1.26 | 208677 | 1.22 | 1E+08 | 1.42 |
| 108150 | 1.11 | 1.01E+08 | 1.26 | 230126 | 1.21 | 67170 | 1.42 |
| 71846 | 1.11 | 74107 | 1.26 | 56421 | 1.21 | 237847 | 1.42 |
| 19016 | 1.11 | novel_G000815 | 1.26 | 16322 | 1.21 | 80288 | 1.41 |
| 83436 | 1.11 | 217558 | 1.26 | 71897 | 1.21 | 13643 | 1.41 |
| 72269 | 1.11 | novel_G000719 | 1.26 | 94089 | 1.21 | 12953 | 1.41 |
| 17192 | 1.10 | 15270 | 1.26 | 27883 | 1.21 | 14704 | 1.41 |
| 15275 | 1.10 | 383348 | 1.26 | 1.05E+08 | 1.21 | 75084 | 1.41 |
| 53333 | 1.10 | 11443 | 1.25 | 56863 | 1.21 | 69399 | 1.41 |
| 1.01E+08 | 1.10 | 1.01E+08 | 1.25 | 17001 | 1.20 | 21991 | 1.41 |
| 11799 | 1.10 | 14066 | 1.25 | 16782 | 1.20 | 27981 | 1.40 |
| 12833 | 1.10 | 108816 | 1.25 | 80976 | 1.20 | 13806 | 1.40 |
| novel_G000247 | 1.10 | 12449 | 1.25 | 434350 | 1.20 | 20971 | 1.40 |
| 29818 | 1.10 | 52276 | 1.24 | 13002 | 1.19 | 108767 | 1.40 |
| 108912 | 1.10 | 211666 | 1.24 | 74246 | 1.19 | 252972 | 1.40 |
| 14630 | 1.10 | 1.01E+08 | 1.24 | 74476 | 1.19 | 74076 | 1.40 |
| 50783 | 1.10 | 399558 | 1.24 | 78512 | 1.19 | novel_G000498 | 1.39 |
| 74268 | 1.09 | 68671 | 1.24 | 432763 | 1.19 | novel_G000197 | 1.39 |
| 230753 | 1.09 | novel_G000494 | 1.24 | 18828 | 1.19 | 383348 | 1.39 |
| 17220 | 1.09 | 109689 | 1.24 | 78330 | 1.19 | 320678 | 1.39 |
| 20586 | 1.09 | 14428 | 1.24 | 12836 | 1.19 | 237928 | 1.39 |
| 68044 | 1.09 | 16526 | 1.24 | 16904 | 1.19 | 11496 | 1.38 |
| 76478 | 1.09 | 233071 | 1.24 | 57436 | 1.19 | novel_G000375 | 1.38 |
| 69639 | 1.09 | 20491 | 1.24 | 110208 | 1.18 | 66834 | 1.38 |
| 21681 | 1.09 | 18451 | 1.23 | 22042 | 1.18 | 68465 | 1.38 |
| 52504 | 1.09 | 109042 | 1.23 | 435766 | 1.18 | 382913 | 1.37 |
| 56441 | 1.09 | 73673 | 1.23 | 100910 | 1.18 | 12177 | 1.37 |
| 12235 | 1.08 | 59027 | 1.23 | 329384 | 1.18 | 66153 | 1.37 |
| 66556 | 1.08 | 50782 | 1.22 | 66610 | 1.17 | 56421 | 1.37 |
| 58859 | 1.08 | NM_001348198 | 1.22 | 12359 | 1.17 | 68763 | 1.37 |
| 239027 | 1.08 | 329650 | 1.22 | 67705 | 1.16 | 71664 | 1.37 |
| 19944 | 1.08 | 50791 | 1.20 | 22364 | 1.16 | 108960 | 1.37 |
| 19076 | 1.08 | 171531 | 1.20 | 70345 | 1.16 | 15166 | 1.37 |
| 236082 | 1.08 | 78767 | 1.20 | 56193 | 1.15 | 15423 | 1.36 |
| 69757 | 1.08 | 67170 | 1.20 | 29818 | 1.15 | 67088 | 1.36 |
| 626904 | 1.07 | 319162 | 1.20 | 78575 | 1.15 | 26401 | 1.36 |
| 230099 | 1.07 | 13358 | 1.19 | 68465 | 1.15 | novel_G000531 | 1.36 |
| 15366 | 1.07 | 68044 | 1.19 | 16475 | 1.15 | 52662 | 1.36 |
| 320842 | 1.07 | 380753 | 1.19 | 11565 | 1.14 | 230098 | 1.35 |
| 57444 | 1.07 | 12215 | 1.19 | 66151 | 1.14 | 12306 | 1.35 |
| novel_G000461 | 1.07 | 77533 | 1.19 | 380912 | 1.14 | 677884 | 1.35 |
| 13730 | 1.07 | 14751 | 1.19 | 1.01E+08 | 1.14 | 98170 | 1.35 |
| 12442 | 1.07 | 192188 | 1.18 | 67170 | 1.14 | 11513 | 1.35 |
| novel_G000491 | 1.07 | 21335 | 1.18 | 224530 | 1.13 | 12790 | 1.35 |
| 71241 | 1.07 | 1.01E+08 | 1.18 | 234683 | 1.13 | 74286 | 1.35 |
| 1E+08 | 1.06 | 14385 | 1.18 | 20409 | 1.13 | 236899 | 1.35 |
| 68052 | 1.06 | 22190 | 1.18 | 22339 | 1.13 | 104252 | 1.35 |
| novel_G000245 | 1.06 | 83673 | 1.18 | 14115 | 1.13 | 171531 | 1.35 |
| 66047 | 1.06 | 11717 | 1.17 | 30963 | 1.13 | 18132 | 1.34 |
| 29861 | 1.06 | 106757 | 1.17 | 268291 | 1.13 | 208659 | 1.34 |
| 224171 | 1.06 | 229841 | 1.17 | 66487 | 1.13 | 14281 | 1.33 |
| 116972 | 1.06 | 19652 | 1.17 | 1E+08 | 1.13 | 104080 | 1.33 |
| 76238 | 1.06 | 15275 | 1.17 | 83674 | 1.13 | 380928 | 1.33 |
| 1E+08 | 1.06 | 217316 | 1.16 | 59027 | 1.13 | 94089 | 1.32 |
| 66276 | 1.06 | 234396 | 1.16 | 68952 | 1.13 | 244238 | 1.32 |
| 71228 | 1.06 | 50997 | 1.16 | 20276 | 1.12 | 268291 | 1.32 |
| 53817 | 1.05 | 50708 | 1.15 | 12663 | 1.12 | 93806 | 1.32 |
| 67078 | 1.05 | 77595 | 1.15 | 243813 | 1.12 | 214239 | 1.32 |
| 171531 | 1.05 | 18817 | 1.15 | 72269 | 1.12 | 73720 | 1.32 |
| 14211 | 1.05 | 18822 | 1.15 | 68603 | 1.12 | 15275 | 1.32 |
| 70235 | 1.05 | 110253 | 1.15 | 12257 | 1.11 | 100705 | 1.31 |
| 22228 | 1.05 | 380912 | 1.15 | 74843 | 1.11 | 69574 | 1.31 |
| 207259 | 1.05 | 214424 | 1.15 | 67689 | 1.11 | 72240 | 1.31 |
| 216345 | 1.05 | 140580 | 1.15 | 668253 | 1.11 | 66166 | 1.31 |
| 233876 | 1.05 | 14235 | 1.14 | 329650 | 1.11 | 380912 | 1.31 |
| 70640 | 1.05 | 13601 | 1.14 | 109672 | 1.11 | 26395 | 1.31 |
| 66570 | 1.05 | 216345 | 1.14 | 71361 | 1.10 | 14579 | 1.31 |
| 18810 | 1.05 | 104252 | 1.14 | 69656 | 1.10 | 67446 | 1.31 |
| 26914 | 1.05 | 108767 | 1.14 | 20682 | 1.10 | 13853 | 1.31 |
| 74081 | 1.05 | 214133 | 1.14 | 20562 | 1.09 | 15460 | 1.31 |
| 229541 | 1.05 | 72657 | 1.14 | 18126 | 1.09 | 216858 | 1.31 |
| 111241 | 1.05 | 22333 | 1.13 | 78514 | 1.09 | novel_G000385 | 1.30 |
| 78890 | 1.04 | 319493 | 1.13 | novel_G000318 | 1.09 | 20657 | 1.30 |
| 56532 | 1.04 | 76441 | 1.13 | novel_G000976 | 1.09 | 110196 | 1.30 |
| 1E+08 | 1.04 | 67486 | 1.13 | 1E+08 | 1.08 | novel_G000091 | 1.29 |
| 276905 | 1.04 | 76238 | 1.12 | 226421 | 1.08 | 76441 | 1.29 |
| 105440 | 1.04 | 69454 | 1.12 | 230806 | 1.08 | 213484 | 1.28 |
| 77782 | 1.04 | 229599 | 1.12 | 15275 | 1.08 | 1E+08 | 1.28 |
| 107435 | 1.04 | 16421 | 1.12 | 15166 | 1.08 | 15258 | 1.28 |
| 13358 | 1.04 | 14205 | 1.11 | 17859 | 1.08 | 12955 | 1.28 |
| novel_G000051 | 1.04 | 12257 | 1.11 | 17750 | 1.08 | 66610 | 1.28 |
| 56505 | 1.04 | 14598 | 1.11 | 56289 | 1.08 | 114606 | 1.28 |
| 21877 | 1.03 | 13497 | 1.11 | 13358 | 1.07 | 70345 | 1.28 |
| 217837 | 1.03 | 112415 | 1.11 | 320184 | 1.07 | 18302 | 1.27 |
| 70025 | 1.03 | 12359 | 1.11 | 66234 | 1.07 | 235435 | 1.27 |
| 14381 | 1.03 | 78733 | 1.11 | 207728 | 1.07 | 13358 | 1.27 |
| 68743 | 1.03 | 1.01E+08 | 1.11 | 104215 | 1.07 | 208677 | 1.26 |
| 1.02E+08 | 1.03 | 105440 | 1.11 | 77619 | 1.07 | 17319 | 1.26 |
| 18822 | 1.03 | 382913 | 1.11 | 22190 | 1.06 | 216850 | 1.26 |
| 70312 | 1.03 | 57436 | 1.10 | 1.01E+08 | 1.06 | 207728 | 1.25 |
| 27214 | 1.02 | 76612 | 1.10 | 244059 | 1.06 | 110208 | 1.25 |
| 194268 | 1.02 | 68969 | 1.10 | 15423 | 1.06 | 14766 | 1.25 |
| 17216 | 1.02 | 234577 | 1.10 | 15399 | 1.06 | 20148 | 1.24 |
| 67305 | 1.02 | 27214 | 1.10 | 17131 | 1.06 | 56863 | 1.24 |
| 68971 | 1.02 | 329384 | 1.10 | 242050 | 1.06 | 216459 | 1.24 |
| 20341 | 1.02 | 217038 | 1.10 | 20364 | 1.06 | 213649 | 1.24 |
| 223920 | 1.02 | 64009 | 1.10 | 29861 | 1.06 | novel_G000309 | 1.24 |
| 621976 | 1.02 | 623781 | 1.10 | 109042 | 1.06 | 100910 | 1.24 |
| 219144 | 1.02 | 22187 | 1.10 | 216858 | 1.05 | 29862 | 1.23 |
| 72662 | 1.02 | 67052 | 1.10 | 79362 | 1.05 | 67971 | 1.23 |
| 66493 | 1.02 | 68982 | 1.10 | 13601 | 1.05 | 270685 | 1.23 |
| 69860 | 1.01 | 1E+08 | 1.10 | 229699 | 1.05 | 1.01E+08 | 1.23 |
| 381022 | 1.01 | 18193 | 1.10 | 66359 | 1.05 | 1.01E+08 | 1.23 |
| 214424 | 1.01 | 11799 | 1.09 | 67103 | 1.05 | 230126 | 1.23 |
| 17319 | 1.01 | 223920 | 1.09 | 381287 | 1.05 | 208777 | 1.22 |
| 223870 | 1.01 | 329421 | 1.09 | 105732 | 1.05 | 68603 | 1.22 |
| 66929 | 1.01 | 56258 | 1.09 | 76238 | 1.05 | 110310 | 1.21 |
| 13555 | 1.01 | 68845 | 1.09 | 383348 | 1.05 | novel_G000797 | 1.21 |
| 16905 | 1.01 | 230806 | 1.09 | 252972 | 1.04 | 67689 | 1.21 |
| novel_G000468 | 1.01 | 20197 | 1.09 | 22352 | 1.04 | 232232 | 1.21 |
| 107817 | 1.00 | 73072 | 1.09 | 230088 | 1.04 | 11770 | 1.20 |
| 12534 | 1.00 | 12177 | 1.09 | 68949 | 1.04 | 19309 | 1.20 |
| 103573 | 1.00 | novel_G000681 | 1.08 | 246256 | 1.04 | 22288 | 1.20 |
| 18439 | -1.00 | 69354 | 1.08 | 20655 | 1.04 | 213988 | 1.20 |
| 21664 | -1.00 | 1.01E+08 | 1.08 | 330737 | 1.04 | 22346 | 1.20 |
| 231507 | -1.00 | 14630 | 1.08 | 68044 | 1.04 | 1.05E+08 | 1.20 |
| 77462 | -1.00 | 68465 | 1.08 | 16859 | 1.04 | 75747 | 1.19 |
| 14239 | -1.00 | 78284 | 1.08 | 232232 | 1.04 | 170720 | 1.19 |
| 80837 | -1.01 | 236733 | 1.08 | 20203 | 1.04 | 380705 | 1.19 |
| 74201 | -1.01 | 239027 | 1.08 | 232430 | 1.03 | 282619 | 1.19 |
| 12876 | -1.01 | 20409 | 1.08 | novel_G000309 | 1.03 | 22190 | 1.19 |
| 50909 | -1.01 | 399101 | 1.08 | 214239 | 1.03 | 330737 | 1.19 |
| 71519 | -1.01 | 330737 | 1.08 | 14066 | 1.03 | 16859 | 1.19 |
| 381126 | -1.01 | 107934 | 1.08 | 319190 | 1.03 | 320440 | 1.19 |
| 19883 | -1.01 | 13849 | 1.08 | NM_001348198 | 1.03 | 233789 | 1.19 |
| 545471 | -1.01 | 72269 | 1.08 | 18193 | 1.03 | 79201 | 1.19 |
| 16404 | -1.01 | 76222 | 1.07 | 13643 | 1.03 | novel_G000525 | 1.19 |
| 69528 | -1.01 | 219144 | 1.07 | 114663 | 1.03 | novel_G000928 | 1.19 |
| 76088 | -1.01 | 13730 | 1.07 | 72590 | 1.03 | 14598 | 1.18 |
| 228911 | -1.01 | novel_G000318 | 1.07 | 11496 | 1.02 | 26388 | 1.18 |
| 20361 | -1.01 | 13167 | 1.07 | 66612 | 1.02 | 17133 | 1.18 |
| 67530 | -1.01 | 213948 | 1.07 | 53322 | 1.02 | 12569 | 1.18 |
| 238330 | -1.01 | 233406 | 1.07 | 52662 | 1.02 | 230088 | 1.18 |
| 13537 | -1.02 | 22339 | 1.07 | 21942 | 1.02 | 13002 | 1.18 |
| 12263 | -1.02 | 22228 | 1.07 | 70025 | 1.02 | 20887 | 1.18 |
| 103220 | -1.02 | 12442 | 1.06 | 69652 | 1.02 | 231147 | 1.18 |
| 20338 | -1.02 | 74470 | 1.06 | 214424 | 1.02 | 235431 | 1.17 |
| 13110 | -1.02 | 18194 | 1.06 | 110196 | 1.02 | 13685 | 1.17 |
| 59031 | -1.02 | novel_G000646 | 1.06 | 665306 | 1.02 | 18412 | 1.17 |
| 93889 | -1.02 | 230899 | 1.06 | 76612 | 1.02 | 18563 | 1.17 |
| 17684 | -1.02 | 328967 | 1.06 | novel_G000385 | 1.02 | 70129 | 1.17 |
| 23831 | -1.02 | 71846 | 1.06 | 22371 | 1.01 | 60406 | 1.16 |
| 67168 | -1.02 | 74173 | 1.06 | 56772 | 1.01 | 22187 | 1.16 |
| 17069 | -1.02 | 12843 | 1.06 | 229599 | 1.01 | 1E+08 | 1.16 |
| 383295 | -1.03 | 244550 | 1.05 | 16421 | 1.01 | 78514 | 1.15 |
| 264895 | -1.03 | 70218 | 1.05 | 234577 | 1.00 | 230899 | 1.15 |
| 338351 | -1.03 | 17855 | 1.05 | 72341 | 1.00 | 14998 | 1.15 |
| novel_G000745 | -1.03 | 110095 | 1.05 | 17391 | 1.00 | 269700 | 1.15 |
| 68192 | -1.03 | 30939 | 1.05 | 21753 | 1.00 | 16475 | 1.15 |
| 70354 | -1.03 | 76650 | 1.05 | 30838 | 1.00 | 269198 | 1.14 |
| 210503 | -1.03 | 13002 | 1.05 | 76927 | 1.00 | 16890 | 1.14 |
| 213332 | -1.04 | 74127 | 1.05 | 107503 | 1.00 | 194231 | 1.14 |
| 1E+08 | -1.04 | 13685 | 1.05 | 56615 | 1.00 | 13121 | 1.14 |
| 78892 | -1.04 | 97165 | 1.05 | 668212 | -1.00 | 319190 | 1.14 |
| 67636 | -1.04 | 11605 | 1.05 | 18751 | -1.00 | 226421 | 1.14 |
| 19092 | -1.04 | 66054 | 1.05 | 20256 | -1.00 | 246256 | 1.14 |
| 69504 | -1.04 | 30878 | 1.05 | 66634 | -1.00 | 29861 | 1.13 |
| 54445 | -1.04 | 66556 | 1.04 | 73368 | -1.01 | 57436 | 1.13 |
| 11828 | -1.04 | 231044 | 1.04 | 231842 | -1.01 | 12514 | 1.13 |
| 24051 | -1.04 | 268291 | 1.04 | 16175 | -1.01 | 20249 | 1.13 |
| 1E+08 | -1.04 | 14910 | 1.04 | 71885 | -1.01 | 18126 | 1.13 |
| 14009 | -1.04 | 14998 | 1.04 | 94212 | -1.01 | 78330 | 1.13 |
| 11492 | -1.04 | 11958 | 1.04 | 320204 | -1.01 | 27883 | 1.13 |
| 80861 | -1.04 | 11816 | 1.04 | 224093 | -1.01 | 53328 | 1.13 |
| 80884 | -1.04 | 414085 | 1.03 | 106795 | -1.01 | novel_G000494 | 1.13 |
| 13835 | -1.05 | 74843 | 1.03 | 74315 | -1.01 | 67451 | 1.13 |
| 21898 | -1.05 | 216505 | 1.03 | 66354 | -1.01 | 217893 | 1.13 |
| 69329 | -1.05 | 224171 | 1.03 | 70110 | -1.01 | 72333 | 1.13 |
| 73442 | -1.05 | 231070 | 1.03 | 12321 | -1.01 | 20444 | 1.12 |
| 77975 | -1.06 | 26388 | 1.03 | 269952 | -1.01 | 105732 | 1.12 |
| 214106 | -1.06 | 30838 | 1.03 | 72205 | -1.02 | 74202 | 1.12 |
| 22134 | -1.06 | 70129 | 1.03 | 68026 | -1.02 | 17859 | 1.12 |
| 66058 | -1.06 | 20686 | 1.03 | 77889 | -1.02 | 71091 | 1.11 |
| 67111 | -1.06 | 26395 | 1.02 | 13171 | -1.02 | 76238 | 1.11 |
| 245867 | -1.06 | 77619 | 1.02 | 19092 | -1.02 | 66395 | 1.11 |
| 171285 | -1.06 | 1E+08 | 1.02 | 271842 | -1.02 | 18453 | 1.11 |
| 73490 | -1.06 | 14283 | 1.02 | 381463 | -1.03 | 98314 | 1.11 |
| 12870 | -1.06 | 71406 | 1.02 | 15574 | -1.03 | 74183 | 1.11 |
| 252973 | -1.06 | 74476 | 1.02 | 76843 | -1.03 | 338360 | 1.11 |
| 68625 | -1.06 | 107029 | 1.02 | 1.01E+08 | -1.03 | 77864 | 1.11 |
| 218639 | -1.06 | 269695 | 1.02 | 69544 | -1.03 | 20411 | 1.11 |
| 233552 | -1.06 | 216456 | 1.02 | 72787 | -1.04 | 75668 | 1.11 |
| 20962 | -1.07 | 72341 | 1.02 | 69066 | -1.04 | 214150 | 1.10 |
| 17207 | -1.07 | 12235 | 1.02 | 381306 | -1.04 | 16973 | 1.10 |
| 68874 | -1.07 | 72119 | 1.02 | 230376 | -1.04 | 11600 | 1.10 |
| novel_G000651 | -1.07 | 77022 | 1.01 | 16470 | -1.05 | 211666 | 1.10 |
| 17948 | -1.07 | 1.01E+08 | 1.01 | 66141 | -1.05 | 105559 | 1.10 |
| 54601 | -1.07 | 12534 | 1.01 | 11989 | -1.05 | 66487 | 1.10 |
| 18174 | -1.07 | 381148 | 1.01 | 17193 | -1.05 | 69354 | 1.10 |
| 216867 | -1.07 | 56421 | 1.01 | 19365 | -1.05 | 1.01E+08 | 1.10 |
| 12577 | -1.07 | 67856 | 1.01 | 26408 | -1.05 | 20276 | 1.10 |
| 223649 | -1.07 | 67951 | 1.01 | 103511 | -1.05 | 16478 | 1.10 |
| 69962 | -1.07 | 14789 | 1.01 | 1E+08 | -1.05 | 242691 | 1.10 |
| 16432 | -1.08 | 1E+08 | 1.01 | 211651 | -1.06 | 215113 | 1.10 |
| 319430 | -1.08 | novel_G000339 | 1.00 | 630579 | -1.06 | 1.01E+08 | 1.09 |
| 66065 | -1.08 | 15366 | 1.00 | 13809 | -1.07 | 14066 | 1.09 |
| 102278 | -1.08 | 14605 | 1.00 | 73610 | -1.07 | 73251 | 1.09 |
| 18707 | -1.08 | 110956 | 1.00 | 69716 | -1.07 | 214133 | 1.09 |
| 235504 | -1.08 | 229445 | -1.00 | 13385 | -1.08 | 74080 | 1.09 |
| 217344 | -1.08 | 216198 | -1.00 | 226351 | -1.08 | 319939 | 1.09 |
| 242362 | -1.08 | 22032 | -1.00 | 11459 | -1.08 | 107581 | 1.09 |
| 54447 | -1.08 | 192231 | -1.00 | 66246 | -1.08 | 633057 | 1.09 |
| 24058 | -1.09 | 74048 | -1.00 | 19075 | -1.08 | 18173 | 1.09 |
| 14632 | -1.09 | 13805 | -1.01 | 20621 | -1.08 | 104601 | 1.08 |
| 226641 | -1.09 | novel_G000805 | -1.01 | 264895 | -1.08 | 227195 | 1.08 |
| 213522 | -1.09 | 14366 | -1.01 | 229949 | -1.08 | 107503 | 1.08 |
| 74959 | -1.09 | 244653 | -1.01 | 72113 | -1.08 | 12904 | 1.08 |
| 229949 | -1.10 | 22761 | -1.01 | 13139 | -1.09 | 226251 | 1.08 |
| 170835 | -1.10 | 68659 | -1.02 | 57138 | -1.09 | 329251 | 1.08 |
| 58182 | -1.10 | 16599 | -1.02 | 28113 | -1.09 | 66612 | 1.08 |
| 320982 | -1.10 | 13110 | -1.02 | 16923 | -1.09 | 381270 | 1.08 |
| 243967 | -1.10 | 56338 | -1.02 | 84653 | -1.10 | 76816 | 1.07 |
| 237930 | -1.10 | 17850 | -1.02 | 11988 | -1.10 | 11830 | 1.07 |
| 97122 | -1.10 | 21899 | -1.02 | 72040 | -1.10 | novel_G000285 | 1.07 |
| 19365 | -1.10 | 237253 | -1.02 | 226691 | -1.11 | 22402 | 1.07 |
| 214048 | -1.10 | 353235 | -1.03 | 18968 | -1.11 | 18129 | 1.07 |
| 12830 | -1.10 | 77889 | -1.03 | 20558 | -1.11 | 30963 | 1.07 |
| 259044 | -1.10 | 74760 | -1.03 | 12616 | -1.11 | 68949 | 1.07 |
| 80907 | -1.10 | 67416 | -1.03 | 16186 | -1.11 | 72269 | 1.07 |
| 171210 | -1.10 | 381511 | -1.04 | 212980 | -1.11 | 100169 | 1.07 |
| 22781 | -1.10 | 17063 | -1.04 | 23892 | -1.11 | 68969 | 1.07 |
| 234854 | -1.11 | 14451 | -1.04 | 71643 | -1.11 | 104885 | 1.07 |
| novel_G000120 | -1.11 | 20361 | -1.04 | 1.01E+08 | -1.11 | 69749 | 1.07 |
| 75731 | -1.11 | 68728 | -1.04 | novel_G000058 | -1.11 | 381022 | 1.06 |
| 53322 | -1.11 | 15064 | -1.04 | 77975 | -1.11 | 77619 | 1.06 |
| 27362 | -1.11 | 59031 | -1.04 | 67416 | -1.11 | 18822 | 1.06 |
| 72512 | -1.11 | 93838 | -1.04 | 71206 | -1.12 | 329384 | 1.06 |
| 235493 | -1.12 | 68127 | -1.05 | 12862 | -1.12 | 74081 | 1.06 |
| 22351 | -1.12 | 319236 | -1.05 | 17951 | -1.12 | 15259 | 1.06 |
| 20530 | -1.12 | 72512 | -1.05 | 18712 | -1.12 | 215653 | 1.06 |
| 12832 | -1.12 | 73998 | -1.05 | 319352 | -1.12 | 13824 | 1.06 |
| 215814 | -1.12 | 11304 | -1.05 | novel_G000686 | -1.13 | 74205 | 1.06 |
| 75605 | -1.12 | 1E+08 | -1.06 | 19368 | -1.14 | 12257 | 1.06 |
| 212974 | -1.12 | 213522 | -1.06 | 14972 | -1.14 | 66151 | 1.05 |
| 69583 | -1.12 | 68176 | -1.06 | 14178 | -1.15 | 244059 | 1.05 |
| 226098 | -1.12 | 80907 | -1.06 | 15901 | -1.15 | 100129 | 1.05 |
| 18220 | -1.12 | 240753 | -1.06 | 230766 | -1.15 | 16210 | 1.05 |
| 77889 | -1.12 | 15483 | -1.07 | 78892 | -1.15 | 56490 | 1.05 |
| 53614 | -1.13 | 74761 | -1.07 | NM_001348100 | -1.16 | 109711 | 1.05 |
| 210853 | -1.13 | 14538 | -1.07 | 116847 | -1.16 | 231070 | 1.05 |
| 1E+08 | -1.13 | 11980 | -1.08 | 58182 | -1.16 | novel_G000173 | 1.05 |
| 15512 | -1.13 | 16186 | -1.08 | 19366 | -1.16 | 68813 | 1.04 |
| 14991 | -1.13 | 68055 | -1.08 | 72151 | -1.17 | 12821 | 1.04 |
| 56745 | -1.13 | 1.01E+08 | -1.08 | 12266 | -1.17 | 320184 | 1.04 |
| novel_G000690 | -1.13 | 1.01E+08 | -1.08 | 433804 | -1.17 | 56436 | 1.04 |
| 213311 | -1.13 | 50723 | -1.08 | 14555 | -1.18 | 78286 | 1.04 |
| 72500 | -1.13 | 327978 | -1.08 | 18933 | -1.18 | 229599 | 1.04 |
| 21827 | -1.13 | 58222 | -1.08 | 667118 | -1.18 | 381801 | 1.04 |
| 70300 | -1.13 | 227671 | -1.09 | 110784 | -1.19 | 620078 | 1.04 |
| 235345 | -1.13 | 75710 | -1.09 | 106877 | -1.19 | 237858 | 1.04 |
| 14828 | -1.13 | 17067 | -1.09 | 379043 | -1.19 | novel_G000339 | 1.04 |
| 668215 | -1.14 | 56309 | -1.09 | 18973 | -1.19 | 99543 | 1.04 |
| 19273 | -1.14 | 17951 | -1.09 | 207704 | -1.19 | 1E+08 | 1.03 |
| 72997 | -1.14 | 230073 | -1.09 | 68550 | -1.20 | 14205 | 1.03 |
| 218203 | -1.14 | 67475 | -1.10 | 11538 | -1.20 | 58991 | 1.03 |
| 1E+08 | -1.14 | novel_G000120 | -1.10 | 26434 | -1.21 | 79362 | 1.03 |
| 13809 | -1.14 | 232560 | -1.10 | 54199 | -1.21 | 72282 | 1.03 |
| 18105 | -1.14 | 93732 | -1.11 | 72121 | -1.21 | 71897 | 1.03 |
| 13924 | -1.14 | 26434 | -1.11 | 232560 | -1.22 | 70025 | 1.03 |
| 27356 | -1.14 | 20215 | -1.11 | 17940 | -1.22 | 13829 | 1.03 |
| 211135 | -1.14 | 22017 | -1.11 | 17217 | -1.22 | 353502 | 1.03 |
| 192970 | -1.14 | 12447 | -1.12 | 11492 | -1.22 | 21366 | 1.03 |
| 16572 | -1.15 | 231474 | -1.12 | 237877 | -1.22 | 15273 | 1.02 |
| 70110 | -1.15 | 11603 | -1.12 | 50723 | -1.22 | 268721 | 1.02 |
| 239552 | -1.15 | 72107 | -1.12 | 117167 | -1.23 | 72194 | 1.02 |
| 67865 | -1.15 | 108902 | -1.12 | 16007 | -1.24 | 13518 | 1.02 |
| 227059 | -1.15 | 226351 | -1.12 | 18105 | -1.24 | 17691 | 1.02 |
| 11474 | -1.15 | 20210 | -1.12 | 72672 | -1.24 | 227737 | 1.02 |
| 69870 | -1.15 | 67474 | -1.12 | 216867 | -1.24 | 21389 | 1.02 |
| 629016 | -1.15 | 18442 | -1.13 | 213556 | -1.25 | 18415 | 1.02 |
| 74954 | -1.15 | 67530 | -1.13 | 66065 | -1.25 | 72091 | 1.02 |
| 76737 | -1.16 | 70354 | -1.13 | 13110 | -1.25 | 75259 | 1.01 |
| 66274 | -1.16 | novel_G000767 | -1.13 | 72107 | -1.25 | 14284 | 1.01 |
| NM_001348222 | -1.16 | 12183 | -1.13 | 15006 | -1.26 | 415115 | 1.01 |
| 68867 | -1.16 | 21926 | -1.14 | 319430 | -1.26 | 67860 | 1.01 |
| novel_G000300 | -1.16 | 101488 | -1.14 | 94094 | -1.26 | 243813 | 1.01 |
| 97998 | -1.17 | 13641 | -1.15 | 18108 | -1.27 | 192188 | 1.01 |
| 70370 | -1.17 | 11642 | -1.15 | 23886 | -1.27 | 1.01E+08 | 1.01 |
| 12916 | -1.17 | 379043 | -1.15 | 224697 | -1.27 | NM_001348198 | 1.01 |
| 245527 | -1.17 | 70561 | -1.15 | 17427 | -1.27 | 50708 | 1.01 |
| 71841 | -1.18 | 56448 | -1.16 | 11910 | -1.28 | 76854 | 1.01 |
| 17777 | -1.18 | 1E+08 | -1.16 | 93876 | -1.28 | 60527 | 1.00 |
| 213389 | -1.18 | 80884 | -1.16 | 12994 | -1.28 | 56193 | 1.00 |
| 19009 | -1.18 | 107227 | -1.16 | 244653 | -1.29 | novel_G000445 | 1.00 |
| novel_G000864 | -1.18 | 26968 | -1.17 | 110696 | -1.29 | novel_G000364 | 1.00 |
| 72560 | -1.18 | 104086 | -1.17 | 72512 | -1.29 | 245007 | 1.00 |
| 17939 | -1.18 | 54598 | -1.17 | 26968 | -1.29 | 66603 | -1.00 |
| 245827 | -1.18 | 83397 | -1.17 | 22654 | -1.29 | 668212 | -1.00 |
| 11694 | -1.19 | 20850 | -1.17 | 14764 | -1.29 | 235559 | -1.00 |
| novel_G000767 | -1.19 | 85031 | -1.17 | 73420 | -1.29 | 52710 | -1.00 |
| 16599 | -1.19 | 13531 | -1.17 | 209558 | -1.29 | 22596 | -1.01 |
| 71481 | -1.19 | 74481 | -1.18 | 16912 | -1.29 | 18712 | -1.01 |
| 16922 | -1.19 | 74153 | -1.18 | 16950 | -1.30 | 66752 | -1.01 |
| 228550 | -1.19 | 15039 | -1.18 | 71738 | -1.30 | 66863 | -1.01 |
| 66263 | -1.19 | 1E+08 | -1.19 | 72560 | -1.30 | 98682 | -1.01 |
| 71586 | -1.20 | 192970 | -1.19 | 207181 | -1.30 | novel_G000919 | -1.01 |
| 18712 | -1.20 | 233529 | -1.19 | 58176 | -1.31 | 15353 | -1.01 |
| 17346 | -1.20 | 11775 | -1.20 | 101488 | -1.31 | novel_G000415 | -1.01 |
| 117590 | -1.20 | 619715 | -1.20 | 381066 | -1.31 | 319701 | -1.02 |
| 192120 | -1.20 | 72560 | -1.20 | 101320 | -1.31 | 67177 | -1.02 |
| 66102 | -1.21 | 76429 | -1.21 | 20957 | -1.32 | 12519 | -1.02 |
| 74580 | -1.21 | 69066 | -1.21 | 14373 | -1.32 | 330173 | -1.02 |
| 1E+08 | -1.21 | 629016 | -1.22 | 384701 | -1.32 | 17220 | -1.02 |
| novel_G000652 | -1.21 | 16950 | -1.22 | 76429 | -1.32 | 319433 | -1.02 |
| 15039 | -1.21 | 11492 | -1.22 | 67138 | -1.32 | 216439 | -1.02 |
| 18826 | -1.21 | 545156 | -1.23 | 70300 | -1.33 | 240261 | -1.02 |
| 60533 | -1.21 | 219151 | -1.23 | 12369 | -1.33 | 12977 | -1.02 |
| 16780 | -1.21 | 1E+08 | -1.23 | 11642 | -1.34 | 213012 | -1.03 |
| 171504 | -1.21 | 1E+08 | -1.24 | 1E+08 | -1.34 | 57434 | -1.03 |
| 226255 | -1.22 | 239122 | -1.24 | 218311 | -1.35 | 66262 | -1.03 |
| 237711 | -1.22 | 384701 | -1.24 | 214901 | -1.35 | 231123 | -1.03 |
| 11988 | -1.22 | 11459 | -1.26 | 68126 | -1.36 | 23954 | -1.03 |
| novel_G000734 | -1.22 | 20621 | -1.26 | 15039 | -1.36 | 78658 | -1.03 |
| 142980 | -1.23 | 68526 | -1.26 | 13655 | -1.37 | 11307 | -1.03 |
| 100732 | -1.23 | 78771 | -1.26 | 15205 | -1.37 | 19153 | -1.03 |
| 24110 | -1.23 | 68852 | -1.26 | 234854 | -1.37 | 99349 | -1.04 |
| 77041 | -1.23 | 58182 | -1.26 | 67246 | -1.37 | 240327 | -1.04 |
| novel_G000095 | -1.23 | 214968 | -1.27 | 214968 | -1.37 | 101488 | -1.04 |
| 320292 | -1.23 | 16408 | -1.27 | 231214 | -1.37 | 276919 | -1.04 |
| 1.01E+08 | -1.23 | 217258 | -1.27 | 13479 | -1.37 | 237911 | -1.04 |
| 83397 | -1.23 | 16404 | -1.28 | 107751 | -1.38 | 56554 | -1.04 |
| 319236 | -1.24 | 625530 | -1.28 | 268930 | -1.39 | 16913 | -1.04 |
| 23887 | -1.24 | 67138 | -1.28 | 19085 | -1.39 | 13531 | -1.05 |
| 382985 | -1.24 | 242362 | -1.29 | 100604 | -1.39 | 17105 | -1.05 |
| NM_001348168 | -1.24 | 18191 | -1.29 | 93875 | -1.40 | 83433 | -1.05 |
| 17067 | -1.24 | 78749 | -1.30 | 637004 | -1.40 | 18755 | -1.05 |
| 56316 | -1.24 | 217410 | -1.30 | 21926 | -1.40 | 52033 | -1.05 |
| 14070 | -1.24 | 207181 | -1.31 | 16400 | -1.41 | 328263 | -1.05 |
| 241520 | -1.24 | 268656 | -1.31 | 13982 | -1.41 | 244653 | -1.05 |
| 50765 | -1.24 | 70370 | -1.32 | 21827 | -1.41 | 264895 | -1.05 |
| 619312 | -1.24 | 208869 | -1.32 | 17067 | -1.41 | 23892 | -1.06 |
| 26367 | -1.24 | 1E+08 | -1.32 | 243277 | -1.41 | 28113 | -1.06 |
| 244237 | -1.25 | 105859 | -1.32 | 192190 | -1.41 | 237211 | -1.06 |
| 244646 | -1.25 | 239559 | -1.32 | 213389 | -1.41 | 171210 | -1.06 |
| 19791 | -1.25 | 23964 | -1.32 | 15364 | -1.41 | 68285 | -1.06 |
| 54614 | -1.25 | 213556 | -1.33 | 14585 | -1.42 | 106344 | -1.06 |
| 1E+08 | -1.25 | 17329 | -1.33 | 1.01E+08 | -1.42 | 75778 | -1.06 |
| 219151 | -1.25 | 18574 | -1.33 | 276919 | -1.42 | 72040 | -1.06 |
| 319263 | -1.26 | 218461 | -1.33 | 16803 | -1.42 | 547253 | -1.06 |
| 13641 | -1.26 | 15205 | -1.33 | 13537 | -1.43 | 78757 | -1.06 |
| 60440 | -1.26 | 70110 | -1.33 | 14281 | -1.43 | 69066 | -1.07 |
| 12183 | -1.26 | 78757 | -1.33 | 667666 | -1.44 | 93732 | -1.07 |
| 71795 | -1.26 | 74315 | -1.33 | 16970 | -1.45 | 319236 | -1.07 |
| 16175 | -1.26 | 52882 | -1.34 | 21853 | -1.45 | 170770 | -1.07 |
| 27219 | -1.26 | 74147 | -1.34 | 68306 | -1.46 | 626391 | -1.07 |
| 78070 | -1.26 | 15567 | -1.34 | novel_G000816 | -1.46 | 14964 | -1.07 |
| 241633 | -1.27 | 1E+08 | -1.35 | 78757 | -1.46 | 208624 | -1.07 |
| 17996 | -1.27 | novel_G000876 | -1.35 | 16011 | -1.47 | 232560 | -1.08 |
| 232748 | -1.27 | 12182 | -1.35 | 16716 | -1.47 | 15574 | -1.08 |
| 319278 | -1.27 | 93878 | -1.35 | 73737 | -1.47 | 60530 | -1.08 |
| 15205 | -1.27 | 110454 | -1.35 | 76088 | -1.47 | 331188 | -1.08 |
| 20229 | -1.27 | 1E+08 | -1.35 | 81879 | -1.47 | novel_G000340 | -1.08 |
| 320571 | -1.28 | 209558 | -1.35 | 233876 | -1.47 | 26968 | -1.08 |
| 55948 | -1.28 | 17540 | -1.36 | 73998 | -1.47 | 71643 | -1.08 |
| 67009 | -1.28 | 81879 | -1.37 | 11447 | -1.48 | 66354 | -1.08 |
| 18791 | -1.28 | 13655 | -1.37 | 214952 | -1.48 | 12616 | -1.09 |
| 231946 | -1.28 | 109676 | -1.38 | 12977 | -1.48 | 16881 | -1.09 |
| 93891 | -1.29 | 12977 | -1.38 | 24066 | -1.48 | 107869 | -1.09 |
| 11541 | -1.29 | 214952 | -1.38 | 219151 | -1.48 | 21939 | -1.09 |
| 238161 | -1.29 | 16175 | -1.38 | 110454 | -1.49 | 16186 | -1.09 |
| 24066 | -1.29 | 16400 | -1.39 | 329002 | -1.49 | 21857 | -1.09 |
| 70381 | -1.29 | 218311 | -1.39 | 211064 | -1.49 | 20684 | -1.09 |
| 19260 | -1.29 | 16425 | -1.39 | 74020 | -1.50 | 71876 | -1.09 |
| 240753 | -1.29 | 71519 | -1.39 | 93837 | -1.50 | 22654 | -1.09 |
| 70266 | -1.29 | 224093 | -1.41 | 1E+08 | -1.50 | 16572 | -1.09 |
| 20620 | -1.29 | 72361 | -1.41 | 67896 | -1.51 | 320204 | -1.09 |
| 75785 | -1.30 | 64385 | -1.42 | 109032 | -1.51 | 14985 | -1.10 |
| 1E+08 | -1.30 | 26399 | -1.43 | 13434 | -1.52 | 445007 | -1.10 |
| novel_G000654 | -1.30 | 13139 | -1.43 | 94279 | -1.52 | 257633 | -1.10 |
| 117606 | -1.30 | 68632 | -1.43 | 101809 | -1.52 | 72113 | -1.10 |
| 14085 | -1.30 | 320664 | -1.43 | 319487 | -1.52 | 72512 | -1.10 |
| 619715 | -1.31 | 26367 | -1.43 | 104759 | -1.53 | 18971 | -1.10 |
| 1.01E+08 | -1.31 | 1E+08 | -1.44 | 11307 | -1.53 | 14979 | -1.10 |
| 74048 | -1.31 | 232087 | -1.44 | 227059 | -1.53 | 246727 | -1.10 |
| 12862 | -1.31 | 11307 | -1.44 | 75292 | -1.54 | 74041 | -1.10 |
| 230073 | -1.31 | 13982 | -1.44 | 54598 | -1.54 | 66141 | -1.10 |
| 56219 | -1.31 | 21827 | -1.44 | 667977 | -1.54 | 114714 | -1.10 |
| 208285 | -1.31 | 16970 | -1.44 | 18022 | -1.55 | 71924 | -1.10 |
| 14347 | -1.31 | 17940 | -1.45 | 20344 | -1.55 | 20621 | -1.10 |
| 11603 | -1.32 | 72500 | -1.45 | 208943 | -1.55 | 67896 | -1.10 |
| 11444 | -1.32 | 66645 | -1.45 | 70809 | -1.55 | 19698 | -1.10 |
| 68737 | -1.32 | 71738 | -1.46 | 208869 | -1.56 | 12362 | -1.11 |
| 74407 | -1.32 | 14469 | -1.46 | 268656 | -1.56 | 211064 | -1.11 |
| 69564 | -1.32 | 11538 | -1.46 | 19739 | -1.57 | 70394 | -1.11 |
| 243906 | -1.32 | 1.01E+08 | -1.47 | 668940 | -1.58 | 77889 | -1.11 |
| 12700 | -1.32 | 14555 | -1.48 | 22695 | -1.59 | 16400 | -1.11 |
| 109095 | -1.32 | 230678 | -1.48 | 654796 | -1.60 | 69941 | -1.11 |
| 630836 | -1.32 | 667666 | -1.48 | 226409 | -1.61 | 73490 | -1.11 |
| 328263 | -1.32 | 77975 | -1.48 | 666806 | -1.62 | 56310 | -1.11 |
| 101488 | -1.33 | 224697 | -1.48 | 69635 | -1.62 | 103511 | -1.11 |
| 11304 | -1.33 | 53880 | -1.48 | 12508 | -1.62 | 14102 | -1.12 |
| novel_G000830 | -1.33 | novel_G000520 | -1.48 | 22361 | -1.63 | 12369 | -1.12 |
| 20583 | -1.33 | 235636 | -1.48 | 667214 | -1.63 | 16803 | -1.12 |
| 11522 | -1.33 | 16803 | -1.48 | 16408 | -1.64 | 668215 | -1.13 |
| 12508 | -1.33 | 239790 | -1.49 | 71660 | -1.65 | 16470 | -1.13 |
| 634650 | -1.33 | 20344 | -1.49 | 15015 | -1.65 | 231946 | -1.13 |
| 14871 | -1.33 | 545554 | -1.49 | 210992 | -1.66 | 214106 | -1.13 |
| 56188 | -1.33 | 13809 | -1.50 | 671535 | -1.66 | 26909 | -1.13 |
| 22695 | -1.33 | 331004 | -1.50 | 56533 | -1.66 | 73420 | -1.14 |
| 20363 | -1.34 | 100604 | -1.51 | 243374 | -1.67 | 93878 | -1.14 |
| 14824 | -1.34 | 76884 | -1.51 | 17472 | -1.67 | 231474 | -1.14 |
| 13653 | -1.34 | 15203 | -1.51 | 13610 | -1.68 | 18933 | -1.14 |
| 16400 | -1.34 | 219131 | -1.51 | 56309 | -1.68 | 21354 | -1.15 |
| 73690 | -1.35 | 12266 | -1.51 | 56336 | -1.69 | 16188 | -1.15 |
| 12815 | -1.35 | 67606 | -1.52 | 17687 | -1.69 | 74407 | -1.15 |
| 232560 | -1.35 | 14281 | -1.52 | 64454 | -1.70 | 13436 | -1.15 |
| 14702 | -1.35 | 103511 | -1.52 | 12609 | -1.70 | 236930 | -1.15 |
| 73737 | -1.35 | 16428 | -1.53 | 93880 | -1.70 | 244654 | -1.16 |
| 353025 | -1.35 | 23886 | -1.53 | 1E+08 | -1.70 | 102920 | -1.16 |
| 12304 | -1.35 | 105855 | -1.53 | NM_001348222 | -1.71 | 107373 | -1.16 |
| 15511 | -1.36 | 72393 | -1.54 | 93674 | -1.71 | 379043 | -1.16 |
| 234593 | -1.37 | 16574 | -1.54 | 20715 | -1.72 | 69550 | -1.16 |
| novel_G000088 | -1.37 | 108154 | -1.54 | 20679 | -1.73 | 50927 | -1.16 |
| 210027 | -1.37 | 109032 | -1.54 | 13617 | -1.73 | 68055 | -1.16 |
| 207785 | -1.37 | 13537 | -1.55 | 140709 | -1.74 | 21683 | -1.17 |
| 20167 | -1.37 | 22695 | -1.55 | 227120 | -1.74 | 16716 | -1.17 |
| 68421 | -1.37 | 13479 | -1.55 | 68279 | -1.75 | 68526 | -1.17 |
| 16323 | -1.37 | 24066 | -1.55 | 192164 | -1.76 | 64385 | -1.17 |
| 381812 | -1.38 | 215303 | -1.55 | 21825 | -1.76 | 24066 | -1.17 |
| 338523 | -1.38 | 241528 | -1.56 | 236366 | -1.77 | 15201 | -1.17 |
| 67880 | -1.38 | 230766 | -1.56 | 16145 | -1.77 | 19650 | -1.17 |
| 110454 | -1.38 | 12223 | -1.57 | 12268 | -1.77 | 72544 | -1.18 |
| 217258 | -1.39 | 70747 | -1.57 | 232087 | -1.77 | 14972 | -1.18 |
| 12695 | -1.39 | 71586 | -1.58 | 228094 | -1.77 | 72787 | -1.18 |
| 67573 | -1.39 | 93837 | -1.59 | novel_G000517 | -1.77 | 243277 | -1.18 |
| 18037 | -1.39 | 58218 | -1.60 | 15203 | -1.78 | 66953 | -1.18 |
| 17687 | -1.39 | 12994 | -1.61 | 76074 | -1.79 | 71841 | -1.18 |
| 67138 | -1.39 | novel_G000190 | -1.61 | 224997 | -1.79 | 192970 | -1.18 |
| 14231 | -1.39 | 243277 | -1.61 | 20128 | -1.79 | 12363 | -1.18 |
| 14697 | -1.39 | 276919 | -1.62 | 21960 | -1.79 | 57138 | -1.18 |
| 228846 | -1.39 | 240913 | -1.62 | novel_G000522 | -1.79 | 17219 | -1.18 |
| 58222 | -1.40 | 268527 | -1.63 | novel_G000355 | -1.80 | 14178 | -1.18 |
| 67731 | -1.40 | 103964 | -1.63 | 100702 | -1.81 | 70300 | -1.18 |
| 72446 | -1.40 | 101320 | -1.63 | novel_G000367 | -1.82 | 77975 | -1.18 |
| 12519 | -1.40 | 11910 | -1.64 | 18260 | -1.82 | 209086 | -1.19 |
| 71981 | -1.40 | 16145 | -1.64 | 229706 | -1.83 | 268465 | -1.19 |
| 103012 | -1.40 | 15936 | -1.65 | 15896 | -1.84 | 381413 | -1.20 |
| 208117 | -1.40 | 208943 | -1.65 | 13406 | -1.85 | 75590 | -1.20 |
| 15213 | -1.41 | 23962 | -1.66 | novel_G000885 | -1.85 | 70024 | -1.20 |
| 17772 | -1.41 | 110696 | -1.66 | 103964 | -1.86 | 19366 | -1.21 |
| 70561 | -1.41 | 66107 | -1.66 | 17761 | -1.87 | 14555 | -1.21 |
| 320139 | -1.42 | 1E+08 | -1.66 | 1.01E+08 | -1.87 | 68550 | -1.21 |
| 78749 | -1.42 | 192190 | -1.66 | 235636 | -1.87 | 83815 | -1.21 |
| 26968 | -1.42 | novel_G000874 | -1.66 | 14164 | -1.88 | 243634 | -1.21 |
| 93878 | -1.42 | 73737 | -1.66 | 16477 | -1.88 | 11538 | -1.21 |
| 17063 | -1.42 | 56336 | -1.67 | 1E+08 | -1.89 | 230376 | -1.22 |
| 246727 | -1.42 | 54199 | -1.67 | 233199 | -1.90 | 11989 | -1.22 |
| 320064 | -1.42 | 67896 | -1.68 | 70127 | -1.91 | 14760 | -1.22 |
| 83965 | -1.42 | 20128 | -1.68 | 12227 | -1.91 | 626316 | -1.22 |
| 234353 | -1.42 | 13653 | -1.68 | 93838 | -1.91 | 11304 | -1.22 |
| 625167 | -1.42 | 104759 | -1.69 | 620913 | -1.92 | 226691 | -1.23 |
| 68177 | -1.42 | 20537 | -1.70 | 14282 | -1.92 | 667977 | -1.23 |
| 574428 | -1.42 | 13617 | -1.71 | 217410 | -1.92 | 12036 | -1.23 |
| 68070 | -1.43 | 319430 | -1.71 | 1E+08 | -1.92 | 58182 | -1.23 |
| 98682 | -1.43 | 214240 | -1.71 | 109979 | -1.92 | 268656 | -1.23 |
| 18783 | -1.43 | 116847 | -1.72 | 18574 | -1.93 | 78925 | -1.24 |
| 13610 | -1.43 | 78892 | -1.73 | 68632 | -1.94 | 15901 | -1.24 |
| 50723 | -1.43 | 207182 | -1.73 | 53416 | -1.95 | 12544 | -1.25 |
| 103949 | -1.43 | 212980 | -1.73 | 16428 | -1.95 | 75801 | -1.25 |
| 103948 | -1.43 | 68396 | -1.74 | 54563 | -1.95 | 13171 | -1.25 |
| 74309 | -1.43 | 24117 | -1.74 | 17972 | -1.96 | 12321 | -1.25 |
| 23886 | -1.44 | 17972 | -1.74 | 20537 | -1.96 | 12144 | -1.25 |
| 218311 | -1.44 | novel_G000188 | -1.75 | 269831 | -1.96 | 631304 | -1.25 |
| 243382 | -1.44 | novel_G000892 | -1.75 | 15559 | -1.96 | 207704 | -1.26 |
| 394432 | -1.44 | 269346 | -1.75 | 72500 | -1.96 | 67731 | -1.26 |
| 213068 | -1.44 | 53416 | -1.76 | 20556 | -1.97 | 269400 | -1.26 |
| 19085 | -1.44 | 75858 | -1.76 | 1E+08 | -1.97 | 66246 | -1.26 |
| 12527 | -1.44 | 12231 | -1.77 | novel_G000668 | -1.97 | 18105 | -1.26 |
| 107885 | -1.44 | 12288 | -1.77 | 70747 | -1.97 | 84653 | -1.26 |
| 74155 | -1.44 | 15953 | -1.77 | 14469 | -1.98 | 1.01E+08 | -1.27 |
| 102294 | -1.45 | NM_001348095 | -1.78 | 18419 | -1.99 | 213389 | -1.27 |
| 330427 | -1.45 | 21452 | -1.78 | 12051 | -2.00 | 93876 | -1.27 |
| 433470 | -1.45 | 12839 | -1.78 | 68774 | -2.00 | 18973 | -1.27 |
| novel_G000653 | -1.45 | 19281 | -1.79 | 215303 | -2.00 | 78895 | -1.27 |
| novel_G000412 | -1.45 | 58176 | -1.79 | 19241 | -2.01 | 66904 | -1.27 |
| 234199 | -1.45 | 13610 | -1.80 | 80721 | -2.02 | 12223 | -1.27 |
| 209692 | -1.45 | novel_G000031 | -1.80 | 215378 | -2.02 | 73737 | -1.27 |
| 319720 | -1.46 | 11447 | -1.81 | 79459 | -2.03 | 18140 | -1.27 |
| 227580 | -1.46 | 98303 | -1.81 | 67606 | -2.03 | 69706 | -1.28 |
| 71086 | -1.46 | 1E+08 | -1.81 | 192161 | -2.05 | 630579 | -1.28 |
| 26399 | -1.46 | 192164 | -1.81 | 16981 | -2.05 | 71743 | -1.28 |
| 20351 | -1.46 | 13164 | -1.81 | 116903 | -2.05 | 13139 | -1.28 |
| 228357 | -1.46 | 666806 | -1.82 | 94176 | -2.08 | 381066 | -1.28 |
| novel_G000301 | -1.46 | 22349 | -1.82 | 14114 | -2.08 | 107751 | -1.28 |
| 93876 | -1.46 | 14347 | -1.82 | 93878 | -2.09 | 76044 | -1.28 |
| 224697 | -1.47 | 381686 | -1.82 | 209387 | -2.10 | 1.01E+08 | -1.28 |
| 76187 | -1.47 | 71884 | -1.83 | 17390 | -2.10 | 192120 | -1.29 |
| 20540 | -1.47 | 76074 | -1.83 | 26381 | -2.11 | novel_G000058 | -1.29 |
| 75311 | -1.47 | 16011 | -1.84 | 74155 | -2.11 | 16950 | -1.29 |
| 56338 | -1.48 | 94176 | -1.84 | 18191 | -2.12 | 15213 | -1.29 |
| 18846 | -1.48 | 12227 | -1.85 | 545554 | -2.13 | 17216 | -1.30 |
| 94094 | -1.48 | 11499 | -1.85 | 546024 | -2.13 | 74155 | -1.30 |
| 319924 | -1.49 | 14051 | -1.85 | novel_G000134 | -2.14 | 13809 | -1.30 |
| 14979 | -1.49 | novel_G000833 | -1.85 | 269120 | -2.15 | 54199 | -1.30 |
| novel_G000561 | -1.49 | 210992 | -1.85 | 244698 | -2.15 | 252973 | -1.30 |
| 232086 | -1.49 | 60440 | -1.86 | 236573 | -2.15 | 13361 | -1.30 |
| 240261 | -1.49 | 74020 | -1.86 | novel_G000833 | -2.15 | 15006 | -1.31 |
| 26382 | -1.50 | 634882 | -1.87 | 21897 | -2.17 | 1E+08 | -1.31 |
| 21819 | -1.50 | 13505 | -1.87 | 619715 | -2.17 | 231842 | -1.31 |
| 14538 | -1.50 | novel_G000189 | -1.88 | 394436 | -2.18 | 229445 | -1.31 |
| 74315 | -1.50 | 74155 | -1.89 | 12839 | -2.19 | 329002 | -1.31 |
| 66734 | -1.50 | 217304 | -1.89 | 67593 | -2.19 | 17427 | -1.31 |
| 106639 | -1.50 | 244698 | -1.90 | 12515 | -2.20 | 54712 | -1.31 |
| 68126 | -1.51 | 637004 | -1.91 | 246190 | -2.20 | 72672 | -1.31 |
| 19698 | -1.51 | 50518 | -1.91 | 58218 | -2.22 | 192190 | -1.31 |
| 20562 | -1.51 | 50498 | -1.91 | 60440 | -2.24 | 224691 | -1.32 |
| 378431 | -1.51 | 20265 | -1.92 | 234673 | -2.25 | 106582 | -1.32 |
| 235527 | -1.52 | 14969 | -1.92 | 16160 | -2.25 | 231507 | -1.32 |
| 64294 | -1.52 | 16149 | -1.92 | 634650 | -2.26 | 56309 | -1.32 |
| 19152 | -1.52 | 12609 | -1.92 | 213002 | -2.27 | 218311 | -1.32 |
| 229445 | -1.52 | 1E+08 | -1.92 | 1E+08 | -2.27 | 71885 | -1.32 |
| 207819 | -1.53 | 76507 | -1.92 | 330122 | -2.27 | 19092 | -1.33 |
| 93882 | -1.53 | 327958 | -1.92 | 1E+08 | -2.28 | 60411 | -1.33 |
| 18162 | -1.54 | 208080 | -1.92 | novel_G000190 | -2.29 | 13653 | -1.34 |
| 56636 | -1.54 | 69017 | -1.92 | 15936 | -2.29 | 69885 | -1.34 |
| 214084 | -1.54 | 671535 | -1.92 | 67603 | -2.30 | 226409 | -1.34 |
| 56734 | -1.54 | 74176 | -1.93 | 20750 | -2.30 | 219151 | -1.34 |
| 192976 | -1.54 | 94090 | -1.93 | 71998 | -2.31 | 67246 | -1.35 |
| 276919 | -1.54 | 15360 | -1.93 | 114564 | -2.32 | 100604 | -1.35 |
| 320705 | -1.55 | 217843 | -1.93 | 12818 | -2.35 | 233529 | -1.35 |
| 22329 | -1.55 | novel_G000192 | -1.93 | novel_G000138 | -2.36 | novel_G000892 | -1.35 |
| 15267 | -1.55 | 71998 | -1.94 | 18606 | -2.39 | 99899 | -1.36 |
| 214854 | -1.55 | 66039 | -1.94 | novel_G000834 | -2.39 | 12361 | -1.36 |
| 67426 | -1.56 | 227059 | -1.95 | 68396 | -2.42 | 77558 | -1.36 |
| 1.01E+08 | -1.56 | novel_G000025 | -1.96 | 66107 | -2.42 | 17193 | -1.36 |
| 22029 | -1.56 | 67593 | -1.96 | 239790 | -2.46 | 216867 | -1.36 |
| 17105 | -1.56 | 192161 | -1.97 | 331004 | -2.47 | 237877 | -1.37 |
| 228858 | -1.56 | 258513 | -1.98 | 94090 | -2.48 | 108671 | -1.37 |
| 66090 | -1.56 | 13654 | -1.98 | 78771 | -2.49 | 13527 | -1.37 |
| 18295 | -1.57 | 74229 | -1.98 | 1E+08 | -2.51 | 15936 | -1.37 |
| 11727 | -1.57 | 67603 | -1.98 | 67928 | -2.52 | 19183 | -1.38 |
| 227671 | -1.57 | 229900 | -1.99 | novel_G000193 | -2.52 | 77974 | -1.38 |
| 68659 | -1.57 | 68774 | -1.99 | novel_G000449 | -2.52 | 23960 | -1.38 |
| 54200 | -1.57 | 12483 | -1.99 | 17395 | -2.53 | 69270 | -1.38 |
| 18752 | -1.58 | 16477 | -1.99 | 238393 | -2.53 | 18968 | -1.38 |
| 71145 | -1.58 | 17312 | -2.00 | 269959 | -2.55 | 213556 | -1.38 |
| 22625 | -1.58 | 17472 | -2.00 | 76998 | -2.57 | 112422 | -1.38 |
| 12363 | -1.58 | 434223 | -2.01 | novel_G000284 | -2.61 | novel_G000886 | -1.38 |
| 93838 | -1.58 | 76998 | -2.01 | 209773 | -2.66 | 214901 | -1.39 |
| 18191 | -1.58 | 16007 | -2.04 | novel_G000031 | -2.66 | 215303 | -1.39 |
| 68347 | -1.58 | 544881 | -2.04 | 20311 | -2.68 | 14468 | -1.40 |
| 245128 | -1.59 | 667214 | -2.04 | novel_G000188 | -2.68 | 11988 | -1.40 |
| 231474 | -1.59 | 238393 | -2.04 | 16193 | -2.75 | 110749 | -1.40 |
| 20741 | -1.59 | 20715 | -2.06 | 236312 | -2.77 | 12994 | -1.40 |
| 71753 | -1.59 | 15511 | -2.06 | novel_G000189 | -2.78 | NM_001348222 | -1.40 |
| 75778 | -1.60 | 269959 | -2.06 | 544881 | -2.81 | 71738 | -1.41 |
| 329735 | -1.60 | 20704 | -2.07 | 192136 | -2.84 | 384701 | -1.41 |
| 230979 | -1.60 | 19241 | -2.08 | 234515 | -2.85 | 13038 | -1.41 |
| 21899 | -1.60 | 79459 | -2.09 | 434223 | -2.91 | 320664 | -1.41 |
| 17940 | -1.60 | novel_G000885 | -2.11 | novel_G000192 | -2.95 | 23964 | -1.41 |
| 18796 | -1.60 | novel_G000134 | -2.11 | 13653 | -2.96 | 1E+08 | -1.41 |
| 20391 | -1.60 | 117167 | -2.11 | novel_G000025 | -2.96 | 214952 | -1.42 |
| 106766 | -1.60 | 14164 | -2.12 | novel_G000516 | -3.00 | 211378 | -1.42 |
| 320879 | -1.61 | 66922 | -2.12 | 224761 | -3.00 | 14373 | -1.43 |
| 11720 | -1.61 | 269854 | -2.13 | 1E+08 | -3.05 | 76282 | -1.43 |
| novel_G000340 | -1.61 | 234515 | -2.13 | 59083 | -3.11 | 14469 | -1.43 |
| 17972 | -1.61 | 22361 | -2.14 | 20704 | -3.15 | 16011 | -1.43 |
| 93885 | -1.61 | novel_G000138 | -2.15 | 11838 | -3.24 | 626904 | -1.43 |
| 93880 | -1.62 | 14114 | -2.15 | 1E+08 | -3.25 | 17217 | -1.43 |
| 1E+08 | -1.62 | 12515 | -2.16 | 27052 | -3.27 | 64685 | -1.43 |
| 242125 | -1.62 | 21960 | -2.18 | 243369 | -3.28 | 72151 | -1.43 |
| 66107 | -1.62 | 20556 | -2.19 | 266459 | -3.28 | 67373 | -1.44 |
| 21886 | -1.62 | 668940 | -2.19 | 17312 | -3.31 | 15331 | -1.44 |
| 228993 | -1.62 | 67928 | -2.19 | 16819 | -3.33 | 60345 | -1.44 |
| 244202 | -1.62 | 215243 | -2.20 | 13654 | -3.34 | 433804 | -1.45 |
| 15483 | -1.63 | novel_G000987 | -2.23 | 26464 | -3.35 | 68632 | -1.46 |
| 14725 | -1.63 | 93880 | -2.24 | 14825 | -3.52 | 1E+08 | -1.46 |
| 208820 | -1.63 | 93875 | -2.25 | 435337 | -3.54 | 78459 | -1.46 |
| 14867 | -1.64 | 12038 | -2.26 | 20310 | -3.68 | 69716 | -1.46 |
| 11775 | -1.64 | 109979 | -2.27 | 1E+08 | -3.70 | 103949 | -1.47 |
| 17951 | -1.64 | 18022 | -2.28 | novel_G000518 | -3.70 | 103948 | -1.47 |
| 54342 | -1.64 | 140709 | -2.32 | novel_G000286 | -3.73 | 71660 | -1.47 |
| 80721 | -1.64 | 71584 | -2.32 | NM_001348087 | -4.06 | 231214 | -1.47 |
| 192216 | -1.65 | 217169 | -2.32 | 20297 | -4.40 | 67733 | -1.47 |
| 73713 | -1.65 | 1E+08 | -2.36 | 20302 | -4.98 | 214968 | -1.48 |
| 103967 | -1.66 | 14067 | -2.37 |  |  | 66570 | -1.48 |
| 68526 | -1.66 | 213002 | -2.43 |  |  | 14408 | -1.48 |
| 58809 | -1.66 | 54563 | -2.43 |  |  | 17218 | -1.48 |
| 24014 | -1.66 | 229706 | -2.43 |  |  | 71206 | -1.49 |
| 15370 | -1.66 | novel_G000515 | -2.46 |  |  | 209558 | -1.49 |
| 244653 | -1.66 | 66300 | -2.48 |  |  | 93837 | -1.49 |
| 19124 | -1.66 | novel_G000193 | -2.48 |  |  | 224093 | -1.50 |
| 243277 | -1.67 | 209773 | -2.53 |  |  | 15015 | -1.50 |
| 12282 | -1.68 | 236573 | -2.53 |  |  | 18751 | -1.50 |
| 16970 | -1.68 | 114564 | -2.54 |  |  | 50723 | -1.50 |
| 22317 | -1.69 | 209387 | -2.57 |  |  | 15016 | -1.50 |
| 16803 | -1.69 | 12818 | -2.58 |  |  | 54598 | -1.50 |
| 381463 | -1.69 | 116903 | -2.58 |  |  | 66634 | -1.51 |
| 72393 | -1.70 | 64454 | -2.60 |  |  | 94094 | -1.51 |
| 80910 | -1.70 | 668039 | -2.60 |  |  | 15364 | -1.51 |
| 17540 | -1.70 | 1E+08 | -2.60 |  |  | 13982 | -1.52 |
| 12354 | -1.70 | 1E+08 | -2.60 |  |  | 217169 | -1.52 |
| 85031 | -1.71 | 12007 | -2.60 |  |  | 67138 | -1.52 |
| 19699 | -1.71 | 56533 | -2.62 |  |  | 241633 | -1.53 |
| 21844 | -1.72 | 330122 | -2.65 |  |  | 11472 | -1.53 |
| 70762 | -1.72 | 435337 | -2.68 |  |  | 330485 | -1.53 |
| 100604 | -1.72 | 1E+08 | -2.69 |  |  | 545554 | -1.53 |
| 1E+08 | -1.72 | 620913 | -2.74 |  |  | 93880 | -1.53 |
| 13654 | -1.72 | 11838 | -2.74 |  |  | 234673 | -1.54 |
| 12227 | -1.73 | 224761 | -2.78 |  |  | 17386 | -1.54 |
| 12266 | -1.73 | 16160 | -2.79 |  |  | 76429 | -1.55 |
| 432611 | -1.73 | 12051 | -2.83 |  |  | 233876 | -1.55 |
| 22371 | -1.73 | 16819 | -2.83 |  |  | 19075 | -1.56 |
| 384701 | -1.73 | 17390 | -2.83 |  |  | 106795 | -1.56 |
| 76884 | -1.73 | 266459 | -2.84 |  |  | 73998 | -1.56 |
| novel_G000579 | -1.73 | novel_G000516 | -2.85 |  |  | 11997 | -1.56 |
| 67416 | -1.73 | 59083 | -2.86 |  |  | 23834 | -1.57 |
| 12479 | -1.73 | 14825 | -2.97 |  |  | 636741 | -1.57 |
| 224079 | -1.74 | 12268 | -2.99 |  |  | 268930 | -1.57 |
| 1E+08 | -1.74 | 243369 | -3.13 |  |  | 58176 | -1.57 |
| novel_G000058 | -1.74 | 27052 | -3.15 |  |  | 81879 | -1.58 |
| 68852 | -1.75 | 20311 | -3.20 |  |  | 230678 | -1.58 |
| 56448 | -1.75 | 20310 | -3.24 |  |  | 76843 | -1.58 |
| 74481 | -1.75 | 20297 | -3.30 |  |  | 66696 | -1.58 |
| 1.01E+08 | -1.76 | 94226 | -3.37 |  |  | 1E+08 | -1.59 |
| 1E+08 | -1.76 | 26464 | -3.37 |  |  | 11642 | -1.59 |
| 12839 | -1.76 | 1E+08 | -3.42 |  |  | 72121 | -1.59 |
| 68055 | -1.76 | 18419 | -3.52 |  |  | 16408 | -1.60 |
| 626058 | -1.77 | 16193 | -4.11 |  |  | 101320 | -1.60 |
| 214111 | -1.77 | 20302 | -5.03 |  |  | 16175 | -1.61 |
| 12038 | -1.77 | 21825 | -5.18 |  |  | 94279 | -1.61 |
| 240667 | -1.77 | novel_G000519 | -5.28 |  |  | 12862 | -1.61 |
| 381339 | -1.78 |  |  |  |  | 56336 | -1.61 |
| 212980 | -1.78 |  |  |  |  | 16425 | -1.62 |
| 396184 | -1.78 |  |  |  |  | 71998 | -1.63 |
| 238871 | -1.78 |  |  |  |  | 18022 | -1.63 |
| 67475 | -1.79 |  |  |  |  | 20215 | -1.63 |
| 67603 | -1.79 |  |  |  |  | 13655 | -1.63 |
| 69635 | -1.79 |  |  |  |  | 67416 | -1.63 |
| 93875 | -1.79 |  |  |  |  | 78892 | -1.63 |
| 214968 | -1.80 |  |  |  |  | 21853 | -1.63 |
| 74761 | -1.80 |  |  |  |  | 109979 | -1.64 |
| 74153 | -1.80 |  |  |  |  | 67775 | -1.64 |
| 381229 | -1.80 |  |  |  |  | 17067 | -1.64 |
| 239559 | -1.81 |  |  |  |  | 208943 | -1.64 |
| 72361 | -1.81 |  |  |  |  | 19085 | -1.64 |
| 13982 | -1.81 |  |  |  |  | 18703 | -1.65 |
| 68339 | -1.82 |  |  |  |  | 68023 | -1.65 |
| 236366 | -1.82 |  |  |  |  | novel_G000451 | -1.65 |
| 110891 | -1.82 |  |  |  |  | 219132 | -1.66 |
| 12349 | -1.83 |  |  |  |  | 14934 | -1.66 |
| 634882 | -1.83 |  |  |  |  | 668940 | -1.66 |
| 213980 | -1.83 |  |  |  |  | 76088 | -1.67 |
| 15936 | -1.84 |  |  |  |  | 13479 | -1.67 |
| novel_G000183 | -1.84 |  |  |  |  | 17972 | -1.67 |
| 16574 | -1.84 |  |  |  |  | 71584 | -1.67 |
| 75568 | -1.84 |  |  |  |  | 16912 | -1.67 |
| 70902 | -1.85 |  |  |  |  | 69635 | -1.68 |
| 1E+08 | -1.85 |  |  |  |  | 11838 | -1.68 |
| 78771 | -1.85 |  |  |  |  | 620913 | -1.68 |
| 18213 | -1.85 |  |  |  |  | 80721 | -1.68 |
| 20210 | -1.85 |  |  |  |  | 667666 | -1.68 |
| 53603 | -1.85 |  |  |  |  | 72107 | -1.69 |
| 27404 | -1.85 |  |  |  |  | 68026 | -1.69 |
| 230787 | -1.86 |  |  |  |  | 20679 | -1.69 |
| 227696 | -1.86 |  |  |  |  | 117167 | -1.69 |
| 11302 | -1.86 |  |  |  |  | 667214 | -1.70 |
| 625530 | -1.86 |  |  |  |  | 236366 | -1.70 |
| 20128 | -1.86 |  |  |  |  | 93838 | -1.70 |
| 105727 | -1.87 |  |  |  |  | 67434 | -1.71 |
| 106861 | -1.87 |  |  |  |  | 18108 | -1.71 |
| 619326 | -1.88 |  |  |  |  | novel_G000561 | -1.72 |
| 17386 | -1.88 |  |  |  |  | 93875 | -1.72 |
| 16477 | -1.88 |  |  |  |  | 22361 | -1.72 |
| 74129 | -1.88 |  |  |  |  | 72361 | -1.73 |
| 271375 | -1.89 |  |  |  |  | 66107 | -1.73 |
| 271424 | -1.89 |  |  |  |  | 55932 | -1.73 |
| 209558 | -1.90 |  |  |  |  | 269120 | -1.73 |
| 71345 | -1.90 |  |  |  |  | 69544 | -1.74 |
| 75292 | -1.90 |  |  |  |  | 21926 | -1.74 |
| 230766 | -1.90 |  |  |  |  | 210992 | -1.74 |
| 217843 | -1.90 |  |  |  |  | novel_G000774 | -1.75 |
| 116838 | -1.90 |  |  |  |  | 14585 | -1.75 |
| 271697 | -1.91 |  |  |  |  | 12508 | -1.77 |
| 66857 | -1.91 |  |  |  |  | 26434 | -1.77 |
| 215512 | -1.91 |  |  |  |  | 24117 | -1.78 |
| 16193 | -1.92 |  |  |  |  | 69865 | -1.78 |
| novel_G000834 | -1.92 |  |  |  |  | 14807 | -1.79 |
| 353234 | -1.92 |  |  |  |  | 20556 | -1.79 |
| 19817 | -1.92 |  |  |  |  | 11720 | -1.79 |
| 233199 | -1.92 |  |  |  |  | 15039 | -1.79 |
| 1.01E+08 | -1.92 |  |  |  |  | 1.01E+08 | -1.79 |
| 629141 | -1.92 |  |  |  |  | 18574 | -1.79 |
| 17390 | -1.92 |  |  |  |  | 13434 | -1.79 |
| 103406 | -1.93 |  |  |  |  | 71872 | -1.80 |
| 18616 | -1.93 |  |  |  |  | 13406 | -1.80 |
| 12490 | -1.93 |  |  |  |  | novel_G000167 | -1.80 |
| 269589 | -1.93 |  |  |  |  | 229949 | -1.81 |
| 12943 | -1.93 |  |  |  |  | 109032 | -1.81 |
| 11910 | -1.93 |  |  |  |  | novel_G000686 | -1.81 |
| 20190 | -1.94 |  |  |  |  | novel_G000367 | -1.81 |
| 16164 | -1.94 |  |  |  |  | 243374 | -1.82 |
| 268902 | -1.94 |  |  |  |  | 1E+08 | -1.82 |
| 229722 | -1.95 |  |  |  |  | 116838 | -1.83 |
| 93671 | -1.95 |  |  |  |  | 634650 | -1.83 |
| 381538 | -1.95 |  |  |  |  | 13610 | -1.83 |
| 14969 | -1.95 |  |  |  |  | 353234 | -1.84 |
| novel_G000879 | -1.95 |  |  |  |  | 235180 | -1.84 |
| 18011 | -1.95 |  |  |  |  | 210530 | -1.85 |
| 19073 | -1.95 |  |  |  |  | 81799 | -1.85 |
| 217517 | -1.95 |  |  |  |  | 13110 | -1.86 |
| 18197 | -1.95 |  |  |  |  | 634882 | -1.86 |
| 66696 | -1.96 |  |  |  |  | 19737 | -1.86 |
| 242408 | -1.96 |  |  |  |  | 235636 | -1.87 |
| 74116 | -1.96 |  |  |  |  | 18705 | -1.88 |
| 72281 | -1.96 |  |  |  |  | 17380 | -1.88 |
| 12483 | -1.96 |  |  |  |  | 78249 | -1.89 |
| 16181 | -1.96 |  |  |  |  | 207921 | -1.89 |
| 26366 | -1.96 |  |  |  |  | 619326 | -1.90 |
| 12496 | -1.96 |  |  |  |  | novel_G000542 | -1.90 |
| 208666 | -1.96 |  |  |  |  | 13505 | -1.90 |
| 13531 | -1.97 |  |  |  |  | 14164 | -1.90 |
| 57911 | -1.97 |  |  |  |  | 18591 | -1.90 |
| 231805 | -1.97 |  |  |  |  | 637004 | -1.91 |
| 26434 | -1.97 |  |  |  |  | 12895 | -1.91 |
| 74008 | -1.98 |  |  |  |  | 207181 | -1.91 |
| 243725 | -1.98 |  |  |  |  | 12051 | -1.92 |
| 71213 | -1.99 |  |  |  |  | 110696 | -1.92 |
| 20556 | -1.99 |  |  |  |  | 72500 | -1.93 |
| 14675 | -1.99 |  |  |  |  | 12515 | -1.93 |
| 545554 | -1.99 |  |  |  |  | 217410 | -1.93 |
| 15064 | -1.99 |  |  |  |  | 98932 | -1.94 |
| 17060 | -1.99 |  |  |  |  | 239849 | -1.94 |
| 12223 | -2.00 |  |  |  |  | 110454 | -1.94 |
| 12509 | -2.00 |  |  |  |  | 78771 | -1.95 |
| 18574 | -2.01 |  |  |  |  | 12661 | -1.96 |
| 330409 | -2.01 |  |  |  |  | 56375 | -1.96 |
| 12609 | -2.01 |  |  |  |  | 208869 | -1.96 |
| 215303 | -2.01 |  |  |  |  | 434218 | -1.97 |
| 93711 | -2.01 |  |  |  |  | 233801 | -1.97 |
| 74419 | -2.01 |  |  |  |  | 74116 | -1.97 |
| 327978 | -2.02 |  |  |  |  | 626578 | -1.97 |
| 14411 | -2.02 |  |  |  |  | 224997 | -1.99 |
| 433944 | -2.02 |  |  |  |  | 545654 | -2.00 |
| 98303 | -2.02 |  |  |  |  | 79459 | -2.00 |
| 224997 | -2.02 |  |  |  |  | 74519 | -2.00 |
| 79459 | -2.03 |  |  |  |  | 245126 | -2.01 |
| 17329 | -2.03 |  |  |  |  | 94176 | -2.01 |
| 116847 | -2.03 |  |  |  |  | 215243 | -2.01 |
| 379043 | -2.04 |  |  |  |  | 217843 | -2.01 |
| 545902 | -2.04 |  |  |  |  | novel_G000290 | -2.01 |
| 224044 | -2.04 |  |  |  |  | 140709 | -2.02 |
| 20511 | -2.04 |  |  |  |  | 13617 | -2.02 |
| 52389 | -2.04 |  |  |  |  | 100702 | -2.02 |
| 13617 | -2.04 |  |  |  |  | 11910 | -2.02 |
| 140493 | -2.04 |  |  |  |  | 192164 | -2.03 |
| 21940 | -2.05 |  |  |  |  | 233079 | -2.04 |
| 208943 | -2.05 |  |  |  |  | 232087 | -2.04 |
| 17068 | -2.05 |  |  |  |  | 272009 | -2.05 |
| 75784 | -2.05 |  |  |  |  | 16145 | -2.06 |
| 73075 | -2.05 |  |  |  |  | 619715 | -2.06 |
| 320664 | -2.05 |  |  |  |  | 12609 | -2.07 |
| 13139 | -2.05 |  |  |  |  | 12686 | -2.07 |
| 1E+08 | -2.06 |  |  |  |  | 16160 | -2.07 |
| 240913 | -2.06 |  |  |  |  | 68396 | -2.08 |
| 58229 | -2.06 |  |  |  |  | novel_G000411 | -2.08 |
| 21859 | -2.06 |  |  |  |  | 67216 | -2.08 |
| 24050 | -2.06 |  |  |  |  | 18419 | -2.09 |
| 74002 | -2.07 |  |  |  |  | 1E+08 | -2.09 |
| 68632 | -2.07 |  |  |  |  | 319487 | -2.10 |
| 52882 | -2.07 |  |  |  |  | 240063 | -2.10 |
| 114249 | -2.07 |  |  |  |  | 234515 | -2.10 |
| 13527 | -2.08 |  |  |  |  | 1E+08 | -2.14 |
| 107751 | -2.08 |  |  |  |  | 243881 | -2.14 |
| 16426 | -2.08 |  |  |  |  | novel_G000502 | -2.14 |
| 319823 | -2.08 |  |  |  |  | 17472 | -2.14 |
| 17912 | -2.08 |  |  |  |  | novel_G000518 | -2.14 |
| novel_G000190 | -2.09 |  |  |  |  | 20558 | -2.15 |
| 319713 | -2.09 |  |  |  |  | 17390 | -2.15 |
| 104816 | -2.10 |  |  |  |  | 106877 | -2.16 |
| 68764 | -2.10 |  |  |  |  | 16428 | -2.17 |
| 71998 | -2.10 |  |  |  |  | 54563 | -2.18 |
| 18703 | -2.10 |  |  |  |  | 67603 | -2.18 |
| 319981 | -2.10 |  |  |  |  | 1.05E+08 | -2.19 |
| 13479 | -2.10 |  |  |  |  | 666060 | -2.19 |
| 14051 | -2.11 |  |  |  |  | 1E+08 | -2.20 |
| 381738 | -2.11 |  |  |  |  | 70747 | -2.20 |
| 192164 | -2.11 |  |  |  |  | 17395 | -2.20 |
| 671535 | -2.11 |  |  |  |  | novel_G000673 | -2.21 |
| 58208 | -2.11 |  |  |  |  | 229900 | -2.22 |
| 545156 | -2.11 |  |  |  |  | 258513 | -2.22 |
| 11899 | -2.12 |  |  |  |  | 244698 | -2.22 |
| 16847 | -2.12 |  |  |  |  | 71724 | -2.24 |
| 71740 | -2.12 |  |  |  |  | 18606 | -2.26 |
| 64454 | -2.12 |  |  |  |  | 93711 | -2.26 |
| 215707 | -2.12 |  |  |  |  | 1E+08 | -2.27 |
| 246049 | -2.12 |  |  |  |  | 116903 | -2.27 |
| 20355 | -2.12 |  |  |  |  | 108672 | -2.29 |
| 29809 | -2.13 |  |  |  |  | 12007 | -2.29 |
| 242607 | -2.13 |  |  |  |  | 72560 | -2.30 |
| 67606 | -2.13 |  |  |  |  | 19241 | -2.31 |
| novel_G000367 | -2.13 |  |  |  |  | 15203 | -2.33 |
| novel_G000878 | -2.13 |  |  |  |  | 20750 | -2.34 |
| 15203 | -2.14 |  |  |  |  | 16981 | -2.35 |
| 272009 | -2.14 |  |  |  |  | 56533 | -2.35 |
| 207596 | -2.15 |  |  |  |  | 192167 | -2.37 |
| 18606 | -2.15 |  |  |  |  | novel_G000833 | -2.38 |
| 17312 | -2.15 |  |  |  |  | 16970 | -2.38 |
| 244562 | -2.15 |  |  |  |  | 20344 | -2.38 |
| 76718 | -2.15 |  |  |  |  | 14114 | -2.38 |
| 327958 | -2.17 |  |  |  |  | 671535 | -2.38 |
| 26464 | -2.17 |  |  |  |  | 654796 | -2.39 |
| 15567 | -2.17 |  |  |  |  | novel_G000522 | -2.39 |
| 14469 | -2.17 |  |  |  |  | 20537 | -2.40 |
| 68922 | -2.18 |  |  |  |  | 20128 | -2.43 |
| 15162 | -2.18 |  |  |  |  | 20715 | -2.45 |
| novel_G000188 | -2.18 |  |  |  |  | 269959 | -2.46 |
| 21960 | -2.18 |  |  |  |  | 68774 | -2.46 |
| 22349 | -2.18 |  |  |  |  | 60440 | -2.47 |
| 71263 | -2.18 |  |  |  |  | 71026 | -2.47 |
| 654459 | -2.19 |  |  |  |  | 224796 | -2.48 |
| 216198 | -2.19 |  |  |  |  | 66922 | -2.51 |
| 83767 | -2.19 |  |  |  |  | 233199 | -2.51 |
| 1E+08 | -2.19 |  |  |  |  | 53416 | -2.51 |
| 13842 | -2.20 |  |  |  |  | 12268 | -2.52 |
| 269120 | -2.20 |  |  |  |  | 219131 | -2.52 |
| 233529 | -2.20 |  |  |  |  | novel_G000834 | -2.54 |
| 20344 | -2.20 |  |  |  |  | 12038 | -2.55 |
| 107227 | -2.21 |  |  |  |  | 75292 | -2.55 |
| 1E+08 | -2.21 |  |  |  |  | 67928 | -2.55 |
| novel_G000134 | -2.21 |  |  |  |  | 12839 | -2.58 |
| 224129 | -2.21 |  |  |  |  | 74469 | -2.63 |
| 13406 | -2.21 |  |  |  |  | 1E+08 | -2.63 |
| 21941 | -2.22 |  |  |  |  | 236451 | -2.68 |
| 74176 | -2.22 |  |  |  |  | 619289 | -2.68 |
| 14468 | -2.22 |  |  |  |  | 76074 | -2.69 |
| 15360 | -2.22 |  |  |  |  | 58218 | -2.69 |
| novel_G000411 | -2.24 |  |  |  |  | 246190 | -2.70 |
| 68728 | -2.24 |  |  |  |  | 209773 | -2.73 |
| 18008 | -2.24 |  |  |  |  | 236312 | -2.74 |
| 224796 | -2.24 |  |  |  |  | novel_G000138 | -2.76 |
| 666806 | -2.24 |  |  |  |  | 236573 | -2.82 |
| 11997 | -2.24 |  |  |  |  | 239790 | -2.87 |
| 235636 | -2.25 |  |  |  |  | novel_G000383 | -2.88 |
| 269831 | -2.25 |  |  |  |  | 22138 | -2.89 |
| 103511 | -2.26 |  |  |  |  | 330122 | -2.90 |
| 1E+08 | -2.26 |  |  |  |  | 666806 | -2.92 |
| 108154 | -2.26 |  |  |  |  | 26381 | -2.93 |
| 20537 | -2.27 |  |  |  |  | 331004 | -2.98 |
| 320360 | -2.27 |  |  |  |  | novel_G000031 | -2.98 |
| 12877 | -2.27 |  |  |  |  | 103964 | -2.99 |
| 70127 | -2.28 |  |  |  |  | 209387 | -3.01 |
| 12515 | -2.28 |  |  |  |  | 224761 | -3.03 |
| 619289 | -2.28 |  |  |  |  | 21897 | -3.07 |
| 12182 | -2.28 |  |  |  |  | 238393 | -3.12 |
| 17395 | -2.28 |  |  |  |  | 94090 | -3.12 |
| 18705 | -2.29 |  |  |  |  | 192161 | -3.18 |
| 67896 | -2.29 |  |  |  |  | 70809 | -3.21 |
| 16145 | -2.29 |  |  |  |  | 76998 | -3.21 |
| 68127 | -2.29 |  |  |  |  | novel_G000193 | -3.23 |
| 214240 | -2.29 |  |  |  |  | novel_G000885 | -3.26 |
| 1.01E+08 | -2.30 |  |  |  |  | 114564 | -3.26 |
| novel_G000890 | -2.30 |  |  |  |  | 269854 | -3.27 |
| 224093 | -2.31 |  |  |  |  | 1E+08 | -3.34 |
| 50722 | -2.31 |  |  |  |  | 11447 | -3.34 |
| novel_G000192 | -2.31 |  |  |  |  | 27052 | -3.37 |
| 56226 | -2.31 |  |  |  |  | 1E+08 | -3.46 |
| 14281 | -2.32 |  |  |  |  | 74020 | -3.47 |
| 20265 | -2.32 |  |  |  |  | novel_G000189 | -3.47 |
| 56533 | -2.33 |  |  |  |  | novel_G000192 | -3.49 |
| 207181 | -2.33 |  |  |  |  | 18191 | -3.50 |
| 667214 | -2.33 |  |  |  |  | novel_G000190 | -3.51 |
| 435337 | -2.33 |  |  |  |  | novel_G000030 | -3.53 |
| 64385 | -2.33 |  |  |  |  | 434223 | -3.55 |
| 66599 | -2.33 |  |  |  |  | 544881 | -3.56 |
| 67216 | -2.34 |  |  |  |  | novel_G000188 | -3.60 |
| 19065 | -2.34 |  |  |  |  | 12818 | -3.64 |
| 94090 | -2.35 |  |  |  |  | 16193 | -3.69 |
| 15945 | -2.35 |  |  |  |  | 20310 | -3.69 |
| 77974 | -2.35 |  |  |  |  | 20704 | -3.72 |
| 266459 | -2.36 |  |  |  |  | 14825 | -3.72 |
| 636741 | -2.36 |  |  |  |  | 67606 | -3.74 |
| 74090 | -2.37 |  |  |  |  | 59083 | -3.75 |
| 11447 | -2.38 |  |  |  |  | 20311 | -3.78 |
| 16516 | -2.39 |  |  |  |  | 213002 | -3.83 |
| 27052 | -2.39 |  |  |  |  | 17312 | -3.86 |
| 108096 | -2.39 |  |  |  |  | 26464 | -3.95 |
| 192113 | -2.40 |  |  |  |  | 243369 | -3.99 |
| 53973 | -2.41 |  |  |  |  | 1E+08 | -4.03 |
| 81879 | -2.41 |  |  |  |  | 16819 | -4.08 |
| 60504 | -2.41 |  |  |  |  | 21825 | -4.09 |
| 74229 | -2.41 |  |  |  |  | novel_G000515 | -4.15 |
| 245126 | -2.41 |  |  |  |  | 266459 | -4.16 |
| 14985 | -2.42 |  |  |  |  | 381546 | -4.26 |
| novel_G000189 | -2.42 |  |  |  |  | 20297 | -4.32 |
| 71884 | -2.42 |  |  |  |  | 435337 | -4.49 |
| 12258 | -2.43 |  |  |  |  | 1E+08 | -4.50 |
| 381113 | -2.44 |  |  |  |  | 66812 | -5.07 |
| 70747 | -2.44 |  |  |  |  | 20302 | -5.60 |
| 69066 | -2.45 |  |  |  |  |  |  |
| novel_G000518 | -2.46 |  |  |  |  |  |  |
| 22355 | -2.46 |  |  |  |  |  |  |
| 192163 | -2.46 |  |  |  |  |  |  |
| 234673 | -2.47 |  |  |  |  |  |  |
| 58176 | -2.47 |  |  |  |  |  |  |
| 213556 | -2.48 |  |  |  |  |  |  |
| novel_G000885 | -2.48 |  |  |  |  |  |  |
| 207182 | -2.48 |  |  |  |  |  |  |
| novel_G000522 | -2.48 |  |  |  |  |  |  |
| 93734 | -2.50 |  |  |  |  |  |  |
| 54139 | -2.50 |  |  |  |  |  |  |
| 258513 | -2.52 |  |  |  |  |  |  |
| 50934 | -2.52 |  |  |  |  |  |  |
| 353235 | -2.53 |  |  |  |  |  |  |
| 12268 | -2.55 |  |  |  |  |  |  |
| 544881 | -2.55 |  |  |  |  |  |  |
| 67547 | -2.56 |  |  |  |  |  |  |
| 104086 | -2.56 |  |  |  |  |  |  |
| 236312 | -2.56 |  |  |  |  |  |  |
| 217169 | -2.56 |  |  |  |  |  |  |
| 16531 | -2.56 |  |  |  |  |  |  |
| 11484 | -2.57 |  |  |  |  |  |  |
| 67593 | -2.57 |  |  |  |  |  |  |
| 12994 | -2.58 |  |  |  |  |  |  |
| 767815 | -2.58 |  |  |  |  |  |  |
| 435626 | -2.59 |  |  |  |  |  |  |
| 76507 | -2.59 |  |  |  |  |  |  |
| 77521 | -2.59 |  |  |  |  |  |  |
| 11668 | -2.59 |  |  |  |  |  |  |
| 20449 | -2.59 |  |  |  |  |  |  |
| 1.01E+08 | -2.59 |  |  |  |  |  |  |
| 18671 | -2.60 |  |  |  |  |  |  |
| 269346 | -2.60 |  |  |  |  |  |  |
| 71724 | -2.61 |  |  |  |  |  |  |
| 269959 | -2.61 |  |  |  |  |  |  |
| 14164 | -2.61 |  |  |  |  |  |  |
| novel_G000193 | -2.62 |  |  |  |  |  |  |
| 1E+08 | -2.62 |  |  |  |  |  |  |
| novel_G000774 | -2.62 |  |  |  |  |  |  |
| 331004 | -2.63 |  |  |  |  |  |  |
| 56375 | -2.64 |  |  |  |  |  |  |
| novel_G000515 | -2.64 |  |  |  |  |  |  |
| 227120 | -2.64 |  |  |  |  |  |  |
| 11838 | -2.65 |  |  |  |  |  |  |
| 14067 | -2.65 |  |  |  |  |  |  |
| 241556 | -2.66 |  |  |  |  |  |  |
| 13164 | -2.66 |  |  |  |  |  |  |
| 75858 | -2.66 |  |  |  |  |  |  |
| 20215 | -2.67 |  |  |  |  |  |  |
| 53416 | -2.67 |  |  |  |  |  |  |
| 233801 | -2.67 |  |  |  |  |  |  |
| 22361 | -2.68 |  |  |  |  |  |  |
| 16425 | -2.68 |  |  |  |  |  |  |
| 238393 | -2.68 |  |  |  |  |  |  |
| 11642 | -2.69 |  |  |  |  |  |  |
| 16819 | -2.71 |  |  |  |  |  |  |
| 192161 | -2.71 |  |  |  |  |  |  |
| 17472 | -2.75 |  |  |  |  |  |  |
| 229706 | -2.75 |  |  |  |  |  |  |
| 105859 | -2.76 |  |  |  |  |  |  |
| 12291 | -2.76 |  |  |  |  |  |  |
| 15043 | -2.77 |  |  |  |  |  |  |
| 21897 | -2.77 |  |  |  |  |  |  |
| 76074 | -2.77 |  |  |  |  |  |  |
| 18022 | -2.77 |  |  |  |  |  |  |
| 12824 | -2.77 |  |  |  |  |  |  |
| 268527 | -2.78 |  |  |  |  |  |  |
| 16011 | -2.79 |  |  |  |  |  |  |
| 114142 | -2.79 |  |  |  |  |  |  |
| 381411 | -2.79 |  |  |  |  |  |  |
| 66922 | -2.80 |  |  |  |  |  |  |
| 239790 | -2.80 |  |  |  |  |  |  |
| 20704 | -2.81 |  |  |  |  |  |  |
| 1E+08 | -2.81 |  |  |  |  |  |  |
| 329152 | -2.83 |  |  |  |  |  |  |
| 223648 | -2.83 |  |  |  |  |  |  |
| 54199 | -2.83 |  |  |  |  |  |  |
| 60345 | -2.84 |  |  |  |  |  |  |
| 140709 | -2.85 |  |  |  |  |  |  |
| 101320 | -2.90 |  |  |  |  |  |  |
| 71584 | -2.92 |  |  |  |  |  |  |
| 54563 | -2.92 |  |  |  |  |  |  |
| novel_G000031 | -2.92 |  |  |  |  |  |  |
| 14585 | -2.92 |  |  |  |  |  |  |
| 224344 | -2.93 |  |  |  |  |  |  |
| 68800 | -2.95 |  |  |  |  |  |  |
| 109032 | -2.95 |  |  |  |  |  |  |
| 66300 | -2.96 |  |  |  |  |  |  |
| 116903 | -2.96 |  |  |  |  |  |  |
| 236573 | -2.97 |  |  |  |  |  |  |
| 209773 | -2.97 |  |  |  |  |  |  |
| 114564 | -2.98 |  |  |  |  |  |  |
| 330122 | -2.98 |  |  |  |  |  |  |
| 13120 | -2.98 |  |  |  |  |  |  |
| 209387 | -2.99 |  |  |  |  |  |  |
| 16007 | -3.00 |  |  |  |  |  |  |
| 12818 | -3.00 |  |  |  |  |  |  |
| 14102 | -3.00 |  |  |  |  |  |  |
| 21683 | -3.00 |  |  |  |  |  |  |
| 58218 | -3.01 |  |  |  |  |  |  |
| 20715 | -3.02 |  |  |  |  |  |  |
| 620913 | -3.02 |  |  |  |  |  |  |
| 19739 | -3.03 |  |  |  |  |  |  |
| 320975 | -3.04 |  |  |  |  |  |  |
| 1E+08 | -3.07 |  |  |  |  |  |  |
| 16160 | -3.07 |  |  |  |  |  |  |
| 243634 | -3.08 |  |  |  |  |  |  |
| 654796 | -3.08 |  |  |  |  |  |  |
| 235180 | -3.09 |  |  |  |  |  |  |
| 208869 | -3.10 |  |  |  |  |  |  |
| 21452 | -3.11 |  |  |  |  |  |  |
| novel_G000516 | -3.11 |  |  |  |  |  |  |
| 215243 | -3.13 |  |  |  |  |  |  |
| 1E+08 | -3.15 |  |  |  |  |  |  |
| 110696 | -3.16 |  |  |  |  |  |  |
| 16149 | -3.18 |  |  |  |  |  |  |
| novel_G000138 | -3.20 |  |  |  |  |  |  |
| 108672 | -3.22 |  |  |  |  |  |  |
| 75801 | -3.22 |  |  |  |  |  |  |
| 109979 | -3.23 |  |  |  |  |  |  |
| 68774 | -3.24 |  |  |  |  |  |  |
| 74020 | -3.25 |  |  |  |  |  |  |
| 192136 | -3.28 |  |  |  |  |  |  |
| novel_G000167 | -3.29 |  |  |  |  |  |  |
| 76998 | -3.29 |  |  |  |  |  |  |
| 13505 | -3.29 |  |  |  |  |  |  |
| 103964 | -3.29 |  |  |  |  |  |  |
| 244698 | -3.32 |  |  |  |  |  |  |
| 93837 | -3.33 |  |  |  |  |  |  |
| 104759 | -3.34 |  |  |  |  |  |  |
| 109676 | -3.36 |  |  |  |  |  |  |
| 241528 | -3.36 |  |  |  |  |  |  |
| 246190 | -3.38 |  |  |  |  |  |  |
| novel_G000874 | -3.39 |  |  |  |  |  |  |
| 12361 | -3.41 |  |  |  |  |  |  |
| 12231 | -3.41 |  |  |  |  |  |  |
| 68396 | -3.45 |  |  |  |  |  |  |
| 71738 | -3.48 |  |  |  |  |  |  |
| 434223 | -3.48 |  |  |  |  |  |  |
| 67928 | -3.50 |  |  |  |  |  |  |
| 12007 | -3.55 |  |  |  |  |  |  |
| 14825 | -3.60 |  |  |  |  |  |  |
| 93732 | -3.61 |  |  |  |  |  |  |
| 74469 | -3.64 |  |  |  |  |  |  |
| 59083 | -3.65 |  |  |  |  |  |  |
| 224761 | -3.72 |  |  |  |  |  |  |
| 1E+08 | -3.75 |  |  |  |  |  |  |
| 94176 | -3.78 |  |  |  |  |  |  |
| 234515 | -3.79 |  |  |  |  |  |  |
| 26381 | -3.82 |  |  |  |  |  |  |
| 19281 | -3.83 |  |  |  |  |  |  |
| 23964 | -3.84 |  |  |  |  |  |  |
| 20311 | -3.85 |  |  |  |  |  |  |
| novel_G000025 | -3.86 |  |  |  |  |  |  |
| 213002 | -3.96 |  |  |  |  |  |  |
| 192190 | -4.03 |  |  |  |  |  |  |
| 668940 | -4.09 |  |  |  |  |  |  |
| novel_G000944 | -4.12 |  |  |  |  |  |  |
| 18419 | -4.22 |  |  |  |  |  |  |
| 20310 | -4.26 |  |  |  |  |  |  |
| novel_G000833 | -4.34 |  |  |  |  |  |  |
| 73998 | -4.50 |  |  |  |  |  |  |
| 16428 | -4.52 |  |  |  |  |  |  |
| 637004 | -4.61 |  |  |  |  |  |  |
| 243369 | -5.04 |  |  |  |  |  |  |
| NM_001348205 | -5.05 |  |  |  |  |  |  |
| 20297 | -5.12 |  |  |  |  |  |  |
| 117167 | -5.32 |  |  |  |  |  |  |
| 20302 | -5.39 |  |  |  |  |  |  |
| novel_G000030 | -5.70 |  |  |  |  |  |  |
| novel_G000519 | -5.71 |  |  |  |  |  |  |
| novel_G000835 | -6.14 |  |  |  |  |  |  |

**Supplementary Table S3:** Differentially expressed proteins in L929 cells treated with 200μM Ni^2+^ for different durations.

| **200μM-12h** | | **200μM-24h** | | **200μM-48h** | |
| --- | --- | --- | --- | --- | --- |
| UniProt Accession | Fold change | UniProt Accession | Fold change | UniProt Accession | Fold change |
| P11087 | 1.8871 | Q3URT2 | 2.9344 | B7ZWC0 | 1.7707 |
| P18828 | 1.8045 | P18828 | 1.6991 | O88207 | 1.3580 |
| Q9DCJ5 | 1.7201 | Q52L49 | 1.5271 | P11087 | 1.3004 |
| Q9CQL6 | 1.6955 | G5E898 | 1.5145 | P02463 | 1.2491 |
| Q91YE3 | 1.6326 | P02463 | 1.3862 | P17563 | 1.2330 |
| O88207 | 1.6140 | Q3UHZ3 | 1.3107 | Q8R180 | 1.2295 |
| P02463 | 1.6106 | G3X8X7 | 1.2857 | P09411 | 0.8294 |
| B7ZWC0 | 1.5859 | Q91X01 | 1.2667 | P08122 | 0.8293 |
| E9QPX1 | 1.5796 | Q3UXJ3 | 1.2043 | P05064 | 0.8283 |
| P07724 | 1.5393 | Q6GQT9 | 0.8297 | Q99LF5 | 0.8127 |
| P17809 | 1.5192 | Q810A7 | 0.8289 | Q3UGP0 | 0.8114 |
| Q8BVG3 | 1.4908 | D3Z7P2 | 0.8285 | Q8K009 | 0.8097 |
| P11157 | 1.4558 | E9Q5B5 | 0.8247 | E9PVX6 | 0.7953 |
| A2A4Z1 | 1.4378 | P58742 | 0.8205 | E9Q5B5 | 0.7951 |
| Q8BG60 | 1.3871 | Q3UJX2 | 0.8166 | O88986 | 0.7936 |
| Q3UXM3 | 1.3368 | D3YUK4 | 0.8139 | P28650 | 0.7885 |
| P17563 | 1.3304 | Q3TIX9 | 0.8113 | P52480 | 0.7885 |
| A2AEB5 | 1.3290 | P28650 | 0.8100 | P17182 | 0.7849 |
| Q9D6J9 | 1.3272 | P05064 | 0.8083 | Q9DBJ1 | 0.7812 |
| Q8BNF0 | 1.3084 | Q9CYG7 | 0.8079 | Q8CEE7 | 0.7727 |
| Q3UIS6 | 1.2313 | Q3UJ44 | 0.8076 | Q9DBG5 | 0.7698 |
| P08122 | 1.2240 | Q3U935 | 0.8053 | P16675 | 0.7697 |
| Q8K039 | 1.2051 | Q921M3 | 0.8012 | Q9Z1E4 | 0.7694 |
| Q61941 | 0.8332 | P62983 | 0.7997 | P17751 | 0.7662 |
| Q8BFY9 | 0.8327 | Q91WD5 | 0.7994 | Q91VC9 | 0.7652 |
| E9Q555 | 0.8321 | Q62418 | 0.7983 | P24668 | 0.7621 |
| Q14DR9 | 0.8293 | Q99JI6 | 0.7951 | Q8BNF0 | 0.7595 |
| Q8K0C4 | 0.8289 | P68373 | 0.7943 | O09172 | 0.7575 |
| Q5SWD9 | 0.8287 | Q8VED9 | 0.7942 | A0PJ57 | 0.7564 |
| A0A087WQE6 | 0.8267 | Q8CCJ3 | 0.7925 | P62983 | 0.7548 |
| Q8BP47 | 0.8242 | P40336 | 0.7924 | Q8K039 | 0.7529 |
| E9PWQ3 | 0.8203 | P20152 | 0.7899 | Q06EZ3 | 0.7525 |
| Q9CQV4 | 0.8196 | Q8BRF7 | 0.7886 | Q6ZQH4 | 0.7522 |
| Q3US29 | 0.8183 | H3BKN0 | 0.7886 | Q3TW01 | 0.7487 |
| P00375 | 0.8170 | P34884 | 0.7845 | P34884 | 0.7477 |
| Q9D7S7 | 0.8153 | Q64337 | 0.7842 | P11438 | 0.7473 |
| Q9CQB4 | 0.8131 | P25206 | 0.7840 | P05063 | 0.7421 |
| Q80W68 | 0.8087 | A2AL12 | 0.7832 | F6SFF5 | 0.7413 |
| O88477 | 0.8070 | Q3U878 | 0.7824 | Q3UAS4 | 0.7395 |
| Q8VED9 | 0.8066 | Q9CZ44 | 0.7823 | Q811N1 | 0.7390 |
| Q8BLN5 | 0.8061 | Q02819 | 0.7820 | P12382 | 0.7383 |
| Q9QYR9 | 0.8049 | P07356 | 0.7790 | Q9DAU1 | 0.7373 |
| Q9R118 | 0.8044 | Q3TW36 | 0.7774 | Q3UDR2 | 0.7372 |
| Q9CRY7 | 0.8041 | Q6P9T6 | 0.7756 | Q9CTI8 | 0.7359 |
| Q5HZH2 | 0.8039 | Q8VBT9 | 0.7745 | P16858 | 0.7356 |
| B2RTP7 | 0.8038 | P50516 | 0.7742 | Q6P1B9 | 0.7340 |
| Q6NZM8 | 0.8038 | P70697 | 0.7738 | A2AUE1 | 0.7339 |
| Q8BI84 | 0.8037 | A2AAN2 | 0.7731 | Q922H4 | 0.7320 |
| Q3TV20 | 0.8036 | Q80UL3 | 0.7729 | Q91YN9 | 0.7310 |
| B2RQQ5 | 0.8005 | Q3UM23 | 0.7728 | P24472 | 0.7301 |
| Q03145 | 0.7958 | Q91VE6 | 0.7716 | Q8C605 | 0.7292 |
| Q9CPT5 | 0.7955 | Q3U4W8 | 0.7698 | Q3UDS4 | 0.7290 |
| D3Z0G0 | 0.7954 | A0A087WSG5 | 0.7690 | A4FUW1 | 0.7288 |
| E9PYJ6 | 0.7948 | Q8BJ71 | 0.7690 | Q9R0E2 | 0.7285 |
| Q9JI13 | 0.7924 | Q3TUQ7 | 0.7689 | P70362 | 0.7285 |
| Q66L45 | 0.7921 | P63328 | 0.7679 | P17047 | 0.7275 |
| A2AKI5 | 0.7907 | Q9JJE7 | 0.7676 | Q9D6E8 | 0.7258 |
| Q3U6U7 | 0.7880 | Q3TJ52 | 0.7672 | Q4FJY5 | 0.7256 |
| K7Q751 | 0.7866 | P70699 | 0.7665 | Q9Z2G9 | 0.7254 |
| Q3TCE7 | 0.7840 | Q922S4 | 0.7662 | Q9EQ66 | 0.7239 |
| Q9CX60 | 0.7763 | D3Z619 | 0.7652 | Q8C586 | 0.7238 |
| D3Z5I1 | 0.7719 | Q9Z2L6 | 0.7618 | Q9D0F3 | 0.7223 |
| Q4VA28 | 0.7696 | Q3UGS9 | 0.7614 | P07356 | 0.7193 |
| Q9JKB3 | 0.7627 | O54734 | 0.7605 | Q3UIS6 | 0.7192 |
| P07141 | 0.7575 | D3YW48 | 0.7603 | A1L3S7 | 0.7191 |
| Q62241 | 0.7521 | Q9R233 | 0.7588 | Q9D8V0 | 0.7189 |
| Q7TQK1 | 0.7507 | Q9JJA4 | 0.7581 | Q66JR8 | 0.7175 |
| F8WHV1 | 0.7504 | Q9D880 | 0.7580 | E9PUH0 | 0.7172 |
| Q9D9V3 | 0.7497 | Q61584 | 0.7576 | Q920A5 | 0.7172 |
| Q3THZ8 | 0.7465 | Q99K48 | 0.7570 | Q8BH64 | 0.7167 |
| Q8VCN5 | 0.7326 | A2ARF6 | 0.7569 | Q8C838 | 0.7156 |
| Q78RK2 | 0.7101 | Q64674 | 0.7568 | Q8BND3 | 0.7151 |
| Q99LS3 | 0.7099 | Q3U468 | 0.7567 | G3X8Y7 | 0.7147 |
| Q3U6F1 | 0.6993 | Q5SF07 | 0.7563 | Q99MV1 | 0.7126 |
| Q1W5W7 | 0.6981 | Q9D0G0 | 0.7558 | Q04899 | 0.7121 |
| Q3TC83 | 0.6636 | Q3U0V1 | 0.7557 | Q8C9L6 | 0.7121 |
| Q5SSP3 | 0.6232 | G5E8R3 | 0.7549 | O88492 | 0.7118 |
| P04104 | 0.6232 | Q9Z0W3 | 0.7546 | O35593 | 0.7110 |
| P09528 | 0.6150 | Q99N87 | 0.7539 | Q7TPR4 | 0.7097 |
| G3X9T8 | 0.6062 | Q3UF95 | 0.7526 | P46737 | 0.7093 |
| Q3USG5 | 0.5953 | Q3UTE4 | 0.7525 | E0CY49 | 0.7089 |
| Q8BGZ7 | 0.5812 | P57784 | 0.7524 | Q3TRX4 | 0.7071 |
| Q64735 | 0.5313 | Q3UDE2 | 0.7523 | Q8BIW1 | 0.7066 |
|  |  | P61161 | 0.7510 | Q9D7J9 | 0.7065 |
|  |  | Q3TJN9 | 0.7498 | P32020 | 0.7064 |
|  |  | P10833 | 0.7494 | Q05DI7 | 0.7058 |
|  |  | Q8C545 | 0.7486 | Q8BFZ9 | 0.7048 |
|  |  | P51125 | 0.7485 | Q3V3N5 | 0.7044 |
|  |  | P08752 | 0.7479 | Q3TMX5 | 0.7031 |
|  |  | Q9EPE9 | 0.7479 | Q8C845 | 0.7030 |
|  |  | Q5SUR0 | 0.7478 | Q9EP83 | 0.7019 |
|  |  | P53702 | 0.7474 | P08207 | 0.7006 |
|  |  | Q6ZPE2 | 0.7469 | D3Z2J6 | 0.6996 |
|  |  | Q7TT04 | 0.7463 | Q924A9 | 0.6994 |
|  |  | Q61213 | 0.7462 | Q3TLJ5 | 0.6993 |
|  |  | A0A087WNV1 | 0.7461 | Q9DBG7 | 0.6989 |
|  |  | Q3TZP3 | 0.7460 | Q05186 | 0.6985 |
|  |  | D3YWT0 | 0.7460 | Q8R0M2 | 0.6984 |
|  |  | Q8CFI7 | 0.7456 | Q3UH60 | 0.6982 |
|  |  | Q5SSI6 | 0.7448 | P10649 | 0.6982 |
|  |  | Q3TV93 | 0.7448 | B1AWZ5 | 0.6973 |
|  |  | Q6PDM2 | 0.7423 | Q3UXM3 | 0.6971 |
|  |  | Q9JMH6 | 0.7407 | O08797 | 0.6970 |
|  |  | Q9D2G2 | 0.7404 | E9PWK1 | 0.6969 |
|  |  | Q8BMG7 | 0.7400 | Q7TQ39 | 0.6968 |
|  |  | P12382 | 0.7398 | Q8BLL4 | 0.6966 |
|  |  | Q3TFD0 | 0.7396 | P42125 | 0.6962 |
|  |  | Q3U3L3 | 0.7389 | Q3URT2 | 0.6953 |
|  |  | G3UYV7 | 0.7386 | P00493 | 0.6947 |
|  |  | Q8R436 | 0.7380 | Q5D098 | 0.6944 |
|  |  | E9Q586 | 0.7379 | Q99K30 | 0.6937 |
|  |  | Q8BHD7 | 0.7379 | A0A0A6YXX3 | 0.6931 |
|  |  | A2AW05 | 0.7378 | Q7TT42 | 0.6930 |
|  |  | G5E8R1 | 0.7371 | O08848 | 0.6926 |
|  |  | Q9JIQ4 | 0.7366 | P23506 | 0.6924 |
|  |  | Q6A022 | 0.7363 | Q3THS6 | 0.6922 |
|  |  | Q3UJP8 | 0.7360 | B7ZCU2 | 0.6919 |
|  |  | P61924 | 0.7350 | Q62422 | 0.6915 |
|  |  | Q3TPD9 | 0.7344 | A2ADY9 | 0.6915 |
|  |  | E0CYI7 | 0.7333 | Q6ZWQ7 | 0.6912 |
|  |  | Q3USP3 | 0.7325 | Q9Z2L6 | 0.6908 |
|  |  | Q3UK61 | 0.7324 | Q5U438 | 0.6899 |
|  |  | Q0P6B2 | 0.7324 | Q9CQ43 | 0.6897 |
|  |  | A2CG44 | 0.7322 | Q62145 | 0.6896 |
|  |  | Q3UX26 | 0.7322 | A0JLU6 | 0.6885 |
|  |  | Q9D6Y7 | 0.7311 | E9Q634 | 0.6884 |
|  |  | F8VQJ3 | 0.7310 | A2A6U3 | 0.6881 |
|  |  | Q00422 | 0.7308 | Q3UMR5 | 0.6879 |
|  |  | Q9WV03 | 0.7304 | Q5NC05 | 0.6875 |
|  |  | Q8CB77 | 0.7303 | Q921J2 | 0.6869 |
|  |  | E9QAJ9 | 0.7303 | Q8VCB1 | 0.6866 |
|  |  | Q61210 | 0.7302 | P70699 | 0.6866 |
|  |  | Q6R891 | 0.7288 | Q3UDQ7 | 0.6863 |
|  |  | P52432 | 0.7284 | Q8C550 | 0.6862 |
|  |  | P61027 | 0.7284 | P31938 | 0.6861 |
|  |  | E9QLB8 | 0.7276 | O54752 | 0.6859 |
|  |  | Q9QZE5 | 0.7264 | Q9DCC4 | 0.6857 |
|  |  | Q8CDZ5 | 0.7255 | P08113 | 0.6847 |
|  |  | Q9CRD0 | 0.7253 | Q9CTR1 | 0.6847 |
|  |  | Q3U6X7 | 0.7248 | Q9CQJ4 | 0.6843 |
|  |  | Q3U026 | 0.7247 | Q91WP9 | 0.6842 |
|  |  | P43247 | 0.7245 | Q8BVQ5 | 0.6842 |
|  |  | B1AZ15 | 0.7244 | Q3THH0 | 0.6841 |
|  |  | E9Q035 | 0.7244 | Q9CY49 | 0.6840 |
|  |  | D3YVX4 | 0.7235 | E9QAT4 | 0.6839 |
|  |  | Q9CYA0 | 0.7228 | P20152 | 0.6834 |
|  |  | O88967 | 0.7225 | Q3TVV9 | 0.6833 |
|  |  | P70188 | 0.7225 | P68368 | 0.6826 |
|  |  | Q9CQ75 | 0.7217 | G5E8R1 | 0.6826 |
|  |  | Q5SSP3 | 0.7214 | G5E850 | 0.6821 |
|  |  | Q9QXS1 | 0.7209 | A2AP32 | 0.6820 |
|  |  | Q61578 | 0.7203 | E9PXX7 | 0.6818 |
|  |  | Q8BML9 | 0.7195 | Q8R1G6 | 0.6815 |
|  |  | O88569 | 0.7190 | Q3UDD3 | 0.6813 |
|  |  | E9Q852 | 0.7176 | F6YQT7 | 0.6811 |
|  |  | Q9D706 | 0.7173 | Q9CPW4 | 0.6808 |
|  |  | Q3UJS6 | 0.7172 | Q9CPX6 | 0.6805 |
|  |  | Q99N93 | 0.7168 | Z4YJU8 | 0.6804 |
|  |  | Q3TIJ4 | 0.7158 | Q9DBS1 | 0.6804 |
|  |  | Q8C129 | 0.7152 | Q8BVL3 | 0.6803 |
|  |  | Q9CQ60 | 0.7152 | Q8VE88 | 0.6798 |
|  |  | Q8R0W0 | 0.7149 | P45878 | 0.6796 |
|  |  | Q9DCB8 | 0.7148 | Q8C545 | 0.6795 |
|  |  | Q8BSX8 | 0.7147 | P62307 | 0.6794 |
|  |  | E9QKZ2 | 0.7146 | Q9DD18 | 0.6794 |
|  |  | Q8K2H2 | 0.7146 | Q8VBT6 | 0.6788 |
|  |  | Q80YG4 | 0.7140 | Q9R0Y5 | 0.6783 |
|  |  | Q3TJ21 | 0.7130 | Q9WV60 | 0.6782 |
|  |  | P46471 | 0.7130 | Q6ZQI3 | 0.6782 |
|  |  | G3UVV4 | 0.7129 | E9QAT0 | 0.6779 |
|  |  | Q3TZZ7 | 0.7126 | Q99LC2 | 0.6777 |
|  |  | Q3V3R1 | 0.7124 | P51859 | 0.6776 |
|  |  | A0A087WNP6 | 0.7111 | Q5F258 | 0.6776 |
|  |  | Q8R326 | 0.7111 | F8WHM5 | 0.6773 |
|  |  | P56480 | 0.7110 | Q9Z2M7 | 0.6772 |
|  |  | E9Q6R3 | 0.7105 | F8VQE9 | 0.6769 |
|  |  | E9QP99 | 0.7097 | P17439 | 0.6767 |
|  |  | E9Q1S3 | 0.7093 | Q3U9U5 | 0.6767 |
|  |  | P68368 | 0.7090 | E9QLA5 | 0.6765 |
|  |  | Q3TW01 | 0.7088 | O88967 | 0.6760 |
|  |  | E9QAI5 | 0.7081 | Q8R361 | 0.6758 |
|  |  | F6ZDS4 | 0.7071 | E0CYE2 | 0.6757 |
|  |  | Q99LI2 | 0.7062 | Q9CZH7 | 0.6756 |
|  |  | P26039 | 0.7062 | O88712 | 0.6748 |
|  |  | G3X963 | 0.7056 | B2RQ68 | 0.6746 |
|  |  | Q63844 | 0.7055 | Q9EQQ2 | 0.6744 |
|  |  | Q9CX60 | 0.7054 | P10630 | 0.6742 |
|  |  | A0A087WQE6 | 0.7052 | D3Z3F8 | 0.6742 |
|  |  | Q3UZI6 | 0.7050 | Q9D828 | 0.6740 |
|  |  | P97429 | 0.7049 | Q6ZPE2 | 0.6739 |
|  |  | P62315 | 0.7047 | P41731 | 0.6737 |
|  |  | Q8K009 | 0.7045 | O54782 | 0.6736 |
|  |  | P54103 | 0.7045 | Q64310 | 0.6735 |
|  |  | Q505F5 | 0.7042 | Q99LN9 | 0.6732 |
|  |  | Q3THK7 | 0.7041 | P08228 | 0.6732 |
|  |  | P42208 | 0.7040 | A2A4B3 | 0.6729 |
|  |  | D3YW87 | 0.7037 | Q9CT36 | 0.6727 |
|  |  | Q9WTI7 | 0.7036 | Q60972 | 0.6726 |
|  |  | Q91VR8 | 0.7031 | Q3UIZ0 | 0.6724 |
|  |  | Q7TMB8 | 0.7028 | Q3U5I9 | 0.6721 |
|  |  | Q6NS46 | 0.7028 | Q3UAG2 | 0.6720 |
|  |  | P53569 | 0.7023 | Q9CR60 | 0.6715 |
|  |  | Q8C605 | 0.7020 | Q9CYA0 | 0.6714 |
|  |  | Q91VJ2 | 0.7014 | Q9QWZ1 | 0.6713 |
|  |  | Q921H8 | 0.7011 | Q07797 | 0.6710 |
|  |  | Q8BYA0 | 0.7006 | Q8BHC7 | 0.6710 |
|  |  | G3X8Y7 | 0.7005 | Q922Q1 | 0.6708 |
|  |  | Q9QXE7 | 0.7003 | Q99KV1 | 0.6708 |
|  |  | P47963 | 0.6997 | Q3UQM5 | 0.6705 |
|  |  | Q8BI84 | 0.6991 | P50516 | 0.6704 |
|  |  | P97855 | 0.6985 | Q01320 | 0.6702 |
|  |  | Q9DCL9 | 0.6982 | Q60749 | 0.6697 |
|  |  | Q8BK57 | 0.6981 | P43275 | 0.6695 |
|  |  | Q62376 | 0.6980 | B2RY51 | 0.6691 |
|  |  | Q3UM45 | 0.6977 | P48678 | 0.6691 |
|  |  | Q3TKC5 | 0.6974 | Q3V3R1 | 0.6690 |
|  |  | Q9CWJ9 | 0.6969 | D3YZA1 | 0.6683 |
|  |  | Q8C166 | 0.6965 | Q9Z1N5 | 0.6680 |
|  |  | Q8BU31 | 0.6964 | Q91WS0 | 0.6679 |
|  |  | Q3UNJ3 | 0.6962 | N0E4C0 | 0.6677 |
|  |  | P48036 | 0.6953 | O35295 | 0.6674 |
|  |  | Q3UAS4 | 0.6949 | Q60575 | 0.6674 |
|  |  | Q80SW1 | 0.6945 | Q8CHC7 | 0.6672 |
|  |  | Q6P5D8 | 0.6940 | Q9CUB4 | 0.6670 |
|  |  | G3X8R5 | 0.6937 | Q9QZL0 | 0.6665 |
|  |  | A0A068F126 | 0.6934 | Q8BMD8 | 0.6665 |
|  |  | Q61753 | 0.6934 | Q80U93 | 0.6663 |
|  |  | Q9CYL5 | 0.6930 | Q8K0Z7 | 0.6662 |
|  |  | Q5XG71 | 0.6929 | Q9QXS1 | 0.6657 |
|  |  | Q9R1P1 | 0.6918 | E9Q4M4 | 0.6655 |
|  |  | P70399 | 0.6912 | Q9DB73 | 0.6652 |
|  |  | Q497Z1 | 0.6911 | Q3TSD4 | 0.6651 |
|  |  | Q9CQ18 | 0.6907 | Q3TGI0 | 0.6646 |
|  |  | Q8BWT1 | 0.6905 | E9Q1V5 | 0.6645 |
|  |  | O08582 | 0.6905 | Q11011 | 0.6643 |
|  |  | A2A5R2 | 0.6902 | P97822 | 0.6643 |
|  |  | Q3TF87 | 0.6901 | A2ALA4 | 0.6640 |
|  |  | P99026 | 0.6900 | Q8BIP0 | 0.6637 |
|  |  | P35486 | 0.6900 | O55143 | 0.6635 |
|  |  | Q8BP92 | 0.6898 | Q5RKP0 | 0.6634 |
|  |  | Q8R086 | 0.6894 | P15532 | 0.6633 |
|  |  | A0A068BGU5 | 0.6894 | Q8C129 | 0.6632 |
|  |  | P43274 | 0.6891 | P50518 | 0.6629 |
|  |  | H3BLJ9 | 0.6889 | J3QPZ8 | 0.6628 |
|  |  | Q3UGC1 | 0.6889 | U3RKD2 | 0.6628 |
|  |  | O08795 | 0.6882 | Q80UU9 | 0.6627 |
|  |  | Q80YR4 | 0.6880 | Q99KQ4 | 0.6624 |
|  |  | Q91V12 | 0.6877 | Q9CPQ3 | 0.6623 |
|  |  | Q99KR3 | 0.6874 | Q9D0S9 | 0.6622 |
|  |  | Q9ESU6 | 0.6873 | S4R294 | 0.6619 |
|  |  | E9QN31 | 0.6862 | I7HJS1 | 0.6618 |
|  |  | Q7TMY8 | 0.6861 | E9PYT3 | 0.6618 |
|  |  | P60335 | 0.6854 | Q8BIA4 | 0.6615 |
|  |  | Q8VDP6 | 0.6850 | P61600 | 0.6614 |
|  |  | Q0KL02 | 0.6847 | Q3TDQ1 | 0.6613 |
|  |  | Q7TQE2 | 0.6845 | Q3U449 | 0.6611 |
|  |  | O54984 | 0.6844 | Q3TW96 | 0.6611 |
|  |  | Q5SUF2 | 0.6843 | Q3UKA1 | 0.6604 |
|  |  | P58389 | 0.6843 | Q61213 | 0.6602 |
|  |  | Q99L13 | 0.6841 | E9Q7A5 | 0.6600 |
|  |  | Q8K354 | 0.6839 | A6H663 | 0.6600 |
|  |  | Q91Z53 | 0.6839 | Q9QYJ3 | 0.6598 |
|  |  | Q9EP82 | 0.6837 | Q923D2 | 0.6595 |
|  |  | O54825 | 0.6836 | P06745 | 0.6591 |
|  |  | Q9CR25 | 0.6825 | Q3V460 | 0.6583 |
|  |  | Q9CX86 | 0.6825 | B9EKJ7 | 0.6582 |
|  |  | Q5RJV4 | 0.6824 | P61982 | 0.6582 |
|  |  | Q9ER72 | 0.6820 | E9Q175 | 0.6581 |
|  |  | P52480 | 0.6819 | Q3TW36 | 0.6580 |
|  |  | Q9D883 | 0.6818 | Q9D4H1 | 0.6579 |
|  |  | Q99JB2 | 0.6808 | Q7TMM9 | 0.6575 |
|  |  | O08529 | 0.6804 | P40124 | 0.6575 |
|  |  | Q05DI7 | 0.6804 | Q99PT1 | 0.6574 |
|  |  | Q3TWN8 | 0.6801 | Q8R3R9 | 0.6571 |
|  |  | Q9CY64 | 0.6799 | Q3TET5 | 0.6570 |
|  |  | Q9DCM0 | 0.6791 | Q3U3L3 | 0.6569 |
|  |  | Q8BGC4 | 0.6790 | Q9CQR4 | 0.6566 |
|  |  | P97351 | 0.6786 | Q91V47 | 0.6563 |
|  |  | Q9CSU0 | 0.6785 | D3Z074 | 0.6563 |
|  |  | Q5SVG5 | 0.6783 | Q91X01 | 0.6562 |
|  |  | P70372 | 0.6781 | P97429 | 0.6560 |
|  |  | Q91W09 | 0.6779 | Q5SW88 | 0.6558 |
|  |  | Q3TKU6 | 0.6777 | G3X926 | 0.6558 |
|  |  | Q9D5T0 | 0.6776 | P20029 | 0.6557 |
|  |  | E9PX48 | 0.6772 | Q9JHS9 | 0.6555 |
|  |  | Q9D1A2 | 0.6763 | P26350 | 0.6555 |
|  |  | Q3THI5 | 0.6754 | Q80UL3 | 0.6553 |
|  |  | A2A547 | 0.6747 | Q62048 | 0.6552 |
|  |  | P62192 | 0.6746 | Q9WVM1 | 0.6551 |
|  |  | F6ZFU0 | 0.6746 | Q80TX7 | 0.6548 |
|  |  | D3YTQ9 | 0.6740 | Q9JHW2 | 0.6547 |
|  |  | Q03265 | 0.6739 | A2ALF0 | 0.6546 |
|  |  | E9QP59 | 0.6734 | B2RTP7 | 0.6545 |
|  |  | Q3UPH1 | 0.6731 | D3Z5P5 | 0.6542 |
|  |  | Q8BGS0 | 0.6718 | Q6NXL1 | 0.6541 |
|  |  | Q62318 | 0.6714 | E9QP99 | 0.6540 |
|  |  | Q9D0S9 | 0.6714 | Q3U9Q8 | 0.6535 |
|  |  | Q3UDI8 | 0.6709 | D3YXK2 | 0.6533 |
|  |  | O09061 | 0.6706 | G8DXR6 | 0.6533 |
|  |  | A6H663 | 0.6698 | Q8VHC3 | 0.6532 |
|  |  | Q8JZM0 | 0.6698 | Q6IRU2 | 0.6531 |
|  |  | P17182 | 0.6696 | Q3UQD0 | 0.6531 |
|  |  | G3UVU9 | 0.6694 | Q6ZPX7 | 0.6529 |
|  |  | Q8R509 | 0.6694 | O09061 | 0.6525 |
|  |  | A1A4T2 | 0.6689 | Q3U0B0 | 0.6525 |
|  |  | Q3TA68 | 0.6689 | Q3TEA8 | 0.6524 |
|  |  | P46664 | 0.6689 | Q3TC93 | 0.6523 |
|  |  | Q64521 | 0.6688 | Q9QUM9 | 0.6520 |
|  |  | P19157 | 0.6687 | O35566 | 0.6515 |
|  |  | D3Z5I1 | 0.6684 | Q9D1A2 | 0.6514 |
|  |  | Q7TPY3 | 0.6683 | Q05BH6 | 0.6513 |
|  |  | F7ACR9 | 0.6680 | Q6NXI6 | 0.6510 |
|  |  | Q921C5 | 0.6678 | Q8R146 | 0.6509 |
|  |  | P54116 | 0.6678 | Q8BU31 | 0.6509 |
|  |  | Q3V0L4 | 0.6673 | E9QKG6 | 0.6505 |
|  |  | Q91V41 | 0.6668 | Q8BHB4 | 0.6504 |
|  |  | Q3TU36 | 0.6666 | P21550 | 0.6503 |
|  |  | Q3TG58 | 0.6665 | O54962 | 0.6503 |
|  |  | P08228 | 0.6662 | Q8C6G4 | 0.6503 |
|  |  | Q6ZPJ3 | 0.6661 | P84084 | 0.6502 |
|  |  | Q3TCE7 | 0.6661 | Q3TBW2 | 0.6499 |
|  |  | Q9DC69 | 0.6652 | P70697 | 0.6497 |
|  |  | Z4YKT6 | 0.6648 | Q9D5V5 | 0.6497 |
|  |  | Q3THP1 | 0.6642 | I6L9H1 | 0.6495 |
|  |  | Q5RL55 | 0.6642 | Q8R5K4 | 0.6495 |
|  |  | B9EHJ3 | 0.6642 | P56873 | 0.6494 |
|  |  | Q3V471 | 0.6641 | Q80X50 | 0.6493 |
|  |  | Q3TH64 | 0.6640 | B8JJI4 | 0.6493 |
|  |  | Q9CW03 | 0.6624 | Q9Z247 | 0.6488 |
|  |  | Q8VCQ8 | 0.6622 | Q3UW53 | 0.6488 |
|  |  | Q3U5L3 | 0.6615 | Q8CCJ3 | 0.6485 |
|  |  | Q9Z1Z0 | 0.6610 | Q8JZX4 | 0.6483 |
|  |  | Q9CPY7 | 0.6608 | Q63844 | 0.6483 |
|  |  | Q91W50 | 0.6608 | Q3UPH1 | 0.6483 |
|  |  | Q3TFE8 | 0.6604 | Q3UE99 | 0.6482 |
|  |  | Q60715 | 0.6602 | P42669 | 0.6480 |
|  |  | Q8C1X9 | 0.6601 | P62141 | 0.6476 |
|  |  | Q9JMD0 | 0.6600 | Q8R1F1 | 0.6474 |
|  |  | Q921E2 | 0.6600 | Q3TM89 | 0.6473 |
|  |  | P26041 | 0.6599 | Q7TMQ7 | 0.6472 |
|  |  | Q9CPN8 | 0.6599 | Q6A0A9 | 0.6471 |
|  |  | Q8CFX1 | 0.6599 | Q62186 | 0.6470 |
|  |  | Q3TB65 | 0.6597 | Q8VD12 | 0.6469 |
|  |  | Q3TWP9 | 0.6594 | D3YWT0 | 0.6466 |
|  |  | P35293 | 0.6588 | Q62165 | 0.6466 |
|  |  | O70194 | 0.6587 | Q9JKR6 | 0.6465 |
|  |  | A2A6U3 | 0.6587 | Q8CFX1 | 0.6464 |
|  |  | Q9WU62 | 0.6585 | Q71FD7 | 0.6464 |
|  |  | B8X349 | 0.6582 | Q8BFW7 | 0.6462 |
|  |  | P30416 | 0.6579 | Q8VCN9 | 0.6462 |
|  |  | O88477 | 0.6574 | Q99JF8 | 0.6462 |
|  |  | P62814 | 0.6573 | Z4YKV1 | 0.6458 |
|  |  | Q9QUM9 | 0.6573 | Q3UE92 | 0.6454 |
|  |  | Q921S7 | 0.6569 | Q9D820 | 0.6453 |
|  |  | Q9D8X2 | 0.6569 | Q99KI3 | 0.6450 |
|  |  | E9Q5I9 | 0.6568 | Q9D7S9 | 0.6449 |
|  |  | Q9QZL0 | 0.6565 | F8WIH0 | 0.6447 |
|  |  | A2A513 | 0.6561 | Q99PL5 | 0.6447 |
|  |  | P60670 | 0.6553 | P33174 | 0.6446 |
|  |  | Q9WVA4 | 0.6543 | Q8BFQ8 | 0.6446 |
|  |  | A2AJ72 | 0.6540 | Q8C7X8 | 0.6445 |
|  |  | Q9EPU0 | 0.6536 | Q8BP71 | 0.6444 |
|  |  | P24547 | 0.6535 | O55135 | 0.6441 |
|  |  | Q61553 | 0.6531 | P52825 | 0.6440 |
|  |  | Q8BY87 | 0.6530 | A2BFF8 | 0.6439 |
|  |  | K3W4Q8 | 0.6530 | B1AZ46 | 0.6439 |
|  |  | Q91VH6 | 0.6529 | F6SPK0 | 0.6437 |
|  |  | P52196 | 0.6524 | P99024 | 0.6437 |
|  |  | Q9DBG5 | 0.6524 | Q0VGB7 | 0.6437 |
|  |  | P62918 | 0.6516 | Q6ZWM4 | 0.6436 |
|  |  | P26040 | 0.6515 | Q8BP60 | 0.6436 |
|  |  | Q8BL36 | 0.6511 | Q3URX8 | 0.6434 |
|  |  | Q8R2Y8 | 0.6510 | P68373 | 0.6434 |
|  |  | Q60931 | 0.6503 | Q8CHP8 | 0.6432 |
|  |  | P52431 | 0.6502 | Q3TAD4 | 0.6432 |
|  |  | P63017 | 0.6500 | E9QKL6 | 0.6432 |
|  |  | Q6P1B9 | 0.6500 | P97868 | 0.6432 |
|  |  | Q8C586 | 0.6494 | O88874 | 0.6431 |
|  |  | Q9Z1Z2 | 0.6492 | D6RG99 | 0.6430 |
|  |  | B1AY13 | 0.6492 | Q8CA04 | 0.6428 |
|  |  | Q3UKA1 | 0.6490 | Q3UJW9 | 0.6427 |
|  |  | E9Q390 | 0.6487 | Q9D7X3 | 0.6425 |
|  |  | P54823 | 0.6485 | Q9DBH5 | 0.6422 |
|  |  | P60710 | 0.6478 | B2RQ83 | 0.6421 |
|  |  | Q9ERS2 | 0.6478 | P70302 | 0.6420 |
|  |  | Q9CRC8 | 0.6473 | Q922R8 | 0.6419 |
|  |  | B1AT03 | 0.6465 | Q8CH18 | 0.6419 |
|  |  | Q922D8 | 0.6464 | Q9CZT6 | 0.6419 |
|  |  | Q01730 | 0.6463 | A2AJ72 | 0.6419 |
|  |  | A0JLN6 | 0.6463 | Q3U646 | 0.6418 |
|  |  | Q3UA06 | 0.6463 | Q9D8B3 | 0.6417 |
|  |  | O88712 | 0.6459 | Q69ZJ6 | 0.6417 |
|  |  | P49586 | 0.6458 | Q9CQL6 | 0.6417 |
|  |  | P80314 | 0.6458 | P61804 | 0.6416 |
|  |  | Q9DCR2 | 0.6456 | B1AZ15 | 0.6414 |
|  |  | P70698 | 0.6455 | Q8R0R0 | 0.6411 |
|  |  | D3YW20 | 0.6449 | P52624 | 0.6409 |
|  |  | Q9Z2M7 | 0.6447 | Q9QZ82 | 0.6409 |
|  |  | Q9DBR0 | 0.6446 | Q8R3B1 | 0.6409 |
|  |  | Q3U9Q8 | 0.6446 | Q3TKB7 | 0.6409 |
|  |  | P16858 | 0.6445 | Q6DVA0 | 0.6409 |
|  |  | Q3TVZ1 | 0.6444 | O08599 | 0.6408 |
|  |  | Q3UW32 | 0.6441 | O08709 | 0.6408 |
|  |  | A1A596 | 0.6439 | Q01853 | 0.6408 |
|  |  | Q61833 | 0.6438 | Q9ESP1 | 0.6406 |
|  |  | Q8BJY1 | 0.6437 | P29595 | 0.6406 |
|  |  | Q9D820 | 0.6435 | Q9CVB6 | 0.6406 |
|  |  | A2A4Z1 | 0.6433 | Q60598 | 0.6406 |
|  |  | Q9JKB1 | 0.6432 | D3YW48 | 0.6405 |
|  |  | A4FUW1 | 0.6431 | Q8CCG5 | 0.6405 |
|  |  | Q05816 | 0.6430 | Q6P9T6 | 0.6404 |
|  |  | O54784 | 0.6429 | P70677 | 0.6401 |
|  |  | Q9DCU6 | 0.6428 | Q9WV03 | 0.6400 |
|  |  | Q80YW9 | 0.6422 | Q9JJ89 | 0.6400 |
|  |  | Q8R395 | 0.6417 | F8WIB1 | 0.6397 |
|  |  | O35134 | 0.6413 | P63328 | 0.6395 |
|  |  | P52825 | 0.6413 | Q8BP92 | 0.6394 |
|  |  | P17751 | 0.6413 | Q8CH25 | 0.6394 |
|  |  | Q60932 | 0.6413 | P47199 | 0.6393 |
|  |  | Q99J36 | 0.6411 | Q62446 | 0.6393 |
|  |  | Q80U72 | 0.6408 | E9QP46 | 0.6392 |
|  |  | Q921T2 | 0.6399 | E9QP59 | 0.6390 |
|  |  | Q64105 | 0.6395 | F6ZFT1 | 0.6389 |
|  |  | Q6ZWX6 | 0.6391 | Q80W54 | 0.6389 |
|  |  | Q3TAD4 | 0.6390 | P17665 | 0.6387 |
|  |  | Q8QZY1 | 0.6389 | P26041 | 0.6386 |
|  |  | D3Z7C0 | 0.6388 | P38060 | 0.6386 |
|  |  | Q9EP83 | 0.6388 | P35564 | 0.6386 |
|  |  | Q3U417 | 0.6387 | Q3TGH6 | 0.6385 |
|  |  | P47856 | 0.6385 | Q6PIU9 | 0.6383 |
|  |  | Q9DCT8 | 0.6382 | B1ATZ0 | 0.6383 |
|  |  | O35129 | 0.6379 | Q8CB77 | 0.6382 |
|  |  | Q3TQP7 | 0.6375 | P28033 | 0.6381 |
|  |  | Q60676 | 0.6374 | Q91W09 | 0.6379 |
|  |  | Q3ULJ0 | 0.6374 | Q6PB51 | 0.6379 |
|  |  | Q3TML7 | 0.6373 | P62192 | 0.6378 |
|  |  | O35841 | 0.6373 | A2AQ17 | 0.6378 |
|  |  | E9QP46 | 0.6372 | Q80ZS3 | 0.6377 |
|  |  | O08553 | 0.6371 | Q8C5B5 | 0.6377 |
|  |  | P36552 | 0.6365 | O55057 | 0.6376 |
|  |  | Q8R323 | 0.6364 | E9Q197 | 0.6371 |
|  |  | Q9CQ48 | 0.6363 | B9EHZ5 | 0.6371 |
|  |  | Q78ZA7 | 0.6363 | F6SQH7 | 0.6371 |
|  |  | Q9D6K5 | 0.6362 | Q9D1M4 | 0.6370 |
|  |  | Q923D2 | 0.6361 | P97930 | 0.6368 |
|  |  | P14152 | 0.6361 | O08847 | 0.6368 |
|  |  | E9QLA5 | 0.6360 | P51125 | 0.6367 |
|  |  | Q99J62 | 0.6359 | O35344 | 0.6365 |
|  |  | Q9DCN2 | 0.6357 | Q9CVT6 | 0.6364 |
|  |  | Q8K1R7 | 0.6355 | Q60932 | 0.6362 |
|  |  | E9Q7G1 | 0.6350 | Q9Z315 | 0.6362 |
|  |  | Q99LC5 | 0.6350 | Q3TCH7 | 0.6362 |
|  |  | Q8VHY0 | 0.6349 | P68372 | 0.6361 |
|  |  | Q8R146 | 0.6349 | O08795 | 0.6358 |
|  |  | Q8BMF4 | 0.6349 | Q8C0I1 | 0.6357 |
|  |  | A0A087WRU0 | 0.6348 | Q9R257 | 0.6356 |
|  |  | Q64511 | 0.6347 | O70475 | 0.6356 |
|  |  | E9Q6U4 | 0.6346 | P63017 | 0.6355 |
|  |  | Q3UGR5 | 0.6343 | Q8R5C5 | 0.6354 |
|  |  | P17439 | 0.6340 | Q8K354 | 0.6354 |
|  |  | Q3ULT8 | 0.6334 | Q9ERE7 | 0.6353 |
|  |  | Q3TSZ4 | 0.6333 | P81117 | 0.6352 |
|  |  | Q91V01 | 0.6333 | Q9D6K8 | 0.6351 |
|  |  | B2RXS4 | 0.6326 | Q8VCQ8 | 0.6350 |
|  |  | Q3UJZ7 | 0.6322 | Q8BNU0 | 0.6349 |
|  |  | E9QL31 | 0.6321 | E9PUD2 | 0.6349 |
|  |  | Q8CI95 | 0.6320 | Q3TG21 | 0.6347 |
|  |  | P20029 | 0.6319 | P48036 | 0.6346 |
|  |  | P35505 | 0.6317 | Q64337 | 0.6346 |
|  |  | Q3UGL3 | 0.6317 | Q80X85 | 0.6346 |
|  |  | Q9CR57 | 0.6316 | Q8BPS5 | 0.6344 |
|  |  | Q8C788 | 0.6314 | Q8VDD5 | 0.6343 |
|  |  | G3X9L6 | 0.6311 | P47753 | 0.6343 |
|  |  | Q3U2G2 | 0.6310 | Q8C7C4 | 0.6340 |
|  |  | Q9WTP6 | 0.6310 | P97855 | 0.6340 |
|  |  | F8WIB1 | 0.6309 | P17918 | 0.6340 |
|  |  | H7BWX9 | 0.6308 | Q3TUW9 | 0.6339 |
|  |  | P97310 | 0.6306 | E9QAH1 | 0.6339 |
|  |  | Q91V89 | 0.6303 | Q8C660 | 0.6339 |
|  |  | P47753 | 0.6303 | O08738 | 0.6338 |
|  |  | Q9D1D4 | 0.6302 | P62075 | 0.6338 |
|  |  | O08810 | 0.6299 | Q8BVA5 | 0.6337 |
|  |  | Q9CSH3 | 0.6298 | Q3TJ10 | 0.6334 |
|  |  | Q8BKC5 | 0.6297 | B1AXN9 | 0.6334 |
|  |  | P54276 | 0.6296 | Q9CQU3 | 0.6333 |
|  |  | Q3U9R7 | 0.6294 | P41241 | 0.6333 |
|  |  | Q9CPT5 | 0.6289 | Q62318 | 0.6333 |
|  |  | B2RQ83 | 0.6284 | P54823 | 0.6332 |
|  |  | Q3TIV5 | 0.6281 | Q8BGS2 | 0.6331 |
|  |  | E9Q197 | 0.6279 | Q9D0J2 | 0.6330 |
|  |  | Q921X9 | 0.6279 | Q6PGB6 | 0.6330 |
|  |  | A2ADY9 | 0.6277 | Q2YDW1 | 0.6330 |
|  |  | P48771 | 0.6274 | B2RXS4 | 0.6329 |
|  |  | Q9JLI6 | 0.6273 | B7ZWI2 | 0.6329 |
|  |  | N0E4C0 | 0.6272 | Q01730 | 0.6328 |
|  |  | O08847 | 0.6269 | A0A068BIU7 | 0.6328 |
|  |  | A0PJ90 | 0.6268 | Q3UBB0 | 0.6327 |
|  |  | Q8BGH2 | 0.6267 | Q3TGI9 | 0.6327 |
|  |  | Q6PF96 | 0.6267 | F6RPJ9 | 0.6326 |
|  |  | Q9WUM3 | 0.6266 | Q99J36 | 0.6325 |
|  |  | Q80UJ7 | 0.6265 | Q05C51 | 0.6325 |
|  |  | P51150 | 0.6245 | Q8C5U8 | 0.6325 |
|  |  | Q8BIZ9 | 0.6242 | Q8BVQ0 | 0.6324 |
|  |  | P06745 | 0.6240 | Q6A022 | 0.6324 |
|  |  | Q9DBJ1 | 0.6236 | D3YW87 | 0.6323 |
|  |  | Q99NB8 | 0.6235 | Q3TDD9 | 0.6322 |
|  |  | Q9R0P5 | 0.6235 | P62334 | 0.6322 |
|  |  | F6VQ81 | 0.6231 | O55234 | 0.6321 |
|  |  | Q3UIJ2 | 0.6227 | Q8C9B9 | 0.6321 |
|  |  | O35344 | 0.6226 | Q8CIB5 | 0.6319 |
|  |  | Q922F4 | 0.6223 | Q8JZM0 | 0.6319 |
|  |  | Q91ZX7 | 0.6223 | G3UVV4 | 0.6319 |
|  |  | O09172 | 0.6221 | Q9DAW9 | 0.6318 |
|  |  | D3YXK2 | 0.6220 | P09405 | 0.6317 |
|  |  | B2RQQ5 | 0.6211 | Q99L47 | 0.6314 |
|  |  | I4DCY6 | 0.6210 | A0A087WQD1 | 0.6313 |
|  |  | Q922R8 | 0.6207 | Q8K2F8 | 0.6313 |
|  |  | A2ALB2 | 0.6207 | P27773 | 0.6311 |
|  |  | J3QK23 | 0.6206 | Q8K3A8 | 0.6309 |
|  |  | P62806 | 0.6201 | Q9D1H8 | 0.6309 |
|  |  | Q9QUR6 | 0.6201 | Q32P00 | 0.6309 |
|  |  | D3YVN7 | 0.6201 | Q99P72 | 0.6309 |
|  |  | P99028 | 0.6200 | Q9D517 | 0.6306 |
|  |  | P28271 | 0.6200 | A2AP78 | 0.6305 |
|  |  | Q80VJ3 | 0.6199 | Q99LJ0 | 0.6304 |
|  |  | P63168 | 0.6197 | P62320 | 0.6302 |
|  |  | Q8CCG5 | 0.6195 | P62137 | 0.6302 |
|  |  | E9QLZ0 | 0.6194 | Q99JW7 | 0.6302 |
|  |  | Q5RKP0 | 0.6192 | Q3U7I9 | 0.6301 |
|  |  | Q3TDF8 | 0.6189 | B7ZNW0 | 0.6300 |
|  |  | Q80X90 | 0.6189 | P50637 | 0.6298 |
|  |  | P61021 | 0.6189 | Q8BJY1 | 0.6297 |
|  |  | Q3TXS7 | 0.6187 | Q9DCN2 | 0.6296 |
|  |  | E9Q7G0 | 0.6186 | Q8R5J9 | 0.6295 |
|  |  | P35979 | 0.6183 | Q99JI4 | 0.6294 |
|  |  | Q0VGB7 | 0.6183 | Q8BTI8 | 0.6294 |
|  |  | D3Z0M9 | 0.6180 | Q9WV55 | 0.6294 |
|  |  | Q80X85 | 0.6178 | E9PYC6 | 0.6294 |
|  |  | Q8R2M2 | 0.6176 | Q8K1A6 | 0.6293 |
|  |  | A0A087WPL5 | 0.6169 | P62754 | 0.6292 |
|  |  | Q3UNN4 | 0.6169 | Q921C5 | 0.6292 |
|  |  | Q8R1B4 | 0.6167 | Q3V1L7 | 0.6291 |
|  |  | Q3UD67 | 0.6165 | Q60855 | 0.6290 |
|  |  | Q3TAW4 | 0.6164 | B2RXR6 | 0.6290 |
|  |  | Q9DAW6 | 0.6161 | Q8BYA0 | 0.6290 |
|  |  | Q8BXZ1 | 0.6154 | B2RXT3 | 0.6290 |
|  |  | Q922J3 | 0.6152 | Q8BIJ6 | 0.6289 |
|  |  | Q8BLN5 | 0.6150 | Q61210 | 0.6288 |
|  |  | B1AT10 | 0.6149 | Q3U9R7 | 0.6288 |
|  |  | D3Z5P5 | 0.6148 | Q5NCU4 | 0.6288 |
|  |  | Q91WP9 | 0.6143 | Q3TI63 | 0.6288 |
|  |  | Q9ERK4 | 0.6143 | Q3US65 | 0.6288 |
|  |  | Q99KJ8 | 0.6143 | Q3UM18 | 0.6287 |
|  |  | O70569 | 0.6140 | Q6ZWS7 | 0.6285 |
|  |  | O08738 | 0.6139 | F8VPK5 | 0.6285 |
|  |  | P13439 | 0.6134 | A0A068BFR6 | 0.6285 |
|  |  | Q9EP69 | 0.6133 | P21981 | 0.6284 |
|  |  | Q8CIV8 | 0.6128 | A2CG44 | 0.6284 |
|  |  | P47754 | 0.6128 | Q9CRD0 | 0.6282 |
|  |  | P57780 | 0.6127 | Q8CIV8 | 0.6282 |
|  |  | Q66L45 | 0.6125 | Q62418 | 0.6282 |
|  |  | P61600 | 0.6125 | Q7TQH0 | 0.6280 |
|  |  | Q3U281 | 0.6124 | Q61425 | 0.6280 |
|  |  | P48678 | 0.6123 | P14211 | 0.6280 |
|  |  | Q7TPT7 | 0.6120 | Q8K2H2 | 0.6280 |
|  |  | Q8BMJ2 | 0.6117 | Q99KR7 | 0.6280 |
|  |  | Q3UXM3 | 0.6117 | P53569 | 0.6279 |
|  |  | P11499 | 0.6111 | Q8K4Q8 | 0.6278 |
|  |  | Q9Z1F9 | 0.6106 | Q3TEU8 | 0.6277 |
|  |  | Q5U438 | 0.6103 | Q68FL6 | 0.6277 |
|  |  | O55234 | 0.6103 | Q91V12 | 0.6277 |
|  |  | Q9QZ23 | 0.6103 | Q99LI2 | 0.6276 |
|  |  | E9Q585 | 0.6102 | Q8BK57 | 0.6276 |
|  |  | Q99P72 | 0.6100 | Q3UXD8 | 0.6275 |
|  |  | Q8BFZ9 | 0.6099 | Q8C2Q8 | 0.6275 |
|  |  | Q6PB52 | 0.6099 | Q61927 | 0.6274 |
|  |  | Q9JIF7 | 0.6097 | Q3TF87 | 0.6274 |
|  |  | P17742 | 0.6096 | Q8C2A3 | 0.6274 |
|  |  | Q9Z1N5 | 0.6095 | Q9CQC6 | 0.6272 |
|  |  | P53994 | 0.6094 | Q80ZW2 | 0.6272 |
|  |  | P49717 | 0.6092 | O70252 | 0.6270 |
|  |  | Q8K0D5 | 0.6092 | Q9D1E6 | 0.6270 |
|  |  | O35075 | 0.6090 | G3UZY2 | 0.6270 |
|  |  | Q3TGI9 | 0.6088 | Q3TBQ3 | 0.6269 |
|  |  | P28474 | 0.6088 | A0A087WNP6 | 0.6269 |
|  |  | Q6A099 | 0.6087 | Q3TV94 | 0.6268 |
|  |  | Q8VDN2 | 0.6087 | Q69ZY3 | 0.6268 |
|  |  | Q921N6 | 0.6080 | Q921M7 | 0.6267 |
|  |  | Q569Z5 | 0.6079 | P53702 | 0.6266 |
|  |  | P45376 | 0.6079 | P70441 | 0.6265 |
|  |  | Q7TPR4 | 0.6078 | D3Z6S1 | 0.6265 |
|  |  | B9EKP5 | 0.6075 | P24527 | 0.6261 |
|  |  | Q8K2Z4 | 0.6075 | D3Z7E5 | 0.6260 |
|  |  | Q99N15 | 0.6071 | O55222 | 0.6260 |
|  |  | Q3UW66 | 0.6071 | P55096 | 0.6260 |
|  |  | Q91XI1 | 0.6065 | Q8R3C0 | 0.6259 |
|  |  | Q8C878 | 0.6059 | O35114 | 0.6258 |
|  |  | P62962 | 0.6057 | E9Q6J5 | 0.6257 |
|  |  | Q8JZQ9 | 0.6057 | Q8C5Q4 | 0.6256 |
|  |  | E9QMV2 | 0.6055 | Q9R1P4 | 0.6256 |
|  |  | Q0QEW9 | 0.6052 | O70435 | 0.6255 |
|  |  | Q05BN2 | 0.6051 | B1AT36 | 0.6255 |
|  |  | Q922A3 | 0.6049 | Q8C788 | 0.6255 |
|  |  | Q6P4S8 | 0.6047 | Q80U72 | 0.6255 |
|  |  | O35593 | 0.6047 | Q61191 | 0.6254 |
|  |  | Q9D7S7 | 0.6046 | E9Q616 | 0.6254 |
|  |  | Q61136 | 0.6045 | D6RHA2 | 0.6254 |
|  |  | Q9DB77 | 0.6045 | P14576 | 0.6254 |
|  |  | F6SQH7 | 0.6045 | E9Q6A3 | 0.6253 |
|  |  | Q99JI4 | 0.6042 | Q6PJ18 | 0.6252 |
|  |  | P62334 | 0.6040 | Q8BU30 | 0.6251 |
|  |  | F6SPK0 | 0.6037 | A0A068BGR9 | 0.6250 |
|  |  | P97370 | 0.6037 | Q9R233 | 0.6248 |
|  |  | Q1KYM0 | 0.6037 | P35700 | 0.6247 |
|  |  | Q8K2B3 | 0.6034 | Q3U4W8 | 0.6247 |
|  |  | P08249 | 0.6030 | Q3TT85 | 0.6246 |
|  |  | Q8VE10 | 0.6029 | Q9D1I2 | 0.6245 |
|  |  | Q3U7R1 | 0.6029 | A2AWI9 | 0.6245 |
|  |  | Q80WJ7 | 0.6028 | Q9ERU9 | 0.6244 |
|  |  | O08759 | 0.6025 | Q60710 | 0.6240 |
|  |  | P80313 | 0.6024 | Q3TYS2 | 0.6240 |
|  |  | E9PYA3 | 0.6023 | H3BKN0 | 0.6239 |
|  |  | Q9D338 | 0.6013 | P14685 | 0.6239 |
|  |  | Q8CC03 | 0.6013 | Q3TB65 | 0.6238 |
|  |  | H7BX88 | 0.6011 | P35282 | 0.6238 |
|  |  | O55029 | 0.6007 | P61967 | 0.6238 |
|  |  | Q3TIU7 | 0.6006 | Q6P5B0 | 0.6237 |
|  |  | Q02053 | 0.6006 | Q8R2Y8 | 0.6236 |
|  |  | Q924A9 | 0.6001 | P62774 | 0.6236 |
|  |  | Q9CY49 | 0.6000 | P24288 | 0.6234 |
|  |  | D3Z061 | 0.5999 | Q3THW7 | 0.6233 |
|  |  | Q3UQU5 | 0.5998 | P49722 | 0.6233 |
|  |  | Q3V3N5 | 0.5996 | O35459 | 0.6231 |
|  |  | P63037 | 0.5996 | D3Z4V1 | 0.6231 |
|  |  | P47911 | 0.5994 | A0A0A6YX73 | 0.6230 |
|  |  | Q3UGN9 | 0.5992 | Q8R016 | 0.6229 |
|  |  | Q9WUM5 | 0.5991 | Q8BWT1 | 0.6229 |
|  |  | Q06EZ3 | 0.5989 | Q80YW9 | 0.6226 |
|  |  | P81117 | 0.5989 | P11157 | 0.6225 |
|  |  | Q8C1V4 | 0.5988 | Q8BI84 | 0.6224 |
|  |  | Q68FL6 | 0.5987 | B9EKJ3 | 0.6224 |
|  |  | Q6ZQ58 | 0.5986 | Q91WM3 | 0.6223 |
|  |  | Q8BTF0 | 0.5979 | A0A0A0MQM0 | 0.6223 |
|  |  | Q8CIG8 | 0.5978 | Q922A3 | 0.6223 |
|  |  | P21981 | 0.5976 | Q91WK1 | 0.6222 |
|  |  | Q01320 | 0.5975 | E9QQ10 | 0.6221 |
|  |  | Q9WVS5 | 0.5974 | Q3TJF2 | 0.6220 |
|  |  | Q9EQQ2 | 0.5969 | Q9JLI8 | 0.6220 |
|  |  | P27048 | 0.5965 | Q1WWK3 | 0.6218 |
|  |  | Q9DCC4 | 0.5964 | Q99JR1 | 0.6218 |
|  |  | Q8CGK3 | 0.5959 | Q3TJZ7 | 0.6217 |
|  |  | Q99PV0 | 0.5959 | Q3U9H4 | 0.6216 |
|  |  | Q61166 | 0.5956 | O89079 | 0.6216 |
|  |  | Q8VI75 | 0.5951 | Q9QYR9 | 0.6215 |
|  |  | Q3UEB3 | 0.5949 | P40336 | 0.6213 |
|  |  | P70279 | 0.5949 | G5E8G0 | 0.6211 |
|  |  | P62075 | 0.5946 | Q9D903 | 0.6211 |
|  |  | A2AE27 | 0.5944 | E9PYA3 | 0.6210 |
|  |  | Q3V2N5 | 0.5944 | Q8VBV7 | 0.6209 |
|  |  | Q9CPQ8 | 0.5944 | Q80UK4 | 0.6209 |
|  |  | P19783 | 0.5944 | Q91Z53 | 0.6207 |
|  |  | P51174 | 0.5943 | Q99LX0 | 0.6206 |
|  |  | Q3UXP2 | 0.5940 | Q8K2C6 | 0.6206 |
|  |  | Q9D903 | 0.5938 | Q3UIJ2 | 0.6205 |
|  |  | P27612 | 0.5936 | Q3TJH1 | 0.6205 |
|  |  | D3Z074 | 0.5935 | P14131 | 0.6204 |
|  |  | Q3THA0 | 0.5933 | Q3TA68 | 0.6204 |
|  |  | Q8K363 | 0.5932 | D3YU17 | 0.6204 |
|  |  | G3X9V2 | 0.5931 | Q9R0E1 | 0.6203 |
|  |  | A2AP32 | 0.5930 | Q6ZQ58 | 0.6203 |
|  |  | Q9EPL8 | 0.5930 | B9EKT6 | 0.6203 |
|  |  | P80318 | 0.5929 | Q6PGL7 | 0.6202 |
|  |  | P05132 | 0.5928 | Q6ZQH8 | 0.6201 |
|  |  | Q99LF5 | 0.5925 | P19324 | 0.6199 |
|  |  | Q5D0F3 | 0.5922 | P56389 | 0.6199 |
|  |  | P83887 | 0.5919 | O08529 | 0.6198 |
|  |  | P52624 | 0.5918 | Q9Z1Q5 | 0.6198 |
|  |  | Q3ULZ3 | 0.5916 | P45591 | 0.6196 |
|  |  | P62880 | 0.5916 | Q921F4 | 0.6196 |
|  |  | Q3U9A8 | 0.5914 | Q3TSX5 | 0.6195 |
|  |  | P97494 | 0.5913 | P63101 | 0.6193 |
|  |  | Q9CQF0 | 0.5909 | Q3U7A6 | 0.6192 |
|  |  | Q6IRT4 | 0.5909 | E9Q6U4 | 0.6191 |
|  |  | Q8CJ53 | 0.5908 | A2ABY3 | 0.6189 |
|  |  | B2RY51 | 0.5907 | P09671 | 0.6187 |
|  |  | A0A0B4J1F2 | 0.5906 | Q8VC85 | 0.6185 |
|  |  | Q9Z0F7 | 0.5901 | Q8BH79 | 0.6183 |
|  |  | P31938 | 0.5899 | P07901 | 0.6183 |
|  |  | Q3TCH7 | 0.5896 | E9PUE7 | 0.6182 |
|  |  | Q6ZQ38 | 0.5895 | Q3UJR8 | 0.6182 |
|  |  | Q68FD5 | 0.5893 | H3BKE1 | 0.6182 |
|  |  | Q9CQH7 | 0.5893 | Q99KR3 | 0.6182 |
|  |  | Q3TFQ8 | 0.5891 | Q9D8S3 | 0.6181 |
|  |  | Q9JKR6 | 0.5890 | P10639 | 0.6180 |
|  |  | Q3UFY8 | 0.5888 | Q8C1V4 | 0.6180 |
|  |  | P50544 | 0.5887 | Q8BL66 | 0.6179 |
|  |  | Q9DC23 | 0.5883 | P62862 | 0.6179 |
|  |  | P70296 | 0.5881 | Q3TGW0 | 0.6178 |
|  |  | Q8BH86 | 0.5880 | P50543 | 0.6178 |
|  |  | P28660 | 0.5880 | H7BX88 | 0.6178 |
|  |  | Q3UE92 | 0.5878 | Q9CSH3 | 0.6177 |
|  |  | P35762 | 0.5876 | O70251 | 0.6177 |
|  |  | Q8C660 | 0.5873 | Q3TXN0 | 0.6177 |
|  |  | Q6A0A9 | 0.5873 | P68510 | 0.6175 |
|  |  | Q3TMB5 | 0.5869 | Q8BIZ9 | 0.6175 |
|  |  | P47955 | 0.5868 | Q6PAM1 | 0.6174 |
|  |  | J3QQ30 | 0.5862 | Q3UF75 | 0.6173 |
|  |  | Q3TXN6 | 0.5854 | E9Q6R3 | 0.6173 |
|  |  | P12787 | 0.5853 | P08752 | 0.6173 |
|  |  | Q3TQY2 | 0.5851 | P56376 | 0.6172 |
|  |  | Q2VPC9 | 0.5849 | D3Z645 | 0.6172 |
|  |  | Q00612 | 0.5847 | Q9D1I6 | 0.6171 |
|  |  | Q3TAV1 | 0.5846 | Q6PB52 | 0.6171 |
|  |  | Q9DCV4 | 0.5846 | Q8K239 | 0.6171 |
|  |  | Q5SS83 | 0.5844 | P35762 | 0.6170 |
|  |  | A0A0A6YWP9 | 0.5841 | Q62426 | 0.6169 |
|  |  | S4R1L5 | 0.5836 | Q3TIV5 | 0.6169 |
|  |  | P50247 | 0.5832 | Q3T9L0 | 0.6168 |
|  |  | B2CY77 | 0.5832 | G5E884 | 0.6167 |
|  |  | G5E866 | 0.5830 | Q3TPD9 | 0.6166 |
|  |  | Q5M9L1 | 0.5830 | Q3UM23 | 0.6165 |
|  |  | Q5JC28 | 0.5830 | P70122 | 0.6165 |
|  |  | P10711 | 0.5827 | Q9CXJ1 | 0.6165 |
|  |  | Q9CQC7 | 0.5826 | P60122 | 0.6165 |
|  |  | P60122 | 0.5822 | Q9CXW3 | 0.6164 |
|  |  | Q3V117 | 0.5821 | Q9JHI5 | 0.6164 |
|  |  | Q3TT85 | 0.5819 | Q8CG76 | 0.6163 |
|  |  | Q3UZ39 | 0.5819 | Q8R1V4 | 0.6163 |
|  |  | Q91W96 | 0.5816 | P63038 | 0.6162 |
|  |  | P45952 | 0.5814 | Q5RKP4 | 0.6162 |
|  |  | D3Z0A2 | 0.5814 | O54941 | 0.6162 |
|  |  | Q4FZL1 | 0.5813 | Q5SVG5 | 0.6160 |
|  |  | Q6NXL1 | 0.5810 | Q542W3 | 0.6160 |
|  |  | Q8BKZ9 | 0.5809 | Q8R5C0 | 0.6160 |
|  |  | Q9D0F3 | 0.5809 | Q9CQH7 | 0.6158 |
|  |  | P58021 | 0.5808 | Q99L45 | 0.6157 |
|  |  | Q925I1 | 0.5805 | Q921I9 | 0.6151 |
|  |  | Q8C4B4 | 0.5803 | Q64511 | 0.6150 |
|  |  | Q3UHX2 | 0.5798 | Q64435 | 0.6149 |
|  |  | P62317 | 0.5795 | F8WHU7 | 0.6148 |
|  |  | P00375 | 0.5795 | Q8C570 | 0.6147 |
|  |  | Q9JHU4 | 0.5790 | Q5RL55 | 0.6145 |
|  |  | Q3TM89 | 0.5790 | Q9CQ71 | 0.6144 |
|  |  | E9Q715 | 0.5790 | A2AQE4 | 0.6143 |
|  |  | Q03958 | 0.5787 | Q3ULI5 | 0.6143 |
|  |  | Q9Z2I0 | 0.5781 | Q68FD5 | 0.6141 |
|  |  | E9QAS4 | 0.5780 | P60487 | 0.6141 |
|  |  | Q3TIC8 | 0.5779 | Q9CXJ4 | 0.6141 |
|  |  | Q91WS0 | 0.5778 | Q8BJW6 | 0.6140 |
|  |  | Q921L3 | 0.5777 | Q9CYN2 | 0.6140 |
|  |  | P05622 | 0.5776 | Q6P5D8 | 0.6140 |
|  |  | Q6P4T2 | 0.5774 | G5E866 | 0.6137 |
|  |  | P17918 | 0.5774 | Q9QZQ8 | 0.6137 |
|  |  | P14576 | 0.5772 | P14733 | 0.6136 |
|  |  | P62137 | 0.5771 | B7ZWC4 | 0.6136 |
|  |  | B9EHZ5 | 0.5769 | P62874 | 0.6136 |
|  |  | Q6PJ18 | 0.5767 | D3Z0F3 | 0.6135 |
|  |  | P19324 | 0.5766 | E9Q1S3 | 0.6134 |
|  |  | Q01853 | 0.5763 | Q8BZA9 | 0.6134 |
|  |  | P56391 | 0.5760 | O08614 | 0.6133 |
|  |  | Q64433 | 0.5760 | Q78IK4 | 0.6133 |
|  |  | Q62186 | 0.5760 | Q91Z49 | 0.6133 |
|  |  | Q3URM4 | 0.5759 | Q3THA6 | 0.6133 |
|  |  | P14824 | 0.5758 | P11983 | 0.6131 |
|  |  | B2RY56 | 0.5756 | Q3TW51 | 0.6131 |
|  |  | Q9D0I8 | 0.5756 | P47962 | 0.6130 |
|  |  | Q3UW53 | 0.5753 | Q91VC3 | 0.6129 |
|  |  | P62754 | 0.5751 | Q7TSB1 | 0.6129 |
|  |  | J3QMC5 | 0.5751 | Q9DBZ5 | 0.6128 |
|  |  | Q5RKP4 | 0.5751 | P27048 | 0.6128 |
|  |  | Q8R0M2 | 0.5750 | Q9CQ48 | 0.6126 |
|  |  | Q6A068 | 0.5750 | Q8K1N4 | 0.6126 |
|  |  | A2AQE4 | 0.5748 | Q7TMB8 | 0.6126 |
|  |  | Q3UPL0 | 0.5747 | Q61941 | 0.6126 |
|  |  | P61222 | 0.5746 | Q00422 | 0.6125 |
|  |  | P61358 | 0.5743 | Q9QUR6 | 0.6125 |
|  |  | Q08093 | 0.5742 | Q9CQS8 | 0.6125 |
|  |  | D3Z0F5 | 0.5741 | P53994 | 0.6124 |
|  |  | A0A0A6YW80 | 0.5739 | Q3UDE2 | 0.6124 |
|  |  | E9PXX7 | 0.5738 | Q9CQF0 | 0.6124 |
|  |  | Q9DBS1 | 0.5737 | Q8BTF0 | 0.6122 |
|  |  | Q8BHL5 | 0.5737 | Q9Z1F9 | 0.6122 |
|  |  | Q80UZ2 | 0.5736 | Q6R891 | 0.6122 |
|  |  | P46737 | 0.5734 | P99028 | 0.6121 |
|  |  | Q8VC85 | 0.5733 | Q8BHD7 | 0.6121 |
|  |  | Q61733 | 0.5729 | Q8BGP6 | 0.6121 |
|  |  | P00493 | 0.5727 | Q91VR5 | 0.6120 |
|  |  | O35295 | 0.5727 | P56391 | 0.6120 |
|  |  | O70310 | 0.5725 | Q3U281 | 0.6119 |
|  |  | J3QPZ8 | 0.5723 | Q60973 | 0.6119 |
|  |  | O70475 | 0.5722 | Q9QYS9 | 0.6118 |
|  |  | P99027 | 0.5721 | Q9WU62 | 0.6116 |
|  |  | Q2L4X1 | 0.5721 | Q3U3I6 | 0.6116 |
|  |  | Q61699 | 0.5717 | P15626 | 0.6116 |
|  |  | Q3TUW9 | 0.5714 | Q3TJA9 | 0.6116 |
|  |  | Q3U0I9 | 0.5712 | P54227 | 0.6115 |
|  |  | Q61927 | 0.5711 | Q9CXR1 | 0.6115 |
|  |  | Q7TPV4 | 0.5705 | Q8C1A5 | 0.6114 |
|  |  | A0A068BIU7 | 0.5703 | Z4YKC4 | 0.6113 |
|  |  | P80316 | 0.5703 | P03958 | 0.6113 |
|  |  | Q8VEE4 | 0.5702 | H3BLI9 | 0.6113 |
|  |  | Q99KQ4 | 0.5699 | Q9D0B6 | 0.6111 |
|  |  | Q8BFQ8 | 0.5694 | P97315 | 0.6110 |
|  |  | Q9CQV8 | 0.5690 | A2ATT5 | 0.6110 |
|  |  | Q7TT37 | 0.5690 | P35550 | 0.6109 |
|  |  | Q62145 | 0.5688 | P19182 | 0.6109 |
|  |  | Q3TGW0 | 0.5686 | Q640N1 | 0.6109 |
|  |  | A2RRK3 | 0.5686 | Q61074 | 0.6109 |
|  |  | Q99PT1 | 0.5685 | Q8R2U6 | 0.6108 |
|  |  | Q60597 | 0.5681 | Q61733 | 0.6106 |
|  |  | Q9JI13 | 0.5680 | Q02819 | 0.6105 |
|  |  | P40142 | 0.5680 | P34022 | 0.6105 |
|  |  | Q6NV83 | 0.5677 | P58389 | 0.6104 |
|  |  | Q8VDD5 | 0.5677 | P11352 | 0.6103 |
|  |  | G3UX26 | 0.5675 | P43247 | 0.6102 |
|  |  | P19182 | 0.5674 | E9Q7G0 | 0.6101 |
|  |  | Q3T9L0 | 0.5673 | P80313 | 0.6100 |
|  |  | Q8R3X4 | 0.5673 | Q7TPV4 | 0.6100 |
|  |  | Q9R118 | 0.5673 | G5E8J3 | 0.6098 |
|  |  | P05202 | 0.5669 | K3W4Q8 | 0.6098 |
|  |  | P51660 | 0.5665 | A2CG35 | 0.6098 |
|  |  | Q8VDG8 | 0.5662 | Q9D1D4 | 0.6098 |
|  |  | Q8BGS2 | 0.5659 | F7ACR9 | 0.6097 |
|  |  | Q9D2R8 | 0.5657 | Q9CU62 | 0.6096 |
|  |  | P27659 | 0.5654 | Q99LB6 | 0.6095 |
|  |  | Q99LX0 | 0.5654 | Q9QXT0 | 0.6093 |
|  |  | P49722 | 0.5654 | Q9CSC2 | 0.6092 |
|  |  | Q3TTX0 | 0.5646 | E9Q5I9 | 0.6092 |
|  |  | P07901 | 0.5646 | Q3UAZ7 | 0.6091 |
|  |  | Q8C1E7 | 0.5645 | V9GXJ1 | 0.6090 |
|  |  | Q8BMS1 | 0.5644 | Q8BP48 | 0.6090 |
|  |  | D3YTP0 | 0.5640 | Q6SLK2 | 0.6089 |
|  |  | Q60605 | 0.5639 | Q91YE7 | 0.6086 |
|  |  | Q8C1B7 | 0.5638 | Q9CWJ9 | 0.6086 |
|  |  | Q04750 | 0.5637 | P08003 | 0.6086 |
|  |  | Q9JJ89 | 0.5636 | E9PXV3 | 0.6086 |
|  |  | Q8VCW8 | 0.5635 | Q8BTB8 | 0.6085 |
|  |  | Q9CUB4 | 0.5635 | A2RRK3 | 0.6085 |
|  |  | P63001 | 0.5632 | Q3UQU5 | 0.6084 |
|  |  | Q9D517 | 0.5631 | Q8BH95 | 0.6084 |
|  |  | Q9R1P4 | 0.5625 | P70296 | 0.6084 |
|  |  | Q8BU30 | 0.5623 | Q3UTE4 | 0.6083 |
|  |  | Q9CQM9 | 0.5620 | Q9D6Y7 | 0.6083 |
|  |  | B2RY90 | 0.5618 | Q3TJG6 | 0.6081 |
|  |  | E9QAZ2 | 0.5616 | Q9CPU0 | 0.6081 |
|  |  | Q9WVG6 | 0.5616 | P60670 | 0.6080 |
|  |  | Q3TW51 | 0.5616 | Q3UF82 | 0.6080 |
|  |  | Q80UK8 | 0.5616 | Q3TEZ2 | 0.6080 |
|  |  | O54774 | 0.5616 | P59708 | 0.6080 |
|  |  | Q8BX02 | 0.5613 | Q9DAR7 | 0.6079 |
|  |  | Q9WVA3 | 0.5613 | Q9ER72 | 0.6077 |
|  |  | Q9R257 | 0.5613 | Q93092 | 0.6075 |
|  |  | Q9D6E8 | 0.5611 | Q921X9 | 0.6075 |
|  |  | Q3TJZ7 | 0.5608 | P31230 | 0.6074 |
|  |  | Q8BP48 | 0.5606 | Q8K1R7 | 0.6074 |
|  |  | Q8R3C0 | 0.5606 | Q9D136 | 0.6074 |
|  |  | B2RUJ7 | 0.5606 | Q3U5R8 | 0.6074 |
|  |  | P42125 | 0.5606 | Q3U417 | 0.6073 |
|  |  | Q6PB51 | 0.5605 | A0A068BFR3 | 0.6073 |
|  |  | Q8CHW4 | 0.5603 | Q8CFI7 | 0.6072 |
|  |  | P67778 | 0.5600 | Q8BU33 | 0.6071 |
|  |  | Q05D44 | 0.5599 | A4FUV9 | 0.6071 |
|  |  | Q9CZT6 | 0.5598 | P62748 | 0.6071 |
|  |  | Q61941 | 0.5592 | Q9QXE7 | 0.6070 |
|  |  | Q3TG21 | 0.5592 | Q8VIM9 | 0.6070 |
|  |  | Q9CS42 | 0.5591 | Q505F5 | 0.6069 |
|  |  | P11438 | 0.5590 | P61089 | 0.6069 |
|  |  | Q9CWZ5 | 0.5589 | Q3TLI6 | 0.6068 |
|  |  | Q3U868 | 0.5589 | P50544 | 0.6068 |
|  |  | Q3TJF2 | 0.5587 | Q921E2 | 0.6067 |
|  |  | Q9Z1T1 | 0.5585 | Q3UID0 | 0.6067 |
|  |  | E9PWQ3 | 0.5582 | Q9QZE5 | 0.6066 |
|  |  | A2AWT6 | 0.5579 | Q9Z2I8 | 0.6066 |
|  |  | Q9CPP6 | 0.5579 | G3X9B1 | 0.6064 |
|  |  | P97352 | 0.5579 | Q810A7 | 0.6063 |
|  |  | P26638 | 0.5578 | Q3UXP2 | 0.6061 |
|  |  | V9GXJ1 | 0.5578 | Q8CGK3 | 0.6060 |
|  |  | Q8CGP5 | 0.5578 | P05202 | 0.6059 |
|  |  | P32067 | 0.5576 | P84089 | 0.6059 |
|  |  | O08663 | 0.5575 | P24369 | 0.6059 |
|  |  | Q5SYD0 | 0.5568 | Q8R2M2 | 0.6058 |
|  |  | Q3ULG4 | 0.5565 | P97390 | 0.6057 |
|  |  | Q9D0T1 | 0.5565 | Q3TFA9 | 0.6057 |
|  |  | Q80TX7 | 0.5558 | O70591 | 0.6056 |
|  |  | Q3TBW2 | 0.5557 | G5E8C4 | 0.6055 |
|  |  | Q9R0X4 | 0.5556 | P47856 | 0.6053 |
|  |  | Q8CE21 | 0.5555 | Q3URM4 | 0.6053 |
|  |  | P21550 | 0.5553 | Q14DR9 | 0.6053 |
|  |  | E9Q9E1 | 0.5549 | Q80YG4 | 0.6053 |
|  |  | Q3TV20 | 0.5549 | Q3ULJ0 | 0.6052 |
|  |  | P62242 | 0.5547 | P26043 | 0.6052 |
|  |  | P29391 | 0.5546 | F6YMR0 | 0.6051 |
|  |  | P70349 | 0.5545 | E9Q4B9 | 0.6051 |
|  |  | Q99LC2 | 0.5539 | F8VPK0 | 0.6050 |
|  |  | Q9DBE9 | 0.5539 | B1AT03 | 0.6050 |
|  |  | P62908 | 0.5538 | Q6DI95 | 0.6050 |
|  |  | A0A0A6YVU8 | 0.5536 | Q9CY64 | 0.6050 |
|  |  | Q8R1Q8 | 0.5536 | A2A5V3 | 0.6050 |
|  |  | Q8BFY9 | 0.5535 | Q3TD41 | 0.6049 |
|  |  | E9QAH1 | 0.5529 | Q3TFQ8 | 0.6049 |
|  |  | Q3U1J4 | 0.5528 | Q9D8W6 | 0.6048 |
|  |  | Q3V1L7 | 0.5528 | Q80WJ7 | 0.6047 |
|  |  | Q8R0R0 | 0.5526 | P83887 | 0.6047 |
|  |  | P08113 | 0.5525 | Q9EQK5 | 0.6047 |
|  |  | Q8C1A5 | 0.5523 | P61924 | 0.6047 |
|  |  | P31230 | 0.5523 | O35382 | 0.6047 |
|  |  | Q8VI84 | 0.5523 | Q91VR8 | 0.6046 |
|  |  | Q8BTB8 | 0.5519 | Q5F2E7 | 0.6046 |
|  |  | Q91VC3 | 0.5517 | P62830 | 0.6046 |
|  |  | Q9ESP1 | 0.5514 | Q8K2B0 | 0.6046 |
|  |  | Q9DBR1 | 0.5512 | Q64521 | 0.6045 |
|  |  | P62717 | 0.5508 | Q9D6S7 | 0.6044 |
|  |  | Q6PIP5 | 0.5503 | Q3TEL0 | 0.6043 |
|  |  | E9PWY9 | 0.5500 | D3YVV4 | 0.6043 |
|  |  | Q71RI9 | 0.5500 | P16045 | 0.6042 |
|  |  | P10649 | 0.5499 | Q7TMI0 | 0.6042 |
|  |  | Q9JKF1 | 0.5499 | Q8CIN4 | 0.6041 |
|  |  | Q91YR7 | 0.5497 | Q3TFE8 | 0.6041 |
|  |  | Q3TI79 | 0.5497 | Q8VBV3 | 0.6041 |
|  |  | P09411 | 0.5496 | Q9CQU0 | 0.6040 |
|  |  | Q3TJ01 | 0.5495 | Q3TXN6 | 0.6040 |
|  |  | Q9JJ28 | 0.5494 | A0A087WQQ5 | 0.6040 |
|  |  | O88398 | 0.5493 | Q5PRF0 | 0.6039 |
|  |  | O55222 | 0.5492 | Q921E4 | 0.6038 |
|  |  | A0A0A6YW28 | 0.5491 | Q99MR6 | 0.6037 |
|  |  | Q99JY0 | 0.5487 | Q8BWM0 | 0.6037 |
|  |  | P08003 | 0.5486 | B2RQQ5 | 0.6036 |
|  |  | A0A087WNZ7 | 0.5485 | P60710 | 0.6034 |
|  |  | Q78JE5 | 0.5483 | Q3UGJ7 | 0.6034 |
|  |  | D3YWK1 | 0.5481 | Q61833 | 0.6034 |
|  |  | Q9WV60 | 0.5479 | Q9EQ06 | 0.6033 |
|  |  | P63276 | 0.5477 | P12815 | 0.6033 |
|  |  | Q45VK5 | 0.5474 | Q8R2Y2 | 0.6033 |
|  |  | Q3TXS9 | 0.5474 | Q8CAQ8 | 0.6032 |
|  |  | P30412 | 0.5472 | A2ALB2 | 0.6032 |
|  |  | P62196 | 0.5472 | Q80X90 | 0.6032 |
|  |  | Q3TG12 | 0.5470 | Q9CQJ2 | 0.6032 |
|  |  | P62270 | 0.5470 | Q9D7E4 | 0.6032 |
|  |  | Q3UMT7 | 0.5469 | Q6NZN9 | 0.6030 |
|  |  | Q9CT23 | 0.5468 | A0A068BGU5 | 0.6030 |
|  |  | Q9DCA5 | 0.5466 | Q6PB44 | 0.6029 |
|  |  | P70122 | 0.5465 | Q3U7R1 | 0.6029 |
|  |  | P38647 | 0.5463 | Q3TWN8 | 0.6028 |
|  |  | Q8CIN4 | 0.5462 | Q61699 | 0.6026 |
|  |  | D3Z637 | 0.5460 | Q3TXS7 | 0.6026 |
|  |  | P17426 | 0.5456 | Q8BG51 | 0.6026 |
|  |  | B7ZWL1 | 0.5453 | Q62376 | 0.6025 |
|  |  | Q3U6D2 | 0.5451 | Q00PI9 | 0.6025 |
|  |  | Q3USG5 | 0.5451 | Q9Z277 | 0.6025 |
|  |  | Q6NVF9 | 0.5451 | Q3U0V1 | 0.6025 |
|  |  | D3YWF6 | 0.5449 | Q91W96 | 0.6024 |
|  |  | Q3UXI9 | 0.5443 | Q9QYB1 | 0.6024 |
|  |  | Q9JKB3 | 0.5443 | P23591 | 0.6024 |
|  |  | D3Z3F8 | 0.5442 | Q8JZQ1 | 0.6023 |
|  |  | Q9WUP7 | 0.5441 | O35864 | 0.6022 |
|  |  | Q7TNV0 | 0.5440 | Q9D172 | 0.6021 |
|  |  | Q9CRF5 | 0.5437 | Q06185 | 0.6021 |
|  |  | Q8BP47 | 0.5436 | P10833 | 0.6020 |
|  |  | Q99MR6 | 0.5431 | Q5SF07 | 0.6019 |
|  |  | Q9WU28 | 0.5430 | Q8BTW3 | 0.6019 |
|  |  | Q8BVA5 | 0.5428 | P80316 | 0.6019 |
|  |  | Q8C5B5 | 0.5427 | D6REF7 | 0.6019 |
|  |  | P63038 | 0.5427 | Q0KL02 | 0.6018 |
|  |  | Q9D6R2 | 0.5426 | Q91YX7 | 0.6018 |
|  |  | Q8BHA3 | 0.5426 | Q8R0W0 | 0.6018 |
|  |  | Q9DAW9 | 0.5426 | Q9WU28 | 0.6017 |
|  |  | Q3TZU7 | 0.5422 | P62814 | 0.6016 |
|  |  | Q91YS7 | 0.5421 | P26516 | 0.6015 |
|  |  | Q7TQH0 | 0.5420 | Q3TE06 | 0.6015 |
|  |  | P24288 | 0.5418 | P30416 | 0.6015 |
|  |  | Q3UA17 | 0.5418 | Q6ZPJ3 | 0.6014 |
|  |  | Q9EP72 | 0.5412 | Q60597 | 0.6014 |
|  |  | P23116 | 0.5412 | Q3THP1 | 0.6014 |
|  |  | P54775 | 0.5410 | P32067 | 0.6014 |
|  |  | Q9Z2I8 | 0.5410 | Q3TN29 | 0.6014 |
|  |  | Q9Z2X1 | 0.5409 | Q9D7V6 | 0.6013 |
|  |  | Q9D1H7 | 0.5404 | P63168 | 0.6013 |
|  |  | Q3TE06 | 0.5402 | P63028 | 0.6012 |
|  |  | A0A087WQQ5 | 0.5401 | Q3TN31 | 0.6012 |
|  |  | Q78XF5 | 0.5401 | P59016 | 0.6011 |
|  |  | Q3U3C4 | 0.5395 | Q95457 | 0.6010 |
|  |  | Q7TMI0 | 0.5392 | Q8R3F5 | 0.6010 |
|  |  | A2AMW0 | 0.5391 | Q9QZB7 | 0.6010 |
|  |  | Q8K411 | 0.5387 | Q3TAW4 | 0.6010 |
|  |  | Q9JHW2 | 0.5386 | Q9CW03 | 0.6009 |
|  |  | P19096 | 0.5385 | Q8BU20 | 0.6009 |
|  |  | O35855 | 0.5384 | B9EIU1 | 0.6009 |
|  |  | F6RPJ9 | 0.5383 | P19157 | 0.6009 |
|  |  | Q9DBZ5 | 0.5382 | Q9CSZ8 | 0.6009 |
|  |  | B9EKT6 | 0.5374 | B2CY77 | 0.6008 |
|  |  | Q8BH59 | 0.5374 | Q3UPL0 | 0.6008 |
|  |  | Q4VA28 | 0.5374 | Q9WU78 | 0.6006 |
|  |  | Q9ERU9 | 0.5373 | Q3UL43 | 0.6006 |
|  |  | P26043 | 0.5365 | Q61599 | 0.6005 |
|  |  | Q6P8N8 | 0.5364 | P97770 | 0.6005 |
|  |  | Q60598 | 0.5364 | P59017 | 0.6005 |
|  |  | Q99K70 | 0.5361 | A1L2Z3 | 0.6004 |
|  |  | Q3ULP8 | 0.5358 | Q8R4R6 | 0.6003 |
|  |  | P08074 | 0.5358 | Q3URN5 | 0.6003 |
|  |  | Q8CHC7 | 0.5355 | Q78ZA7 | 0.6002 |
|  |  | A0A0A6YX73 | 0.5349 | Q8C1B7 | 0.6002 |
|  |  | P10630 | 0.5346 | Q9WUD1 | 0.6002 |
|  |  | Q8R502 | 0.5346 | Q9JKF1 | 0.6001 |
|  |  | P70388 | 0.5345 | A7VJ98 | 0.6001 |
|  |  | Q9DBU8 | 0.5345 | P27659 | 0.6000 |
|  |  | B1AU25 | 0.5344 | P26883 | 0.6000 |
|  |  | B2RQS1 | 0.5343 | Q9CSN5 | 0.6000 |
|  |  | O35387 | 0.5340 | P21619 | 0.5999 |
|  |  | P40124 | 0.5339 | Q9DBP5 | 0.5999 |
|  |  | Q3UYD0 | 0.5337 | Q9R1P0 | 0.5998 |
|  |  | P35282 | 0.5337 | Q9JJE7 | 0.5998 |
|  |  | P18760 | 0.5335 | Q3U2G2 | 0.5998 |
|  |  | P11983 | 0.5335 | Q9R0X4 | 0.5998 |
|  |  | Q9R1C7 | 0.5334 | Q7TMH5 | 0.5996 |
|  |  | O88544 | 0.5333 | P60867 | 0.5995 |
|  |  | O54833 | 0.5332 | E9QAJ9 | 0.5993 |
|  |  | Q3UT19 | 0.5328 | P70372 | 0.5993 |
|  |  | P10852 | 0.5327 | Q99LS5 | 0.5993 |
|  |  | Q9CPU0 | 0.5327 | Q9EQQ9 | 0.5992 |
|  |  | Q9CTR1 | 0.5327 | A2AHX9 | 0.5992 |
|  |  | Q80UE5 | 0.5323 | P62259 | 0.5991 |
|  |  | Q3TW28 | 0.5321 | Q9DCA5 | 0.5991 |
|  |  | P60867 | 0.5319 | E3SRG7 | 0.5991 |
|  |  | Q3UJ70 | 0.5309 | M0QWY0 | 0.5991 |
|  |  | Q61171 | 0.5308 | Q920L1 | 0.5991 |
|  |  | Q9JJ94 | 0.5307 | E9QME5 | 0.5990 |
|  |  | Q9DCA4 | 0.5297 | Q99K51 | 0.5989 |
|  |  | F8VPX1 | 0.5292 | P68254 | 0.5989 |
|  |  | Q8BWZ3 | 0.5288 | P46471 | 0.5989 |
|  |  | F6ZFT1 | 0.5288 | Q3TQY2 | 0.5989 |
|  |  | F8WGB3 | 0.5285 | E9Q368 | 0.5988 |
|  |  | Q8BL66 | 0.5283 | B1AY13 | 0.5988 |
|  |  | P97742 | 0.5280 | Q3TSZ4 | 0.5988 |
|  |  | Q3UFR4 | 0.5278 | Q99LP6 | 0.5987 |
|  |  | Q9CX34 | 0.5277 | P47911 | 0.5986 |
|  |  | P26516 | 0.5276 | Q8BPB0 | 0.5986 |
|  |  | Q9WVQ5 | 0.5276 | Q6GQT9 | 0.5986 |
|  |  | Q62422 | 0.5274 | A0A087WRU0 | 0.5985 |
|  |  | Q3U0B0 | 0.5273 | P11031 | 0.5985 |
|  |  | Q9D8N0 | 0.5272 | Q9JI10 | 0.5984 |
|  |  | Q3UDX4 | 0.5271 | P14069 | 0.5984 |
|  |  | R4H4V1 | 0.5268 | G3X934 | 0.5984 |
|  |  | Q9CU62 | 0.5264 | Q8R323 | 0.5984 |
|  |  | Q3UVI9 | 0.5263 | Q8K3W0 | 0.5983 |
|  |  | P16460 | 0.5259 | P38647 | 0.5983 |
|  |  | Q9D1M7 | 0.5257 | P31324 | 0.5983 |
|  |  | Q9CPW4 | 0.5256 | Q3UFY8 | 0.5982 |
|  |  | Q3TXJ4 | 0.5254 | P48758 | 0.5982 |
|  |  | Q9WUD1 | 0.5252 | Q922R9 | 0.5981 |
|  |  | Q8BSL7 | 0.5252 | Q2L4X1 | 0.5981 |
|  |  | Q9EQK5 | 0.5250 | P26443 | 0.5981 |
|  |  | Q9D051 | 0.5249 | Q9D020 | 0.5980 |
|  |  | Q8C1M0 | 0.5248 | Q9R118 | 0.5980 |
|  |  | P49710 | 0.5248 | G3X963 | 0.5980 |
|  |  | Q8K1J6 | 0.5245 | P28352 | 0.5980 |
|  |  | F8WHU9 | 0.5245 | Q8BSX8 | 0.5979 |
|  |  | Q921I9 | 0.5244 | O35435 | 0.5979 |
|  |  | Q62351 | 0.5244 | Q6PCM2 | 0.5979 |
|  |  | Q8BH79 | 0.5243 | Q921H8 | 0.5979 |
|  |  | P23506 | 0.5243 | O55201 | 0.5979 |
|  |  | P35550 | 0.5242 | Q9CZD3 | 0.5978 |
|  |  | Q9D8E6 | 0.5240 | O09131 | 0.5978 |
|  |  | P10107 | 0.5239 | Q99N93 | 0.5977 |
|  |  | Q3UUX9 | 0.5239 | Q9CYG7 | 0.5977 |
|  |  | Q68FC6 | 0.5233 | Q91V41 | 0.5976 |
|  |  | Q9DAR7 | 0.5230 | Q9D0I8 | 0.5976 |
|  |  | S4R1E5 | 0.5224 | Q99LJ6 | 0.5975 |
|  |  | O35685 | 0.5223 | P37913 | 0.5974 |
|  |  | P68040 | 0.5221 | E9Q390 | 0.5972 |
|  |  | Q9CRT8 | 0.5220 | Q9DBE9 | 0.5971 |
|  |  | P48758 | 0.5219 | D3Z637 | 0.5970 |
|  |  | O35215 | 0.5218 | Q3TYR7 | 0.5970 |
|  |  | P35279 | 0.5218 | Q9DC29 | 0.5968 |
|  |  | P54227 | 0.5215 | Q8K1J6 | 0.5966 |
|  |  | Q4FJY5 | 0.5211 | Q8BH86 | 0.5965 |
|  |  | D3YVW2 | 0.5211 | O35685 | 0.5964 |
|  |  | Q9QYB1 | 0.5206 | Q60520 | 0.5964 |
|  |  | O89079 | 0.5199 | Q3TFD0 | 0.5964 |
|  |  | P11031 | 0.5197 | Q3TK27 | 0.5964 |
|  |  | Q3V1M8 | 0.5196 | P70349 | 0.5963 |
|  |  | Q9CXW2 | 0.5194 | Q3TIC8 | 0.5963 |
|  |  | Q91WK1 | 0.5193 | Q9D880 | 0.5963 |
|  |  | P42669 | 0.5193 | P80314 | 0.5963 |
|  |  | B9EIU1 | 0.5190 | P62962 | 0.5963 |
|  |  | Q8K2C6 | 0.5189 | P17742 | 0.5962 |
|  |  | Q61656 | 0.5183 | Q3UJK2 | 0.5962 |
|  |  | P97807 | 0.5182 | Q3TL58 | 0.5962 |
|  |  | B7ZWF1 | 0.5178 | P62196 | 0.5961 |
|  |  | Q9D0I9 | 0.5177 | Q922F4 | 0.5961 |
|  |  | Q99LJ6 | 0.5175 | Q8BMZ7 | 0.5961 |
|  |  | Q9WU78 | 0.5174 | D3Z2F7 | 0.5960 |
|  |  | Q9Z130 | 0.5158 | Q6NS46 | 0.5958 |
|  |  | Q0VGU9 | 0.5151 | Q5XJY5 | 0.5956 |
|  |  | O70591 | 0.5144 | P97807 | 0.5956 |
|  |  | Q3TN29 | 0.5142 | Q9ESU6 | 0.5956 |
|  |  | G3UWE1 | 0.5139 | P83917 | 0.5955 |
|  |  | D3YUM1 | 0.5138 | Q3THI5 | 0.5955 |
|  |  | Q3TUI9 | 0.5137 | Q60605 | 0.5953 |
|  |  | Q62426 | 0.5136 | Q9D6N1 | 0.5953 |
|  |  | D3Z2J6 | 0.5136 | Z4YL78 | 0.5953 |
|  |  | Q3UL78 | 0.5130 | P99029 | 0.5952 |
|  |  | Q99KP6 | 0.5130 | Q3TZZ7 | 0.5952 |
|  |  | Q8BH80 | 0.5129 | Q9CQC7 | 0.5951 |
|  |  | Q9DCW4 | 0.5129 | A2AMW0 | 0.5950 |
|  |  | E9QKV6 | 0.5121 | P48428 | 0.5949 |
|  |  | Q3TN42 | 0.5121 | Q3TW28 | 0.5949 |
|  |  | G3X9B1 | 0.5120 | D3Z061 | 0.5948 |
|  |  | Q9DBG3 | 0.5120 | Q8VDF7 | 0.5948 |
|  |  | E9Q555 | 0.5119 | Q3USX2 | 0.5947 |
|  |  | P04117 | 0.5118 | P62852 | 0.5946 |
|  |  | Q3THB3 | 0.5116 | Q9Z1Z0 | 0.5946 |
|  |  | Q5SWU9 | 0.5114 | O35855 | 0.5946 |
|  |  | A2CG35 | 0.5111 | Q60931 | 0.5945 |
|  |  | Q8C2D1 | 0.5110 | O88544 | 0.5945 |
|  |  | Q6ZWZ6 | 0.5110 | D3Z7P3 | 0.5944 |
|  |  | P14869 | 0.5109 | P56480 | 0.5944 |
|  |  | P17427 | 0.5107 | Q3UZ39 | 0.5943 |
|  |  | Q9CQR4 | 0.5105 | Q5SSI6 | 0.5943 |
|  |  | P56376 | 0.5105 | Q8R0A0 | 0.5943 |
|  |  | Q3TXN0 | 0.5104 | Q3UMB9 | 0.5942 |
|  |  | Q9DB05 | 0.5101 | Q9CYH6 | 0.5942 |
|  |  | Q9CTT4 | 0.5101 | D3Z7C0 | 0.5942 |
|  |  | Q3TEU8 | 0.5100 | Q9WVE8 | 0.5941 |
|  |  | P70362 | 0.5099 | Q8BMJ3 | 0.5941 |
|  |  | O55143 | 0.5099 | P30412 | 0.5940 |
|  |  | Q9D0L7 | 0.5094 | Q80Y81 | 0.5939 |
|  |  | Q8C7C4 | 0.5089 | Q78XF5 | 0.5939 |
|  |  | P54822 | 0.5089 | Q8BVG3 | 0.5939 |
|  |  | Q9R062 | 0.5089 | Q9D1P4 | 0.5939 |
|  |  | E0CXN5 | 0.5089 | Q6P5F9 | 0.5939 |
|  |  | Q3THW7 | 0.5086 | Q8VE47 | 0.5939 |
|  |  | Q3TXV1 | 0.5083 | Q3TXV1 | 0.5938 |
|  |  | Q6ZWU9 | 0.5077 | P61358 | 0.5937 |
|  |  | Q3TFF0 | 0.5070 | P61961 | 0.5936 |
|  |  | P47740 | 0.5069 | O35134 | 0.5936 |
|  |  | Q3TDE4 | 0.5068 | Q8BTY3 | 0.5935 |
|  |  | Q91WQ3 | 0.5067 | Q61753 | 0.5935 |
|  |  | Q3TDQ1 | 0.5066 | A0A0B4J1F2 | 0.5935 |
|  |  | Q8VC94 | 0.5065 | I3PQW3 | 0.5934 |
|  |  | F6XC25 | 0.5064 | B2RY56 | 0.5934 |
|  |  | G3UXZ5 | 0.5064 | Q8CFZ0 | 0.5933 |
|  |  | Z4YL78 | 0.5062 | Q99N87 | 0.5933 |
|  |  | Q3TF41 | 0.5061 | Q99J09 | 0.5932 |
|  |  | Q00PI9 | 0.5056 | Q8K411 | 0.5932 |
|  |  | Q6PFD9 | 0.5056 | P27612 | 0.5931 |
|  |  | Q6ZQI3 | 0.5053 | Q921H9 | 0.5931 |
|  |  | Q8BKS9 | 0.5053 | Q9CRY7 | 0.5930 |
|  |  | Q3UJK2 | 0.5052 | E9PWB1 | 0.5930 |
|  |  | Q9CQU0 | 0.5050 | Q99PV0 | 0.5930 |
|  |  | P80315 | 0.5048 | Q8R086 | 0.5930 |
|  |  | P62259 | 0.5048 | P47963 | 0.5929 |
|  |  | O88492 | 0.5047 | A2A5R2 | 0.5929 |
|  |  | Q8CG48 | 0.5046 | P62880 | 0.5928 |
|  |  | Q9D2R0 | 0.5042 | P36552 | 0.5928 |
|  |  | Q9D0R2 | 0.5039 | P80318 | 0.5928 |
|  |  | A6PWC3 | 0.5038 | Q3UGN9 | 0.5927 |
|  |  | F8VQC1 | 0.5036 | P97742 | 0.5926 |
|  |  | E9PYI8 | 0.5032 | P51863 | 0.5926 |
|  |  | Q3UK68 | 0.5031 | Q8BGC4 | 0.5926 |
|  |  | Q3UIS6 | 0.5029 | Q91VE6 | 0.5926 |
|  |  | Q91V76 | 0.5025 | P46664 | 0.5926 |
|  |  | Q9CRY7 | 0.5022 | Q9ER88 | 0.5926 |
|  |  | P58252 | 0.5014 | A7VL18 | 0.5925 |
|  |  | Q9D172 | 0.5014 | Q3TXJ4 | 0.5924 |
|  |  | A3KMJ8 | 0.5010 | Q8VDM6 | 0.5924 |
|  |  | Q5XJY5 | 0.5002 | P60335 | 0.5923 |
|  |  | P47738 | 0.5000 | Q3TJ52 | 0.5922 |
|  |  | Q8BK67 | 0.4995 | Q9R1P1 | 0.5921 |
|  |  | Q03145 | 0.4994 | Q8R436 | 0.5921 |
|  |  | Q3THQ5 | 0.4993 | Q05D44 | 0.5921 |
|  |  | Q99LP6 | 0.4991 | P97452 | 0.5920 |
|  |  | I7HJS1 | 0.4990 | Q3V2N5 | 0.5920 |
|  |  | Q3THZ8 | 0.4990 | Q91WC0 | 0.5919 |
|  |  | Q6ZWS7 | 0.4990 | Q8CG48 | 0.5918 |
|  |  | Q6DFW4 | 0.4984 | Q6P9Q4 | 0.5917 |
|  |  | Q99K51 | 0.4984 | Q8K0C9 | 0.5916 |
|  |  | O35326 | 0.4982 | Q8CE96 | 0.5913 |
|  |  | Q3U2B5 | 0.4980 | D3YWF6 | 0.5913 |
|  |  | Q3U5R8 | 0.4980 | Q8R010 | 0.5912 |
|  |  | E9PYT3 | 0.4977 | Q9JHU4 | 0.5912 |
|  |  | P47199 | 0.4975 | P17427 | 0.5911 |
|  |  | P29341 | 0.4973 | P99026 | 0.5911 |
|  |  | P16045 | 0.4969 | P35585 | 0.5911 |
|  |  | P05201 | 0.4968 | Q3TDU5 | 0.5910 |
|  |  | P32233 | 0.4965 | Q9DC23 | 0.5909 |
|  |  | Q9CWT6 | 0.4965 | Q8C0D5 | 0.5909 |
|  |  | Q9Z1Q5 | 0.4964 | P40142 | 0.5908 |
|  |  | P21619 | 0.4962 | Q6NXX6 | 0.5907 |
|  |  | Z4YKM2 | 0.4960 | P18760 | 0.5907 |
|  |  | Q3UAZ7 | 0.4956 | Q3TQI7 | 0.5907 |
|  |  | Q8C845 | 0.4956 | Q03958 | 0.5906 |
|  |  | Q3TEN9 | 0.4954 | Q3TH64 | 0.5906 |
|  |  | P62855 | 0.4948 | E9Q4Q2 | 0.5906 |
|  |  | Z4YKV1 | 0.4947 | O35129 | 0.5905 |
|  |  | Q9CVT6 | 0.4947 | Q9QXB9 | 0.5905 |
|  |  | P62748 | 0.4946 | P62204 | 0.5905 |
|  |  | P62900 | 0.4944 | Q3UUX9 | 0.5904 |
|  |  | Q3UDR2 | 0.4942 | Q3TVM1 | 0.5904 |
|  |  | P31786 | 0.4941 | Q6ZQ38 | 0.5904 |
|  |  | Q3UBB0 | 0.4940 | E9QL31 | 0.5903 |
|  |  | P09671 | 0.4937 | Q8R038 | 0.5903 |
|  |  | Q9CZU6 | 0.4936 | Q9CR16 | 0.5902 |
|  |  | Q8WTY4 | 0.4934 | Q9CPT4 | 0.5902 |
|  |  | Q3UGJ7 | 0.4934 | Q8CIF2 | 0.5901 |
|  |  | Q99M31 | 0.4924 | P24547 | 0.5900 |
|  |  | Q3U6U7 | 0.4924 | Q64727 | 0.5899 |
|  |  | A2BDX2 | 0.4923 | Q8BH58 | 0.5899 |
|  |  | Q7TMH5 | 0.4923 | P61965 | 0.5899 |
|  |  | Q3UJW9 | 0.4920 | Q8BHG9 | 0.5898 |
|  |  | Q9R1P3 | 0.4920 | A2BE93 | 0.5898 |
|  |  | Q8JZX4 | 0.4918 | Q8K2Z4 | 0.5898 |
|  |  | Q6P5F9 | 0.4912 | Q9Z1Z2 | 0.5898 |
|  |  | A2A9X5 | 0.4909 | Q9D0R2 | 0.5897 |
|  |  | Q3UAG2 | 0.4909 | B2RUJ7 | 0.5897 |
|  |  | F6RJV6 | 0.4908 | P47857 | 0.5896 |
|  |  | Q3THU8 | 0.4898 | Q9DCM0 | 0.5896 |
|  |  | P68510 | 0.4897 | Q61768 | 0.5894 |
|  |  | Q99ME9 | 0.4897 | Q8VBZ3 | 0.5894 |
|  |  | Q8BPB0 | 0.4895 | A0A068F126 | 0.5894 |
|  |  | Q922Q8 | 0.4895 | Q9DCT8 | 0.5894 |
|  |  | Q9QZD9 | 0.4893 | Q62383 | 0.5893 |
|  |  | Q9D0R8 | 0.4893 | Q91W50 | 0.5893 |
|  |  | Q8VCN5 | 0.4889 | P62311 | 0.5890 |
|  |  | P53811 | 0.4889 | Q9D1H7 | 0.5890 |
|  |  | G3X956 | 0.4888 | Q3TUI9 | 0.5889 |
|  |  | Q91VR5 | 0.4886 | P16254 | 0.5889 |
|  |  | Q6PHZ1 | 0.4883 | P23492 | 0.5888 |
|  |  | P14211 | 0.4882 | P57759 | 0.5888 |
|  |  | Q9D7V6 | 0.4878 | P25206 | 0.5888 |
|  |  | Q9D6J6 | 0.4877 | Q99M31 | 0.5888 |
|  |  | P99029 | 0.4875 | H3BIX4 | 0.5887 |
|  |  | P27773 | 0.4874 | Q8BMJ2 | 0.5887 |
|  |  | P68254 | 0.4871 | A6PWC3 | 0.5887 |
|  |  | Q8JZN5 | 0.4871 | E9Q855 | 0.5887 |
|  |  | Q8BUM1 | 0.4870 | P27601 | 0.5886 |
|  |  | Q9JLV5 | 0.4869 | P50396 | 0.5886 |
|  |  | E9PUE7 | 0.4869 | P35979 | 0.5886 |
|  |  | A0AUN0 | 0.4869 | P51150 | 0.5885 |
|  |  | O55135 | 0.4866 | E9Q6Q8 | 0.5885 |
|  |  | P50543 | 0.4861 | B9EHJ3 | 0.5885 |
|  |  | P62830 | 0.4858 | P51410 | 0.5884 |
|  |  | P45377 | 0.4857 | Q64442 | 0.5883 |
|  |  | Q7TT42 | 0.4856 | Q9D8N0 | 0.5883 |
|  |  | P15864 | 0.4852 | Q99J77 | 0.5883 |
|  |  | Q3UQD0 | 0.4852 | Q6A0A2 | 0.5882 |
|  |  | P59325 | 0.4851 | Q5U5W6 | 0.5882 |
|  |  | Q8VCE7 | 0.4848 | Q4V9X9 | 0.5882 |
|  |  | Q9DBH5 | 0.4848 | Q7TT04 | 0.5882 |
|  |  | P14733 | 0.4846 | B9EKP5 | 0.5882 |
|  |  | Q99KV1 | 0.4841 | P52196 | 0.5881 |
|  |  | Q6IRU2 | 0.4837 | Q64010 | 0.5881 |
|  |  | Q9QWZ1 | 0.4835 | Q14CH7 | 0.5881 |
|  |  | P62869 | 0.4833 | P21279 | 0.5881 |
|  |  | P52503 | 0.4833 | P61620 | 0.5880 |
|  |  | Q3U9H4 | 0.4832 | Q6P7V9 | 0.5880 |
|  |  | P16332 | 0.4832 | Q8BJ71 | 0.5879 |
|  |  | Q99JW7 | 0.4828 | E9QKZ2 | 0.5879 |
|  |  | O35655 | 0.4821 | Q8C1M0 | 0.5878 |
|  |  | Q62165 | 0.4820 | Q9CPY7 | 0.5878 |
|  |  | B7ZP20 | 0.4811 | D3YYC2 | 0.5878 |
|  |  | Q9D2Y4 | 0.4805 | Q8BMF4 | 0.5878 |
|  |  | P02469 | 0.4805 | B2RXU2 | 0.5878 |
|  |  | P46460 | 0.4805 | Q8CI86 | 0.5877 |
|  |  | A0A068BFR3 | 0.4804 | Q925I1 | 0.5877 |
|  |  | E0CY49 | 0.4802 | Q3U026 | 0.5876 |
|  |  | Q3TCW5 | 0.4800 | O35887 | 0.5876 |
|  |  | Q3U5I9 | 0.4799 | P35831 | 0.5875 |
|  |  | Q8BU20 | 0.4796 | H3BK68 | 0.5875 |
|  |  | Q32P00 | 0.4792 | Q3UXU8 | 0.5875 |
|  |  | Q04207 | 0.4792 | Q8VCF0 | 0.5874 |
|  |  | F6RDM4 | 0.4791 | P57780 | 0.5874 |
|  |  | O35381 | 0.4785 | Q9ERA0 | 0.5874 |
|  |  | Q501J6 | 0.4784 | A0AUN0 | 0.5873 |
|  |  | Q9CWU9 | 0.4782 | Q9CZ44 | 0.5873 |
|  |  | Q61081 | 0.4781 | Q99JB2 | 0.5872 |
|  |  | Q9D6K8 | 0.4780 | Q76MZ3 | 0.5872 |
|  |  | Q9ERR7 | 0.4778 | Q0P6B2 | 0.5871 |
|  |  | O55057 | 0.4777 | Q9DCR2 | 0.5870 |
|  |  | P61290 | 0.4771 | Q3TWV4 | 0.5869 |
|  |  | Q9QYR9 | 0.4769 | Q8R1B4 | 0.5869 |
|  |  | Q8R5H1 | 0.4767 | Q60737 | 0.5869 |
|  |  | H3BKK2 | 0.4767 | E9Q586 | 0.5868 |
|  |  | A2ATT5 | 0.4767 | Q9D892 | 0.5868 |
|  |  | P62204 | 0.4766 | Q3U2B5 | 0.5867 |
|  |  | D3Z2F7 | 0.4763 | Q3UW66 | 0.5867 |
|  |  | Q9CZD3 | 0.4762 | Q99KJ8 | 0.5867 |
|  |  | Q3TL58 | 0.4762 | G5E8R3 | 0.5867 |
|  |  | Q9JI10 | 0.4761 | Q9CQE8 | 0.5867 |
|  |  | P47962 | 0.4754 | P39053 | 0.5866 |
|  |  | Q3TN31 | 0.4754 | P45376 | 0.5866 |
|  |  | Q91V55 | 0.4754 | P10126 | 0.5866 |
|  |  | Q9ERE7 | 0.4753 | Q9ERN0 | 0.5866 |
|  |  | Q99J77 | 0.4753 | Q8C622 | 0.5865 |
|  |  | Q9CQZ5 | 0.4752 | Q8K297 | 0.5865 |
|  |  | P14131 | 0.4750 | F6VQ81 | 0.5865 |
|  |  | Q6PAR5 | 0.4744 | Q9JJ78 | 0.5864 |
|  |  | P63325 | 0.4743 | Q3TKT4 | 0.5863 |
|  |  | Q99PL5 | 0.4743 | P54276 | 0.5863 |
|  |  | Q9JJI8 | 0.4742 | Q8WTY4 | 0.5863 |
|  |  | Q3U561 | 0.4742 | Q9DBR1 | 0.5862 |
|  |  | Q9D0J2 | 0.4741 | Q8BN64 | 0.5862 |
|  |  | Q99KD6 | 0.4741 | G3UWZ0 | 0.5862 |
|  |  | Q8BTY3 | 0.4740 | O88811 | 0.5862 |
|  |  | Q9D554 | 0.4739 | P63325 | 0.5862 |
|  |  | A2BFF8 | 0.4736 | Q8BK29 | 0.5861 |
|  |  | Q3TVM1 | 0.4734 | P84096 | 0.5861 |
|  |  | Q8CI86 | 0.4734 | P08249 | 0.5860 |
|  |  | O70251 | 0.4732 | Q61749 | 0.5860 |
|  |  | P51881 | 0.4730 | Q8BH80 | 0.5859 |
|  |  | Q8BIP0 | 0.4724 | Q61553 | 0.5857 |
|  |  | G3X9U9 | 0.4723 | Q9DC50 | 0.5856 |
|  |  | Q3TK27 | 0.4717 | Q99K70 | 0.5856 |
|  |  | P62911 | 0.4715 | Q9DBG3 | 0.5856 |
|  |  | Q5M9N6 | 0.4712 | Q8CC03 | 0.5856 |
|  |  | E9Q616 | 0.4711 | Q8BKT8 | 0.5855 |
|  |  | Q3UGS4 | 0.4711 | Q7TPT7 | 0.5855 |
|  |  | P15626 | 0.4711 | E9QAS4 | 0.5855 |
|  |  | P62267 | 0.4709 | Q9CQD1 | 0.5854 |
|  |  | O70435 | 0.4701 | Q922Q8 | 0.5854 |
|  |  | Q99KI0 | 0.4700 | G3UVU2 | 0.5854 |
|  |  | P63028 | 0.4698 | P70444 | 0.5854 |
|  |  | G3UYD0 | 0.4697 | P58021 | 0.5854 |
|  |  | A2BE93 | 0.4689 | Q3TIX9 | 0.5853 |
|  |  | G3UZY2 | 0.4687 | Q3U643 | 0.5853 |
|  |  | P63005 | 0.4687 | Q6P5E4 | 0.5852 |
|  |  | O88811 | 0.4686 | Q9Z1T1 | 0.5852 |
|  |  | Q99JX4 | 0.4685 | Q91YZ2 | 0.5852 |
|  |  | A2AU61 | 0.4682 | P62827 | 0.5852 |
|  |  | Q9R1P0 | 0.4681 | P45377 | 0.5851 |
|  |  | Q8C2Q8 | 0.4676 | B1AU25 | 0.5850 |
|  |  | Q9D1P4 | 0.4675 | Q3UD67 | 0.5849 |
|  |  | Q3UF75 | 0.4674 | Q3V1M8 | 0.5849 |
|  |  | Q1WWK3 | 0.4670 | P46460 | 0.5848 |
|  |  | Q811N1 | 0.4667 | P16460 | 0.5846 |
|  |  | O35737 | 0.4661 | Q9Z0H8 | 0.5846 |
|  |  | S4R294 | 0.4660 | Q3THK3 | 0.5845 |
|  |  | Q4VA53 | 0.4658 | Q9DCW4 | 0.5845 |
|  |  | Q64727 | 0.4655 | Q91YS7 | 0.5844 |
|  |  | Q3TD51 | 0.4649 | Q99K48 | 0.5844 |
|  |  | Q8C1Y3 | 0.4644 | Q3THQ5 | 0.5844 |
|  |  | P99024 | 0.4644 | Q9D0L7 | 0.5844 |
|  |  | Q3U7I9 | 0.4643 | P00375 | 0.5844 |
|  |  | Q9D1G1 | 0.4642 | P54071 | 0.5842 |
|  |  | Q9Z2Y8 | 0.4642 | Q9CT23 | 0.5841 |
|  |  | Q8BH95 | 0.4639 | O35655 | 0.5841 |
|  |  | Q922D4 | 0.4638 | O88398 | 0.5840 |
|  |  | P54071 | 0.4636 | Q3UGS9 | 0.5840 |
|  |  | Q06138 | 0.4631 | Q9QZ08 | 0.5840 |
|  |  | Q8R3R9 | 0.4626 | P19783 | 0.5839 |
|  |  | Q60737 | 0.4625 | Q91XU3 | 0.5837 |
|  |  | Q7TMK9 | 0.4622 | A0A087WNV1 | 0.5834 |
|  |  | Q9DBL7 | 0.4621 | P59999 | 0.5833 |
|  |  | A2AKI5 | 0.4618 | R4H4V1 | 0.5832 |
|  |  | Q8BK72 | 0.4616 | Q9JLI6 | 0.5831 |
|  |  | O08749 | 0.4608 | Q61166 | 0.5831 |
|  |  | K7Q751 | 0.4607 | Q9D1M7 | 0.5831 |
|  |  | Q3UAX2 | 0.4607 | Q8BV40 | 0.5830 |
|  |  | Q9D020 | 0.4594 | E9Q310 | 0.5830 |
|  |  | Q6P069 | 0.4574 | Q99JI6 | 0.5828 |
|  |  | P51410 | 0.4573 | Q5MJ56 | 0.5827 |
|  |  | P56812 | 0.4571 | Q8BXZ1 | 0.5827 |
|  |  | P59708 | 0.4566 | Q8CIG8 | 0.5827 |
|  |  | Q99020 | 0.4566 | Q91WN1 | 0.5825 |
|  |  | Q99KF1 | 0.4552 | Q3UYV9 | 0.5825 |
|  |  | Q06185 | 0.4551 | Q8BTE1 | 0.5825 |
|  |  | Q4FE56 | 0.4544 | G3UXW9 | 0.5824 |
|  |  | O09131 | 0.4542 | Q3TG12 | 0.5824 |
|  |  | Q3U8R9 | 0.4539 | Q7TMK9 | 0.5823 |
|  |  | P43275 | 0.4534 | Q4FJZ2 | 0.5823 |
|  |  | Q05BH6 | 0.4531 | Q9JLV5 | 0.5822 |
|  |  | Q9WV55 | 0.4524 | P31786 | 0.5822 |
|  |  | P62320 | 0.4523 | Q9DCA4 | 0.5821 |
|  |  | P61089 | 0.4521 | Q1KYM0 | 0.5821 |
|  |  | A2ATP5 | 0.4517 | Q7TMY8 | 0.5820 |
|  |  | P50518 | 0.4510 | Q8BTS4 | 0.5819 |
|  |  | P18155 | 0.4507 | P61027 | 0.5819 |
|  |  | Q8BG51 | 0.4506 | Q99KG1 | 0.5819 |
|  |  | Q91VJ4 | 0.4501 | O54734 | 0.5818 |
|  |  | G3UZ26 | 0.4499 | Q8BKS9 | 0.5817 |
|  |  | Q9WUL7 | 0.4499 | G3UYV7 | 0.5817 |
|  |  | Q3TI63 | 0.4498 | P35486 | 0.5817 |
|  |  | Q6P5E4 | 0.4495 | Q8BK67 | 0.5816 |
|  |  | Q5EBG5 | 0.4494 | Q3TA75 | 0.5815 |
|  |  | Q9DAU1 | 0.4494 | Q3TFP0 | 0.5815 |
|  |  | P35564 | 0.4494 | B1ARA5 | 0.5814 |
|  |  | Q9CQN7 | 0.4492 | P19253 | 0.5814 |
|  |  | Q9DBP5 | 0.4489 | B2M1R7 | 0.5813 |
|  |  | Q76MZ3 | 0.4488 | Z4YLI8 | 0.5813 |
|  |  | Q62446 | 0.4488 | F6SVV1 | 0.5812 |
|  |  | Q8CGZ0 | 0.4483 | P62242 | 0.5811 |
|  |  | Q9D828 | 0.4482 | O88447 | 0.5811 |
|  |  | P63101 | 0.4482 | Q8C2E7 | 0.5810 |
|  |  | P46935 | 0.4476 | A2BGI8 | 0.5810 |
|  |  | P97825 | 0.4473 | Q99JX6 | 0.5810 |
|  |  | Q6P7V9 | 0.4473 | D3YVN7 | 0.5808 |
|  |  | P62889 | 0.4470 | P47754 | 0.5808 |
|  |  | B7ZWC4 | 0.4466 | Q8K298 | 0.5808 |
|  |  | A2ALV7 | 0.4465 | G3UXL2 | 0.5807 |
|  |  | Q8CH18 | 0.4457 | Q9DBC3 | 0.5806 |
|  |  | O08709 | 0.4450 | P62869 | 0.5806 |
|  |  | O08599 | 0.4447 | P11499 | 0.5806 |
|  |  | P26350 | 0.4440 | Q9CPN8 | 0.5805 |
|  |  | F8WH20 | 0.4439 | Q05BN2 | 0.5804 |
|  |  | O70252 | 0.4439 | Q8BHC4 | 0.5804 |
|  |  | P26443 | 0.4433 | G3X956 | 0.5804 |
|  |  | Q9CQB4 | 0.4433 | Q9JJ28 | 0.5803 |
|  |  | Q05186 | 0.4431 | Q3UL78 | 0.5802 |
|  |  | Q9JIX0 | 0.4425 | Q6P6I4 | 0.5801 |
|  |  | O88874 | 0.4423 | Q9R1T2 | 0.5801 |
|  |  | P97770 | 0.4417 | Q5M9L1 | 0.5801 |
|  |  | Q9WV98 | 0.4417 | Q3USP3 | 0.5801 |
|  |  | Q6P8X1 | 0.4411 | Q9WVA4 | 0.5800 |
|  |  | G3X9T8 | 0.4411 | Q9D2R8 | 0.5799 |
|  |  | Q91WN1 | 0.4408 | B2RWW1 | 0.5799 |
|  |  | D3Z4V1 | 0.4391 | Q8BMS1 | 0.5797 |
|  |  | Q99LC3 | 0.4391 | A0ZVB6 | 0.5796 |
|  |  | Q99KG1 | 0.4386 | Q9D706 | 0.5796 |
|  |  | Q62425 | 0.4378 | Q62087 | 0.5796 |
|  |  | P28658 | 0.4375 | O35737 | 0.5795 |
|  |  | Q6PGB6 | 0.4374 | E0CYI7 | 0.5795 |
|  |  | Q3TEL0 | 0.4373 | Q9D8X2 | 0.5794 |
|  |  | P19536 | 0.4359 | Q497Z1 | 0.5793 |
|  |  | Q61768 | 0.4352 | Q6NVF9 | 0.5793 |
|  |  | Q99L45 | 0.4350 | Q3TJ21 | 0.5792 |
|  |  | Q80XR5 | 0.4350 | P47941 | 0.5790 |
|  |  | Q3TB79 | 0.4331 | Q921S7 | 0.5789 |
|  |  | Q9WUU7 | 0.4326 | O08583 | 0.5788 |
|  |  | Q3TL79 | 0.4324 | P62281 | 0.5786 |
|  |  | A7VL18 | 0.4316 | Q9D1G1 | 0.5786 |
|  |  | Q91VM5 | 0.4316 | Q9CWT6 | 0.5786 |
|  |  | Q8VIJ6 | 0.4315 | Q3U292 | 0.5784 |
|  |  | Q9CQE8 | 0.4304 | B7ZNJ0 | 0.5784 |
|  |  | Q3U7A6 | 0.4302 | Q3THA0 | 0.5784 |
|  |  | P16125 | 0.4299 | Q9JIF7 | 0.5783 |
|  |  | O54692 | 0.4298 | Q9DB15 | 0.5783 |
|  |  | Q99P31 | 0.4295 | Q4FZL1 | 0.5783 |
|  |  | Q62159 | 0.4294 | Q3ULP8 | 0.5782 |
|  |  | A2AP78 | 0.4292 | Q3TSX8 | 0.5782 |
|  |  | Q8C140 | 0.4287 | Q3TDD8 | 0.5781 |
|  |  | Q60973 | 0.4287 | E9PWY9 | 0.5780 |
|  |  | Q9Z2G9 | 0.4286 | Q3U781 | 0.5780 |
|  |  | Q80X50 | 0.4286 | D3Z619 | 0.5780 |
|  |  | B2RXU2 | 0.4286 | G3UX26 | 0.5779 |
|  |  | Q99KR7 | 0.4282 | Q3UHX2 | 0.5779 |
|  |  | Q5DU34 | 0.4278 | Q3UJ44 | 0.5779 |
|  |  | E0CX20 | 0.4275 | P49710 | 0.5778 |
|  |  | P14685 | 0.4272 | Q3TJ01 | 0.5777 |
|  |  | Q9D5V5 | 0.4258 | A3KMJ8 | 0.5777 |
|  |  | B1ARA5 | 0.4257 | Q9Z0P5 | 0.5777 |
|  |  | A2RRJ4 | 0.4254 | A0A0A6YW80 | 0.5775 |
|  |  | Q3TE45 | 0.4253 | A0A075DC90 | 0.5775 |
|  |  | Q3TX72 | 0.4247 | Q91VM9 | 0.5775 |
|  |  | Q9DB15 | 0.4247 | Q5QNU0 | 0.5774 |
|  |  | Q9CR59 | 0.4246 | P35279 | 0.5774 |
|  |  | P62331 | 0.4238 | Q6NWW1 | 0.5773 |
|  |  | Q8R1V4 | 0.4234 | A2AAN2 | 0.5772 |
|  |  | P58044 | 0.4230 | Q9R1C7 | 0.5771 |
|  |  | I7HLV2 | 0.4226 | Q3ULT8 | 0.5770 |
|  |  | Q60865 | 0.4224 | Q91VA7 | 0.5770 |
|  |  | B1ATZ0 | 0.4224 | Q8BKZ9 | 0.5769 |
|  |  | P04104 | 0.4222 | P10711 | 0.5768 |
|  |  | Q61074 | 0.4214 | Q3TB79 | 0.5767 |
|  |  | Q9CYH6 | 0.4213 | P20108 | 0.5767 |
|  |  | Q3ULI5 | 0.4209 | A0A0A6YWP9 | 0.5767 |
|  |  | Q4V9X9 | 0.4205 | Q99L13 | 0.5767 |
|  |  | Q8CFQ9 | 0.4203 | F8WHU9 | 0.5765 |
|  |  | A2ALF0 | 0.4200 | Q8C1X9 | 0.5764 |
|  |  | Q6P5B0 | 0.4195 | P97370 | 0.5764 |
|  |  | E9PXV3 | 0.4189 | Q3TVD9 | 0.5763 |
|  |  | Q3UMR5 | 0.4185 | Q91YR7 | 0.5762 |
|  |  | Q9CWW6 | 0.4179 | Q03265 | 0.5762 |
|  |  | A3KGQ6 | 0.4177 | Q6ZWV7 | 0.5761 |
|  |  | O88653 | 0.4175 | Q8JZQ9 | 0.5761 |
|  |  | P14148 | 0.4170 | Q3TKU6 | 0.5760 |
|  |  | Q99L47 | 0.4169 | Q3UK61 | 0.5760 |
|  |  | Q2YDW1 | 0.4169 | Q3TTX0 | 0.5759 |
|  |  | O35864 | 0.4160 | Q3U0J1 | 0.5758 |
|  |  | P17047 | 0.4144 | D3Z5M2 | 0.5757 |
|  |  | E9Q634 | 0.4140 | Q64433 | 0.5756 |
|  |  | Q8R5L1 | 0.4138 | Q9CPQ8 | 0.5756 |
|  |  | Q80W54 | 0.4135 | F6ZDS4 | 0.5755 |
|  |  | D3Z6S1 | 0.4133 | G8JL40 | 0.5754 |
|  |  | Q8R050 | 0.4129 | E9PXY1 | 0.5754 |
|  |  | A1L0U3 | 0.4124 | B7ZWF1 | 0.5753 |
|  |  | O08997 | 0.4124 | O35841 | 0.5752 |
|  |  | Q5SW88 | 0.4116 | Q3TJD4 | 0.5752 |
|  |  | Q6NZM8 | 0.4115 | F6TBV1 | 0.5751 |
|  |  | P09405 | 0.4107 | P28658 | 0.5751 |
|  |  | Q3TMX5 | 0.4100 | Q3UNF6 | 0.5750 |
|  |  | Q3U617 | 0.4091 | Q8VC94 | 0.5749 |
|  |  | B2RXR6 | 0.4090 | Q3TF81 | 0.5749 |
|  |  | P84084 | 0.4083 | Q9D5T0 | 0.5748 |
|  |  | Q99JF8 | 0.4076 | E9QAI5 | 0.5747 |
|  |  | P62827 | 0.4046 | J3QMC5 | 0.5746 |
|  |  | Q6PB66 | 0.4045 | Q8CDZ5 | 0.5745 |
|  |  | Q7TMM9 | 0.4039 | Q8VIJ6 | 0.5745 |
|  |  | Q8BIW1 | 0.4029 | Q9EP69 | 0.5745 |
|  |  | Q9CQU3 | 0.4023 | Q3TDE4 | 0.5744 |
|  |  | E9PYC6 | 0.4018 | O55029 | 0.5744 |
|  |  | P33174 | 0.4002 | Q3UNJ3 | 0.5744 |
|  |  | P63330 | 0.3987 | P47740 | 0.5742 |
|  |  | P19253 | 0.3982 | Q3TIB7 | 0.5742 |
|  |  | Q3UK38 | 0.3977 | Q99LC5 | 0.5741 |
|  |  | Q9CPN9 | 0.3975 | Q6IRT4 | 0.5741 |
|  |  | Q3US29 | 0.3970 | A2ALV7 | 0.5740 |
|  |  | B1AWZ5 | 0.3964 | Q3TBU6 | 0.5740 |
|  |  | P51859 | 0.3959 | Q8VCE7 | 0.5738 |
|  |  | Q9D1M4 | 0.3959 | Q3UT02 | 0.5738 |
|  |  | Q8C0Z3 | 0.3949 | P02469 | 0.5738 |
|  |  | Q9WVE8 | 0.3948 | Q501J6 | 0.5738 |
|  |  | Q9CQS8 | 0.3947 | P49312 | 0.5737 |
|  |  | Q9CRS5 | 0.3947 | Q9D1N9 | 0.5737 |
|  |  | E9PWK1 | 0.3946 | Q6P4S8 | 0.5736 |
|  |  | Q8BGZ7 | 0.3932 | B2RWW6 | 0.5735 |
|  |  | Q8VE47 | 0.3931 | Q8CCV9 | 0.5735 |
|  |  | F6YQT7 | 0.3921 | Q9CZU6 | 0.5735 |
|  |  | Q6NZN9 | 0.3920 | P54775 | 0.5734 |
|  |  | Q9CQF3 | 0.3885 | Q3UYD0 | 0.5733 |
|  |  | Q80VD1 | 0.3882 | D3YTP0 | 0.5732 |
|  |  | P34022 | 0.3881 | G3X9L6 | 0.5732 |
|  |  | Q3U6Y9 | 0.3879 | Q6P4T2 | 0.5732 |
|  |  | Q9QZQ8 | 0.3836 | Q3UDI8 | 0.5729 |
|  |  | G3UYI5 | 0.3836 | Q3THB3 | 0.5729 |
|  |  | O88844 | 0.3832 | Q9JKY0 | 0.5728 |
|  |  | P12265 | 0.3832 | Q8BIF7 | 0.5728 |
|  |  | Q8K0C4 | 0.3817 | Q9CQ80 | 0.5726 |
|  |  | P24472 | 0.3812 | Q9CR57 | 0.5726 |
|  |  | Q78RK2 | 0.3791 | Q8R059 | 0.5725 |
|  |  | Q6ZWV7 | 0.3765 | Q8K4Z3 | 0.5725 |
|  |  | Q9CQI6 | 0.3754 | Q9CWU9 | 0.5725 |
|  |  | Q9CZ04 | 0.3742 | Q3UW32 | 0.5724 |
|  |  | D3YZ09 | 0.3739 | Q3TDN2 | 0.5723 |
|  |  | F8WHV1 | 0.3723 | Q3UGC1 | 0.5723 |
|  |  | Q6NVC2 | 0.3721 | Q3TJN9 | 0.5722 |
|  |  | Q8CH72 | 0.3721 | P61222 | 0.5722 |
|  |  | Q61425 | 0.3717 | Q9QXA5 | 0.5722 |
|  |  | P48962 | 0.3704 | Q91ZW3 | 0.5722 |
|  |  | P10126 | 0.3695 | Q9WUM3 | 0.5722 |
|  |  | P62702 | 0.3688 | Q921T2 | 0.5721 |
|  |  | P47915 | 0.3686 | G3UXZ5 | 0.5721 |
|  |  | P41105 | 0.3685 | Q921L3 | 0.5720 |
|  |  | G3UVU2 | 0.3674 | F6V084 | 0.5719 |
|  |  | P61982 | 0.3662 | A0A023T672 | 0.5719 |
|  |  | P29595 | 0.3656 | Q6P8X1 | 0.5718 |
|  |  | Q9DC16 | 0.3642 | Q9R1Q6 | 0.5717 |
|  |  | P45878 | 0.3640 | Q9WVG6 | 0.5717 |
|  |  | P07091 | 0.3634 | Q8R5L1 | 0.5716 |
|  |  | P35700 | 0.3618 | Q71RI9 | 0.5716 |
|  |  | Q8R5J9 | 0.3618 | P62908 | 0.5714 |
|  |  | A0JNY7 | 0.3612 | E9Q1M6 | 0.5713 |
|  |  | Q91YN9 | 0.3608 | Q3UEB3 | 0.5713 |
|  |  | Q9Z204 | 0.3600 | Q3TU36 | 0.5713 |
|  |  | P16254 | 0.3583 | Q61656 | 0.5712 |
|  |  | G3UWG1 | 0.3572 | Q5SUR0 | 0.5711 |
|  |  | D3Z041 | 0.3555 | Q8K183 | 0.5711 |
|  |  | Q3TLJ5 | 0.3553 | Q80TX4 | 0.5710 |
|  |  | Q8VCB1 | 0.3526 | P62911 | 0.5708 |
|  |  | Q9D8M4 | 0.3525 | Q8BY87 | 0.5707 |
|  |  | Q9CXW3 | 0.3520 | Q91VM5 | 0.5707 |
|  |  | Q3TDR0 | 0.3511 | Q9CQI6 | 0.5706 |
|  |  | Q4FZG9 | 0.3500 | P53811 | 0.5706 |
|  |  | Q8CFZ0 | 0.3496 | G3X9U9 | 0.5706 |
|  |  | Q3THE6 | 0.3447 | Q61655 | 0.5703 |
|  |  | Q6ZWQ7 | 0.3432 | Q9CRF5 | 0.5703 |
|  |  | P62862 | 0.3431 | F7BTZ2 | 0.5703 |
|  |  | O54962 | 0.3428 | Q9CQV8 | 0.5702 |
|  |  | Q3TFA9 | 0.3411 | Q80U87 | 0.5702 |
|  |  | Q8C2T9 | 0.3389 | Q5SYD0 | 0.5702 |
|  |  | Q5D098 | 0.3381 | P50247 | 0.5702 |
|  |  | Q8BMD8 | 0.3374 | Q3U319 | 0.5702 |
|  |  | Q9JLZ3 | 0.3363 | Q6ZWX6 | 0.5701 |
|  |  | Q3U3C2 | 0.3352 | Q3UGL3 | 0.5701 |
|  |  | Q9CVB6 | 0.3351 | F8VQC1 | 0.5699 |
|  |  | Q05DE0 | 0.3329 | Q7TT37 | 0.5699 |
|  |  | B8JJI4 | 0.3328 | Q8VDN2 | 0.5698 |
|  |  | Q91VA7 | 0.3322 | P47738 | 0.5698 |
|  |  | Q61686 | 0.3314 | Q9QZ23 | 0.5697 |
|  |  | Q3TJN6 | 0.3311 | P35278 | 0.5696 |
|  |  | Q9Z2I9 | 0.3309 | P67778 | 0.5694 |
|  |  | P24369 | 0.3307 | Q3TQP7 | 0.5693 |
|  |  | P09528 | 0.3305 | Q3U057 | 0.5693 |
|  |  | Q921H9 | 0.3269 | Q9DAW6 | 0.5692 |
|  |  | P48428 | 0.3259 | P97450 | 0.5691 |
|  |  | Q60749 | 0.3239 | Q62084 | 0.5689 |
|  |  | P45591 | 0.3232 | Q9CR68 | 0.5688 |
|  |  | E9Q4B9 | 0.3219 | A2AA71 | 0.5686 |
|  |  | P61079 | 0.3215 | D3YYP5 | 0.5686 |
|  |  | Q9WUV0 | 0.3208 | Q6PB66 | 0.5686 |
|  |  | Q3UDM8 | 0.3167 | P97393 | 0.5685 |
|  |  | Q9CXR1 | 0.3154 | Q8R035 | 0.5682 |
|  |  | Q3TWW8 | 0.3149 | Z4YKT6 | 0.5681 |
|  |  | P07141 | 0.3128 | Q99KP6 | 0.5680 |
|  |  | Q8BP67 | 0.3120 | Q9CR59 | 0.5680 |
|  |  | Q3U9U5 | 0.3108 | P52431 | 0.5680 |
|  |  | Q8CD09 | 0.3099 | Q6PAR5 | 0.5680 |
|  |  | O88848 | 0.3059 | P62918 | 0.5679 |
|  |  | P54923 | 0.3057 | Q61578 | 0.5679 |
|  |  | Q9CR16 | 0.3046 | O35387 | 0.5679 |
|  |  | Q9D2M8 | 0.3040 | Q9CSU0 | 0.5679 |
|  |  | Q8BMJ3 | 0.3038 | Q3UIH7 | 0.5678 |
|  |  | Q3B7Z2 | 0.3026 | Q9CRT8 | 0.5674 |
|  |  | P41241 | 0.2993 | Q99020 | 0.5674 |
|  |  | Q3THB4 | 0.2980 | Q8BML9 | 0.5673 |
|  |  | Q9CQC6 | 0.2979 | Q8CAC4 | 0.5673 |
|  |  | H3BLI9 | 0.2960 | A2AMY5 | 0.5672 |
|  |  | P61750 | 0.2936 | E9Q035 | 0.5672 |
|  |  | Q9D1H8 | 0.2932 | Q9Z2I0 | 0.5671 |
|  |  | Q99MV1 | 0.2926 | A2A513 | 0.5670 |
|  |  | Q9D6N1 | 0.2910 | Q64105 | 0.5669 |
|  |  | F8WHU7 | 0.2900 | P62331 | 0.5669 |
|  |  | P62245 | 0.2899 | Q6ZWQ9 | 0.5669 |
|  |  | Q8BKT8 | 0.2896 | F6XC25 | 0.5668 |
|  |  | A2AQ17 | 0.2877 | Q8BGB7 | 0.5668 |
|  |  | H3BKE1 | 0.2865 | Q3ULG5 | 0.5667 |
|  |  | P20060 | 0.2843 | Q6PHZ1 | 0.5666 |
|  |  | Q3TEA8 | 0.2839 | Q9D6K5 | 0.5666 |
|  |  | P08207 | 0.2828 | Q9WUL7 | 0.5665 |
|  |  | Q3THA6 | 0.2812 | Q9Z0W3 | 0.5665 |
|  |  | P27661 | 0.2801 | A1A4T2 | 0.5665 |
|  |  | Q9CPT4 | 0.2779 | Q9EPK7 | 0.5663 |
|  |  | Q9QYJ3 | 0.2755 | Q9JKB1 | 0.5662 |
|  |  | B7ZCU2 | 0.2751 | Q5JC28 | 0.5662 |
|  |  | Q3U781 | 0.2751 | Q91WU5 | 0.5661 |
|  |  | P10639 | 0.2742 | Q3UA06 | 0.5660 |
|  |  | F6SVV1 | 0.2676 | Q9Z0F7 | 0.5660 |
|  |  | Q8CGC6 | 0.2675 | O70194 | 0.5660 |
|  |  | Q80UU6 | 0.2673 | Q6PHN9 | 0.5659 |
|  |  | P41731 | 0.2662 | Q04857 | 0.5659 |
|  |  | D3YXP6 | 0.2640 | P63037 | 0.5658 |
|  |  | P62281 | 0.2622 | P12265 | 0.5658 |
|  |  | Q3TJD4 | 0.2583 | O89001 | 0.5657 |
|  |  | D3Z0F3 | 0.2576 | P37040 | 0.5654 |
|  |  | Q8BKE6 | 0.2570 | Q9CQ54 | 0.5654 |
|  |  | P55264 | 0.2569 | P51881 | 0.5653 |
|  |  | P97315 | 0.2565 | Q9JMH6 | 0.5653 |
|  |  | D3YVV7 | 0.2530 | P62900 | 0.5652 |
|  |  | F6SFF5 | 0.2530 | P56812 | 0.5651 |
|  |  | Q569Z6 | 0.2492 | Q8K0L1 | 0.5651 |
|  |  | P50637 | 0.2491 | Q8BP47 | 0.5649 |
|  |  | Q3TSX5 | 0.2463 | Q8CD92 | 0.5648 |
|  |  | Q9CZX8 | 0.2442 | B9EJ77 | 0.5648 |
|  |  | Q3U449 | 0.2436 | Q3TCW5 | 0.5648 |
|  |  | E9QKL6 | 0.2434 | Q8C166 | 0.5647 |
|  |  | P62774 | 0.2428 | Q9JL35 | 0.5647 |
|  |  | A0A087WQD1 | 0.2420 | P51660 | 0.5647 |
|  |  | Q91ZW3 | 0.2419 | Q99KI0 | 0.5647 |
|  |  | P17665 | 0.2417 | Q0QEW9 | 0.5647 |
|  |  | G5E8G0 | 0.2410 | P58044 | 0.5646 |
|  |  | P61082 | 0.2379 | Q9DB05 | 0.5645 |
|  |  | Q9CPQ1 | 0.2367 | G5E896 | 0.5644 |
|  |  | Q9D6J9 | 0.2327 | E9Q9E1 | 0.5643 |
|  |  | Q9D1R9 | 0.2314 | Q8C1Y3 | 0.5642 |
|  |  | P62852 | 0.2281 | Q8BL36 | 0.5642 |
|  |  | Q3UJR8 | 0.2276 | Q8JZN5 | 0.5641 |
|  |  | Q922Q1 | 0.2273 | Q684I8 | 0.5640 |
|  |  | Q91VG6 | 0.2258 | Q3V493 | 0.5640 |
|  |  | B2RTP7 | 0.2247 | Q8CGC6 | 0.5638 |
|  |  | D3Z5M2 | 0.2247 | A1A596 | 0.5637 |
|  |  | Q921E4 | 0.2233 | Q3UJP8 | 0.5636 |
|  |  | Q3TSX8 | 0.2231 | Q8BGS0 | 0.5636 |
|  |  | Q93092 | 0.2224 | Q4VA53 | 0.5634 |
|  |  | Q3UM18 | 0.2210 | P14115 | 0.5634 |
|  |  | Q3TJG6 | 0.2154 | E9Q852 | 0.5634 |
|  |  | Q9DCJ5 | 0.2151 | Q8K265 | 0.5634 |
|  |  | Q3TL33 | 0.2113 | Q9DD06 | 0.5634 |
|  |  | Q6ZWN5 | 0.2086 | Q68FC6 | 0.5634 |
|  |  | A2AUE1 | 0.2028 | G3UWX1 | 0.5633 |
|  |  | Q3TC83 | 0.2007 | A2RRJ4 | 0.5633 |
|  |  | Q3TVV6 | 0.1971 | P42208 | 0.5632 |
|  |  | E9PUH0 | 0.1933 | P63330 | 0.5632 |
|  |  | Q3TZK4 | 0.1931 | A0A0A0MQ80 | 0.5630 |
|  |  | Q6PJ91 | 0.1912 | B1AT10 | 0.5630 |
|  |  | Q6PHN9 | 0.1909 | Q6PIP5 | 0.5629 |
|  |  | Q3UYV7 | 0.1906 | A0A0A6YWG8 | 0.5628 |
|  |  | Q66JR8 | 0.1905 | D3YZ09 | 0.5628 |
|  |  | P32020 | 0.1893 | Q08509 | 0.5628 |
|  |  | O55142 | 0.1831 | Q8CEG3 | 0.5628 |
|  |  | Q9JL35 | 0.1776 | Q3THU8 | 0.5627 |
|  |  | P19001 | 0.1762 | Q9CR61 | 0.5626 |
|  |  | A0A087WRY3 | 0.1715 | P15864 | 0.5626 |
|  |  | G8DXR6 | 0.1599 | Q91ZX7 | 0.5626 |
|  |  |  |  | D3Z7P2 | 0.5625 |
|  |  |  |  | H7BWX9 | 0.5624 |
|  |  |  |  | Q921N6 | 0.5623 |
|  |  |  |  | A0A087WS18 | 0.5622 |
|  |  |  |  | Q8CJ53 | 0.5621 |
|  |  |  |  | Q6PFD9 | 0.5621 |
|  |  |  |  | Q45VK5 | 0.5621 |
|  |  |  |  | Q9WUV0 | 0.5620 |
|  |  |  |  | Q80VJ3 | 0.5619 |
|  |  |  |  | Q9CQV4 | 0.5619 |
|  |  |  |  | Q61081 | 0.5618 |
|  |  |  |  | Q3TKY6 | 0.5616 |
|  |  |  |  | O35381 | 0.5616 |
|  |  |  |  | Q8R326 | 0.5616 |
|  |  |  |  | P97352 | 0.5616 |
|  |  |  |  | Q3THK7 | 0.5616 |
|  |  |  |  | P62717 | 0.5615 |
|  |  |  |  | Q99N15 | 0.5615 |
|  |  |  |  | Q8CD09 | 0.5615 |
|  |  |  |  | O70310 | 0.5615 |
|  |  |  |  | Q3U878 | 0.5614 |
|  |  |  |  | Q3TWW8 | 0.5614 |
|  |  |  |  | D3Z0F5 | 0.5614 |
|  |  |  |  | A3KGQ6 | 0.5613 |
|  |  |  |  | Q9Z2X1 | 0.5613 |
|  |  |  |  | Q9D338 | 0.5613 |
|  |  |  |  | Q8BH59 | 0.5613 |
|  |  |  |  | Q3V0L4 | 0.5613 |
|  |  |  |  | Q8R395 | 0.5612 |
|  |  |  |  | Q60902 | 0.5611 |
|  |  |  |  | O09005 | 0.5611 |
|  |  |  |  | Q8BTV2 | 0.5610 |
|  |  |  |  | Q8BX02 | 0.5610 |
|  |  |  |  | Q6PDG0 | 0.5610 |
|  |  |  |  | P80315 | 0.5609 |
|  |  |  |  | Q9CSS1 | 0.5608 |
|  |  |  |  | E9PYI8 | 0.5607 |
|  |  |  |  | P51174 | 0.5607 |
|  |  |  |  | Q9ERR7 | 0.5607 |
|  |  |  |  | Q9CRD2 | 0.5607 |
|  |  |  |  | Q3U9G9 | 0.5606 |
|  |  |  |  | Q6PF96 | 0.5606 |
|  |  |  |  | Q8CHK3 | 0.5605 |
|  |  |  |  | P61082 | 0.5604 |
|  |  |  |  | Q9EPE9 | 0.5603 |
|  |  |  |  | B8X349 | 0.5603 |
|  |  |  |  | D5MCW4 | 0.5602 |
|  |  |  |  | Q3U868 | 0.5602 |
|  |  |  |  | Q8VDG8 | 0.5602 |
|  |  |  |  | Q3UDX4 | 0.5601 |
|  |  |  |  | Q9D4H8 | 0.5600 |
|  |  |  |  | P36916 | 0.5600 |
|  |  |  |  | Q07417 | 0.5600 |
|  |  |  |  | O54833 | 0.5600 |
|  |  |  |  | E9Q0W8 | 0.5599 |
|  |  |  |  | Q8BRF7 | 0.5599 |
|  |  |  |  | G3X922 | 0.5599 |
|  |  |  |  | G3UYI5 | 0.5598 |
|  |  |  |  | O54774 | 0.5598 |
|  |  |  |  | Q6ZWZ6 | 0.5598 |
|  |  |  |  | Q91YU8 | 0.5598 |
|  |  |  |  | A2AKI5 | 0.5597 |
|  |  |  |  | Q3U3B9 | 0.5596 |
|  |  |  |  | Q922D8 | 0.5595 |
|  |  |  |  | O88477 | 0.5592 |
|  |  |  |  | Q9D1R9 | 0.5592 |
|  |  |  |  | Q922J3 | 0.5592 |
|  |  |  |  | Q3U3C4 | 0.5592 |
|  |  |  |  | E9PZF0 | 0.5591 |
|  |  |  |  | Q8VCT3 | 0.5591 |
|  |  |  |  | Q9D883 | 0.5591 |
|  |  |  |  | Q99KD5 | 0.5590 |
|  |  |  |  | Q8BFY6 | 0.5590 |
|  |  |  |  | F8VQJ3 | 0.5589 |
|  |  |  |  | P63001 | 0.5589 |
|  |  |  |  | Q7TNE3 | 0.5589 |
|  |  |  |  | Q99LC3 | 0.5589 |
|  |  |  |  | F8WH20 | 0.5588 |
|  |  |  |  | Q91WQ3 | 0.5588 |
|  |  |  |  | J3QQ30 | 0.5588 |
|  |  |  |  | O08553 | 0.5588 |
|  |  |  |  | E9QN31 | 0.5588 |
|  |  |  |  | Q6DFW4 | 0.5588 |
|  |  |  |  | D3YTQ9 | 0.5587 |
|  |  |  |  | Q6P069 | 0.5587 |
|  |  |  |  | Q9DBL7 | 0.5584 |
|  |  |  |  | P43274 | 0.5583 |
|  |  |  |  | Q7TNV0 | 0.5582 |
|  |  |  |  | Q8BKC5 | 0.5582 |
|  |  |  |  | A7ISP9 | 0.5581 |
|  |  |  |  | D3Z0B9 | 0.5581 |
|  |  |  |  | Q9QXK3 | 0.5581 |
|  |  |  |  | Q9WV98 | 0.5581 |
|  |  |  |  | O88653 | 0.5580 |
|  |  |  |  | Q5D0F3 | 0.5579 |
|  |  |  |  | O54692 | 0.5577 |
|  |  |  |  | P99027 | 0.5577 |
|  |  |  |  | P58252 | 0.5577 |
|  |  |  |  | Q9DBR0 | 0.5576 |
|  |  |  |  | P14869 | 0.5576 |
|  |  |  |  | Q3TIU7 | 0.5576 |
|  |  |  |  | Q8R0F6 | 0.5576 |
|  |  |  |  | P49717 | 0.5575 |
|  |  |  |  | Q99JW2 | 0.5575 |
|  |  |  |  | Q3TDE6 | 0.5575 |
|  |  |  |  | Q8C1E7 | 0.5574 |
|  |  |  |  | P07091 | 0.5574 |
|  |  |  |  | Q9D554 | 0.5574 |
|  |  |  |  | D3YVW2 | 0.5572 |
|  |  |  |  | Q91V64 | 0.5571 |
|  |  |  |  | Q05DE0 | 0.5571 |
|  |  |  |  | Q9D0I9 | 0.5570 |
|  |  |  |  | Q5HZH2 | 0.5570 |
|  |  |  |  | P70388 | 0.5570 |
|  |  |  |  | Q91VJ4 | 0.5569 |
|  |  |  |  | Q569X8 | 0.5569 |
|  |  |  |  | Q6NVC2 | 0.5568 |
|  |  |  |  | Q62348 | 0.5568 |
|  |  |  |  | Q61792 | 0.5566 |
|  |  |  |  | Q80UJ7 | 0.5566 |
|  |  |  |  | Q8CGZ0 | 0.5566 |
|  |  |  |  | E9PWG6 | 0.5565 |
|  |  |  |  | O35130 | 0.5565 |
|  |  |  |  | Q8C4B4 | 0.5565 |
|  |  |  |  | P46061 | 0.5564 |
|  |  |  |  | P70188 | 0.5564 |
|  |  |  |  | Q5DU34 | 0.5564 |
|  |  |  |  | Q8C140 | 0.5563 |
|  |  |  |  | D3YUM1 | 0.5563 |
|  |  |  |  | A0JNY7 | 0.5562 |
|  |  |  |  | P69566 | 0.5561 |
|  |  |  |  | P49718 | 0.5559 |
|  |  |  |  | F8WJG3 | 0.5557 |
|  |  |  |  | A2A4Z1 | 0.5557 |
|  |  |  |  | Q8CC88 | 0.5557 |
|  |  |  |  | Q60692 | 0.5556 |
|  |  |  |  | E9Q9A5 | 0.5555 |
|  |  |  |  | G5E829 | 0.5555 |
|  |  |  |  | P97825 | 0.5555 |
|  |  |  |  | Q9D6R2 | 0.5554 |
|  |  |  |  | Q5BKS2 | 0.5554 |
|  |  |  |  | E9Q7G1 | 0.5553 |
|  |  |  |  | E9PUC2 | 0.5553 |
|  |  |  |  | P47915 | 0.5553 |
|  |  |  |  | O55142 | 0.5551 |
|  |  |  |  | P12787 | 0.5551 |
|  |  |  |  | Q3UKJ7 | 0.5551 |
|  |  |  |  | F8WGB3 | 0.5551 |
|  |  |  |  | Q00612 | 0.5548 |
|  |  |  |  | P26638 | 0.5548 |
|  |  |  |  | Q9CW46 | 0.5548 |
|  |  |  |  | Q3TXH6 | 0.5547 |
|  |  |  |  | P28474 | 0.5546 |
|  |  |  |  | Q8VI84 | 0.5546 |
|  |  |  |  | Q3V1L4 | 0.5544 |
|  |  |  |  | Q9CXW2 | 0.5543 |
|  |  |  |  | P97310 | 0.5542 |
|  |  |  |  | Q02053 | 0.5542 |
|  |  |  |  | Q3TF41 | 0.5541 |
|  |  |  |  | Q9Z204 | 0.5541 |
|  |  |  |  | Q3UG53 | 0.5541 |
|  |  |  |  | P23116 | 0.5540 |
|  |  |  |  | P61161 | 0.5539 |
|  |  |  |  | Q9CQF3 | 0.5539 |
|  |  |  |  | P05201 | 0.5538 |
|  |  |  |  | G3X9V2 | 0.5538 |
|  |  |  |  | Q8BU88 | 0.5536 |
|  |  |  |  | E9Q585 | 0.5535 |
|  |  |  |  | Q91YP0 | 0.5535 |
|  |  |  |  | Q08093 | 0.5535 |
|  |  |  |  | Q5XG71 | 0.5534 |
|  |  |  |  | Q8VBT9 | 0.5534 |
|  |  |  |  | Q9D0G0 | 0.5534 |
|  |  |  |  | Q3THG9 | 0.5533 |
|  |  |  |  | P68040 | 0.5533 |
|  |  |  |  | Q3ULZ3 | 0.5533 |
|  |  |  |  | P62317 | 0.5533 |
|  |  |  |  | Q8BR63 | 0.5532 |
|  |  |  |  | Q9CX34 | 0.5532 |
|  |  |  |  | Q8BJW5 | 0.5530 |
|  |  |  |  | Q9WUM5 | 0.5530 |
|  |  |  |  | O88792 | 0.5529 |
|  |  |  |  | Q8VDP6 | 0.5528 |
|  |  |  |  | O89110 | 0.5527 |
|  |  |  |  | Q3TJ76 | 0.5527 |
|  |  |  |  | Q2VPC9 | 0.5527 |
|  |  |  |  | Q569Z5 | 0.5526 |
|  |  |  |  | Q9EPU0 | 0.5526 |
|  |  |  |  | Q8C878 | 0.5526 |
|  |  |  |  | Q8K363 | 0.5523 |
|  |  |  |  | Q91VH6 | 0.5523 |
|  |  |  |  | Q569Z6 | 0.5522 |
|  |  |  |  | B7ZWL1 | 0.5522 |
|  |  |  |  | Q8C5G6 | 0.5522 |
|  |  |  |  | Q3UK38 | 0.5519 |
|  |  |  |  | Q9CZ04 | 0.5519 |
|  |  |  |  | Q80Y14 | 0.5518 |
|  |  |  |  | O08810 | 0.5518 |
|  |  |  |  | Q9CS42 | 0.5517 |
|  |  |  |  | Q9DCU6 | 0.5515 |
|  |  |  |  | Q3TXS9 | 0.5515 |
|  |  |  |  | P62245 | 0.5514 |
|  |  |  |  | Q8VCH8 | 0.5514 |
|  |  |  |  | A2AL12 | 0.5514 |
|  |  |  |  | Q8R050 | 0.5514 |
|  |  |  |  | Q9D0T1 | 0.5513 |
|  |  |  |  | Q3UNN4 | 0.5513 |
|  |  |  |  | Q9R0P5 | 0.5511 |
|  |  |  |  | Q62351 | 0.5510 |
|  |  |  |  | P35293 | 0.5510 |
|  |  |  |  | Q9QZD9 | 0.5508 |
|  |  |  |  | P17426 | 0.5508 |
|  |  |  |  | Q8BK66 | 0.5507 |
|  |  |  |  | A2A547 | 0.5506 |
|  |  |  |  | Q8BK72 | 0.5506 |
|  |  |  |  | Q60865 | 0.5506 |
|  |  |  |  | Q91VG6 | 0.5505 |
|  |  |  |  | Q7TSC1 | 0.5505 |
|  |  |  |  | Q8VEE4 | 0.5504 |
|  |  |  |  | O35215 | 0.5502 |
|  |  |  |  | P62855 | 0.5502 |
|  |  |  |  | Q9DCT2 | 0.5502 |
|  |  |  |  | P63005 | 0.5500 |
|  |  |  |  | B8ZXI1 | 0.5500 |
|  |  |  |  | Q91V89 | 0.5500 |
|  |  |  |  | O88848 | 0.5499 |
|  |  |  |  | Q8VDF2 | 0.5497 |
|  |  |  |  | Q3UMT7 | 0.5497 |
|  |  |  |  | P16332 | 0.5496 |
|  |  |  |  | Q3UDH4 | 0.5495 |
|  |  |  |  | Q9D8E6 | 0.5495 |
|  |  |  |  | P14148 | 0.5494 |
|  |  |  |  | G3UWE1 | 0.5493 |
|  |  |  |  | Q921R2 | 0.5492 |
|  |  |  |  | Q3TWP9 | 0.5492 |
|  |  |  |  | Q8CFQ9 | 0.5490 |
|  |  |  |  | Q3UJX2 | 0.5490 |
|  |  |  |  | E9PX48 | 0.5489 |
|  |  |  |  | Q05816 | 0.5487 |
|  |  |  |  | Q5U4C3 | 0.5486 |
|  |  |  |  | B2RSW8 | 0.5486 |
|  |  |  |  | B7ZP20 | 0.5485 |
|  |  |  |  | A2AN08 | 0.5483 |
|  |  |  |  | A2BE28 | 0.5482 |
|  |  |  |  | P62315 | 0.5482 |
|  |  |  |  | Q9CRC8 | 0.5482 |
|  |  |  |  | Q61033 | 0.5480 |
|  |  |  |  | Q3UF95 | 0.5480 |
|  |  |  |  | Q9CPP6 | 0.5480 |
|  |  |  |  | Q9EP82 | 0.5480 |
|  |  |  |  | E9QMV2 | 0.5480 |
|  |  |  |  | Q3U0I9 | 0.5479 |
|  |  |  |  | Q3UA17 | 0.5479 |
|  |  |  |  | Q8K366 | 0.5479 |
|  |  |  |  | O54984 | 0.5479 |
|  |  |  |  | P49586 | 0.5479 |
|  |  |  |  | B2RUG7 | 0.5479 |
|  |  |  |  | P54103 | 0.5478 |
|  |  |  |  | Q61171 | 0.5477 |
|  |  |  |  | P32233 | 0.5477 |
|  |  |  |  | Q3TZP3 | 0.5476 |
|  |  |  |  | Q3UDM8 | 0.5476 |
|  |  |  |  | Q91V55 | 0.5475 |
|  |  |  |  | Q3TE45 | 0.5474 |
|  |  |  |  | Q99JB8 | 0.5473 |
|  |  |  |  | Q80TU6 | 0.5472 |
|  |  |  |  | Q8CGF7 | 0.5472 |
|  |  |  |  | I6L960 | 0.5472 |
|  |  |  |  | P42128 | 0.5471 |
|  |  |  |  | P06797 | 0.5469 |
|  |  |  |  | A0A087WSG5 | 0.5468 |
|  |  |  |  | O08997 | 0.5468 |
|  |  |  |  | Q9Z2B9 | 0.5467 |
|  |  |  |  | Q14AX9 | 0.5466 |
|  |  |  |  | Q8K2B3 | 0.5464 |
|  |  |  |  | Q9CPT5 | 0.5463 |
|  |  |  |  | Q9WTP6 | 0.5462 |
|  |  |  |  | Q3TUQ7 | 0.5462 |
|  |  |  |  | I7HLV2 | 0.5462 |
|  |  |  |  | P97351 | 0.5458 |
|  |  |  |  | Q11136 | 0.5458 |
|  |  |  |  | Q9JJX7 | 0.5456 |
|  |  |  |  | Q9DC61 | 0.5455 |
|  |  |  |  | Q9ESY9 | 0.5454 |
|  |  |  |  | I4DCY6 | 0.5453 |
|  |  |  |  | Q8BSL7 | 0.5453 |
|  |  |  |  | Q61584 | 0.5452 |
|  |  |  |  | F6RJV6 | 0.5452 |
|  |  |  |  | Q9D104 | 0.5451 |
|  |  |  |  | Q3USC0 | 0.5451 |
|  |  |  |  | P26040 | 0.5450 |
|  |  |  |  | Q8R3X4 | 0.5450 |
|  |  |  |  | P29341 | 0.5449 |
|  |  |  |  | P47955 | 0.5448 |
|  |  |  |  | Q8VED9 | 0.5448 |
|  |  |  |  | Q3TVV6 | 0.5444 |
|  |  |  |  | E9PUB7 | 0.5444 |
|  |  |  |  | Q4KMV6 | 0.5443 |
|  |  |  |  | Q9WVS5 | 0.5443 |
|  |  |  |  | O08807 | 0.5443 |
|  |  |  |  | Q1KYM2 | 0.5443 |
|  |  |  |  | P70279 | 0.5441 |
|  |  |  |  | Q8C0Z3 | 0.5441 |
|  |  |  |  | Q9D2M8 | 0.5440 |
|  |  |  |  | D3Z5I1 | 0.5439 |
|  |  |  |  | Q8BHL5 | 0.5437 |
|  |  |  |  | P28660 | 0.5436 |
|  |  |  |  | Q6P8N8 | 0.5434 |
|  |  |  |  | Q3U3C2 | 0.5433 |
|  |  |  |  | Q8VHZ7 | 0.5433 |
|  |  |  |  | O70503 | 0.5431 |
|  |  |  |  | Q9R059 | 0.5430 |
|  |  |  |  | Q80W68 | 0.5430 |
|  |  |  |  | Q8C2T9 | 0.5429 |
|  |  |  |  | Q64674 | 0.5428 |
|  |  |  |  | B2RY90 | 0.5428 |
|  |  |  |  | O88569 | 0.5426 |
|  |  |  |  | D3YWK1 | 0.5425 |
|  |  |  |  | Q3UZJ4 | 0.5425 |
|  |  |  |  | D3YUK4 | 0.5423 |
|  |  |  |  | Q8BMG7 | 0.5421 |
|  |  |  |  | A2AWT6 | 0.5421 |
|  |  |  |  | O54724 | 0.5421 |
|  |  |  |  | Q9CQZ5 | 0.5420 |
|  |  |  |  | Q3UJZ7 | 0.5419 |
|  |  |  |  | Q05BD4 | 0.5419 |
|  |  |  |  | Q921M3 | 0.5418 |
|  |  |  |  | Q8CJ26 | 0.5416 |
|  |  |  |  | Q6A068 | 0.5415 |
|  |  |  |  | Q3TMB5 | 0.5414 |
|  |  |  |  | Q8R1Q8 | 0.5414 |
|  |  |  |  | Q3UK68 | 0.5412 |
|  |  |  |  | Q9CQ60 | 0.5408 |
|  |  |  |  | Q3TJN6 | 0.5408 |
|  |  |  |  | Q3ULG4 | 0.5407 |
|  |  |  |  | B2RQS1 | 0.5404 |
|  |  |  |  | Q3TI79 | 0.5404 |
|  |  |  |  | Q3UT23 | 0.5403 |
|  |  |  |  | Q9Z2Y8 | 0.5403 |
|  |  |  |  | P41105 | 0.5403 |
|  |  |  |  | Q9CQM9 | 0.5402 |
|  |  |  |  | P47968 | 0.5399 |
|  |  |  |  | Q6PDM2 | 0.5399 |
|  |  |  |  | Q03145 | 0.5399 |
|  |  |  |  | Q3T992 | 0.5398 |
|  |  |  |  | E9Q715 | 0.5396 |
|  |  |  |  | P10518 | 0.5396 |
|  |  |  |  | Q3UU20 | 0.5396 |
|  |  |  |  | Q9JJA4 | 0.5392 |
|  |  |  |  | P05622 | 0.5392 |
|  |  |  |  | Q9D051 | 0.5391 |
|  |  |  |  | B1AZI6 | 0.5390 |
|  |  |  |  | Q9D2G2 | 0.5390 |
|  |  |  |  | P57784 | 0.5389 |
|  |  |  |  | Q922S4 | 0.5389 |
|  |  |  |  | Q99J62 | 0.5387 |
|  |  |  |  | Q8BGH2 | 0.5386 |
|  |  |  |  | P49962 | 0.5386 |
|  |  |  |  | Q8BFY9 | 0.5383 |
|  |  |  |  | A0A087WPL5 | 0.5383 |
|  |  |  |  | Q8K0D5 | 0.5382 |
|  |  |  |  | Q3V117 | 0.5381 |
|  |  |  |  | D3Z0A2 | 0.5381 |
|  |  |  |  | Q3UN02 | 0.5381 |
|  |  |  |  | Q3V212 | 0.5380 |
|  |  |  |  | Q3TL79 | 0.5378 |
|  |  |  |  | Q8CHW4 | 0.5377 |
|  |  |  |  | P18155 | 0.5377 |
|  |  |  |  | P55264 | 0.5376 |
|  |  |  |  | G3XA25 | 0.5375 |
|  |  |  |  | Q8VHY0 | 0.5375 |
|  |  |  |  | P68181 | 0.5375 |
|  |  |  |  | Q9CQI7 | 0.5372 |
|  |  |  |  | Q9CZX8 | 0.5371 |
|  |  |  |  | Q8BUM1 | 0.5371 |
|  |  |  |  | P62702 | 0.5371 |
|  |  |  |  | Q99ME9 | 0.5368 |
|  |  |  |  | Q8VI75 | 0.5367 |
|  |  |  |  | P10852 | 0.5363 |
|  |  |  |  | Q3U6X7 | 0.5363 |
|  |  |  |  | D3YVV7 | 0.5362 |
|  |  |  |  | Q9Z2I9 | 0.5362 |
|  |  |  |  | E9QKA4 | 0.5362 |
|  |  |  |  | Q8R3C6 | 0.5362 |
|  |  |  |  | H3BKK2 | 0.5362 |
|  |  |  |  | P48962 | 0.5359 |
|  |  |  |  | Q04750 | 0.5359 |
|  |  |  |  | D3Z4M2 | 0.5354 |
|  |  |  |  | Q9CYL5 | 0.5352 |
|  |  |  |  | Q91WG4 | 0.5351 |
|  |  |  |  | P35505 | 0.5350 |
|  |  |  |  | A2AW05 | 0.5350 |
|  |  |  |  | Q80UE5 | 0.5349 |
|  |  |  |  | Q91WD5 | 0.5346 |
|  |  |  |  | Q5U5I3 | 0.5345 |
|  |  |  |  | Q06138 | 0.5345 |
|  |  |  |  | Q9JI13 | 0.5345 |
|  |  |  |  | P61290 | 0.5344 |
|  |  |  |  | Q3UHD6 | 0.5344 |
|  |  |  |  | P26039 | 0.5344 |
|  |  |  |  | F8VPN4 | 0.5343 |
|  |  |  |  | A0A087WNZ7 | 0.5343 |
|  |  |  |  | Q80XR5 | 0.5341 |
|  |  |  |  | Q8BTW7 | 0.5337 |
|  |  |  |  | Q9WTI7 | 0.5337 |
|  |  |  |  | P05132 | 0.5336 |
|  |  |  |  | Q99K28 | 0.5335 |
|  |  |  |  | Q3UVI9 | 0.5333 |
|  |  |  |  | A2ATP5 | 0.5333 |
|  |  |  |  | Q9DC16 | 0.5333 |
|  |  |  |  | Q9D1L9 | 0.5332 |
|  |  |  |  | Q9EPL8 | 0.5331 |
|  |  |  |  | Q61686 | 0.5330 |
|  |  |  |  | P70698 | 0.5330 |
|  |  |  |  | Q8K3H0 | 0.5328 |
|  |  |  |  | Q6DFV1 | 0.5328 |
|  |  |  |  | Q1W5W7 | 0.5326 |
|  |  |  |  | Q9CX86 | 0.5326 |
|  |  |  |  | Q05CC5 | 0.5325 |
|  |  |  |  | Q91V01 | 0.5325 |
|  |  |  |  | Q3UM45 | 0.5318 |
|  |  |  |  | P59325 | 0.5317 |
|  |  |  |  | Q3U935 | 0.5317 |
|  |  |  |  | Q99JY0 | 0.5314 |
|  |  |  |  | Q6A099 | 0.5314 |
|  |  |  |  | Q9D0M3 | 0.5314 |
|  |  |  |  | Q8R509 | 0.5314 |
|  |  |  |  | E9QND8 | 0.5313 |
|  |  |  |  | Q9ES97 | 0.5313 |
|  |  |  |  | Q3V222 | 0.5312 |
|  |  |  |  | Q9R1P3 | 0.5311 |
|  |  |  |  | Q3U1J4 | 0.5310 |
|  |  |  |  | Q3B7Z2 | 0.5309 |
|  |  |  |  | Q3UX26 | 0.5302 |
|  |  |  |  | P14152 | 0.5301 |
|  |  |  |  | Q3UJN6 | 0.5300 |
|  |  |  |  | P10107 | 0.5300 |
|  |  |  |  | Q9CTT4 | 0.5298 |
|  |  |  |  | Q3UXI9 | 0.5296 |
|  |  |  |  | Q8QZY1 | 0.5296 |
|  |  |  |  | Q3UGR5 | 0.5291 |
|  |  |  |  | Q3U5L3 | 0.5291 |
|  |  |  |  | Q3TKC5 | 0.5290 |
|  |  |  |  | A0JLN6 | 0.5290 |
|  |  |  |  | Q7TMF3 | 0.5288 |
|  |  |  |  | Q8VCW8 | 0.5288 |
|  |  |  |  | P52432 | 0.5285 |
|  |  |  |  | Q80X95 | 0.5284 |
|  |  |  |  | Q9DC69 | 0.5283 |
|  |  |  |  | Q80SZ7 | 0.5282 |
|  |  |  |  | Q80YR5 | 0.5281 |
|  |  |  |  | D3Z0M9 | 0.5279 |
|  |  |  |  | Q9CQN7 | 0.5277 |
|  |  |  |  | Q8CE21 | 0.5277 |
|  |  |  |  | O88844 | 0.5276 |
|  |  |  |  | Q9DBU8 | 0.5276 |
|  |  |  |  | Q3U4F0 | 0.5275 |
|  |  |  |  | D6RGL6 | 0.5274 |
|  |  |  |  | O35326 | 0.5274 |
|  |  |  |  | Q924T2 | 0.5271 |
|  |  |  |  | P54822 | 0.5270 |
|  |  |  |  | G3UZ26 | 0.5270 |
|  |  |  |  | O08582 | 0.5265 |
|  |  |  |  | H3BJU7 | 0.5261 |
|  |  |  |  | Q78RK2 | 0.5259 |
|  |  |  |  | Q6ZWN5 | 0.5257 |
|  |  |  |  | D3Z0G0 | 0.5257 |
|  |  |  |  | D3Z4U3 | 0.5256 |
|  |  |  |  | O88587 | 0.5255 |
|  |  |  |  | Q9CQW1 | 0.5255 |
|  |  |  |  | Q3U6Q1 | 0.5254 |
|  |  |  |  | O70569 | 0.5254 |
|  |  |  |  | P16125 | 0.5253 |
|  |  |  |  | P48771 | 0.5252 |
|  |  |  |  | P49817 | 0.5251 |
|  |  |  |  | Q5EBG5 | 0.5250 |
|  |  |  |  | D3Z041 | 0.5250 |
|  |  |  |  | Q78JE5 | 0.5248 |
|  |  |  |  | Q3U561 | 0.5247 |
|  |  |  |  | Q9ERK4 | 0.5246 |
|  |  |  |  | Q9ERS2 | 0.5246 |
|  |  |  |  | Q9JIQ4 | 0.5246 |
|  |  |  |  | Q3UHZ3 | 0.5244 |
|  |  |  |  | O35218 | 0.5241 |
|  |  |  |  | P97384 | 0.5241 |
|  |  |  |  | P04104 | 0.5241 |
|  |  |  |  | P62806 | 0.5240 |
|  |  |  |  | Q3U6U7 | 0.5240 |
|  |  |  |  | E9QAZ2 | 0.5240 |
|  |  |  |  | P52293 | 0.5238 |
|  |  |  |  | P54116 | 0.5238 |
|  |  |  |  | Q3TG58 | 0.5237 |
|  |  |  |  | Q4TVN0 | 0.5237 |
|  |  |  |  | Q3UKP5 | 0.5236 |
|  |  |  |  | Q80VI1 | 0.5232 |
|  |  |  |  | Q6NZM8 | 0.5232 |
|  |  |  |  | Q91VJ2 | 0.5230 |
|  |  |  |  | Q3TFF0 | 0.5230 |
|  |  |  |  | Q9D2Y4 | 0.5229 |
|  |  |  |  | Q3TDF8 | 0.5229 |
|  |  |  |  | Q3UJ70 | 0.5227 |
|  |  |  |  | A2AE27 | 0.5227 |
|  |  |  |  | Q6NV83 | 0.5224 |
|  |  |  |  | Q8R502 | 0.5223 |
|  |  |  |  | Q64261 | 0.5222 |
|  |  |  |  | G3UWG1 | 0.5222 |
|  |  |  |  | Q9CQB4 | 0.5220 |
|  |  |  |  | F8WH41 | 0.5219 |
|  |  |  |  | Q3TZU7 | 0.5217 |
|  |  |  |  | Q3UPG1 | 0.5216 |
|  |  |  |  | Q9DCL9 | 0.5216 |
|  |  |  |  | Q3UVK0 | 0.5215 |
|  |  |  |  | Q9EQ61 | 0.5214 |
|  |  |  |  | Q3TAV1 | 0.5213 |
|  |  |  |  | Q99JX4 | 0.5202 |
|  |  |  |  | G3UYD0 | 0.5197 |
|  |  |  |  | P62267 | 0.5197 |
|  |  |  |  | Q9JJI8 | 0.5196 |
|  |  |  |  | Q3UT19 | 0.5195 |
|  |  |  |  | Q9WVA3 | 0.5191 |
|  |  |  |  | A2AU61 | 0.5189 |
|  |  |  |  | Q3UAY3 | 0.5189 |
|  |  |  |  | Q91V76 | 0.5189 |
|  |  |  |  | Q3UJS6 | 0.5188 |
|  |  |  |  | Q9CZX0 | 0.5186 |
|  |  |  |  | Q8K2M0 | 0.5186 |
|  |  |  |  | Q9DB20 | 0.5186 |
|  |  |  |  | Q8C2D1 | 0.5181 |
|  |  |  |  | Q61249 | 0.5178 |
|  |  |  |  | Q3U9A8 | 0.5175 |
|  |  |  |  | Q9CWZ5 | 0.5173 |
|  |  |  |  | P45952 | 0.5169 |
|  |  |  |  | Q60676 | 0.5167 |
|  |  |  |  | Q80UU6 | 0.5166 |
|  |  |  |  | Q5FWK3 | 0.5165 |
|  |  |  |  | P97477 | 0.5160 |
|  |  |  |  | O08749 | 0.5157 |
|  |  |  |  | Q8VC28 | 0.5156 |
|  |  |  |  | A2BDX2 | 0.5156 |
|  |  |  |  | P60469 | 0.5153 |
|  |  |  |  | Q62425 | 0.5153 |
|  |  |  |  | Q6PAI8 | 0.5152 |
|  |  |  |  | Q3TD51 | 0.5151 |
|  |  |  |  | Q8K3J2 | 0.5150 |
|  |  |  |  | Q9D967 | 0.5145 |
|  |  |  |  | Q8C2Q3 | 0.5145 |
|  |  |  |  | Q80YR4 | 0.5144 |
|  |  |  |  | Q9DB77 | 0.5140 |
|  |  |  |  | Q8BP67 | 0.5137 |
|  |  |  |  | Q3TN42 | 0.5130 |
|  |  |  |  | K7Q751 | 0.5128 |
|  |  |  |  | F6VG18 | 0.5124 |
|  |  |  |  | F6ZFU0 | 0.5123 |
|  |  |  |  | Q5SWD9 | 0.5121 |
|  |  |  |  | E9PZK7 | 0.5108 |
|  |  |  |  | Q9JMD0 | 0.5104 |
|  |  |  |  | A7M7Q8 | 0.5103 |
|  |  |  |  | A0A068BIT8 | 0.5100 |
|  |  |  |  | Q5SS83 | 0.5099 |
|  |  |  |  | F8VPX1 | 0.5098 |
|  |  |  |  | A0A0A6YVU8 | 0.5097 |
|  |  |  |  | Q9Z130 | 0.5093 |
|  |  |  |  | Q9JJ94 | 0.5092 |
|  |  |  |  | Q3U6F1 | 0.5091 |
|  |  |  |  | P28271 | 0.5087 |
|  |  |  |  | P62889 | 0.5084 |
|  |  |  |  | P04117 | 0.5079 |
|  |  |  |  | P13439 | 0.5078 |
|  |  |  |  | Q91W92 | 0.5074 |
|  |  |  |  | Q62159 | 0.5067 |
|  |  |  |  | Q8K3X4 | 0.5064 |
|  |  |  |  | Q61206 | 0.5061 |
|  |  |  |  | Z4YKM2 | 0.5059 |
|  |  |  |  | E9PYJ6 | 0.5058 |
|  |  |  |  | O54784 | 0.5053 |
|  |  |  |  | Q3TCE7 | 0.5050 |
|  |  |  |  | Q922D4 | 0.5049 |
|  |  |  |  | Q8BWZ3 | 0.5049 |
|  |  |  |  | Q9WVQ5 | 0.5047 |
|  |  |  |  | P63276 | 0.5043 |
|  |  |  |  | Q7TQK1 | 0.5035 |
|  |  |  |  | Q6GU23 | 0.5032 |
|  |  |  |  | A0A087WSP0 | 0.5030 |
|  |  |  |  | Q8BVF2 | 0.5029 |
|  |  |  |  | Q8BLN5 | 0.5028 |
|  |  |  |  | Q8BYY4 | 0.5025 |
|  |  |  |  | Q99NB8 | 0.5022 |
|  |  |  |  | S4R1L5 | 0.5021 |
|  |  |  |  | A0A087WQE6 | 0.5021 |
|  |  |  |  | Q7TPY3 | 0.5018 |
|  |  |  |  | Q99KD6 | 0.5015 |
|  |  |  |  | Q6PJN8 | 0.5013 |
|  |  |  |  | Q61136 | 0.5012 |
|  |  |  |  | Q8BXV2 | 0.5002 |
|  |  |  |  | Q9CXV9 | 0.4998 |
|  |  |  |  | Q9JI75 | 0.4995 |
|  |  |  |  | Q3UTW6 | 0.4994 |
|  |  |  |  | Q8CGP5 | 0.4993 |
|  |  |  |  | P19096 | 0.4992 |
|  |  |  |  | Q9CQ75 | 0.4989 |
|  |  |  |  | P19536 | 0.4988 |
|  |  |  |  | Q9DCB8 | 0.4986 |
|  |  |  |  | Q3TCZ2 | 0.4986 |
|  |  |  |  | P08074 | 0.4985 |
|  |  |  |  | Q5SSZ5 | 0.4977 |
|  |  |  |  | Q8VCN5 | 0.4975 |
|  |  |  |  | F8WHV1 | 0.4974 |
|  |  |  |  | Q80VD1 | 0.4974 |
|  |  |  |  | Q3U617 | 0.4969 |
|  |  |  |  | Q9D6J6 | 0.4969 |
|  |  |  |  | E9Q933 | 0.4966 |
|  |  |  |  | Q9CQM5 | 0.4965 |
|  |  |  |  | O88271 | 0.4957 |
|  |  |  |  | Q3TIJ4 | 0.4957 |
|  |  |  |  | O89086 | 0.4954 |
|  |  |  |  | Q91YR1 | 0.4951 |
|  |  |  |  | Q3V300 | 0.4947 |
|  |  |  |  | Q05DU8 | 0.4943 |
|  |  |  |  | Q5SUF2 | 0.4932 |
|  |  |  |  | Q9D7S7 | 0.4925 |
|  |  |  |  | Q6P289 | 0.4923 |
|  |  |  |  | Q9JKB3 | 0.4921 |
|  |  |  |  | Q3TAP5 | 0.4907 |
|  |  |  |  | P62270 | 0.4905 |
|  |  |  |  | D3YZC1 | 0.4904 |
|  |  |  |  | E9Q555 | 0.4904 |
|  |  |  |  | P58742 | 0.4895 |
|  |  |  |  | P27046 | 0.4894 |
|  |  |  |  | Q3UAX2 | 0.4892 |
|  |  |  |  | F6QKD2 | 0.4890 |
|  |  |  |  | Q3TMP1 | 0.4887 |
|  |  |  |  | Q3UY05 | 0.4885 |
|  |  |  |  | Q9D2R0 | 0.4878 |
|  |  |  |  | Q8K2V6 | 0.4870 |
|  |  |  |  | Q6PGH6 | 0.4866 |
|  |  |  |  | Q9R062 | 0.4857 |
|  |  |  |  | Q62241 | 0.4854 |
|  |  |  |  | Q9DBY8 | 0.4853 |
|  |  |  |  | Q8CH72 | 0.4847 |
|  |  |  |  | Q0VGU9 | 0.4846 |
|  |  |  |  | Q80SW1 | 0.4845 |
|  |  |  |  | Q5SWU9 | 0.4845 |
|  |  |  |  | Q99LS3 | 0.4839 |
|  |  |  |  | O54825 | 0.4825 |
|  |  |  |  | G3X8X7 | 0.4822 |
|  |  |  |  | Q3TP27 | 0.4819 |
|  |  |  |  | D3YWT1 | 0.4818 |
|  |  |  |  | A2A9X5 | 0.4806 |
|  |  |  |  | A0PJ90 | 0.4804 |
|  |  |  |  | Q9ET22 | 0.4802 |
|  |  |  |  | E9PW15 | 0.4797 |
|  |  |  |  | Q3TV93 | 0.4789 |
|  |  |  |  | D3YVX4 | 0.4780 |
|  |  |  |  | Q04207 | 0.4771 |
|  |  |  |  | Q99P31 | 0.4771 |
|  |  |  |  | Q3UMQ8 | 0.4770 |
|  |  |  |  | Q9D8M4 | 0.4769 |
|  |  |  |  | Q3THZ8 | 0.4752 |
|  |  |  |  | Q6P9J9 | 0.4751 |
|  |  |  |  | B2RUG6 | 0.4746 |
|  |  |  |  | Q3U468 | 0.4744 |
|  |  |  |  | Q9CX60 | 0.4744 |
|  |  |  |  | O35075 | 0.4742 |
|  |  |  |  | Q8CCX9 | 0.4739 |
|  |  |  |  | Q8R5H1 | 0.4738 |
|  |  |  |  | P46935 | 0.4726 |
|  |  |  |  | Q8BY71 | 0.4717 |
|  |  |  |  | Q66L45 | 0.4712 |
|  |  |  |  | Q9D0L4 | 0.4710 |
|  |  |  |  | O08663 | 0.4687 |
|  |  |  |  | B7ZWM8 | 0.4683 |
|  |  |  |  | E0CXN5 | 0.4681 |
|  |  |  |  | Q99J93 | 0.4592 |
|  |  |  |  | Q9CR25 | 0.4586 |
|  |  |  |  | Q9D9V3 | 0.4585 |
|  |  |  |  | Q3US29 | 0.4582 |
|  |  |  |  | D3Z113 | 0.4566 |
|  |  |  |  | Q4VA28 | 0.4535 |
|  |  |  |  | F7A1B4 | 0.4534 |
|  |  |  |  | J3QK23 | 0.4521 |
|  |  |  |  | O35654 | 0.4508 |
|  |  |  |  | Q3TX72 | 0.4504 |
|  |  |  |  | P07607 | 0.4496 |
|  |  |  |  | Q6ZWU9 | 0.4489 |
|  |  |  |  | A0A0A6YXY1 | 0.4489 |
|  |  |  |  | Q9WUP7 | 0.4479 |
|  |  |  |  | E0CX20 | 0.4473 |
|  |  |  |  | Q3TEN9 | 0.4472 |
|  |  |  |  | Q8BGZ7 | 0.4464 |
|  |  |  |  | Q80UZ2 | 0.4405 |
|  |  |  |  | S4R1E5 | 0.4296 |
|  |  |  |  | Q5SSP3 | 0.4273 |
|  |  |  |  | Q3TC83 | 0.4266 |
|  |  |  |  | A0A0A6YW28 | 0.4184 |
|  |  |  |  | Q9DC42 | 0.4164 |
|  |  |  |  | Q3TV20 | 0.4158 |
|  |  |  |  | Q3U6Y9 | 0.4130 |
|  |  |  |  | D3YWS7 | 0.4103 |
|  |  |  |  | Q5M9N6 | 0.4094 |
|  |  |  |  | Q9JLI0 | 0.4071 |
|  |  |  |  | Q3U8R9 | 0.4070 |
|  |  |  |  | P07141 | 0.3995 |
|  |  |  |  | D3YXP6 | 0.3952 |
|  |  |  |  | E9PWQ3 | 0.3951 |
|  |  |  |  | P33610 | 0.3903 |
|  |  |  |  | Q3USG5 | 0.3863 |
|  |  |  |  | G3X9T8 | 0.3821 |
|  |  |  |  | Q4FK16 | 0.3775 |
|  |  |  |  | O08759 | 0.3750 |
|  |  |  |  | Q8VE10 | 0.3605 |
|  |  |  |  | P09528 | 0.3582 |
|  |  |  |  | O08915 | 0.3494 |
|  |  |  |  | Q3UFR4 | 0.3468 |
|  |  |  |  | Q8K0C4 | 0.3446 |
|  |  |  |  | Q8BFZ3 | 0.3380 |
|  |  |  |  | Q3THE6 | 0.3379 |
|  |  |  |  | Q64735 | 0.3244 |

**Supplementary Table S4.** Genes differentially expressed in 5, 6, 7 and 8 Ni^2+^-treated groups.

| **Differentially expressed in 5 groups** | **Differentially expressed in 6 groups** | **Differentially expressed in 7 groups** | **Differentially expressed in 8 groups** |
| --- | --- | --- | --- |
| *Grhpr* | *Apln* | *Zfhx3* | *Pdk1* |
| *Elp6* | *Cyb5a* | *Wfdc17* | *G630025P09Rik* |
| *Cth* | *Figf* | *Usp17la* | *Mgst3* |
| *Cpne2* | *Fbln2* | *Unc79* | *A930004D18Rik* |
| *Uqcrb* | *Hist1h1c* | *Traf3ip3* | *Serpine1* |
| *Podnl1* | *5430416O09Rik* | *Tenm2* | *Ldha* |
| *Angpt1* | *Olfml3* | *Slc5a4b* | *Pkm* |
| *Ucp2* | *Vim* | *Slc45a3* | *Plin4* |
| *Slc25a1* | *Gm17762* | *Slc25a35* | *Atxn7l1* |
| *Hirip3* | *Hk2* | *Slc25a34* | *Bnip3* |
| *Hist1h2bg* | *S100a4* | *Sgcd* | *Higd1a* |
| *Mt1* | *Tfrc* | *Selenbp2* | *Ankrd37* |
| *Gale* | *Impa2* | *Selenbp1* | *Bvht* |
| *Fabp4* | *Msmo1* | *Sdc4* | *Lgals3* |
| *Mt2* | *Pgd* | *S1pr3* | *Gbe1* |
| *Clca3a1* | *Pmvk* | *Rras2* | *Pgam1* |
| *3300005D01Rik* | *Ldlr* | *Rn45s* | *Kdm3a* |
| *Chpf2* | *Fdps* | *Rbms3* | *Pgm2* |
| *Cda* | *Lss* | *Raet1e* | *Serpinb9e* |
| *Serpina3f* | *Mfsd6* | *Pyhin1* | *Pfkl* |
| *Adamts10* | *Ank2* | *Prnd* | *Slc2a1* |
| *Slc39a10* | *Hes1* | *Plekha2* | *Ccng2* |
| *Cd74* | *Zdhhc15* | *Pde2a* | *Scd2* |
| *Nsd1* | *9530036O11Rik* | *Pcx* | *Ddit4* |
| *Dazl* | *Rtp3* | *Pcdhb4* | *Egln3* |
| *Camk1g* | *4930447C04Rik* | *Pcdha9* | *Zbtb8b* |
| *Paqr8* | *4930519G04Rik* | *Olfr536* | *Rtn4rl1* |
| *Greb1* | *H2-Eb1* | *Ndrg1* | *Mthfd1l* |
| *Slc6a17* | *Spry4* | *Mndal* | *Mcc* |
| *Creld2* | *H2-T22* | *Me1* | *Acsbg1* |
| *Lbp* | *Adnp* | *Mat2a* | *Egln1* |
| *Fah* | *Fam167b* | *Ly6a* | *Slc11a1* |
| *Junb* | *Ak5* | *Lgals2* | *5031425E22Rik* |
| *Egr2* | *Lrrc55* | *Kdm4b* | *Tpi1* |
| *Lbh* | *Dgka* | *Itih5l-ps* | *Fabp5* |
| *Kif5c* | *Gm15987* | *Itih2* | *Nxph4* |
| *Rnf145* | *Dusp2* | *Isg20* | *Gpr35* |
| *Gm2042* | *Cass4* | *Igfbp5* | *Adm* |
| *Mrvi1* | *Slfn2* | *Ier5l* | *Sorcs2* |
| *Tcn2* | *Serpina3g* | *Ier2* | *4631405J19Rik* |
| *Cd80* | *Prokr1* | *Hyal1* | *Htra3* |
| *Wfdc21* | *Fam26e* | *Hrct1* | *4930447F24Rik* |
| *Akr1b7* | *Grhl2* | *Hilpda* | *Crocc2* |
| *Hyou1* | *Pcdhb7* | *Herc6* | *Cox7a1* |
| *Hecw2* | *Esr1* | *H2-M10.3* | *Loxl2* |
| *Cyfip2* | *Mboat2* | *Gsta1* | *Aldoc* |
| *Kcnma1* | *Prrxl1* | *Gpr137c* | *Eno1b* |
| *Mmp9* | *Pld4* | *Gm5039* | *Adipor2* |
| *Prelp* | *F5* | *Gm4027* | *Slc16a3* |
| *Abca4* | *Dennd2a* | *Gm13275* | *Eno1* |
| *Icosl* | *Bche* | *Gm13247* | *Ak4* |
| *Gpr155* | *Ptprt* | *Glt8d2* | *Tnfaip3* |
| *Zfp36* | *Taf7l* | *Gdf15* | *Gapdh* |
| *Flrt1* | *Otoa* | *Gbp9* | *2810013P06Rik* |
| *Ccdc80* | *Slc28a2* | *Gbp8* | *Tnni3k* |
| *Iigp1* | *H2-M10.1* | *Gbp2* | *Hist2h2be* |
| *Tlr1* | *Hist2h4* | *Galnt7* | *Lman1l* |
| *Catsperg2* | *Paqr3* | *Fos* | *Bhlhe40* |
| *Prdm9* | *Btg2* | *Flrt2* | *Gys1* |
| *Naip1* | *Nrxn3* | *Fgf11* | *Pgk1* |
| *Chit1* | *Cyr61* | *Fgf1* | *Serpinb9f* |
| *Dtna* | *Dapk1* | *Fbxw27* | *Cst6* |
| *Optc* | *Btc* | *Esrrg* | *Gm1966* |
| *Ctso* | *Ggt7* | *Esd* | *Tspan2* |
| *Rdh12* | *Lrrc8c* | *Enpp2* | *Otog* |
| *Txndc16* | *Gm17757* | *Eno2* | *Usp17ld* |
| *Azgp1* | *Gfra1* | *Dusp6* | *Mctp1* |
| *Acot2* | *Fam43a* | *Dsc1* | *Armcx2* |
| *Nrip2* | *Tecta* | *Dpep1* | *Cpne4* |
| *Sema4f* | *Dhrs11* | *Dock2* | *Abca14* |
| *Cd53* | *Zfp455* | *Dclk1* | *Col14a1* |
| *Tns4* | *Gm6093* | *Cyp4f14* | *Lcn2* |
| *Zfp600* | *Oas3* | *Cxcl3* | *Cxcl2* |
| *Ggt1* | *Kalrn* | *Cox6a2* | *Trem3* |
| *Kctd14* | *Nat14* | *Col9a1* | *Steap4* |
| *Eya4* | *Adgrd1* | *Chrnd* | *Myct1* |
| *Them4* | *Gm13242* | *Ces2g* | *Pde1b* |
| *Trim12c* | *Faim2* | *Cebpd* | *Dach2* |
| *Snx31* | *Pla1a* | *Ccrl2* | *Myo5c* |
| *Islr* | *Sema6d* | *Ccdc58* | *Gm2016* |
| *AU021092* | *Sag* | *Car9* | *Nfe2* |
| *Plod1* | *Plod2* | *C4b* | *Trim9* |
| *Ankk1* | *Jmjd6* | *Bsn* | *Igtp* |
| *Slc16a4* | *Ndufa4l2* | *Atf3* | *Gm5662* |
| *Usp13* | *Mgst2* | *Art3* | *Mamdc2* |
| *B430319G15Rik* | *4931408D14Rik* | *Arhgef39* | *Nat8* |
| *Prelid2* | *A930033H14Rik* | *Arc* | *Stk39* |
| *Mif* | *Abhd18* | *Aoc2* | *Ccl20* |
| *Megf10* | *Nucb2* | *Aoah* | *Gm12185* |
| *Gnrh1* | *Mlph* | *Anxa2* | *Dock3* |
| *Ftl1* | *Itpk1* | *Ankrd34a* | *Col26a1* |
| *1700025G04Rik* | *Rec114* | *Aldoa* | *Csn3* |
| *Oit3* | *P4ha1* | *Acvr1c* | *Ednra* |
| *Sap30* | *Gm5893* | *Acox2* | *Fibin* |
| *Gemin4* | *F730043M19Rik* | *Acap1* | *Ms4a6d* |
| *Prkar1b* | *Rsrp1* | *A930012L18Rik* | *Cd69* |
| *Rex2* | *Fam162a* | *8430408G22Rik* | *BB287469* |
| *Pcdhb9* | *Tspo* | *2610528A11Rik* | *Akap3* |
| *Lpcat1* | *Ero1l* | *1810010H24Rik* | *Rhbg* |
| *Itgal* | *Zbtb7c* | *1600014C23Rik* | *Csprs* |
| *Scara3* | *P4ha2* |  | *Gbp4* |
| *Tmem50b* | *B4galt5* |  | *Slc5a1* |
| *Fndc7* | *1110008P14Rik* |  | *9930111J21Rik1* |
| *Bdh2* | *Tnf* |  | *Vmn2r3* |
| *Cdc42ep2* | *Gipr* |  | *Ifitm6* |
| *Gpi1* | *Zfp395* |  | *Vnn3* |
| *Hpdl* |  |  | *Il6* |
| *Rab17* |  |  | *Cyp2j6* |
| *Ackr3* |  |  | *Sp110* |
| *Hist2h2aa1* |  |  | *Ncf4* |
| *Chrnb1* |  |  | *Nup210* |
| *Ifi202b* |  |  | *Pkhd1l1* |
| *Nrn1* |  |  | *Enpp3* |
| *Fhl2* |  |  | *Dyrk4* |
| *Trp53cor1* |  |  | *Heph* |
| *Arrb1* |  |  | *Rgs17* |
| *Sprr1a* |  |  | *H2-M10.5* |
| *Hist2h2bb* |  |  | *Plekhh2* |
| *Epm2a* |  |  | *Serpina1e* |
| *1600029O15Rik* |  |  | *Selp* |
| *Hist3h2a* |  |  | *Cxcl1* |
| *Errfi1* |  |  | *Il12b* |
| *Csf1* |  |  | *Tfcp2l1* |
| *Mycbp* |  |  | *Sspo* |
| *Lhpp* |  |  | *Gm4070* |
| *Egr1* |  |  | *Itk* |
| *Hk1* |  |  | *Ccl3* |
|  |  |  | *Ostn* |
|  |  |  | *Slc9a9* |
|  |  |  | *Hephl1* |
|  |  |  | *Clec10a* |
|  |  |  | *Myh7b* |
|  |  |  | *Lrmp* |
|  |  |  | *Try5* |
|  |  |  | *Herc3* |
|  |  |  | *Dthd1* |
|  |  |  | *Parp10* |
|  |  |  | *Calcb* |
|  |  |  | *Trim30a* |
|  |  |  | *Cxcl5* |
|  |  |  | *Trim30d* |
|  |  |  | *Adamtsl3* |
|  |  |  | *Gm10436* |
|  |  |  | *Dqx1* |
|  |  |  | *Vnn1* |
|  |  |  | *Fetub* |
|  |  |  | *Inpp4b* |

**Supplementary Table S5.** Proteins differentially expressed in 5 and 6 Ni^2+^-treated groups.

| **Differentially expressed in 5 groups** | | | **Differentially expressed in 6 groups** |
| --- | --- | --- | --- |
| AARS | GDPD1 | RAB18 | ACOT2 |
| ACACA | Gm10020 | RAD50 | ASNS |
| ACO1 | HINT2 | RAP1B | CSF1 |
| ACOT9 | HIST1H1C | RARS | CTH |
| ACTR2 | Hist1h2af | RBPJ | CYP51 |
| ALDH18A1 | Hist1h4h | RPL10A | EPHA2 |
| ALDH3A2 | HSD17B10 | RPL12 | FDPS |
| ANXA3 | HTRA1 | RPL17 | FTH1 |
| ARPC1B | HYOU1 | RPL18A | GNPNAT1 |
| ASNA1 | IKBKAP | RPL19 | IGF2BP1 |
| ATP5L | Isoc2a | RPL30 | ITGAV |
| AVIL | KIFAP3 | RPL31 | KRT75 |
| BCAT1 | KRT1 | RPL38 | LBH |
| C1QBP | KRT2 | RPL4 | LGALSL |
| CDIPT | LAP3 | RPL7L1 | LSS |
| COL4A1 | LIPI | RPL8 | MAP1B |
| COL6A3 | LRRC59 | RPL9 | MIA3 |
| COPB1 | MAK16 | RPLP1 | MORF4L1 |
| CP | MTCH2 | RPS12 | NARS |
| Ctps | mthfd2 | RPS17 | RNF213 |
| DHFR | MYDGF | RPS18 | RPL21 |
| DLD | NAA25 | RPS26 | RPL22L1 |
| DNAJA2 | NIF3L1 | RPS27 | SLC1A4 |
| DNAJC21 | NNT | Rps3a1 | SPOUT1 |
| DPH2 | NOP16 | RPS5 | STARD5 |
| DPM1 | NUCB2 | SIGMAR1 | TCEB1 |
| EIF2B5 | OSTF1 | SLC25A12 | TNPO1 |
| EIF3L | OTUD6B | SLC3A2 | Uqcrb |
| EIF3M | PAPSS1 | SRI | UTP3 |
| EIF5 | PDAP1 | SRM | WARS |
| EMILIN1 | PHGDH | STOM | ZC3HAV1 |
| ERO1LB | PLCH1 | SURF6 |  |
| FAH | pmvk | TSR1 |  |
| FAM96B | PPP5C | TSR3 |  |
| FBXO22 | prkaa1 | TXNRD1 |  |
| FECH | PSAT1 | UFL1 |  |
| FKBP11 | PSPH | YARS |  |
| FOCAD | PTBP1 | YBX3 |  |
| G6pdx | PTK2 | Zfp207 |  |
| GARS | QARS |  |  |

**Supplementary Table S6:** Biological pathways involved by 7 candidate gene/protein biomarkers

| **No.** | **Gene symbol** | **Pathway** |
| --- | --- | --- |
| 1 | CSF1 | 1. **Cytokine-cytokine receptor interaction** 2. **MAPK signaling pathway** 3. **Ras signaling pathway** 4. **PI3K-Akt signaling pathway** 5. **Rap1 signaling pathway** 6. **TNF signaling pathway** 7. Alzheimer disease 8. Pathways of neurodegeneration - multiple diseases 9. Rheumatoid arthritis 10. Viral protein interaction with cytokine and cytokine receptor 11. Hematopoietic cell lineage 12. Osteoclast differentiation |
| 2 | ACOT2 | 1. **Biosynthesis of unsaturated fatty acids** 2. **Fatty acid elongation** 3. Metabolic pathways 4. Ovarian steroidogenesis |
| 3 | FAH | 1. Metabolic pathways 2. Tyrosine metabolism |
| 4 | LBH |  |
| 5 | HYOU1 | 1. Protein processing in endoplasmic reticulum |
| 6 | UQCRB | 1. **Oxidative phosphorylation** 2. Metabolic pathways 3. Alzheimer disease 4. Pathways of neurodegeneration - multiple diseases 5. Diabetic cardiomyopathy 6. Thermogenesis 7. Non-alcoholic fatty liver disease 8. Chemical carcinogenesis - reactive oxygen species 9. Parkinson disease 10. Amyotrophic lateral sclerosis 11. Prion disease 12. Huntington disease 13. Cardiac muscle contraction |
| 7 | CTH | 1. **Biosynthesis of amino acids** 2. **Cysteine and methionine metabolism** 3. **Glycine, serine and threonine metabolism** 4. Metabolic pathways 5. Selenocompound metabolism |

(A)

(B)

(C)

(D)

**Supplementary Figure S1:** The gel electropherogram: (A) CSF1, (B) CTH, (C) ACOT2, (D) UQCRB.

**Supplementary Table S7:** The semiquantitative data of CSF1.

| **Groups** | **GAPDH-1** | **GAPDH-2** | **GAPDH-3** | **CSF1-1** | **CSF1-2** | **CSF1-3** |
| --- | --- | --- | --- | --- | --- | --- |
| Untreated 1 | 103099 | 127771 | 80246 | 65819 | 43844 | 39798 |
| 100µM Ni^2+^-12h | 118875 | 121231 | 70565 | 25069 | 18585 | 22001 |
| 100µM Ni^2+^-24h | 115078 | 106232 | 67827 | 18427 | 18219 | 14326 |
| 100µM Ni^2+^-48h | 100398 | 119569 | 80927 | 28109 | 22473 | 16490 |
| 200µM Ni^2+^-12h | 112457 | 126776 | 81130 | 47829 | 27710 | 23586 |
| 200µM Ni^2+^-24h | 102315 | 115827 | 77050 | 49253 | 28599 | 26123 |
| 200µM Ni^2+^-48h | 103195 | 144634 | 90558 | 45911 | 24181 | 23601 |
| Untreated 2 | 472239 | 511123 | 282346 | 126439 | 118801 | 103486 |
| 100µM Ni^2+^-72h | 523257 | 496685 | 265715 | 82483 | 75507 | 82777 |
| 200µM Ni^2+^-72h | 547827 | 508291 | 277343 | 110892 | 97270 | 79652 |

**Supplementary Table S8:** The semiquantitative data of CTH.

| **Groups** | **GAPDH-1** | **GAPDH-2** | **GAPDH-3** | **CTH-1** | **CTH-2** | **CTH-3** |
| --- | --- | --- | --- | --- | --- | --- |
| Untreated | 389240 | 398794 | 281716 | 540850 | 387131 | 149317 |
| 100µM Ni^2+^-12h | 275115 | 322991 | 235133 | 451417 | 348925 | 134177 |
| 100µM Ni^2+^-24h | 384304 | 312128 | 271757 | 407650 | 251272 | 105022 |
| 100µM Ni^2+^-48h | 487290 | 386877 | 246657 | 271250 | 169037 | 90536 |
| 100µM Ni^2+^-72h | 396516 | 347528 | 285830 | 231484 | 183005 | 89298 |
| 200µM Ni^2+^-12h | 420951 | 304198 | 281287 | 375691 | 270596 | 111169 |
| 200µM Ni^2+^-24h | 464747 | 406048 | 300589 | 309073 | 205539 | 105709 |
| 200µM Ni^2+^-48h | 470283 | 454821 | 248471 | 305076 | 160273 | 89015 |
| 200µM Ni^2+^-72h | 464516 | 418130 | 304162 | 241673 | 189933 | 92201 |

**Supplementary Table S9:** The semiquantitative data of ACOT2.

| **Groups** | **GAPDH-1** | **GAPDH-2** | **GAPDH-3** | **ACOT2-1** | **ACOT2-2** | **ACOT2-3** |
| --- | --- | --- | --- | --- | --- | --- |
| Untreated | 389240 | 398794 | 281716 | 130998 | 171141 | 265763 |
| 100µM Ni^2+^-12h | 275115 | 322991 | 235133 | 144301 | 214272 | 304537 |
| 100µM Ni^2+^-24h | 384304 | 312128 | 271757 | 146349 | 170625 | 292556 |
| 100µM Ni^2+^-48h | 487290 | 386877 | 246657 | 139764 | 157503 | 276245 |
| 100µM Ni^2+^-72h | 396516 | 347528 | 285830 | 169130 | 232427 | 325800 |
| 200µM Ni^2+^-12h | 420951 | 304198 | 281287 | 146956 | 205915 | 324715 |
| 200µM Ni^2+^-24h | 464747 | 406048 | 300589 | 171804 | 204284 | 322157 |
| 200µM Ni^2+^-48h | 470283 | 454821 | 248471 | 140189 | 179431 | 289297 |
| 200µM Ni^2+^-72h | 464516 | 418130 | 304162 | 146755 | 180831 | 330695 |

**Supplementary Table S10:** The semiquantitative data of UQCRB.

| **Groups** | **GAPDH-1** | **GAPDH-2** | **GAPDH-3** | **UQCRB -1** | **UQCRB-2** | **UQCRB-3** |
| --- | --- | --- | --- | --- | --- | --- |
| Untreated 1 | 103099 | 127771 | 80246 | 51943 | 66004 | 49611 |
| 100µM Ni^2+^-12h | 118875 | 121231 | 70565 | 41279 | 44674 | 31696 |
| 100µM Ni^2+^-24h | 115078 | 106232 | 67827 | 30762 | 30031 | 22419 |
| 100µM Ni^2+^-48h | 100398 | 119569 | 80927 | 29854 | 32082 | 29108 |
| 200µM Ni^2+^-12h | 112457 | 126776 | 81130 | 32194 | 42614 | 36679 |
| 200µM Ni^2+^-24h | 102315 | 115827 | 77050 | 35822 | 35726 | 35325 |
| 200µM Ni^2+^-48h | 103195 | 144634 | 90558 | 26710 | 32293 | 26839 |
| Untreated 2 | 472239 | 511123 | 282346 | 410765 | 466895 | 144685 |
| 100µM Ni^2+^-72h | 523257 | 496685 | 265715 | 361940 | 368779 | 140309 |
| 200µM Ni^2+^-72h | 547827 | 508291 | 277343 | 224244 | 321377 | 107065 |
